# Supplementary figures and images for: PathoGFAIR: a collection of FAIR and adaptable (meta)genomics workflows for (foodborne) pathogens detection and tracking
Source: Gigascience. 2025 Sep 26;14:giaf017. doi: 10.1093/gigascience/giaf017 (PMC12466118; doi:10.1093/gigascience/giaf017)

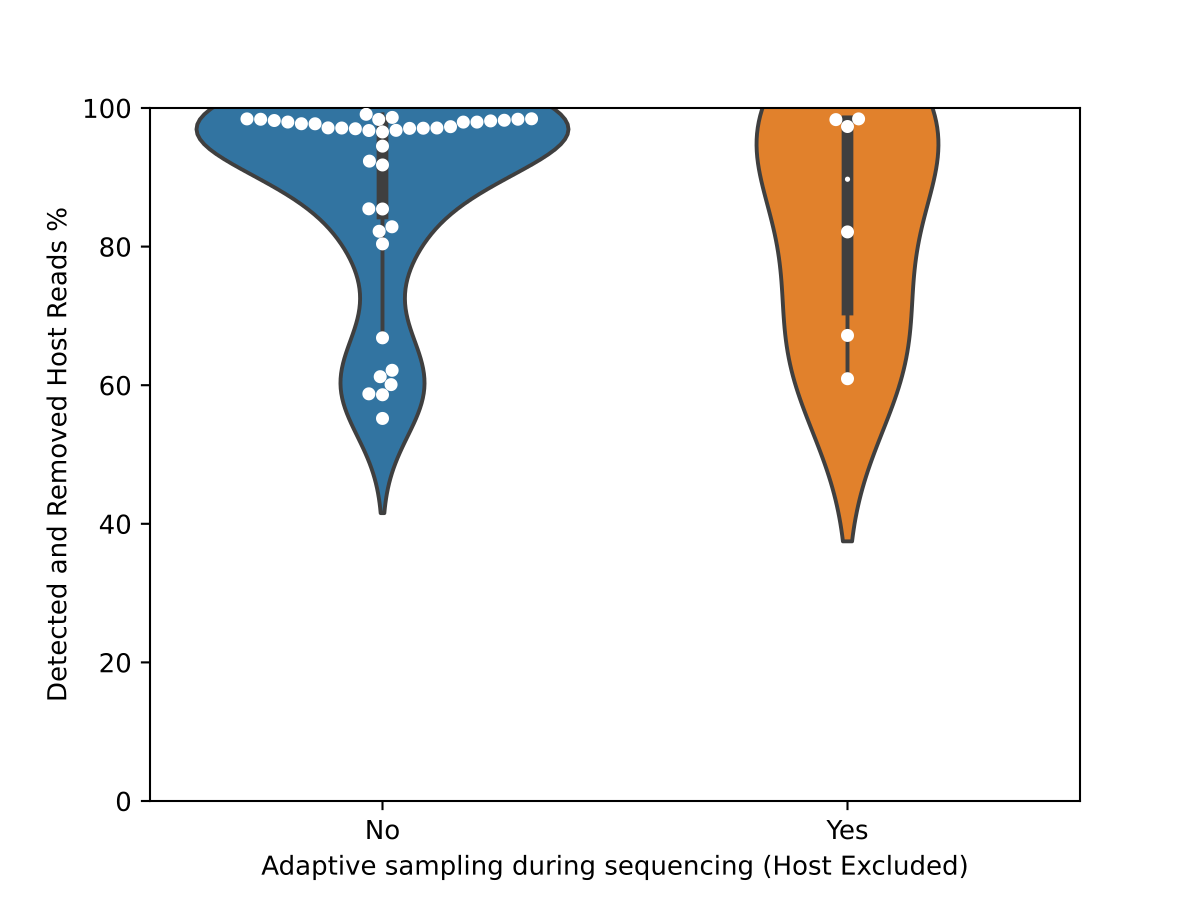

Supplement: giaf017_Supplemental_Files [file giaf017_supplemental_files.zip › Supplementary_Figure_S1.png]

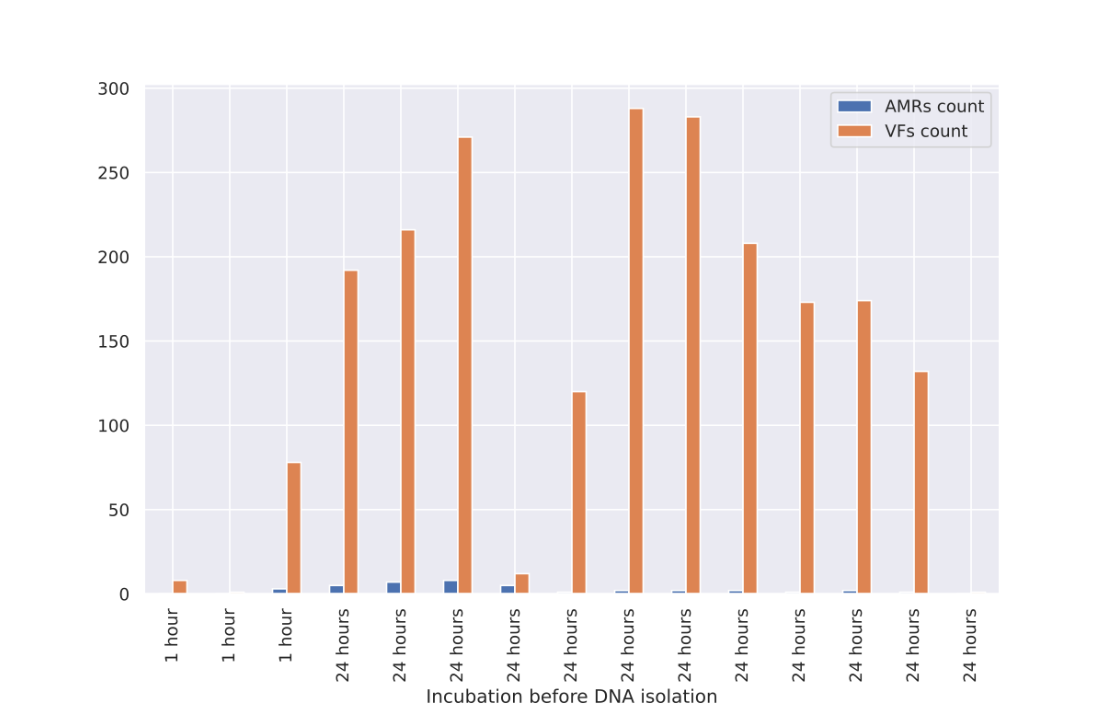

Supplement: giaf017_Supplemental_Files [file giaf017_supplemental_files.zip › Supplementary_Figure_S3.png]

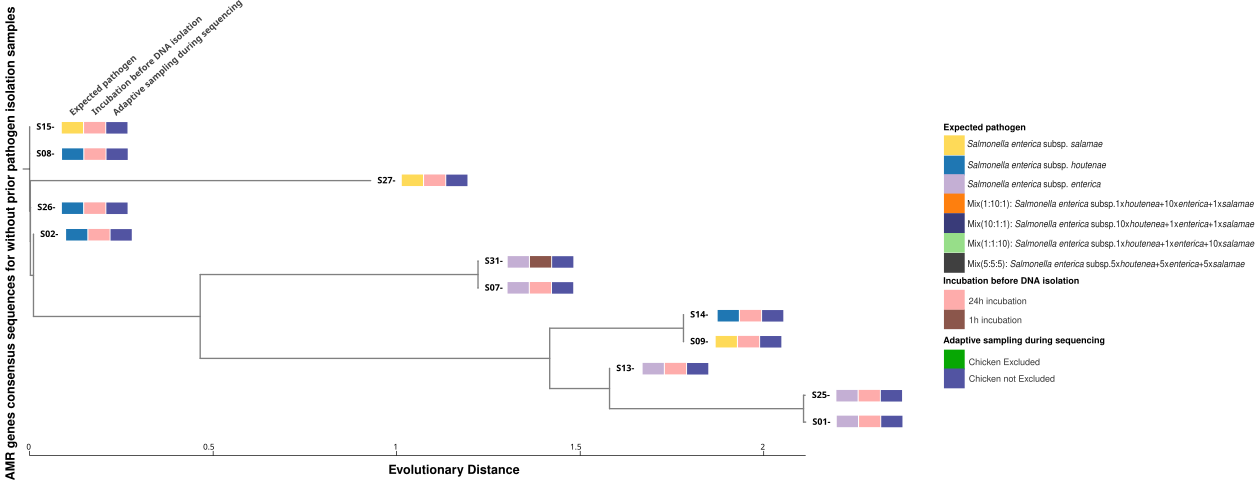

Supplement: giaf017_Supplemental_Files [file giaf017_supplemental_files.zip › Supplementary_Figure_S4.png]

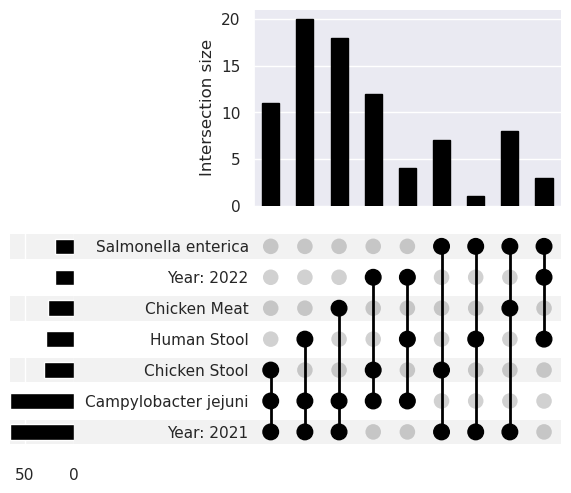

Supplement: giaf017_Supplemental_Files [file giaf017_supplemental_files.zip › Supplementary_Figure_S5.png]

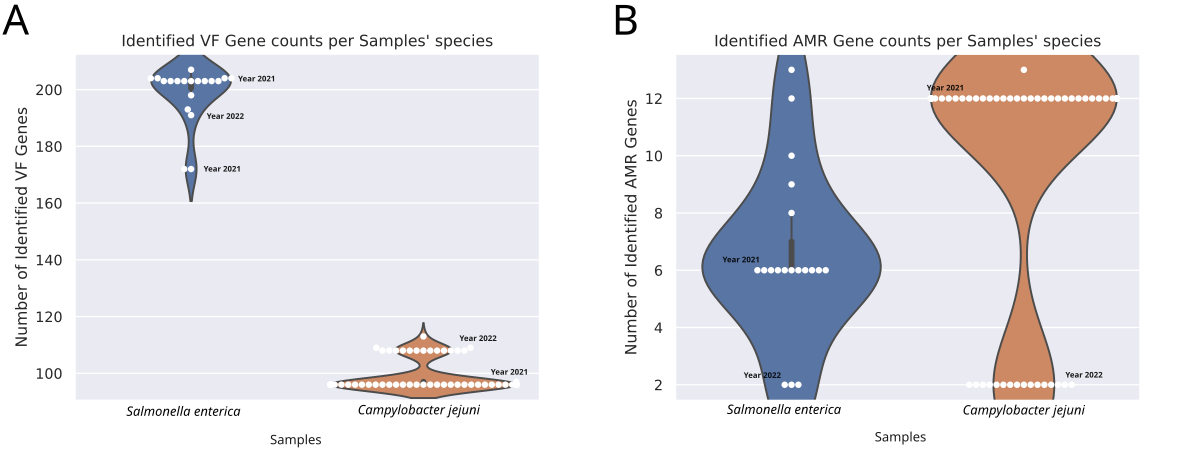

Supplement: giaf017_Supplemental_Files [file giaf017_supplemental_files.zip › Supplementary_Figure_S7.png]

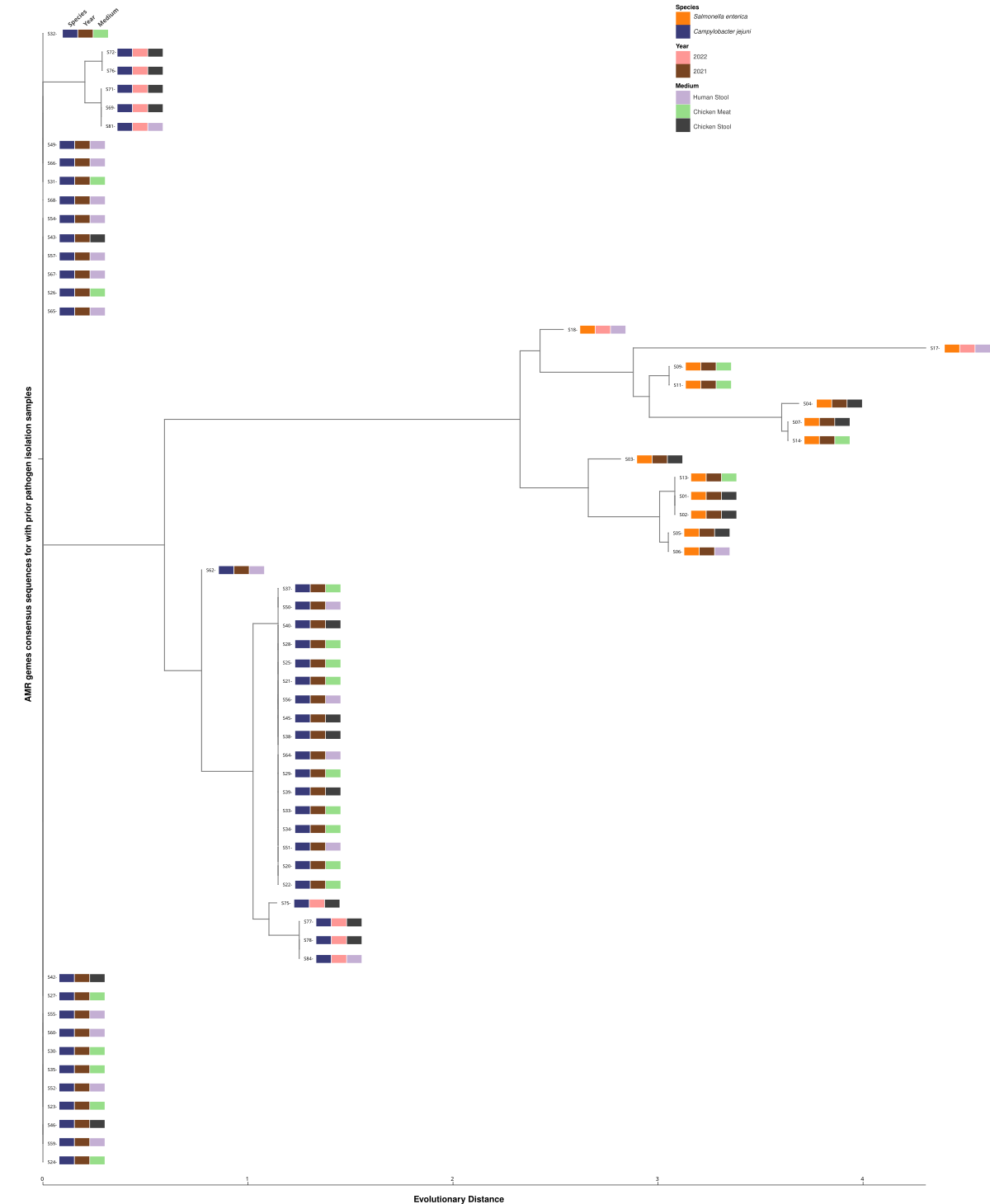

Supplement: giaf017_Supplemental_Files [file giaf017_supplemental_files.zip › Supplementary_Figure_S8.png]

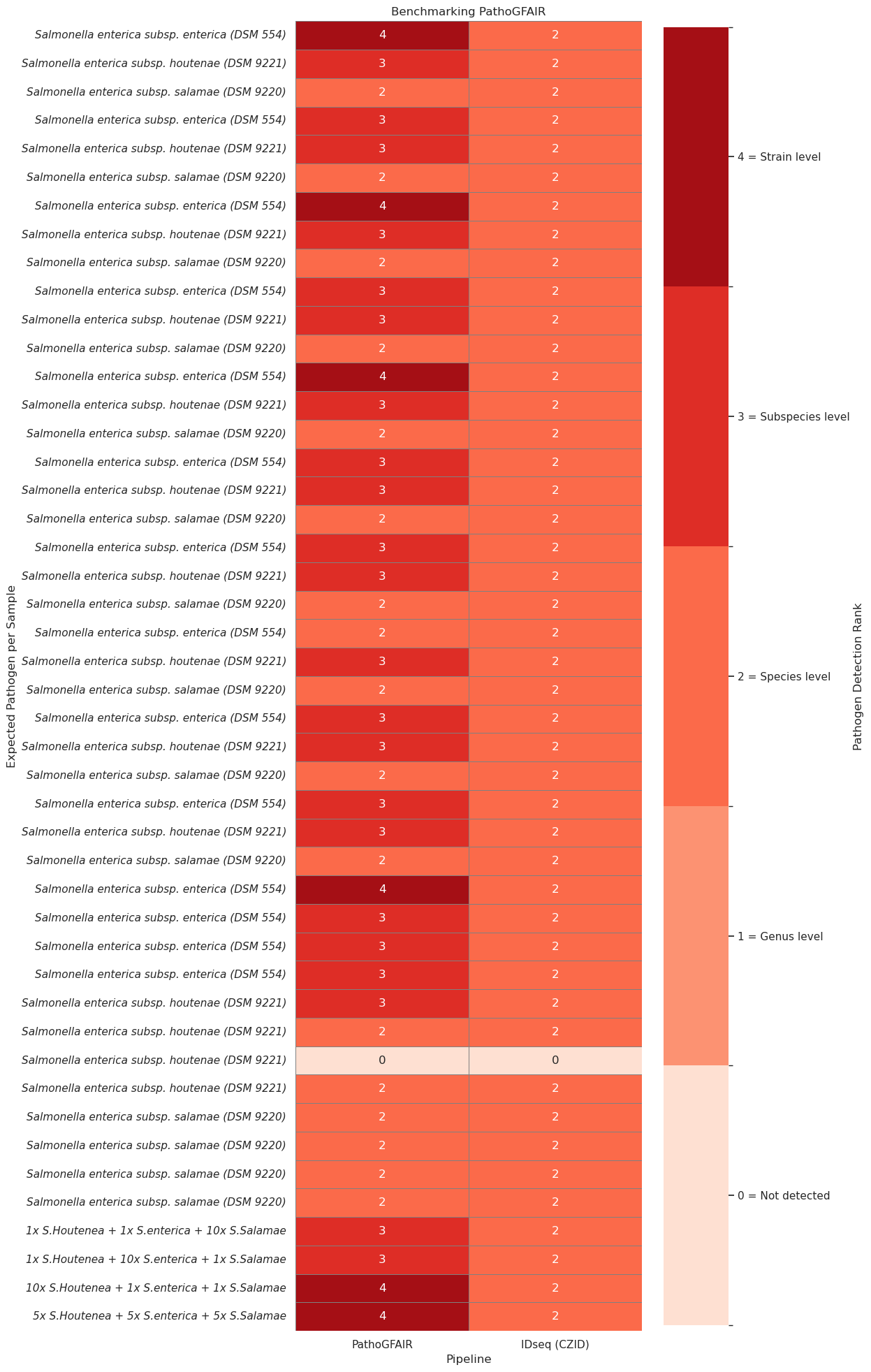

Supplement: giaf017_Supplemental_Files [file giaf017_supplemental_files.zip › Supplementary_Figure_S9.png]
