# Supplementary material for: PathoGFAIR: a collection of FAIR and adaptable (meta)genomics workflows for (foodborne) pathogens detection and tracking
Source: Gigascience. 2025 Sep 26;14:giaf017. doi: 10.1093/gigascience/giaf017 (PMC12466118; doi:10.1093/gigascience/giaf017)

GigaScience

PathoGFAIR: a collection of FAIR and adaptable (meta)genomics workflows for (foodborne) pathogens detection and tracking

--Manuscript Draft--

**Manuscript Number:**	GIGA-D-24-00270
**Full Title:**	PathoGFAIR: a collection of FAIR and adaptable (meta)genomics workflows for (foodborne) pathogens detection and tracking
**Article Type:**	Technical Note
**Funding Information:**	EOSC-Life — Dr Bérénice Batut<br>Bundesministerium für Bildung, Wissenschaft und Forschung (031 A538A de.NBI-RBC) — Dr Björn Grüning<br>Ministerium für Wissenschaft, Forschung und Kunst Baden-Württemberg — Dr Björn Grüning<br>Agence Nationale de la Recherche (ANR-11-INBS-0013) — Not applicable
**Abstract:**	Background: Food contamination by pathogens poses a global health threat, affecting an estimated 600 million people annually. During a foodborne outbreak investigation, microbiological analysis of food vehicles detects responsible pathogens and traces contamination sources. Metagenomic approaches offer a comprehensive view of the genomic composition of microbial communities, facilitating the detection of potential pathogens in samples. Combined with sequencing techniques like Oxford Nanopore sequencing, such metagenomic approaches become faster and easier to apply. A key limitation of these approaches is the lack of accessible, easy-to-use, and openly available pipelines for pathogen identification and tracking from (meta)genomic data.<br>Findings: PathoGFAIR is a collection of Galaxy-based FAIR workflows employing state-of-the-art tools to detect and track pathogens from metagenomic Nanopore sequencing. Although initially developed for foodborne pathogen data, the workflows can be applied to any metagenomic Nanopore pathogenic data. PathoGFAIR incorporates visualisations and reports for comprehensive results. We tested PathoGFAIR on 130 benchmark samples containing different pathogens from multiple hosts under various experimental conditions. Workflows have successfully detected and tracked expected pathogens at least at the species rank in both pathogen-isolated and non-pathogen-isolated samples with sufficient Colony-forming unit and Cycle Threshold values.<br>Conclusions: PathoGFAIR detects the pathogens or the subspecies of the pathogens in any sample, regardless of whether the sample is isolated or incubated before sequencing. Importantly, PathoGFAIR is easy to use and can be straightforwardly adapted and extended for other types of analysis and sequencing techniques, making it usable in various pathogen detection scenarios.
**Corresponding Author:**	Bérénice Batut, Ph.D.<br>CNRS UAR 3601: Institut Français de Bioinformatique<br>Evry, FRANCE
**Corresponding Author Secondary Information:**	
**Corresponding Author's Institution:**	CNRS UAR 3601: Institut Français de Bioinformatique
**Corresponding Author's Secondary Institution:**	
**First Author:**	Engy Nasr
**First Author Secondary Information:**	
**Order of Authors:**	Engy Nasr<br>Anna Henger, Ph.D.<br>Björn Grüning, Ph.D.

Powered by Editorial Manager® and ProduXion Manager® from Aries Systems Corporation

	Paul Zierep, Ph.D.
	Bérénice Batut, Ph.D.
**Order of Authors Secondary Information:**	
**Additional Information:**	
**Question**	**Response**
Are you submitting this manuscript to a special series or article collection?	No
**Experimental design and statistics**<br><br>Full details of the experimental design and statistical methods used should be given in the Methods section, as detailed in our Minimum Standards Reporting Checklist. Information essential to interpreting the data presented should be made available in the figure legends.<br><br>Have you included all the information requested in your manuscript?	Yes
**Resources**<br><br>A description of all resources used, including antibodies, cell lines, animals and software tools, with enough information to allow them to be uniquely identified, should be included in the Methods section. Authors are strongly encouraged to cite Research Resource Identifiers (RRIDs) for antibodies, model organisms and tools, where possible.<br><br>Have you included the information requested as detailed in our Minimum Standards Reporting Checklist?	Yes
**Availability of data and materials**<br><br>All datasets and code on which the conclusions of the paper rely must be either included in your submission or deposited in publicly available repositories (where available and ethically appropriate), referencing such data using	Yes

a unique identifier in the references and in the "Availability of Data and Materials" section of your manuscript.<br><br>Have you have met the above requirement as detailed in our Minimum Standards Reporting Checklist?	

LaTeX - Main Document (TeX file)

Click here to access/download;LaTeX - Main Document (TeX file);main.tex

Placeholder for OUP logo oup.pdf

GigaScience, 2023, 1–12

doi: xx.xxxx/xxxx
Manuscript in Preparation
Technical Note

TECHNICAL NOTE

PathoGFAIR: a collection of FAIR and adaptable (meta)genomics workflows for (foodborne) pathogens detection and tracking

Engy Nasr[1], Anna Henger[2], Björn Grüning[1], Paul Zierep[1] and Bérénice Batut[3,4,*]

[1]Bioinformatics Group, Department of Computer Science, University of Freiburg, Georges-Koehler-Allee 106, 79110 Freiburg im Breisgau, Germany and [2]Biolytix AG, 4243 Dittingen, Switzerland and [3]CNRS, Institut Français de Bioinformatique, IFB-Core, UAR 3601, 91000 Evry, France and [4]Plateforme AuBi, Mésocentre Clermont-Auvergne, Université Clermont Auvergne, 63170 Aubière, France

*berenice.batut@gmail.com

Abstract

Background: Food contamination by pathogens poses a global health threat, affecting an estimated 600 million people annually. During a foodborne outbreak investigation, microbiological analysis of food vehicles detects responsible pathogens and traces contamination sources. Metagenomic approaches offer a comprehensive view of the genomic composition of microbial communities, facilitating the detection of potential pathogens in samples. Combined with sequencing techniques like Oxford Nanopore sequencing, such metagenomic approaches become faster and easier to apply. A key limitation of these approaches is the lack of accessible, easy-to-use, and openly available pipelines for pathogen identification and tracking from (meta)genomic data.
Findings: PathoGFAIR is a collection of Galaxy-based FAIR workflows employing state-of-the-art tools to detect and track pathogens from metagenomic Nanopore sequencing. Although initially developed for foodborne pathogen data, the workflows can be applied to any metagenomic Nanopore pathogenic data. PathoGFAIR incorporates visualisations and reports for comprehensive results. We tested PathoGFAIR on 130 benchmark samples containing different pathogens from multiple hosts under various experimental conditions. Workflows have successfully detected and tracked expected pathogens at least at the species rank in both pathogen-isolated and non-pathogen-isolated samples with sufficient Colony-forming unit and Cycle Threshold values.
Conclusions: PathoGFAIR detects the pathogens or the subspecies of the pathogens in any sample, regardless of whether the sample is isolated or incubated before sequencing. Importantly, PathoGFAIR is easy to use and can be straightforwardly adapted and extended for other types of analysis and sequencing techniques, making it usable in various pathogen detection scenarios.

Key words: Galaxy; Public health; Nanopore; Pipeline; Open source; Benchmark samples; Visualisation

Introduction

Foodborne pathogens pose a significant threat to public health worldwide, causing millions of cases of illness and even death every year [1, 2]. These diverse microorganisms, spanning bacteria, viruses, parasites, and fungi, can contaminate a variety of foods, leading to outbreaks and epidemics. The impact of foodborne pathogens on public health is a critical concern, and efforts to mitigate their spread and ensure food safety are of great importance. Food safety and controlling foodborne pathogens have been key priorities for global public health authorities [3].

Traditional methods for identifying the source of food contamination require isolation of the target pathogen. This process is not only time-consuming but can be complex and lacks a guaranteed success rate [4]. In contrast, shotgun metagenomic approaches provide a solution to these challenges, as they give an overview of the genomic composition in the sample, including the food source itself, the microbial community, and any possible pathogens and their

Compiled on: July 5, 2024.
Draft manuscript prepared by the author.

complete genetic information [5]. Importantly, shotgun metagenomic approaches eliminate the need for prior isolation of the targeted pathogen, as required by Whole Genome Sequencing (WGS) methods, and they are not limited to specific genes as opposed to real-time PCR approaches [6].

The utilisation of Nanopore sequencing data, as exemplified in studies like [7], enhances the capabilities of metagenomic approaches for outbreak investigations. Nanopore sequencing provides real-time, long-read data that can capture comprehensive genetic information, allowing for more accurate and rapid pathogen detection. This advancement becomes particularly crucial in outbreak scenarios where timely responses are essential.

Once (meta)genomics data has been generated, bioinformatics approaches enable the rapid and accurate detection, as well as the identification of genetic variations and potential Virulence Factor (VF) genes of pathogens [8, 9]. However, using already available tools and workflows requires bioinformatic and computational knowledge and expertise. Tool arguments and parameters need to be adapted to the specific use case. End-to-end workflows and platforms (1) that allow users to analyse their samples are either restricted, require high computational resources, or paid subscription (e.g. SURPI [10], OneCodex [11], Sunbeam [12]). For some of the free resources, the underlying workflow is not available and adaptable for the user. For example, IDseq [13] (also known as CZID [14]), a free cloud-based service for pathogen detection can only be externally accessed through the dedicated online user interface. Furthermore, some of these workflows are specific to a certain host, pathogen, or sequencing technique, lacking the flexibility for customisation.

Galaxy [15] is an open-source platform for FAIR data analysis. It enables users to apply a comprehensive suite of bioinformatics tools (that can be combined into workflows) through either its user-friendly web interface or its automatable Application Programming Interface (API) for integrating and customising workflows, enhancing user flexibility. It ensures reproducibility by capturing the necessary information to repeat and understand data analyses. Galaxy offers a collection of high-quality pre-built workflows that can be either used directly or are easily adapted to the user's needs via the Galaxy workflow editor. Galaxy workflows can be executed on any Galaxy server, even on the private Galaxy server, making it suitable also for data where privacy concerns are important. Furthermore, Galaxy via the major public servers [15] freely provides a large computing infrastructure allowing for the execution of computationally challenging workflows, which is often the case for metagenomic analysis.

Here, we present PathoGFAIR, a collection of Galaxy-based workflows for pathogen identification and tracking from (meta)genomics Oxford Nanopore sequencing data. The workflows are openly available on two workflow registries; Dockstore [16] and WorkflowHub [17]. They can be used directly on three major Galaxy servers (usegalaxy.org, usegalaxy.eu, usegalaxy.org.au) or installed in any other Galaxy server. The workflows are created to work agnostically, detecting all pathogens present in the samples without prior knowledge of the target pathogen. As the workflows are created in Galaxy, they can be adapted, e.g. for other sequencing techniques or with various downstream analyses, such as differential expression analysis, or further statistics and visualisations [15]. Workflows are documented and supported by an extensive tutorial freely available via the Galaxy Training Network (GTN) [18]. Overall, PathoGFAIR offers an easy-to-use computational solution that speeds up the process of sampling, detecting, and tracking pathogens. Links to workflows and tutorials can be found on PathoGFAIR homepage: https://usegalaxy-eu.github.io/PathoGFAIR/

Implementation

Overview

PathoGFAIR comprises a collection of 5 workflows, implemented in Galaxy (Figure 1). Each workflow serves a specific function and can be executed independently, enabling users to tailor their analysis according to their requirements.

The input data for PathoGFAIR comprises sequencing data generated using Oxford Nanopore technologies, along with a metadata table describing the datasets. The datasets undergo preprocessing in Workflow 1, which includes quality control and host removal procedures.

Subsequently, the preprocessed data is directed to three parallel workflows: taxonomy profiling (Workflow 2), gene-based pathogen identification (Workflow 3), and allele-based pathogen identification (Workflow 4). This parallel execution allows for efficient analysis and flexibility in workflow selection. Notably, Workflow 4 can optionally synchronise with Workflow 2 or Workflow 3 to leverage prior taxonomic analysis or gene-based pathogen identification results, providing users with flexibility based on specific use cases. By using detailed taxonomic identification from Workflow 2 or gene-based pathogen identification from Workflow 3, Workflow 4 enhances mapping and SNP detection accuracy and efficiency. This process involves selecting the correct reference genome of the pathogen for mapping, informed by results from Workflow 2, Workflow 3, or even Workflow 1, which performs initial taxonomy assignment during the host filtering step.

Since each workflow can be executed independently, users can focus on specific aspects of pathogen detection or analysis. This modular approach empowers users to utilise the full range of functions offered by each workflow individually or to combine them as needed for comprehensive pathogen detection.

Finally, in Workflow 5, outputs from the previous workflows and the metadata of the dataset are aggregated and visualised for comprehensive pathogen tracking across samples. This aggregation step ensures a holistic view of pathogen presence and distribution, facilitating further insights and analysis.

Overall, the independent nature of PathoGFAIR's workflows provides users with a user-friendly and customisable approach to pathogen detection, allowing for both comprehensive analyses and targeted investigations based on specific research needs or objectives.

PathoGFAIR offers a competitive, and accessible solution (Table 1) to detect and track pathogens in metagenomic Nanopore data through its five Galaxy-based FAIR and customisable workflows.

Workflow 1: Preprocessing

Workflow 1 encompasses essential preprocessing steps to ensure the quality and integrity of sequencing data.

Quality control and sequence filtering, based on quality, length, or low complexity, are performed using Fastp (v 0.23.2) [22]. Porechop (v 0.2.4) [23] trims low-quality base pairs and removes duplicates and adapters.

Quality-controlled (QC) reads are cleaned of sequences from the food vehicle animal or infected host by mapping to their reference genome using Minimap2 (v 2.26) (RRID:SCR_018550) [24], a tool tens of times faster than mainstream long-read mappers such as BLASR [25], BWA-MEM [26], NGMLR [27] and GMAP [28] and three times as fast as Bowtie2 [29] designed for Illumina short reads [24]. A variety of reference genomes (e.g. Human, Chicken, or Cow) can be installed on Galaxy servers to work with Minimap2. Kraken2 (v 1.2) [30] is applied for further contamination detection using the Kalamari database. The Kalamari database includes mitochondrial sequences of various known hosts including food hosts [31]. Reads matched to the Kalamari database are assessed and removed using Krakentools (v 1.2) [30].

The workflow returns QC reads without contamination or

Table 1. Comparison table between PathoGFAIR and other similar pipelines or systems. This comparison sheds light on various features and characteristics, such as accessibility, technical specifications, and the scope of analyses offered by each system. It serves as a reference for users to evaluate the suitability of PathoGFAIR for their specific needs and requirements

Features	PathoGFAIR	IDseq	SURPI	OneCodex	Sunbeam	Innuendo [19]	PAIPline [20]	Victors [21]
**General Characteristics**								
Free of Charge	✓	✓	✓	✗	✓	✓	✓	✓
Open Source Code	✓	✗	✓	✗	✓	✓	✓	✗
Web Interface	✓	✓	✗	✓	✗	✓	✗	✓*
Automatable API	✓	✗	✗	✗	✓	✓	✗	✗
**Accessibility and Availability**								
Simple end-user Modification	✓	✗	✗	✗	✗	✗	✗	✗
Publicly Available Web-server	✓	✓	✗	✓	✗	✗	✗	✓
Last Updated	2024	2023	2014	2023	2024	2018	2018	2019
**User Support and Documentation**								
Tutorial	✓	✗	✗	✗	✗	✗	✗	✗
Documentation	✓	✗	✗	✗	✓	✓	✗	✓
User support	✓	✗	✗	✗	✗	✗	✗	✗
**Technical Specifications**								
Workflow Manager	Galaxy	-	-	-	Snakemake	Nextflow	-	-
Sequencing Technique	Nanopore**	Illumina & Nanopore	Illumina	-	Illumina	-	Illumina	-
**Analyses**								
Preprocessing	✓	✓	✓	✓	✓	✓	✓	-
Taxonomy Profiling	✓	✓	✓	✓	✓	✓	✓	✗
Gene-based Pathogen Identification	✓	✓	✗	✗	✗	✓	✗	✓
Allele-based Pathogen Identification	✓	✗	✗	✗	✗	✗	✗	✗
Samples aggregation and Visualisations	✓	✓	✗	✓	✗	✗	✗	✗

* Malfunctioned when tested.
** Can be easily adapted to any other types of sequencing techniques via Galaxy, a customisable and automatable API.

host sequences as well as interactive reports, produced by FastQC (v 0.12.1) (RRID:SCR_014583), fastp and MultiQC (v 1.11) (RRID:SCR_014982) [32]. Furthermore, Nanoplot (v 1.39.0) [33] is employed to provide detailed quality metrics specifically tailored to the preprocessing step, enriching the suite of analytical insights and facilitating robust data evaluation.

Workflow 2: Taxonomy Profiling

The microbial communities are profiled, for QC reads from Workflow 1, using Kraken2 (v 1.2) [30] and the PlusPF (archaea, bacteria, viral, plasmid, human, UniVec_Core, protozoa, fungi, and plant) Refseq database (June 7, 2022). Although Kraken2 is a tool designed for short-read sequencing and is known for its false positive taxonomy assignments, particularly at lower microbial abundances [34], its application to long-reads can still yield a substantial overview of the microbial community. This is particularly true for discerning bacteria that could potentially be pathogenic at genus and species taxonomic ranks [35, 36]. The produced community profile is visualised using Krona (RRID:SCR_012785) [37] and observed interactively for different taxonomic ranks using Phinch [38] or Pavian [39].

Workflow 3: Gene-based Pathogen Identification

In this workflow, the pathogens are identified by the presence of genes associated with pathogenicity. QC reads from Workflow 1 are assembled into contigs using Metaflye (v 2.9.1) (RRID:SCR_017016) [40]. The contigs are then polished using the Medaka Consensus Pipeline (v 1.7.2) [41], which generates consensus sequences using neural networks and shows improved accuracy over graph-based approaches for Oxford Nanopore reads. The polished contigs are afterwards screened using ABRicate (v 1.0.1) [42] for virulence factors (VF) with the Virulence Factor DataBase (VFDB) [43] and for antimicrobial resistance (AMR) genes withAMRFinderPlus [44] database.

Workflow 4: Allele-based Pathogen Identification

Another approach to identifying pathogens is to use an allelic approach by detecting SNPs, i.e. markers showing evolutionary histories of homogeneous strains [45]. This process includes SNP calling, aimed at identifying novel pathogen strains and elucidating discrepancies compared to reference sequences, thereby facilitating the tracking of emerging variants. Within Workflow 4, both complex variants and SNPs are discerned, serving as crucial elements for subsequent pathogen identification and variant tracking purposes.

QC reads from Workflow 1 are mapped using Minimap2 (v 2.26) to a selected reference genome of a suspected pathogen. Users can choose the reference genome based on their prior knowledge of the target pathogen, the taxonomic analysis in Workflow 2, or the detected pathogenic genes in Workflow 3. Variant calling for mapped reads is performed using Clair3 (v 0.1.12) [46]. Clair3, a tool developed for long reads, has been chosen because it is demonstrated to be faster and more accurate than the Medaka variant pipeline, which its developer has declared deprecated in favour of Clair3 [41]. After that, all complex variants along with their information, such as type, genomics position, and quality score, are normalised using bcftools norm (v 1.9) [47]. The normalised reads are filtered using SnpSift filter (v 4.3) (RRID:SCR_015624) [48] based on the SNP quality computed in the SNPs identification step with Clair3. Filtered variants fields required for further analyses are extracted using SnpSift extract fields (v 4.3) (RRID:SCR_015624) [48]. Finally, a consensus sequence for each sample is built using bcftools consensus (v 1.9) (RRID:SCR_005227) [49]. In addition to the variants,

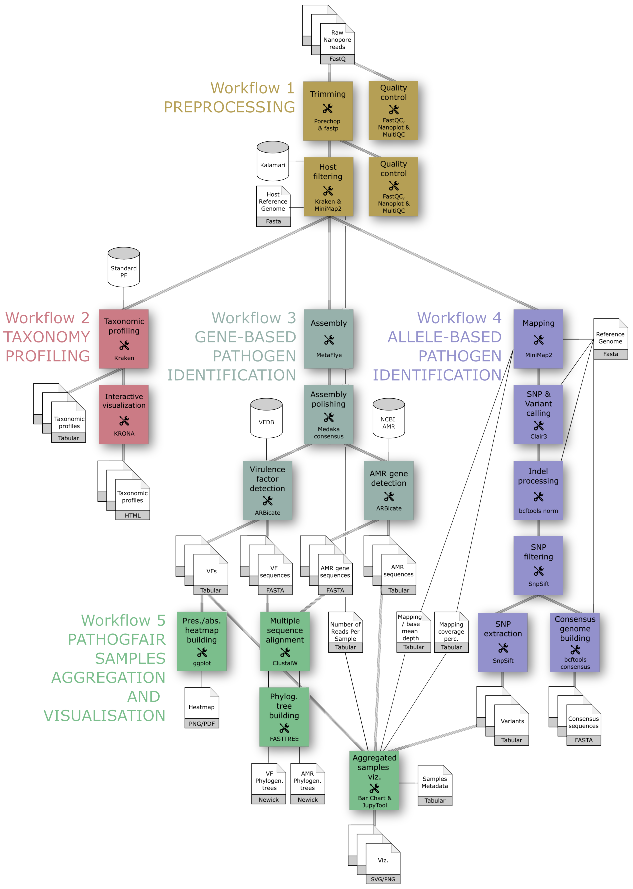

Figure 1. Flowchart of the PathoGFAIR workflows. Workflow 1 (olive green) takes as input sequencing data generated by Oxford Nanopore technologies and performs quality control and host filtering. Then three parallel workflows are executed on the output of Workflow 1: Workflow 2 (red) for taxonomy profiling, Workflow 3 (dark cyan) for gene-based pathogen identification, and Workflow 4 (purple) for SNP-based pathogen identification. These four workflows can run individually and in parallel. Finally, all outputs for the different provided datasets are aggregated in Workflow 5 (green) for PathoGFAIR Samples Aggregation and Visualisation.

this workflow outputs tables including summary metrics like the mapping coverage (breadth of coverage) percentages for every sample, per base covering mean depth (depth of covering), and quality filtered complex variants and SNPs numbers.

Workflow 5: PathoGFAIR Samples Aggregation and Visualisation

In all previously described workflows, individual samples are analysed separately. Workflow 5 consolidates the outputs from Workflows 1, 2, 3, and 4 along with sample metadata to generate various visualisations and reports. These reports illustrate the detected pathogens and facilitate the visualisation and tracking of their presence across all samples.

Virulence Factor (VF) tables from Workflow 3 are used to generate clustered heatmaps showing the VF genes using ggplot2 Heatmap (v 3.4.0) (RRID:SCR_014601). VF sequences are concatenated per sample and aligned over all samples using ClustalW (v 2.1). A phylogenetic tree of the virulence gene sequences is then generated from the multiple sequence alignment using FASTTREE (v 2.1.10) (RRID:SCR_015501) [50] and visualised using Newick Display (v 1.6). The same is performed on the antimicrobial resistance (AMR) tables from Workflow 3. From Workflows 1 and 4 output tables, bar charts are generated.

Other outputs are aggregated and processed within a Jupyter Notebook [51], interactively launched in Galaxy using JupyTool (v 1.0.0). This Notebook showcases the integration of sample metadata to generate analysis-specific plots, leveraging Python (v 3.10.12) libraries such as Pandas (v 1.5.3), Matplotlib (v 3.7.1), Seaborn (v 0.12.2), and Numpy (v 1.24.3) [52]. Examples of these plots include bar plots illustrating the number of reads before and after quality control for all samples, scatter plots visualising relationships between different variables such as pathogen count and sample characteristics, and interactive cluster maps displaying the clustering patterns of samples based on pathogen composition. These visualisation techniques are further elucidated and exemplified in the Use Cases section of this study, where the output tables from the workflows are aggregated with the corresponding sample metadata and visualised to facilitate comprehensive visual analysis.

Workflow Reports

As all PathoGFAIR workflows are designed to run seamlessly on the Galaxy platform, an interactive report is automatically generated upon completion of each workflow. These reports provide a comprehensive overview of the respective workflow's inputs and outputs. In PathoGFAIR, special attention has been given to refining these reports for enhanced user experience. The reports are carefully curated to automatically showcase and emphasise only the most informative, easily interpretable, and accessible outputs for each workflow. This ensures that users can efficiently extract key insights from the results, facilitating a streamlined and user-friendly analysis experience.

Easily Adaptable Workflows

The workflows can process raw shotgun (meta)genomics sequencing data from any sample, not only food.

PathoGFAIR has been initially developed to take Oxford Nanopore data as inputs. However, PathoGFAIR can work with Illumina data or other types of sequencing technique data. To adapt to Illumina sequencing only one tool needs to be changed in Workflow 1: Porechop [23] with Cutadapt (RRID:SCR_011841) [53]. Workflows 2, 3, 4, and 5 can be used directly with Illumina datasets without any adaptation. Some tools can be changed based on the tool's known performance towards short and long reads, such as Clair3 (v 0.1.12) [46] and Metaflye (v 2.9.1) [40]. All the mentioned tools are accessible within Galaxy, allowing for seamless interchangeability.

The workflows can also be adapted to process paired-end reads, by adjusting the tools' parameters to take paired-end read samples instead of single-end reads. These changes can be applied with little effort by using the user-friendly workflow editor in Galaxy.

Users can seamlessly switch between different host reference genomes and Kraken2 databases, as PathoGFAIR supports various pre-installed databases on the Galaxy servers. This feature enhances user convenience and efficiently explores different configurations to suit specific analysis requirements.

Similarly, tool versions and parameters can be adapted, e.g. to compare results with legacy versions of the workflows. New tool versions are automatically installed on public Galaxy servers using a sophisticated update infrastructure, ensuring a straightforward mechanism to keep the infrastructure up-to-date [54]. Every time a tool is updated, an update of the workflows is suggested, tested with functional tests, and released on the workflow registries once accepted.

Each of the five PathoGFAIR workflows is designed for a distinct type of analysis. Workflows 2, 3, and 4 operate independently, offering the flexibility to run them concurrently or skip them as per user requirements. This modular structure allows users to tailor the analysis to their specific needs, activating only the functionalities necessary for the desired workflow outcome.

FAIR Workflows

The FAIR principles [55], which emphasise the importance of making research objects Findable, Accessible, Interoperable, and Reusable, offer valuable guidance for optimising the utility and promoting the reproducibility and reusability of any research object (data, software [56], or workflows).

PathoGFAIR has been developed with the FAIR principle in mind and follows the ten tips for building FAIR workflows, as suggested by de Visser *et al.* [57]. First, by using Galaxy as a workflow manager, the workflows are portable (Tip 6) and come with a reproducible computational environment (Tip 7). The tools integrated into the workflows use file format standards such as FASTA and FASTQ for sequence data, SAM and BAM from the Samtools project for alignment data, VCF for genetic variations, GenBank and GFF3 for genomic annotations, and PDB for structural data (Tip 5) [55]. As explained in the previous section, the workflows are provided with default values (Tip 8) and are modular (Tip 9).

The workflows are available on the GitHub repository of IWC, the Intergalactic Workflow Commission of the Galaxy community (Tip 3) [58]. Workflows in this repository are reviewed and tested using test data before publication and with every new Galaxy release. The IWC automatically updates the workflows whenever a new version of any tool used in these workflows is released. Deposited workflows follow best practices, are versioned using GitHub releases, and contain important metadata (e.g. License, Author, Institutes) (Tip 2). The workflows are automatically added to two workflow repositories (Dockstore [16] and WorkflowHub [17]) to facilitate the discovery and re-use of workflows in an accessible and interoperable way (Tip 1). Via Dockstore or WorkflowHub, the PathoGFAIR workflows can be installed on any up-to-date Galaxy server. They are already publicly available on three main Galaxy servers (usegalaxy.org, usegalaxy.eu, usegalaxy.org.au), which any user can use and modify without restriction.

A thorough explanation of how to use the workflows in PathoGFAIR including a more global description of pathogen identification from Oxford Nanopore data can be found in a dedicated extensive tutorial [59] together with example input data and results (Tips 4 and 10), freely available and hosted via the Galaxy Training Network (GTN) [18] infrastructure.

Finally, for every invocation of the workflows, a Research Object Crate (RO-Crate [60, 61]) can be created to store the data products of the different steps, along with the run-associated metadata (including parameters, tool, and workflow version).

Use Cases

To demonstrate PathoGFAIR and its features, 130 samples from two studies (without or with prior pathogen isolation) were analysed. All samples contained pathogens known beforehand and were sequenced using Oxford Nanopore technology. All workflows of PathoGFAIR were evaluated for their main intended tasks, e.g., the preprocessing workflow for its reads quality retaining and hosts sequences removal performance, but also for their ability to identify the correct pathogen, and how well the accuracy with respect to different sampling conditions is.

Samples Without Prior Pathogen Isolation

Data Generation

In this study, 46 samples have been prepared given the following protocol [62]. Chicken meat was spiked with either one of three *Salmonella Enterica* subspecies (Salmonella enterica subsp. Houtenae DSM 9221, Salmonella enterica subsp. Enterica DSM 554, Salmonella enterica subsp. Salamae DSM 9220) or a mix of them, with concentrations that give Cycle Threshold (Ct) values between 25 and 33. 15 samples were incubated at 37°C for 24 hours before DNA isolation to let the bacteria grow. All samples were incubated at 56°C for 1 hour with lysis buffer and 20 $ng/\mu l$ Proteinase K, followed by DNA extraction according to the STAR BEADS Pathogen DNA/RNA Extraction kit (CYANAGEN SRL, Bologna, Italy) instructions. DNA concentrations were measured with the Qubit® 4.0 Fluorometer (Thermo Fisher Scientific) using the double-stranded DNA (dsDNA) High-Sensitivity (HS) assay kit (Thermo Fisher Scientific), following the manufacturer's protocol. The quality was evaluated with a Nanodrop® 1000 (Thermo Fisher Scientific), assessing the 260/280 nm and 260/230 nm ratios. 260/280 and 260/230 ratios were close to the expected ranges 1.8–2.0 and 2.0–2.2, respectively. Extracted DNA was barcoded before sequencing using the Native barcoding genomic DNA (with EXP-NBD104, EXP-NBD114, and SQK-LSK109) protocol (Oxford Nanopore). DNA was then loaded on an R9.4.1 MinION Mk flow cell (Oxford Nanopore). SpotON sample port cover and priming port were closed and sequencing was started. The sequencing device control, data acquisition, and real-time basecalling were carried out by the MinKNOW software the MinION Mk1C device. For 6 samples, adaptive sampling to exclude chicken DNA was used. Generated sequencing data is available via BioProject PRJNA982679. Metadata for the 46 samples is summarised in Supplementary Table T1 into five pieces of information: (i) expected strain(s), (ii) incubation before DNA isolation, (iii) adaptive sampling during sequencing, (iv) Colony-forming unit (CFU)/mL [63], a measure providing a quantitative assessment of viable microbial entities within a given sample and measured using standard microbiological techniques such as serial dilution and plating on agar medium, provides, (iv) Cycle Threshold (Ct) values [64], a measure inversely proportional to the the amount of nucleic acid in the samples.

Preprocessing

The number of reads after quality control varies significantly between samples (Figure 2 A), which impacts downstream analyses.

For host detection using Minimap2 (v 2.26), the option *PacBio/Oxford Nanopore read to reference mapping* was set here. As expected from the samples sequencing protocol (chicken samples and not isolated pathogen), most sequences were assigned to chicken: above 90% in 31 samples and between 55% to 85% for the remaining 15 samples (Supplementary Figure S1). However, the percentage of identified host DNA (between 60% and 98%) is not as low as expected for the 6 samples that have undergone adaptive sampling to exclude chicken DNA during sequencing. This shows that the adaptive sampling to exclude chicken in some samples during sequencing may not have removed all the chicken sequences. All sequences identified as chicken were removed (Figure 2 A). After QC and host removal, 19 samples had less than 1,000 reads. These samples could only be analysed using the taxonomy profiling as highlighted in the next sections.

Taxonomy Profiling

Salmonella was detected in Workflow 2 for all samples, at least at the genus taxonomic rank (Supplementary Figure S2, interactive KRONA plot in Supplementary online Figure S3).

Gene-based Pathogen Identification

In Workflow 3, Metaflye (v 2.9.1) tool mode's option is chosen to be *Nanopore-HQ*, users can expand the workflow and change this option according to their datasets sequencing technique.

No contig was built for 10 of the 27 samples with less than 2,700 reads. The identification of Virulence Factors (VF) or Anti Microbial Resistance (AMR) genes is then made impossible. For the other 17 samples, only 1 or 2 contigs were created not enough for identifying VF and AMR genes.

For the remaining 19 samples with created contigs (from 3 to 157) and number of reads higher than 2,700, VF genes were identified in 15 samples (Figure 2 B), 12 of which were incubated before

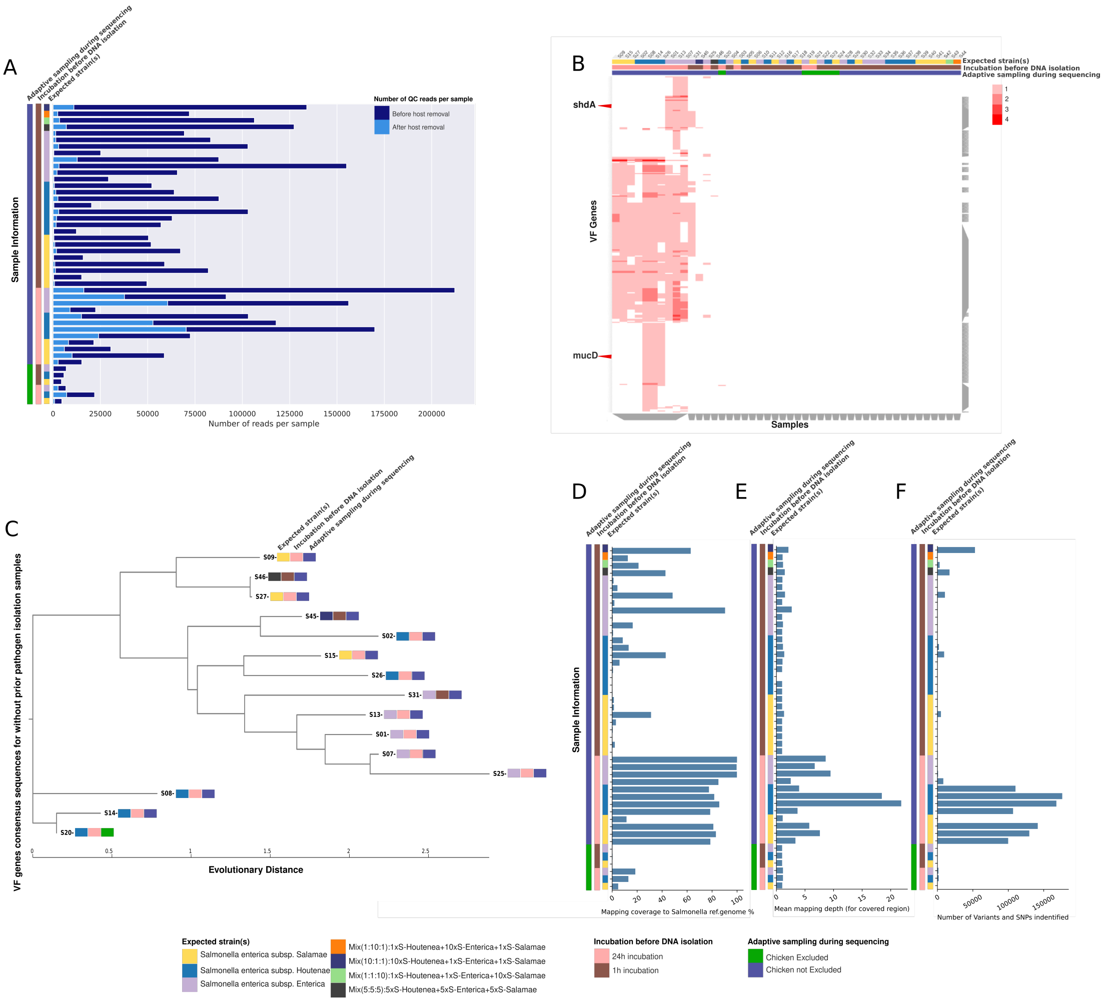

Figure 2. (**A**) Bar plot showing the total number of quality-controlled reads per sample before (dark blue) and after (light blue) host sequences removal. On the left, the metadata of the samples are displayed: (i) the expected *Salmonella* strain (*Salamae* in yellow, *Houtenae* in blue, and *Enterica* in light purple), (ii) incubation before DNA isolation (incubated for 24h in pink and incubated for 1h in brown), and (iii) adaptive sampling during sequencing (chicken excluded in green and chicken not excluded in purple)(**B**) Cluster-map displaying the identified VF genes' abundances per sample. The VF genes are presented on the y-axis and all 46 non-isolated samples are on the x-axis along with their sample information. On the top are the metadata of the samples with the same color code as in **A**. (**C**) Phylogenetic tree, using the nucleotide evolution model; General Time Reversible (GTR) model with a CAT approximation for rate heterogeneity across sites [50]. The Phylogenetic tree is built on the VF genes consensus sequences concatenated per sample and aligned for all samples. (**D**) Bar plot with the mapping coverage (breadth of coverage), i.e. the percentage of covered bases of each sample to the reference genome, measured by calculating the percentage of positions within each bin with at least one base aligned against it. (**E**) Bar plot with the mean of the mapping depth (depth of coverage) of bases mapped to corresponding bases in the reference genome for every sample. (**F**) Bar plot with the number of variants and SNPs found per sample. Mapping coverage percentage and the depth mean indicate whether to trust the variants and SNPs found by the workflow or not, the higher the coverage percentage and the depth mean, the more trusted the SNPs results for the sample.

DNA isolation for 24 hours. 3 of the 15 samples were incubated for only 1 hour before DNA isolation, resulting in a few VF genes (Figure 2 B) identified, compared to the other 12 samples, mostly because of the low number of reads (Figure 3 E) from almost the absence of incubation (Figure 2 A). It is for example the case for the mixed samples, i.e. samples spiked with all 3 *Salmonella* strains, or samples spiked with *Houtenae* and adaptively sampled during sequencing.

Some identified VF genes were found more than once in the same sample, with a maximum of 4 times. Common VF genes were identified for samples expecting identical *Salmonella* strains (Figure 2 B), such as the mucD gene, a serine protease MucD precursor, which is only found in *Houtenae* strains, or shdA, an AIDA autotransporter-like protein, only found in *Enterica* strain samples but not in samples spiked with *Houtenae* or *Salamae* strains.

Similar results were found for AMR genes (Supplementary Figure S4, 3 F). The sampling conditions affected the number of identified VF and AMR genes as shown by the relationships between the Ct value, CFU/mL value, or the number of remaining reads after preprocessing (Figure 3). The lower the Ct value, the higher the number of VF genes and AMR genes identified (Figure 3 A & B). No VF or AMR genes were detected for samples with Ct values above 26. For Ct values below 26, there is a negative correlation (Pearson $R = -0.85$, p-value $= 6 \times 10^{-05}$) between the Ct value and the number of identified AMR genes. Similar but inverse relations are observed for CFU/mL value (Figure 3 C & D), with a threshold for VF and AMR gene detection at 10^6. VF and AMR genes are then detected if several conditions are fulfilled: a Ct value below 26, CFU/mL value above 10^6, and at least 5,000 reads after preprocessing. The further the samples are from these thresholds, the higher the number of VF genes and AMR genes identified. Indeed, the three top scattered dots with identified VF genes between 250 and 300 (Figure 3 A, C, E) are the samples with the highest number of reads, higher CFU/mL value, and a relatively lower Ct value compared to other samples. Generally, allowing samples to incubate for a short period before sequencing enhances microbial growth, resulting in higher CFU/mL values and lower Ct values. This increase in microbial concentration improves the efficiency of direct sequencing by providing more genetic material for analysis, facilitating faster and more accurate pathogen detection.

Allele-based Pathogen Identification

In Workflow 4, samples were mapped against a reference genome of an expected pathogen chosen by the user. *Salmonella enterica subspecies enterica serovar typhimarium* (NC_003197.2) is chosen for this data, as it is widely recognised and extensively used in genomic studies due to its complete and well-annotated genome sequence [65]. However, given the diversity among *Salmonella* subspecies in the samples, a high number of complex variants and SNPs were anticipated.

The provided mapping statistics (mapping coverage (breadth of coverage) and mapping depth (depth of coverage) in Figure 2 D, E) serve as proxies for assessing the number and quality of identified SNPs (Figure 2 F). SNPs with low mapping depth are less reliable than those with higher depth. Reliable SNP calling typically requires a depth of at least 10, achieved in 2 samples. Samples with the highest mean mapping depth correspond to samples with the highest number of reads after preprocessing (Figure 2 A). The higher the coverage and the mean mapping depth, the more quality SNPs have been identified (Figure 2 D-F). Some of the samples spiked with *Salmonella* subsp. Enterica has a high breadth of coverage but a low mean depth of coverage depth, as a result, the number of their quality filtered identified SNPs is low.

PathoGFAIR Samples Aggregation and Visualisation

For the samples for which VF or AMR genes have been identified, phylogenetic trees are built on the concatenated genes consensus sequences (Figure 2 C for VF genes, Supplementary Figure S5 for

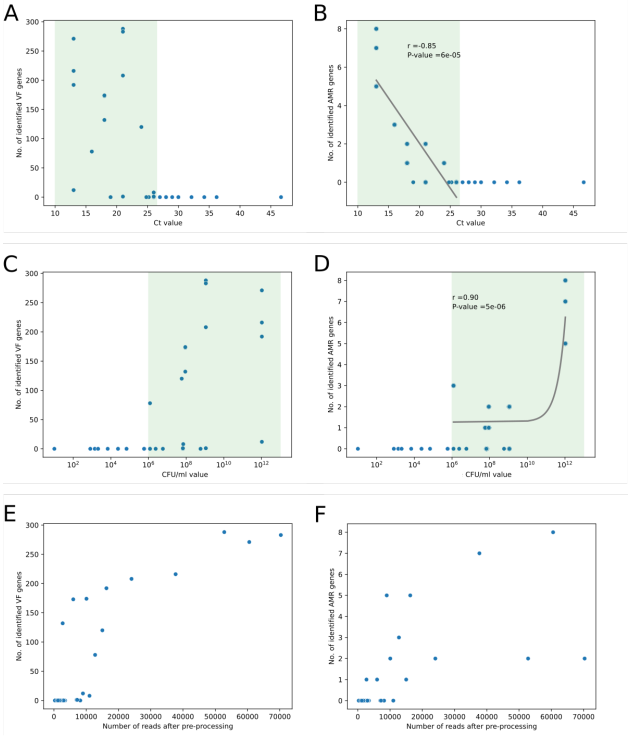

Figure 3. Scatterplots of the number of identified VF (**A, C, E**) and AMR (**B, D, F**) genes in relationship to proxies for sampling conditions: the Ct value (**A, B**), CFU/mL value (**C, D**), and the number of reads after preprocessing (**E, F**). (**A, B, C, D**) The green area highlights Ct values or CFU/mL values for which genes have been detected. Pearson correlation for values in the pistachio light green area: (**A**)$R = -0.33$, p-value $= 0.23$, (**B**) $R = -0.85$, p-value $= 5.83e^{-05}$, (**C**) $R = 0.17$, p-value $= 0.53$, (**D**) $R = 0.90$, p-value $= 4.68e^{-06}$

AMR genes). These trees help track divergence between samples and could then highlight the contamination point or an evolution of the strains because of mutations. Indeed, samples spiked with *Enterica* strain are found together in the VFs-based tree (Figure 2 C), so the identified VF genes are unique to these samples and can clearly separate the samples from samples with other strains. The samples spiked with *Houtenae* strain are mostly clustered together, except 2 samples because of extra identified VF genes common with samples spiked with *Enterica* and/or *Salamae* strains. The 2 samples spiked with a mix of the 3 stains are found in the middle of the tree (Figure 2 C), showing that a mix of VF genes related to the different strains was identified. The mixed sample, S45, spiked with a higher concentration of *Houtenae* strain than the other strains, is close to the sample, S02, spiked with *Houtenae* strain only. For AMR genes phylogenetic tree (Supplementary Figure S5), samples are not as clearly separated as the tree for VF genes, mostly because the number of identified AMR genes is relatively low compared to the number of identified VF genes.

Samples With Prior Pathogen Isolation

Data Description

To benchmark PathoGFair on additional data, 84 samples (Supplementary Table T2), sampled in Palestine were provided by the Swiss Tropical and Public Health Institute (Supplementary Figure S6) [66]. These samples were sampled either from chicken meat, chicken stool, or human stool, in 2021 or 2022. In these samples, *Salmonella Enterica* has been isolated in 19 samples, and *Campylobacter Jejuni* in 65 samples. The wet lab procedures performed to isolate and prepare these samples for sequencing adhered to standard microbiological techniques, including cultivation, enrichment, and isolation steps [66]. The generated sequencing data

were provided under BioProjects PRJNA942086 (S. enterica [67]) and PRJNA942088 (C. jejuni [68]).

Preprocessing

Negligible contamination or host sequences were found between 0% and 0.02% (Supplementary online Figure S7), as expected because of the prior isolation of the pathogen. The number of reads ranges between 3k and 217k reads per sample, after quality control.

Taxonomy Profiling

As presented in the interactive KRONA plot (Supplementary online Figure S7), the first 19 samples, *Salmonella Enterica* isolates, are assigned correctly to *Salmonella Enterica*, and the remaining 65 samples are assigned correctly to *Campylobacter Jejuni*. With the KRONA plot (Supplementary online Figure S7), the total number of reads for each sample can be seen along with detailed percentages on the assigned taxa at each taxonomic rank.

Gene-based Pathogen Identification

In this workflow, we identified VF and AMR genes for all samples, thanks to the higher number of reads retained after preprocessing and the prior isolation of the pathogens. Consequently, VF genes were detected in all samples, with more VF genes identified than AMR genes (Supplementary Figure S8), as in general, the number of VF genes in a bacterial genome is often higher than the number of AMR genes. Samples containing *Salmonella* exhibited more VF genes (172 to 207) compared to samples with *Campylobacter* (96 to 113). The opposite trend was observed for AMR genes, *Campylobacter* samples typically had 12 AMR genes detected, while *Salmonella* samples mostly had 6 AMR genes (Supplementary Figure S8)

The analysis revealed that samples with similarly isolated pathogens clustered together based on detected VF genes (4). For example, samples with *Salmonella* and *Campylobacter* formed distinct clusters. Moreover, correlations were observed among samples from different hosts, sampling years, and pathogenic species.

Specific VF genes were found in samples with similar isolated pathogens, indicating potential strain-specific differences. For instance, Cj1419c, a methyltransferase Capsule biosynthesis and transport gene product, was exclusively found in samples with *Campylobacter* sequenced in 2022, while flgB gene, encoding flagellar basal body rod protein, was only detected in *Campylobacter* sequenced in 2021. flaA (flagellin), a VF gene product identifying *Campylobacter* jejuni, was present in samples with *Salmonella* from human stool sampled in 2022 and all samples with *Campylobacter*, but not in samples with *Salmonella* from chicken meat, chicken stool, or human stool sampled in 2021.

Furthermore, certain VF genes such as spvC (type III secretion system effector SpvC phosphothreonine lyase) and pefB (plasmid-encoded fimbriae regulatory protein), associated with *Salmonella* subsp. enterica serovar Typhimurium str. LT2, were exclusively found in *Salmonella* from human stool sampled in 2022. Conversely, fyuA, a pesticin/yersiniabactin receptor protein that identifies *Yersiniabactin* Yersinia pestis, was detected in every *Salmonella* sample except those from human stool sampled in 2022. Finally, some VF genes, like flif, a flagellar M-ring protein known in *Yersinia* enterocolitica subsp. enterocolitica, were found in all samples, irrespective of the pathogen species.

Allele-based Pathogen Identification

The 19 *Salmonella Enterica* samples were mapped against the reference genome of the expected pathogen, *Salmonella enterica subspecies enterica serovar typhimurium* (NC_003197.2 [65]), and the 65 *Campylobacter Jejuni* samples were mapped against *Campylobacter Jejuni* (NC_002163.1).

The 19 *Salmonella Enterica* samples have an average mapping coverage of 94.6% and an average mean mapping depth of 31 per base. The average total number of variants found per *Salmonella Enterica* sample is 43,420. For the 65 *Campylobacter Jejuni* samples the average mapping coverage is 93.7%, the average mean mapping depth is 42 per base and the average total number of variants found per sample is 26,654. These high values for the average total number of variants identified for samples are expected since the used subspecies for the mapping are different than the subspecies of the samples.

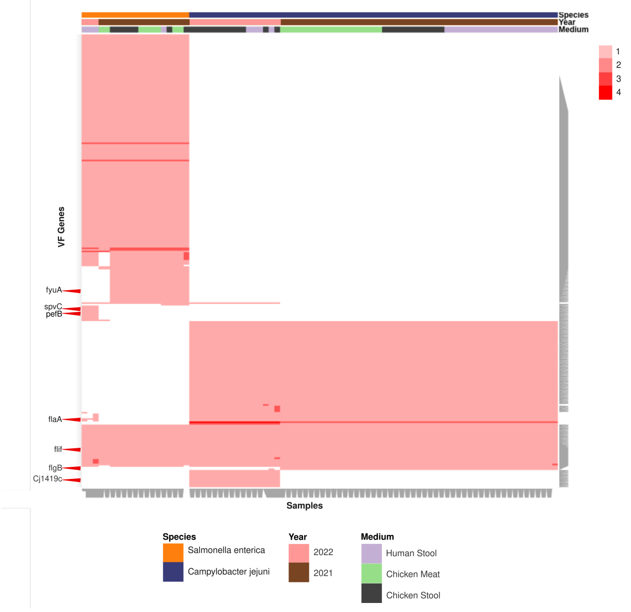

Figure 4. Cluster-map showing the identified VF genes on the y-axis for tested samples presented on the x-axis, clustered based on sample information such as sampling year, isolated pathogen species, and the original host of the sample. Clustering was performed using hierarchical clustering implemented in the Clustergrammer Python package

PathoGFAIR Samples Aggregation and Visualisation

The isolated samples exhibited a higher count of identified AMR genes compared to the metagenomic samples without prior isolation, enabling the incorporation of additional genes into concatenated gene consensus sequences. The resulting phylogenetic tree, constructed based on the AMR genes (Supplementary Figure S9), distinctly delineated different *Salmonella* strains. Similarly, this differentiation was evident in the phylogenetic tree based on the VF genes.

Conclusion

In conclusion, we present PathoGFAIR, a collection of Galaxy FAIR adaptable workflows, designed for pathogens detection and tracking. These five workflows span the entire analysis pipeline, ranging from preprocessing reads to advanced analyses including taxonomy profiling, virulence and antimicrobial resistance gene identification, SNP detection, and evolutionary history comparisons. The workflows generate diverse visualisations for a comprehensive understanding of the results, accompanied by interactive reports detailing all relevant inputs and outputs.

Our workflows have successfully identified pathogens down to genus, species, or subspecies taxonomic ranks across diverse samples, surpassing limitations observed in comparable pipelines. Our workflows facilitate comprehensive sample comparisons across diverse types, conditions, and sequencing techniques by offering interpretative and publication-ready visualisations. The open-access and user-friendly design of PathoGFAIR mitigates accessibility challenges and reduces reliance on local computational resources by leveraging Galaxy's infrastructure for computational tasks, a feature that sets it apart from similar pipelines. This scalable workflow

is a versatile solution for processing (meta)genomic samples, extending its utility beyond detecting foodborne pathogens.

In our findings, optimising sampling, preparation, and sequencing conditions, such as a 24-hour sample incubation, significantly enhances the identification of virulence and antimicrobial resistance genes. Indeed, the workflows' performance correlates with sample characteristics, with higher CFU/mL values and read counts, and lower Ct values yielding more comprehensive results, which can be used to establish sampling guidelines. Moreover, as the Pre-processing workflow effectively removes host sequences, adaptive sampling during sequencing to exclude host DNA is not necessary. The workflows are still able to detect pathogens at least at genus taxonomic rank for samples without prior pathogen isolation.

We further supported the scientific community by introducing new 46 benchmark samples, making them publicly available. This demonstrates our significant investment of time and resources, providing valuable assets for future research.

In addition to the allele-based pathogen identification method, our workflow can be further enhanced by incorporating MLST. MLST, or Multi-Locus Sequence Typing, offers an alternative approach by characterizing isolates through the sequences of house-keeping genes [45]. This method provides valuable information about the genetic diversity and evolutionary relationships among isolates, allowing for more precise identification and tracking of pathogens. By integrating MLST using MLST (v 2.22.0) tool [69]into our workflow, users can benefit from a comprehensive analysis that combines both alleles and variants identification methods, providing a more robust and accurate pathogen detection and tracking solution.

In the future, integrating PathoGFAIR with Galaxy's automated bot system holds the promise of ongoing updates and analyses requiring minimal human involvement. By establishing a dedicated bot for PathoGFAIR, continuous results will be effortlessly refreshed whenever new datasets are uploaded, similar to the Galaxy bot created for SARS-CoV-2 [70]. The Galaxy bot for SARS-CoV-2 automatically updates and reanalyses data with each new upload, maintaining up-to-date results and reducing the need for manual intervention. This automation ensures real-time, efficient data processing and analysis, enhancing the workflow's accuracy and timeliness. Leveraging the user-friendly interface of the Galaxy platform ensures accessibility for users of all computational skill levels, streamlining the entire process from sample upload to result interpretation with ease. This study not only presents a robust computational solution but also lays the groundwork for semi-automated, efficient, and user-friendly pathogen detection and tracking workflows.

Additional Files

Supplementary Figure S1. Violin plot with the percentage of quality-controlled host reads detected and removed in samples with respect to adaptive sampling during sequencing (Host excluded or not) - Samples Without Prior Pathogen Isolation
Supplementary Figure S2. Cluster-map for the taxonomy profiling at the subspecies taxonomic rank, created using hierarchical clustering to group similar taxa based on their abundance profiles - Samples Without Prior Pathogen Isolation
Supplementary online Figure S3. Krone Pie Chart for the taxonomy profiling - Samples Without Prior Pathogen Isolation
Supplementary Figure S4. Bar chart for the total number of VF genes (orange) and AMR genes (blue) found in samples with respect to incubation duration before DNA isolation - Samples Without Prior Pathogen Isolation
Supplementary Figure S5. Phylogenetic tree, using the nucleotide evolution model; General Time Reversible (GTR) model with a CAT approximation for rate heterogeneity across sites [50], for the identified AMR genes - Samples Without Prior Pathogen Isolation
Supplementary Figure S6. Upset plot illustrating the intersections of different metadata categories, including sampling year, pathogen species, and the original host of the samples, highlighting common and unique attributes among the datasets - Samples With Prior Pathogen Isolation
Supplementary online Figure S7. Krona Pie Chart for the taxonomy profiling - Samples With Prior Pathogen Isolation
Supplementary Figure S8. Violin plot for the total number of VFs and AMR genes - Samples With Prior Pathogen Isolation
Supplementary Figure S9. Phylogenetic tree, using the nucleotide evolution model; General Time Reversible (GTR) model with a CAT approximation for rate heterogeneity across sites [50], for the identified AMR genes - Samples With Prior Pathogen Isolation
Supplementary Table T1. Metadata for Samples Without Prior Pathogen Isolation
Supplementary Table T2. Metadata for Samples With Prior Pathogen Isolation

Availability of Source Code and Requirements

Lists the following:

- Project name: PathoGFAIR https://usegalaxy-eu.github.io/PathoGFAIR/
- Workflows on public Galaxy servers: https://training.galaxyproject.org/training-material/workflows/embed.html?query=pathogfair
- Workflows (v 0.1) on WorkflowHub: https://workflowhub.eu/search?utf8=%E2%9C%93&q=pathogfair
- Workflows (v 0.1) on Dockstore: https://dockstore.org/search?organization=iwc-workflows&entryType=workflows&search=engy
- Tutorial: https://training.galaxyproject.org/training-material/topics/microbiome/tutorials/pathogen-detection-from-nanopore-foodborne-data/tutorial.html
- Data analysis home page: https://github.com/usegalaxy-eu/PathoGFAIR
- Operating system(s): Platform independent
- Other requirements: Account on a Galaxy server
- License: MIT license

Data Availability

The raw sequence reads of the 46 samples without prior isolation are available on Sequence Read Archive (SRA) under BioProjects [71]. The protocol for the preparation of these samples is available on Protocol.io [62]. The workflows presented in the Methods section are available on Intergalactic Workflow Commission (IWC) and two workflow registries (Dockstore and WorkflowHub). The training material to understand, learn, and try the workflows is available on the Galaxy Training Network (GTN) [59]. The Jupyter notebook for additional visualisations and generating the figures of this paper is available in a GitHub repository [72].

Declarations

List of Abbreviations

AMR: Antimicrobial resistance; API: Application Programming Interface; CFU: Colony-forming unit; Ct: Cycle Threshold; EFSA: European Food Safety Authority; EU: European Union; FAIR: Findable Accessible Interoperable Resulable; GTN: Galaxy Training Network; IWC: Intergalactic Workflow Commission; MLST: Multilocus sequence typing; NGS: Next-Generation Sequencing; QC: Quality Con-

trol; RKI: Robert Koch Institute; SNP: Single-nucleotide polymorphism; SRA: Sequence Read Archive; VF: Virulence Factor; VFDB: Virulence Factor database; WHO: World Health Organization; WGS: Whole Genome Sequencing.

Funding

This research was supported by the Digital Life Science Call for Academia-Industry Collaborations under EOSC-Life funding [73]. Additionally, financial support was provided by the German Federal Ministry of Education and Research BMBF grant 031 A538A de.NBI-RBC and the Ministry of Science, Research and the Arts Baden-Württemberg (MWK) within the framework of LIBIS/de.NBI Freiburg. This work was supported by the Programme d'Investissements d'Avenir (PIA), grant Agence Nationale de la Recherche, number ANR-11-INBS-0013.

Author's Contributions

Engy Nasr (E.N.) led the formal analysis, investigation, software development, validation, visualisation, and writing of both the original draft and the review and editing phases. E.N. also designed the workflows, tested them, created training materials, documented and published datasets, workflows, codes, protocols, and wrote the manuscript. Anna Henger (A.H.) supported conceptualisation, formal analysis, funding acquisition, investigation, project administration, validation, visualisation, and writing. A.H. also prepared and sequenced all project datasets, ensuring alignment of analysis results with lab preparation conditions. Björn Grüning (B.G.) supported conceptualisation, formal analysis, funding acquisition, investigation, project administration, and software development. B.G. integrated new databases into analytical tools and maintained Galaxy tools used in PathoGFAIR. Paul Zierep (P.Z.) contributed to formal analysis, investigation, software development, supervision, validation, visualisation, and writing. P.Z. supervised the project and edited parts of the workflows and training materials. Bérénice Batut (B.B.) led the conceptualisation, funding acquisition, methodology, project administration, software development, and supervision. B.B. applied for the EOSC Life Industry Call Grant, designed the project's main goals and guidelines, and managed and edited the full project. All authors read and approved the final manuscript.

Acknowledgements

This research was made possible by the invaluable support of the entire Freiburg Galaxy team, Bioinformatics, University of Freiburg. The authors extend their special thanks to Wolfgang Maier and Mina Ansari for their technical expertise and academic guidance. We also appreciate the contributions of all the researchers who provided input to the project, with particular gratitude to Tobias Schindler for his exceptional assistance and to Peter van Heusden for his insightful contributions.

References

1. Elbehiry A, Abalkhail A, Marzouk E, Elmanssury AE, Almuzaini AM, Alfheeaid H, et al. An Overview of the Public Health Challenges in Diagnosing and Controlling Human Foodborne Pathogens. Vaccines 2023 Mar;11(4):725. https://www.mdpi.com/2076-393X/11/4/725.
2. Wei X, Zhao X. Advances in typing and identification of foodborne pathogens. Current Opinion in Food Science 2021 Feb;37:52–57. https://linkinghub.elsevier.com/retrieve/pii/S2214799320300692, doi:10.1016/j.cofs.2020.09.002.
3. Organization WH. WHO global strategy for food safety 2022–2030: towards stronger food safety systems and global cooperation: executive summary. World Health Organization; 2022. https://www.who.int/publications/i/item/9789240057685, accessed 24 June 2024.
4. Priyanka B, Patil RK, Dwarakanath S. A review on detection methods used for foodborne pathogens. Indian Journal of Medical Research 2016;144(3):327. http://www.ijmr.org.in/text.asp?2016/144/3/327/198677.
5. Yang S, Johnson MA, Hansen MA, Bush E, Li S, Vinatzer BA. Metagenomic sequencing for detection and identification of the boxwood blight pathogen Calonectria pseudonaviculata. Scientific Reports 2022 Jan;12(1):1399. https://www.nature.com/articles/s41598-022-05381-x.
6. Thomas T, Gilbert J, Meyer F. Metagenomics - a guide from sampling to data analysis. Microbial Informatics and Experimentation 2012 Dec;2(1):3. https://microbialinformaticsj.biomedcentral.com/articles/10.1186/2042-5783-2-3.
7. Bogaerts B, Van den Bossche A, Verhaegen B, Delbrassinne L, Mattheus W, Nouws S, et al. Closing the gap: Oxford Nanopore Technologies R10 sequencing allows comparable results to Illumina sequencing for SNP-based outbreak investigation of bacterial pathogens. Journal of Clinical Microbiology 2024 Mar;0(0):e01576–23. https://journals.asm.org/doi/10.1128/jcm.01576-23, doi:10.1128/jcm.01576-23.
8. Joensen KG, Scheutz F, Lund O, Hasman H, Kaas RS, Nielsen EM, et al. Real-Time Whole-Genome Sequencing for Routine Typing, Surveillance, and Outbreak Detection of Verotoxigenic Escherichia coli. Journal of Clinical Microbiology 2014 May;52(5):1501–1510. https://journals.asm.org/doi/10.1128/JCM.03617-13, doi:10.1128/JCM.03617-13.
9. Allard MW, Bell R, Ferreira CM, Gonzalez-Escalona N, Hoffmann M, Muruvanda T, et al. Genomics of foodborne pathogens for microbial food safety. Current Opinion in Biotechnology 2018 Feb;49:224–229. https://linkinghub.elsevier.com/retrieve/pii/S0958166917301398, doi:10.1016/j.copbio.2017.11.002.
10. Naccache SN, Federman S, Veeraraghavan N, Zaharia M, Lee D, Samayoa E, et al. A cloud-compatible bioinformatics pipeline for ultrarapid pathogen identification from next-generation sequencing of clinical samples. Genome Research 2014 Jul;24(7):1180–1192. http://genome.cshlp.org/lookup/doi/10.1101/gr.171934.113, doi:10.1101/gr.171934.113.
11. One Codex | A fast, easy-to-use platform for microbiome sequencing and analysis;, accessed 24 June 2024.
12. Clarke EL, Taylor LJ, Zhao C, Connell A, Lee JJ, Fett B, et al. Sunbeam: an extensible pipeline for analyzing metagenomic sequencing experiments. Microbiome 2019 Dec;7(1):46. https://microbiomejournal.biomedcentral.com/articles/10.1186/s40168-019-0658-x, doi:10.1186/s40168-019-0658-x.
13. Kalantar KL, Carvalho T, de Bourcy CFA, Dimitrov B, Dingle G, Egger R, et al. IDseq—An open source cloud-based pipeline and analysis service for metagenomic pathogen detection and monitoring. GigaScience 2020 Oct;9(10):giaa111. https://academic.oup.com/gigascience/article/doi/10.1093/gigascience/giaa111/5918865, doi:10.1093/gigascience/giaa111.
14. Chan Zuckerberg ID - Detect & Track Infectious Diseases;, accessed 24 June 2024.
15. The Galaxy Community, Afgan E, Nekrutenko A, Grüning BA, Blankenberg D, Goecks J, et al. The Galaxy platform for accessible, reproducible and collaborative biomedical analyses: 2022 update. Nucleic Acids Research 2022 Jul;50(W1):W345–W351. https://academic.oup.com/nar/article/50/W1/W345/6572001, doi:10.1093/nar/gkac247.
16. Yuen D, Cabansay L, Duncan A, Luu G, Hogue G, Overbeck C, et al. The Dockstore: enhancing a community platform for

sharing reproducible and accessible computational protocols. Nucleic Acids Research 2021 Jul;49(W1):W624–W632. https://doi.org/10.1093/nar/gkab346.
17. Implementing FAIR Digital Objects in the EOSC-Life Workflow Collaboratory;, https://zenodo.org/records/4605654, doi:10.5281/zenodo.4605654. Accessed 25 June 2024.
18. Hiltemann S, Rasche H, Gladman S, Hotz HR, Larivière D, Blankenberg D, et al. Galaxy Training: A powerful framework for teaching! PLOS Computational Biology 2023 Jan;19(1):e1010752. https://dx.plos.org/10.1371/journal.pcbi.1010752, doi:10.1371/journal.pcbi.1010752.
19. Llarena A, Ribeiro-Gonçalves BF, Nuno Silva D, Halkilahti J, Machado MP, Da Silva MS, et al. INNUENDO: A cross-sectoral platform for the integration of genomics in the surveillance of food-borne pathogens. EFSA Supporting Publications 2018 Nov;15(11). https://data.europa.eu/doi/10.2903/sp.efsa.2018.EN-1498, doi:10.2903/sp.efsa.2018.EN-1498.
20. Andrusch A, Dabrowski PW, Klenner J, Tausch SH, Kohl C, Osman AA, et al. PAIPline: pathogen identification in metagenomic and clinical next generation sequencing samples. Bioinformatics 2018 Sep;34(17):i715–i721. https://academic.oup.com/bioinformatics/article/34/17/i715/5093217, doi:10.1093/bioinformatics/bty595.
21. Sayers S, Li L, Ong E, Deng S, Fu G, Lin Y, et al. Victors: a web-based knowledge base of virulence factors in human and animal pathogens. Nucleic Acids Research 2019 Jan;47(D1):D693–D700. https://dx.doi.org/10.1093/nar/gky999, doi:10.1093/nar/gky999.
22. Chen S, Zhou Y, Chen Y, Gu J. fastp: an ultra-fast all-in-one FASTQ preprocessor. Bioinformatics 2018 Sep;34(17):i884–i890. https://academic.oup.com/bioinformatics/article/34/17/i884/5093234, doi:10.1093/bioinformatics/bty560.
23. Porechop — de.NBI Nanopore Training Course latest documentation;, https://denbi-nanopore-training-course.readthedocs.io/en/latest/read_qc/Porechop_1.html, accessed 24 June 2024.
24. Li H. Minimap2: pairwise alignment for nucleotide sequences. Bioinformatics 2018 Sep;34(18):3094–3100. https://academic.oup.com/bioinformatics/article/34/18/3094/4994778, doi:10.1093/bioinformatics/bty191.
25. Chaisson MJ, Tesler G. Mapping single molecule sequencing reads using basic local alignment with successive refinement (BLASR): application and theory. BMC Bioinformatics 2012 Dec;13(1):238. https://bmcbioinformatics.biomedcentral.com/articles/10.1186/1471-2105-13-238, doi:10.1186/1471-2105-13-238.
26. Li H, Durbin R. Fast and accurate short read alignment with Burrows–Wheeler transform. Bioinformatics 2009 Jul;25(14):1754–1760. https://academic.oup.com/bioinformatics/article/25/14/1754/225615, doi:10.1093/bioinformatics/btp324.
27. Sedlazeck FJ, Rescheneder P, Smolka M, Fang H, Nattestad M, Von Haeseler A, et al. Accurate detection of complex structural variations using single-molecule sequencing. Nature Methods 2018 Jun;15(6):461–468. https://www.nature.com/articles/s41592-018-0001-7, doi:10.1038/s41592-018-0001-7.
28. Wu TD, Watanabe CK. GMAP: a genomic mapping and alignment program for mRNA and EST sequences. Bioinformatics 2005 May;21(9):1859–1875. https://academic.oup.com/bioinformatics/article-lookup/doi/10.1093/bioinformatics/bti310, doi:10.1093/bioinformatics/bti310.
29. Langmead B, Salzberg SL. Fast gapped-read alignment with Bowtie 2. Nature Methods 2012 Apr;9(4):357–359. https://www.nature.com/articles/nmeth.1923, doi:10.1038/nmeth.1923.
30. Lu J, Rincon N, Wood DE, Breitwieser FP, Pockrandt C, Langmead B, et al. Metagenome analysis using the Kraken software suite. Nature Protocols 2022 Dec;17(12):2815–2839. https://www.nature.com/articles/s41596-022-00738-y, doi:10.1038/s41596-022-00738-y.
31. Katz LS, Griswold T, Lindsey R, Lauer A, Im MS, Williams G, et al. Kraken with Kalamari: Contamination Detection; 2021, accessed 24 June 2024.
32. Ewels P, Magnusson M, Lundin S, Käller M. MultiQC: summarize analysis results for multiple tools and samples in a single report. Bioinformatics 2016 Oct;32(19):3047–3048. https://academic.oup.com/bioinformatics/article/32/19/3047/2196507, doi:10.1093/bioinformatics/btw354.
33. Nanoplot — RCAC Biocontainers v1.0 documentation;, https://biocontainer-doc.readthedocs.io/en/latest/source/nanoplot/nanoplot.html, accessed 24 June 2024.
34. Portik DM, Brown CT, Pierce-Ward NT. Evaluation of taxonomic classification and profiling methods for long-read shotgun metagenomic sequencing datasets. Bioinformatics; 2022.
35. Leidenfrost RM, Pöther DC, Jäckel U, Wünschiers R. Benchmarking the MinION: Evaluating long reads for microbial profiling. Scientific Reports 2020;10(1):5125. https://www.nature.com/articles/s41598-020-61989-x, doi:10.1038/s41598-020-61989-x.
36. Govender KN, Eyre DW. Benchmarking taxonomic classifiers with Illumina and Nanopore sequence data for clinical metagenomic diagnostic applications. Microbial Genomics 2022 Oct;8(10). https://www.microbiologyresearch.org/content/journal/mgen/10.1099/mgen.0.000886, doi:10.1099/mgen.0.000886.
37. Ondov BD, Bergman NH, Phillippy AM. Interactive metagenomic visualization in a Web browser. BMC Bioinformatics 2011 Dec;12(1):385. https://bmcbioinformatics.biomedcentral.com/articles/10.1186/1471-2105-12-385, doi:10.1186/1471-2105-12-385.
38. Bik HM, Pitch Interactive. Phinch: An interactive, exploratory data visualization framework for –Omic datasets. Genomics; 2014.
39. Breitwieser FP, Salzberg SL. Pavian: Interactive analysis of metagenomics data for microbiomics and pathogen identification; 2016, https://www.biorxiv.org/content/10.1101/084715v1, doi:10.1101/084715.
40. Lin Y, Yuan J, Kolmogorov M, Shen MW, Chaisson M, Pevzner PA. Assembly of long error-prone reads using de Bruijn graphs. Proceedings of the National Academy of Sciences 2016 Dec;113(52). https://pnas.org/doi/full/10.1073/pnas.1604560113, doi:10.1073/pnas.1604560113.
41. Oxford Nanopore Technologies. Medaka;, https://github.com/nanoporetech/medaka, accessed 24 June 2024.
42. Seemann T. ABRicate;, https://github.com/tseemann/abricate, accessed 24 June 2024. Version: 2014-07-17.
43. Chen L, Zheng D, Liu B, Yang J, Jin Q. VFDB 2016: hierarchical and refined dataset for big data analysis—10 years on. Nucleic Acids Research 2016 Jan;44(D1):D694–D697. https://academic.oup.com/nar/article-lookup/doi/10.1093/nar/gkv1239, doi:10.1093/nar/gkv1239.
44. Feldgarden M, Brover V, Haft DH, Prasad AB, Slotta DJ, Tolstoy I, et al. Validating the AMRFinder Tool and Resistance Gene Database by Using Antimicrobial Resistance Genotype-Phenotype Correlations in a Collection of Isolates. Antimicrobial Agents and Chemotherapy 2019 Nov;63(11):e00483–19. https://journals.asm.org/doi/10.1128/AAC.00483-19, doi:10.1128/AAC.00483-19.
45. Pearce ME, Alikhan NF, Dallman TJ, Zhou Z, Grant K, Maiden MCJ. Comparative analysis of core genome MLST and SNP typing within a European Salmonella serovar Enteritidis outbreak. International Journal of Food Microbiology 2018;274:1–11. https://linkinghub.elsevier.com/retrieve/pii/S0168160518300746, doi:10.1016/j.ijfoodmicro.2018.02.023.
46. Hong Kong University - Biomedical Algorithms Lab (BAL). Clair3 - Symphonizing pileup and full-alignment for high-performance long-read variant calling;, original Release Date:

2021-03-30. Accessed 25 June 2024.
47. Danecek P, Bonfield JK, Liddle J, Marshall J, Ohan V, Pollard MO, et al. Twelve years of SAMtools and BCFtools. GigaScience 2021 Jan;10(2):giab008. https://academic.oup.com/gigascience/article/doi/10.1093/gigascience/giab008/6137722.
48. Cingolani P, Platts A, Wang LL, Coon M, Nguyen T, Wang L, et al. A program for annotating and predicting the effects of single nucleotide polymorphisms, SnpEff: SNPs in the genome of Drosophila melanogaster strain w ^{1118} ; iso-2; iso-3. Fly 2012 Apr;6(2):80–92. http://www.tandfonline.com/doi/abs/10.4161/fly.19695.
49. Danecek P, McCarthy SA. BCFtools/csq: Haplotype-aware variant consequences;, https://www.biorxiv.org/content/10.1101/090811v2, accessed 24 June 2024.
50. Price MN, Dehal PS, Arkin AP. FastTree 2 – Approximately Maximum-Likelihood Trees for Large Alignments. PLoS ONE 2010 Mar;5(3):e9490. https://dx.plos.org/10.1371/journal.pone.0009490, doi:10.1371/journal.pone.0009490.
51. jupyter/jupyter: Jupyter metapackage for installation, docs and chat;, https://github.com/jupyter/jupyter/tree/master, accessed 24 June 2024.
52. Hafeez A, Sial A. Comparative Analysis of Data Visualization Libraries Matplotlib and Seaborn in Python [HEC Y Cat]. International Journal of Advanced Trends in Computer Science and Engineering 2021 Feb;10:2770–281. Doi:10.30534/ijatcse/2021/391012021.
53. Martin M. Cutadapt removes adapter sequences from high-throughput sequencing reads. EMBnetjournal 2011 May;17(1):10. http://journal.embnet.org/index.php/embnetjournal/article/view/200, doi:10.14806/ej.17.1.200.
54. Bray S, Chilton J, Bernt M, Soranzo N, van den Beek M, Batut B, et al. The Planemo toolkit for developing, deploying, and executing scientific data analyses in Galaxy and beyond. Genome Research 2023;33(2):261–268. https://genome.cshlp.org/content/33/2/261, doi:10.1101/gr.276963.122.
55. Wilkinson MD, Dumontier M, Aalbersberg IJ, Appleton G, Axton M, Baak A, et al. The FAIR Guiding Principles for scientific data management and stewardship. Scientific Data 2016 Mar;3(1):160018. https://www.nature.com/articles/sdata201618.
56. Chue Hong NP, Katz DS, Barker M, Lamprecht AL, Martinez C, Psomopoulos FE, et al. FAIR Principles for Research Software (FAIR4RS Principles); 2021, https://rd-alliance.org/group/fair-research-software-fair4rs-wg/outcomes/fair-principles-research-software-fair4rs, doi:10.15497/RDA00065. Accessed 24 June 2024.
57. Visser Cd, Johansson LF, Kulkarni P, Mei H, Neerincx P, Velde KJvd, et al. Ten quick tips for building FAIR workflows. PLOS Computational Biology 2023 Sep;19(9):e1011369. https://journals.plos.org/ploscompbiol/article?id=10.1371/journal.pcbi.1011369.
58. Project G. Galaxy Workflows maintained by the Intergalactic Workflow Commission; 2024, accessed 24 June 2024. Original date: June 30, 2018.
59. Pathogen detection from (direct Nanopore) sequencing data using Galaxy - Foodborne Edition;, https://training.galaxyproject.org/training-material/topics/metagenomics/tutorials/pathogen-detection-from-nanopore-foodborne-data/tutorial.html, accessed 24 June 2024.
60. Soiland-Reyes S, Sefton P, Crosas M, Castro LJ, Coppens F, Fernández JM, et al. Packaging research artefacts with RO-Crate. Data Science 2022 Jan;5(2):97–138. https://content.iospress.com/articles/data-science/ds210053.
61. Sefton P, Ó Carragáin E, Soiland-Reyes S, Corcho O, Garijo D, Palma R, et al. RO-Crate Metadata Specification 1.1.3; 2023, https://zenodo.org/records/7867028, doi:10.5281/zenodo.7867028. Accessed 25 June 2024.
62. Nasr E, Henger A, Grüning B, Zierep P, Batut B. Samples Preparation for Foodborne Pathogen Detection and Tracking project; 2023, https://www.protocols.io/view/samples-preparation-for-foodborne-pathogen-detecti-cwhdxb26, doi:dx.doi.org/10.17504/protocols.io.8epv5x1jdg1b/v1. Accessed 01 July 2024.
63. CFU Full Form;, https://unacademy.com/content/neet-ug/full-forms/cfu/, accessed 24 June 2024.
64. Bioscientia. What do the terms dual target PCR and Ct value mean? | Laboratory Diagnostics; 2020, https://www.bioscientia.de/en/home/our-news/2020/07/what-do-the-terms-dual-target-pcr-and-ct-value-mean/, accessed 24 June 2024.
65. McClelland M, Sanderson KE, Spieth J, Clifton SW, Latreille P, Courtney L, et al. Complete genome sequence of Salmonella enterica serovar Typhimurium LT2. Nature 2001;413(6858):852–856. https://www.nature.com/articles/35101614, doi:10.1038/35101614.
66. Abukhattab S, Hosch S, Abu-Rmeileh NME, Hasan S, Vonaesch P, Crump L, et al. Whole-genome sequencing for One Health surveillance of antimicrobial resistance in conflict zones: a case study of Salmonella spp. and Campylobacter spp. in the West Bank, Palestine. Applied and Environmental Microbiology 2023 Sep;89(9):e00658–23. https://journals.asm.org/doi/10.1128/aem.00658-23, doi:10.1128/aem.00658-23.
67. Salmonella enterica subsp. enterica (ID 942086) - BioProject - NCBI;, https://www.ncbi.nlm.nih.gov/bioproject/942086, accessed 24 June 2024.
68. Campylobacter jejuni subsp. jejuni (ID 942088) - BioProject - NCBI;, https://www.ncbi.nlm.nih.gov/bioproject/942088, accessed 24 June 2024.
69. Seemann T. mlst;, original Release Date: 2014-05-03. Accessed 24 June 2024.
70. Maier W, Bray S, van den Beek M, Bouvier D, Coraor N, Miladi M, et al. Ready-to-use public infrastructure for global SARS-CoV-2 monitoring. Nature Biotechnology 2021 Oct;39(10):1178–1179. https://www.nature.com/articles/s41587-021-01069-1, doi:10.1038/s41587-021-01069-1.
71. PRJNA982679 - SRA - NCBI;, https://www.ncbi.nlm.nih.gov/sra/PRJNA982679, accessed 24 June 2024.
72. usegalaxy-eu/PathoGFAIR: PathoGFAIR: Galaxy FAIR Workflows for Pathogen Detection and Samples Comparison;, https://github.com/usegalaxy-eu/PathoGFAIR, accessed 25 June 2024.
73. Digital Life Sciences Internal Call for Academia-Industry Collaborations;, https://www.eosc-life.eu/industrycall/, accessed 24 June 2024.

Manuscript

Click here to access/download;Manuscript;bwbvymqccxfnztcrvsffjbtrknsfbpd

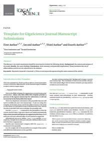

GigaScience, 2023, 1–12

doi: xx.xxxx/xxxx
Manuscript in Preparation
Technical Note

TECHNICAL NOTE

PathoGFAIR: a collection of FAIR and adaptable (meta)genomics workflows for (foodborne) pathogens detection and tracking

Engy Nasr[1], Anna Henger[2], Björn Grüning[1], Paul Zierep[1] and Bérénice Batut[3,4,*]

[1]Bioinformatics Group, Department of Computer Science, University of Freiburg, Georges-Koehler-Allee 106, 79110 Freiburg im Breisgau, Germany and [2]Biolytix AG, 4243 Dittingen, Switzerland and [3]CNRS, Institut Français de Bioinformatique, IFB-Core, UAR 3601, 91000 Evry, France and [4]Plateforme AuBi, Mésocentre Clermont-Auvergne, Université Clermont Auvergne, 63170 Aubière, France

[*]berenice.batut@gmail.com

Abstract

Background: Food contamination by pathogens poses a global health threat, affecting an estimated 600 million people annually. During a foodborne outbreak investigation, microbiological analysis of food vehicles detects responsible pathogens and traces contamination sources. Metagenomic approaches offer a comprehensive view of the genomic composition of microbial communities, facilitating the detection of potential pathogens in samples. Combined with sequencing techniques like Oxford Nanopore sequencing, such metagenomic approaches become faster and easier to apply. A key limitation of these approaches is the lack of accessible, easy-to-use, and openly available pipelines for pathogen identification and tracking from (meta)genomic data.
Findings: PathoGFAIR is a collection of Galaxy-based FAIR workflows employing state-of-the-art tools to detect and track pathogens from metagenomic Nanopore sequencing. Although initially developed for foodborne pathogen data, the workflows can be applied to any metagenomic Nanopore pathogenic data. PathoGFAIR incorporates visualisations and reports for comprehensive results. We tested PathoGFAIR on 130 benchmark samples containing different pathogens from multiple hosts under various experimental conditions. Workflows have successfully detected and tracked expected pathogens at least at the species rank in both pathogen-isolated and non-pathogen-isolated samples with sufficient Colony-forming unit and Cycle Threshold values.
Conclusions: PathoGFAIR detects the pathogens or the subspecies of the pathogens in any sample, regardless of whether the sample is isolated or incubated before sequencing. Importantly, PathoGFAIR is easy to use and can be straightforwardly adapted and extended for other types of analysis and sequencing techniques, making it usable in various pathogen detection scenarios.

Key words: Galaxy; Public health; Nanopore; Pipeline; Open source; Benchmark samples; Visualisation

Introduction

Foodborne pathogens pose a significant threat to public health worldwide, causing millions of cases of illness and even death every year [1, 2]. These diverse microorganisms, spanning bacteria, viruses, parasites, and fungi, can contaminate a variety of foods, leading to outbreaks and epidemics. The impact of foodborne pathogens on public health is a critical concern, and efforts to mitigate their spread and ensure food safety are of great importance. Food safety and controlling foodborne pathogens have been key priorities for global public health authorities [3].

Traditional methods for identifying the source of food contamination require isolation of the target pathogen. This process is not only time-consuming but can be complex and lacks a guaranteed success rate [4]. In contrast, shotgun metagenomic approaches provide a solution to these challenges, as they give an overview of the genomic composition in the sample, including the food source itself, the microbial community, and any possible pathogens and their

Compiled on: July 5, 2024.
Draft manuscript prepared by the author.

complete genetic information [5]. Importantly, shotgun metagenomic approaches eliminate the need for prior isolation of the targeted pathogen, as required by Whole Genome Sequencing (WGS) methods, and they are not limited to specific genes as opposed to real-time PCR approaches [6].

The utilisation of Nanopore sequencing data, as exemplified in studies like [7], enhances the capabilities of metagenomic approaches for outbreak investigations. Nanopore sequencing provides real-time, long-read data that can capture comprehensive genetic information, allowing for more accurate and rapid pathogen detection. This advancement becomes particularly crucial in outbreak scenarios where timely responses are essential.

Once (meta)genomics data has been generated, bioinformatics approaches enable the rapid and accurate detection, as well as the identification of genetic variations and potential Virulence Factor (VF) genes of pathogens [8, 9]. However, using already available tools and workflows requires bioinformatic and computational knowledge and expertise. Tool arguments and parameters need to be adapted to the specific use case. End-to-end workflows and platforms (1) that allow users to analyse their samples are either restricted, require high computational resources, or paid subscription (e.g. SURPI [10], OneCodex [11], Sunbeam [12]). For some of the free resources, the underlying workflow is not available and adaptable for the user. For example, IDseq [13] (also known as CZID [14]), a free cloud-based service for pathogen detection can only be externally accessed through the dedicated online user interface. Furthermore, some of these workflows are specific to a certain host, pathogen, or sequencing technique, lacking the flexibility for customisation.

Galaxy [15] is an open-source platform for FAIR data analysis. It enables users to apply a comprehensive suite of bioinformatics tools (that can be combined into workflows) through either its user-friendly web interface or its automatable Application Programming Interface (API) for integrating and customising workflows, enhancing user flexibility. It ensures reproducibility by capturing the necessary information to repeat and understand data analyses. Galaxy offers a collection of high-quality pre-built workflows that can be either used directly or are easily adapted to the user's needs via the Galaxy workflow editor. Galaxy workflows can be executed on any Galaxy server, even on the private Galaxy server, making it suitable also for data where privacy concerns are important. Furthermore, Galaxy via the major public servers [15] freely provides a large computing infrastructure allowing for the execution of computationally challenging workflows, which is often the case for metagenomic analysis.

Here, we present PathoGFAIR, a collection of Galaxy-based workflows for pathogen identification and tracking from (meta)genomics Oxford Nanopore sequencing data. The workflows are openly available on two workflow registries; Dockstore [16] and WorkflowHub [17]. They can be used directly on three major Galaxy servers (usegalaxy.org, usegalaxy.eu, usegalaxy.org.au) or installed in any other Galaxy server. The workflows are created to work agnostically, detecting all pathogens present in the samples without prior knowledge of the target pathogen. As the workflows are created in Galaxy, they can be adapted, e.g. for other sequencing techniques or with various downstream analyses, such as differential expression analysis, or further statistics and visualisations [15]. Workflows are documented and supported by an extensive tutorial freely available via the Galaxy Training Network (GTN) [18]. Overall, PathoGFAIR offers an easy-to-use computational solution that speeds up the process of sampling, detecting, and tracking pathogens. Links to workflows and tutorials can be found on PathoGFAIR homepage: `https://usegalaxy-eu.github.io/PathoGFAIR/`

Implementation

Overview

PathoGFAIR comprises a collection of 5 workflows, implemented in Galaxy (Figure 1). Each workflow serves a specific function and can be executed independently, enabling users to tailor their analysis according to their requirements.

The input data for PathoGFAIR comprises sequencing data generated using Oxford Nanopore technologies, along with a metadata table describing the datasets. The datasets undergo preprocessing in Workflow 1, which includes quality control and host removal procedures.

Subsequently, the preprocessed data is directed to three parallel workflows: taxonomy profiling (Workflow 2), gene-based pathogen identification (Workflow 3), and allele-based pathogen identification (Workflow 4). This parallel execution allows for efficient analysis and flexibility in workflow selection. Notably, Workflow 4 can optionally synchronise with Workflow 2 or Workflow 3 to leverage prior taxonomic analysis or gene-based pathogen identification results, providing users with flexibility based on specific use cases. By using detailed taxonomic identification from Workflow 2 or gene-based pathogen identification from Workflow 3, Workflow 4 enhances mapping and SNP detection accuracy and efficiency. This process involves selecting the correct reference genome of the pathogen for mapping, informed by results from Workflow 2, Workflow 3, or even Workflow 1, which performs initial taxonomy assignment during the host filtering step.

Since each workflow can be executed independently, users can focus on specific aspects of pathogen detection or analysis. This modular approach empowers users to utilise the full range of functions offered by each workflow individually or to combine them as needed for comprehensive pathogen detection.

Finally, in Workflow 5, outputs from the previous workflows and the metadata of the dataset are aggregated and visualised for comprehensive pathogen tracking across samples. This aggregation step ensures a holistic view of pathogen presence and distribution, facilitating further insights and analysis.

Overall, the independent nature of PathoGFAIR's workflows provides users with a user-friendly and customisable approach to pathogen detection, allowing for both comprehensive analyses and targeted investigations based on specific research needs or objectives.

PathoGFAIR offers a competitive, and accessible solution (Table 1) to detect and track pathogens in metagenomic Nanopore data through its five Galaxy-based FAIR and customisable workflows.

Workflow 1: Preprocessing

Workflow 1 encompasses essential preprocessing steps to ensure the quality and integrity of sequencing data.

Quality control and sequence filtering, based on quality, length, or low complexity, are performed using Fastp (v 0.23.2) [22]. Porechop (v 0.2.4) [23] trims low-quality base pairs and removes duplicates and adapters.

Quality-controlled (QC) reads are cleaned of sequences from the food vehicle animal or infected host by mapping to their reference genome using Minimap2 (v 2.26) (RRID:SCR_018550) [24], a tool tens of times faster than mainstream long-read mappers such as BLASR [25], BWA-MEM [26], NGMLR [27] and GMAP [28] and three times as fast as Bowtie2 [29] designed for Illumina short reads [24]. A variety of reference genomes (e.g. Human, Chicken, or Cow) can be installed on Galaxy servers to work with Minimap2. Kraken2 (v 1.2) [30] is applied for further contamination detection using the Kalamari database. The Kalamari database includes mitochondrial sequences of various known hosts including food hosts [31]. Reads matched to the Kalamari database are assessed and removed using Krakentools (v 1.2) [30].

The workflow returns QC reads without contamination or

Table 1. Comparison table between PathoGFAIR and other similar pipelines or systems. This comparison sheds light on various features and characteristics, such as accessibility, technical specifications, and the scope of analyses offered by each system. It serves as a reference for users to evaluate the suitability of PathoGFAIR for their specific needs and requirements

Features	PathoGFAIR	IDseq	SURPI	OneCodex	Sunbeam	Innuendo [19]	PAIPline [20]	Victors [21]
**General Characteristics**								
Free of Charge	✓	✓	✓	✗	✓	✓	✓	✓
Open Source Code	✓	✗	✓	✗	✓	✓	✓	✗
Web Interface	✓	✓	✗	✓	✗	✓	✗	✓*
Automatable API	✓	✗	✗	✗	✓	✓	✗	✗
**Accessibility and Availability**								
Simple end-user Modification	✓	✗	✗	✗	✗	✗	✗	✗
Publicly Available Web-server	✓	✓	✗	✓	✗	✗	✗	✓
Last Updated	2024	2023	2014	2023	2024	2018	2018	2019
**User Support and Documentation**								
Tutorial	✓	✗	✗	✗	✗	✗	✗	✗
Documentation	✓	✗	✗	✗	✓	✓	✗	✓
User support	✓	✗	✗	✗	✗	✗	✗	✗
**Technical Specifications**								
Workflow Manager	Galaxy	-	-	-	Snakemake	Nextflow	-	-
Sequencing Technique	Nanopore**	Illumina & Nanopore	Illumina	-	Illumina	-	Illumina	-
**Analyses**								
Preprocessing	✓	✓	✓	✓	✓	✓	✓	-
Taxonomy Profiling	✓	✓	✓	✓	✓	✓	✓	✗
Gene-based Pathogen Identification	✓	✓	✗	✗	✗	✓	✗	✓
Allele-based Pathogen Identification	✓	✗	✗	✗	✗	✗	✗	✗
Samples aggregation and Visualisations	✓	✓	✗	✓	✗	✗	✗	✗

* Malfunctioned when tested.
** Can be easily adapted to any other types of sequencing techniques via Galaxy, a customisable and automatable API.

host sequences as well as interactive reports, produced by FastQC (v 0.12.1) (RRID:SCR_014583), fastp and MultiQC (v 1.11) (RRID:SCR_014982) [32]. Furthermore, Nanoplot (v 1.39.0) [33] is employed to provide detailed quality metrics specifically tailored to the preprocessing step, enriching the suite of analytical insights and facilitating robust data evaluation.

Workflow 2: Taxonomy Profiling

The microbial communities are profiled, for QC reads from Workflow 1, using Kraken2 (v 1.2) [30] and the PlusPF (archaea, bacteria, viral, plasmid, human, UniVec_Core, protozoa, fungi, and plant) Refseq database (June 7, 2022). Although Kraken2 is a tool designed for short-read sequencing and is known for its false positive taxonomy assignments, particularly at lower microbial abundances [34], its application to long-reads can still yield a substantial overview of the microbial community. This is particularly true for discerning bacteria that could potentially be pathogenic at genus and species taxonomic ranks [35, 36]. The produced community profile is visualised using Krona (RRID:SCR_012785) [37] and observed interactively for different taxonomic ranks using Phinch [38] or Pavian [39].

Workflow 3: Gene-based Pathogen Identification

In this workflow, the pathogens are identified by the presence of genes associated with pathogenicity. QC reads from Workflow 1 are assembled into contigs using Metaflye (v 2.9.1) (RRID:SCR_017016) [40]. The contigs are then polished using the Medaka Consensus Pipeline (v 1.7.2) [41], which generates consensus sequences using neural networks and shows improved accuracy over graph-based approaches for Oxford Nanopore reads. The polished contigs are afterwards screened using ABRicate (v 1.0.1) [42] for virulence factors (VF) with the Virulence Factor DataBase (VFDB) [43] and for antimicrobial resistance (AMR) genes withAMRFinderPlus [44] database.

Workflow 4: Allele-based Pathogen Identification

Another approach to identifying pathogens is to use an allelic approach by detecting SNPs, i.e. markers showing evolutionary histories of homogeneous strains [45]. This process includes SNP calling, aimed at identifying novel pathogen strains and elucidating discrepancies compared to reference sequences, thereby facilitating the tracking of emerging variants. Within Workflow 4, both complex variants and SNPs are discerned, serving as crucial elements for subsequent pathogen identification and variant tracking purposes.

QC reads from Workflow 1 are mapped using Minimap2 (v 2.26) to a selected reference genome of a suspected pathogen. Users can choose the reference genome based on their prior knowledge of the target pathogen, the taxonomic analysis in Workflow 2, or the detected pathogenic genes in Workflow 3. Variant calling for mapped reads is performed using Clair3 (v 0.1.12) [46]. Clair3, a tool developed for long reads, has been chosen because it is demonstrated to be faster and more accurate than the Medaka variant pipeline, which its developer has declared deprecated in favour of Clair3 [41]. After that, all complex variants along with their information, such as type, genomics position, and quality score, are normalised using bcftools norm (v 1.9) [47]. The normalised reads are filtered using SnpSift filter (v 4.3) (RRID:SCR_015624) [48] based on the SNP quality computed in the SNPs identification step with Clair3. Filtered variants fields required for further analyses are extracted using SnpSift extract fields (v 4.3) (RRID:SCR_015624) [48]. Finally, a consensus sequence for each sample is built using bcftools consensus (v 1.9) (RRID:SCR_005227) [49]. In addition to the variants,

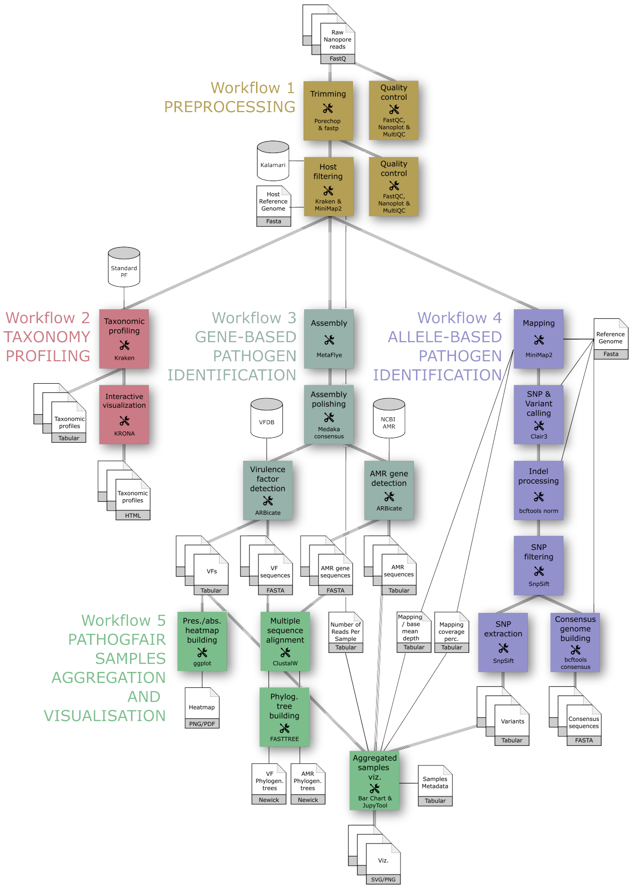

Figure 1. Flowchart of the PathoGFAIR workflows. Workflow 1 (olive green) takes as input sequencing data generated by Oxford Nanopore technologies and performs quality control and host filtering. Then three parallel workflows are executed on the output of Workflow 1: Workflow 2 (red) for taxonomy profiling, Workflow 3 (dark cyan) for gene-based pathogen identification, and Workflow 4 (purple) for SNP-based pathogen identification. These four workflows can run individually and in parallel. Finally, all outputs for the different provided datasets are aggregated in Workflow 5 (green) for PathoGFAIR Samples Aggregation and Visualisation.

this workflow outputs tables including summary metrics like the mapping coverage (breadth of coverage) percentages for every sample, per base covering mean depth (depth of covering), and quality filtered complex variants and SNPs numbers.

Workflow 5: PathoGFAIR Samples Aggregation and Visualisation

In all previously described workflows, individual samples are analysed separately. Workflow 5 consolidates the outputs from Workflows 1, 2, 3, and 4 along with sample metadata to generate various visualisations and reports. These reports illustrate the detected pathogens and facilitate the visualisation and tracking of their presence across all samples.

Virulence Factor (VF) tables from Workflow 3 are used to generate clustered heatmaps showing the VF genes using ggplot2 Heatmap (v 3.4.0) (RRID:SCR_014601). VF sequences are concatenated per sample and aligned over all samples using ClustalW (v 2.1). A phylogenetic tree of the virulence gene sequences is then generated from the multiple sequence alignment using FASTTREE (v 2.1.10) (RRID:SCR_015501) [50] and visualised using Newick Display (v 1.6). The same is performed on the antimicrobial resistance (AMR) tables from Workflow 3. From Workflows 1 and 4 output tables, bar charts are generated.

Other outputs are aggregated and processed within a Jupyter Notebook [51], interactively launched in Galaxy using JupyTool (v 1.0.0). This Notebook showcases the integration of sample metadata to generate analysis-specific plots, leveraging Python (v 3.10.12) libraries such as Pandas (v 1.5.3), Matplotlib (v 3.7.1), Seaborn (v 0.12.2), and Numpy (v 1.24.3) [52]. Examples of these plots include bar plots illustrating the number of reads before and after quality control for all samples, scatter plots visualising relationships between different variables such as pathogen count and sample characteristics, and interactive cluster maps displaying the clustering patterns of samples based on pathogen composition. These visualisation techniques are further elucidated and exemplified in the Use Cases section of this study, where the output tables from the workflows are aggregated with the corresponding sample metadata and visualised to facilitate comprehensive visual analysis.

Workflow Reports

As all PathoGFAIR workflows are designed to run seamlessly on the Galaxy platform, an interactive report is automatically generated upon completion of each workflow. These reports provide a comprehensive overview of the respective workflow's inputs and outputs. In PathoGFAIR, special attention has been given to refining these reports for enhanced user experience. The reports are carefully curated to automatically showcase and emphasise only the most informative, easily interpretable, and accessible outputs for each workflow. This ensures that users can efficiently extract key insights from the results, facilitating a streamlined and user-friendly analysis experience.

Easily Adaptable Workflows

The workflows can process raw shotgun (meta)genomics sequencing data from any sample, not only food.

PathoGFAIR has been initially developed to take Oxford Nanopore data as inputs. However, PathoGFAIR can work with Illumina data or other types of sequencing technique data. To adapt to Illumina sequencing only one tool needs to be changed in Workflow 1: Porechop [23] with Cutadapt (RRID:SCR_011841) [53]. Workflows 2, 3, 4, and 5 can be used directly with Illumina datasets without any adaptation. Some tools can be changed based on the tool's known performance towards short and long reads, such as Clair3 (v 0.1.12) [46] and Metaflye (v 2.9.1) [40]. All the mentioned tools are accessible within Galaxy, allowing for seamless interchangeability.

The workflows can also be adapted to process paired-end reads, by adjusting the tools' parameters to take paired-end read samples instead of single-end reads. These changes can be applied with little effort by using the user-friendly workflow editor in Galaxy.

Users can seamlessly switch between different host reference genomes and Kraken2 databases, as PathoGFAIR supports various pre-installed databases on the Galaxy servers. This feature enhances user convenience and efficiently explores different configurations to suit specific analysis requirements.

Similarly, tool versions and parameters can be adapted, e.g. to compare results with legacy versions of the workflows. New tool versions are automatically installed on public Galaxy servers using a sophisticated update infrastructure, ensuring a straightforward mechanism to keep the infrastructure up-to-date [54]. Every time a tool is updated, an update of the workflows is suggested, tested with functional tests, and released on the workflow registries once accepted.

Each of the five PathoGFAIR workflows is designed for a distinct type of analysis. Workflows 2, 3, and 4 operate independently, offering the flexibility to run them concurrently or skip them as per user requirements. This modular structure allows users to tailor the analysis to their specific needs, activating only the functionalities necessary for the desired workflow outcome.

FAIR Workflows

The FAIR principles [55], which emphasise the importance of making research objects Findable, Accessible, Interoperable, and Reusable, offer valuable guidance for optimising the utility and promoting the reproducibility and reusability of any research object (data, software [56], or workflows).

PathoGFAIR has been developed with the FAIR principle in mind and follows the ten tips for building FAIR workflows, as suggested by de Visser *et al.* [57]. First, by using Galaxy as a workflow manager, the workflows are portable (Tip 6) and come with a reproducible computational environment (Tip 7). The tools integrated into the workflows use file format standards such as FASTA and FASTQ for sequence data, SAM and BAM from the Samtools project for alignment data, VCF for genetic variations, GenBank and GFF3 for genomic annotations, and PDB for structural data (Tip 5) [55]. As explained in the previous section, the workflows are provided with default values (Tip 8) and are modular (Tip 9).

The workflows are available on the GitHub repository of IWC, the Intergalactic Workflow Commission of the Galaxy community (Tip 3) [58]. Workflows in this repository are reviewed and tested using test data before publication and with every new Galaxy release. The IWC automatically updates the workflows whenever a new version of any tool used in these workflows is released. Deposited workflows follow best practices, are versioned using GitHub releases, and contain important metadata (e.g. License, Author, Institutes) (Tip 2). The workflows are automatically added to two workflow repositories (Dockstore [16] and WorkflowHub [17]) to facilitate the discovery and re-use of workflows in an accessible and interoperable way (Tip 1). Via Dockstore or WorkflowHub, the PathoGFAIR workflows can be installed on any up-to-date Galaxy server. They are already publicly available on three main Galaxy servers (usegalaxy.org, usegalaxy.eu, usegalaxy.org.au), which any user can use and modify without restriction.

A thorough explanation of how to use the workflows in PathoGFAIR including a more global description of pathogen identification from Oxford Nanopore data can be found in a dedicated extensive tutorial [59] together with example input data and results (Tips 4 and 10), freely available and hosted via the Galaxy Training Network (GTN) [18] infrastructure.

Finally, for every invocation of the workflows, a Research Object Crate (RO-Crate [60, 61]) can be created to store the data products of the different steps, along with the run-associated metadata (including parameters, tool, and workflow version).

Use Cases

To demonstrate PathoGFAIR and its features, 130 samples from two studies (without or with prior pathogen isolation) were analysed. All samples contained pathogens known beforehand and were sequenced using Oxford Nanopore technology. All workflows of PathoGFAIR were evaluated for their main intended tasks, e.g., the preprocessing workflow for its reads quality retaining and hosts sequences removal performance, but also for their ability to identify the correct pathogen, and how well the accuracy with respect to different sampling conditions is.

Samples Without Prior Pathogen Isolation

Data Generation

In this study, 46 samples have been prepared given the following protocol [62]. Chicken meat was spiked with either one of three *Salmonella Enterica* subspecies (Salmonella enterica subsp. Houtenae DSM 9221, Salmonella enterica subsp. Enterica DSM 554, Salmonella enterica subsp. Salamae DSM 9220) or a mix of them, with concentrations that give Cycle Threshold (Ct) values between 25 and 33. 15 samples were incubated at 37°C for 24 hours before DNA isolation to let the bacteria grow. All samples were incubated at 56°C for 1 hour with lysis buffer and 20 $ng/\mu l$ Proteinase K, followed by DNA extraction according to the STAR BEADS Pathogen DNA/RNA Extraction kit (CYANAGEN SRL, Bologna, Italy) instructions. DNA concentrations were measured with the Qubit® 4.0 Fluorometer (Thermo Fisher Scientific) using the double-stranded DNA (dsDNA) High-Sensitivity (HS) assay kit (Thermo Fisher Scientific), following the manufacturer's protocol. The quality was evaluated with a Nanodrop® 1000 (Thermo Fisher Scientific), assessing the 260/280 nm and 260/230 nm ratios. 260/280 and 260/230 ratios were close to the expected ranges 1.8–2.0 and 2.0–2.2, respectively. Extracted DNA was barcoded before sequencing using the Native barcoding genomic DNA (with EXP-NBD104, EXP-NBD114, and SQK-LSK109) protocol (Oxford Nanopore). DNA was then loaded on an R9.4.1 MinION Mk flow cell (Oxford Nanopore). SpotON sample port cover and priming port were closed and sequencing was started. The sequencing device control, data acquisition, and real-time basecalling were carried out by the MinKNOW software the MinION Mk1C device. For 6 samples, adaptive sampling to exclude chicken DNA was used. Generated sequencing data is available via BioProject PRJNA982679. Metadata for the 46 samples is summarised in Supplementary Table T1 into five pieces of information: (i) expected strain(s), (ii) incubation before DNA isolation, (iii) adaptive sampling during sequencing, (iv) Colony-forming unit (CFU)/mL [63], a measure providing a quantitative assessment of viable microbial entities within a given sample and measured using standard microbiological techniques such as serial dilution and plating on agar medium, provides, (iv) Cycle Threshold (Ct) values [64], a measure inversely proportional to the the amount of nucleic acid in the samples.

Preprocessing

The number of reads after quality control varies significantly between samples (Figure 2 A), which impacts downstream analyses.

For host detection using Minimap2 (v 2.26), the option *PacBio/Oxford Nanopore read to reference mapping* was set here. As expected from the samples sequencing protocol (chicken samples and not isolated pathogen), most sequences were assigned to chicken: above 90% in 31 samples and between 55% to 85% for the remaining 15 samples (Supplementary Figure S1). However, the percentage of identified host DNA (between 60% and 98%) is not as low as expected for the 6 samples that have undergone adaptive sampling to exclude chicken DNA during sequencing. This shows that the adaptive sampling to exclude chicken in some samples during sequencing may not have removed all the chicken sequences. All sequences identified as chicken were removed (Figure 2 A). After QC and host removal, 19 samples had less than 1,000 reads. These samples could only be analysed using the taxonomy profiling as highlighted in the next sections.

Taxonomy Profiling

Salmonella was detected in Workflow 2 for all samples, at least at the genus taxonomic rank (Supplementary Figure S2, interactive KRONA plot in Supplementary online Figure S3).

Gene-based Pathogen Identification

In Workflow 3, Metaflye (v 2.9.1) tool mode's option is chosen to be *Nanopore-HQ*, users can expand the workflow and change this option according to their datasets sequencing technique.

No contig was built for 10 of the 27 samples with less than 2,700 reads. The identification of Virulence Factors (VF) or Anti Microbial Resistance (AMR) genes is then made impossible. For the other 17 samples, only 1 or 2 contigs were created not enough for identifying VF and AMR genes.

For the remaining 19 samples with created contigs (from 3 to 157) and number of reads higher than 2,700, VF genes were identified in 15 samples (Figure 2 B), 12 of which were incubated before

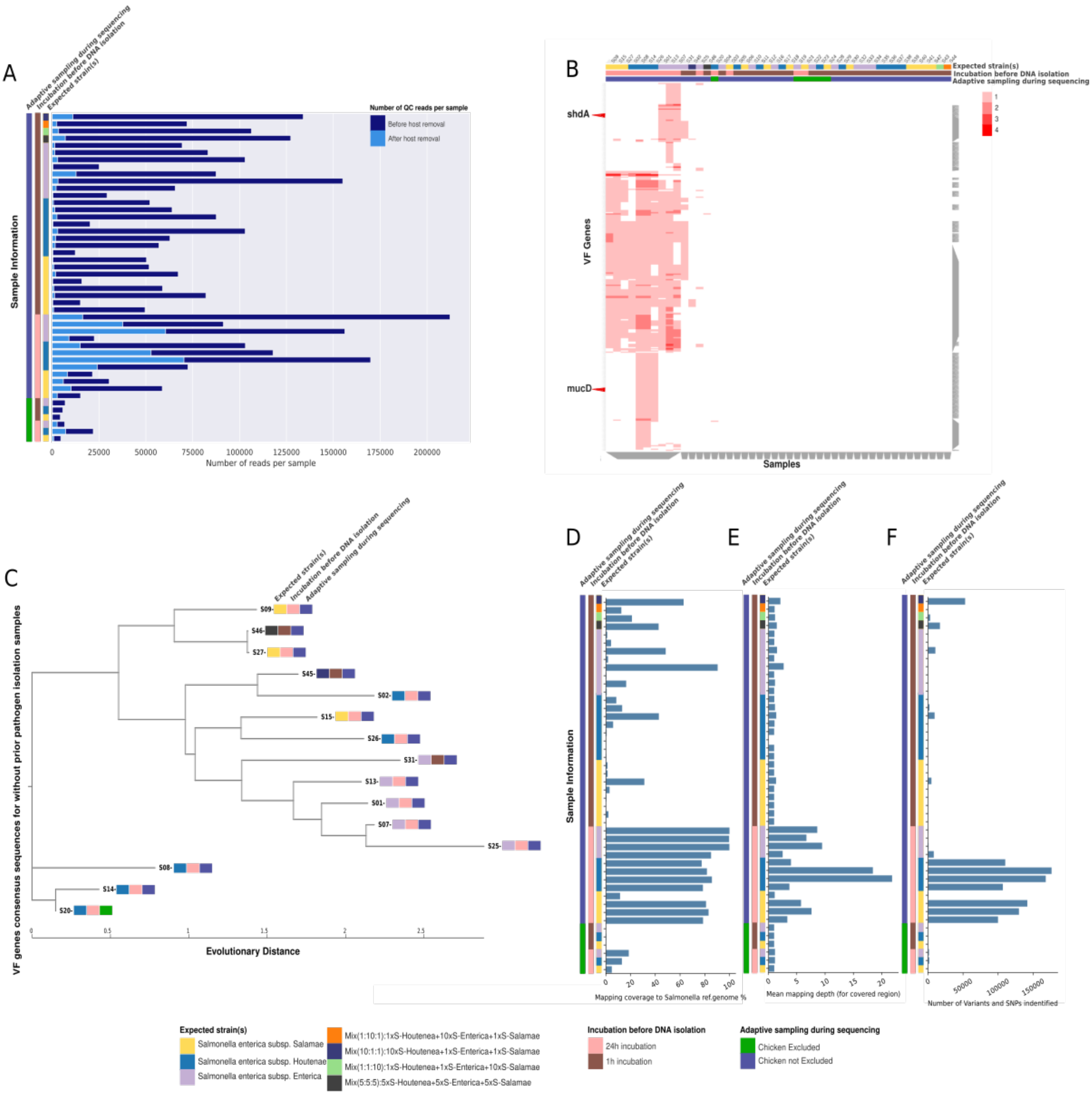

Figure 2. (**A**) Bar plot showing the total number of quality-controlled reads per sample before (dark blue) and after (light blue) host sequences removal. On the left, the metadata of the samples are displayed: (i) the expected *Salmonella* strain (*Salamae* in yellow, *Houtenae* in blue, and *Enterica* in light purple), (ii) incubation before DNA isolation (incubated for 24h in pink and incubated for 1h in brown), and (iii) adaptive sampling during sequencing (chicken excluded in green and chicken not excluded in purple)(**B**) Cluster-map displaying the identified VF genes' abundances per sample. The VF genes are presented on the y-axis and all 46 non-isolated samples are on the x-axis along with their sample information. On the top are the metadata of the samples with the same color code as in **A**. (**C**) Phylogenetic tree, using the nucleotide evolution model; General Time Reversible (GTR) model with a CAT approximation for rate heterogeneity across sites [50]. The Phylogenetic tree is built on the VF genes consensus sequences concatenated per sample and aligned for all samples. (**D**) Bar plot with the mapping coverage (breadth of coverage), i.e. the percentage of covered bases of each sample to the reference genome, measured by calculating the percentage of positions within each bin with at least one base aligned against it. (**E**) Bar plot with the mean of the mapping depth (depth of coverage) of bases mapped to corresponding bases in the reference genome for every sample. (**F**) Bar plot with the number of variants and SNPs found per sample. Mapping coverage percentage and the depth mean indicate whether to trust the variants and SNPs found by the workflow or not, the higher the coverage percentage and the depth mean, the more trusted the SNPs results for the sample.

DNA isolation for 24 hours. 3 of the 15 samples were incubated for only 1 hour before DNA isolation, resulting in a few VF genes (Figure 2 B) identified, compared to the other 12 samples, mostly because of the low number of reads (Figure 3 E) from almost the absence of incubation (Figure 2 A). It is for example the case for the mixed samples, i.e. samples spiked with all 3 *Salmonella* strains, or samples spiked with *Houtenae* and adaptively sampled during sequencing.

Some identified VF genes were found more than once in the same sample, with a maximum of 4 times. Common VF genes were identified for samples expecting identical *Salmonella* strains (Figure 2 B), such as the mucD gene, a serine protease MucD precursor, which is only found in *Houtenae* strains, or shdA, an AIDA autotransporter-like protein, only found in *Enterica* strain samples but not in samples spiked with *Houtenae* or *Salamae* strains.

Similar results were found for AMR genes (Supplementary Figure S4, 3 F). The sampling conditions affected the number of identified VF and AMR genes as shown by the relationships between the Ct value, CFU/mL value, or the number of remaining reads after preprocessing (Figure 3). The lower the Ct value, the higher the number of VF genes and AMR genes identified (Figure 3 A & B). No VF or AMR genes were detected for samples with Ct values above 26. For Ct values below 26, there is a negative correlation (Pearson $R = -0.85$, p-value $= 6 \times 10^{-05}$) between the Ct value and the number of identified AMR genes. Similar but inverse relations are observed for CFU/mL value (Figure 3 C & D), with a threshold for VF and AMR gene detection at 10^6. VF and AMR genes are then detected if several conditions are fulfilled: a Ct value below 26, CFU/mL value above 10^6, and at least 5,000 reads after preprocessing. The further the samples are from these thresholds, the higher the number of VF genes and AMR genes identified. Indeed, the three top scattered dots with identified VF genes between 250 and 300 (Figure 3 A, C, E) are the samples with the highest number of reads, higher CFU/mL value, and a relatively lower Ct value compared to other samples. Generally, allowing samples to incubate for a short period before sequencing enhances microbial growth, resulting in higher CFU/mL values and lower Ct values. This increase in microbial concentration improves the efficiency of direct sequencing by providing more genetic material for analysis, facilitating faster and more accurate pathogen detection.

Allele-based Pathogen Identification

In Workflow 4, samples were mapped against a reference genome of an expected pathogen chosen by the user. *Salmonella enterica subspecies enterica serovar typhimarium* (NC_003197.2) is chosen for this data, as it is widely recognised and extensively used in genomic studies due to its complete and well-annotated genome sequence [65]. However, given the diversity among *Salmonella* subspecies in the samples, a high number of complex variants and SNPs were anticipated.

The provided mapping statistics (mapping coverage (breadth of coverage) and mapping depth (depth of coverage) in Figure 2 D, E) serve as proxies for assessing the number and quality of identified SNPs (Figure 2 F). SNPs with low mapping depth are less reliable than those with higher depth. Reliable SNP calling typically requires a depth of at least 10, achieved in 2 samples. Samples with the highest mean mapping depth correspond to samples with the highest number of reads after preprocessing (Figure 2 A). The higher the coverage and the mean mapping depth, the more quality SNPs have been identified (Figure 2 D-F). Some of the samples spiked with *Salmonella* subsp. Enterica has a high breadth of coverage but a low mean depth of coverage depth, as a result, the number of their quality filtered identified SNPs is low.

PathoGFAIR Samples Aggregation and Visualisation

For the samples for which VF or AMR genes have been identified, phylogenetic trees are built on the concatenated genes consensus sequences (Figure 2 C for VF genes, Supplementary Figure S5 for

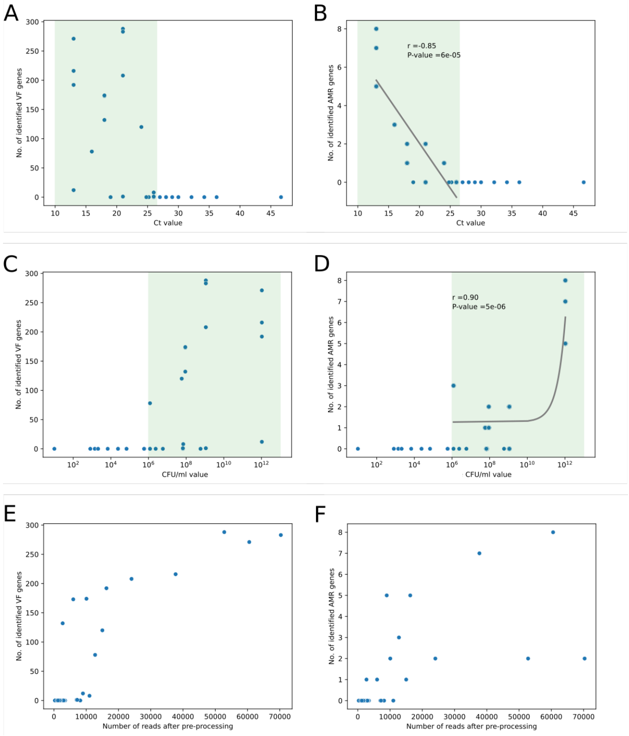

Figure 3. Scatterplots of the number of identified VF (**A, C, E**) and AMR (**B, D, F**) genes in relationship to proxies for sampling conditions: the Ct value (**A, B**), CFU/mL value (**C, D**), and the number of reads after preprocessing (**E, F**). (**A, B, C, D**) The green area highlights Ct values or CFU/mL values for which genes have been detected. Pearson correlation for values in the pistachio light green area: (**A**)$R = -0.33$, p-value $= 0.23$, (**B**) $R = -0.85$, p-value $= 5.83e^{-05}$, (**C**) $R = 0.17$, p-value $= 0.53$, (**D**) $R = 0.90$, p-value $= 4.68e^{-06}$

AMR genes). These trees help track divergence between samples and could then highlight the contamination point or an evolution of the strains because of mutations. Indeed, samples spiked with *Enterica* strain are found together in the VFs-based tree (Figure 2 C), so the identified VF genes are unique to these samples and can clearly separate the samples from samples with other strains. The samples spiked with *Houtenae* strain are mostly clustered together, except 2 samples because of extra identified VF genes common with samples spiked with *Enterica* and/or *Salamae* strains. The 2 samples spiked with a mix of the 3 stains are found in the middle of the tree (Figure 2 C), showing that a mix of VF genes related to the different strains was identified. The mixed sample, S45, spiked with a higher concentration of *Houtenae* strain than the other strains, is close to the sample, S02, spiked with *Houtenae* strain only. For AMR genes phylogenetic tree (Supplementary Figure S5), samples are not as clearly separated as the tree for VF genes, mostly because the number of identified AMR genes is relatively low compared to the number of identified VF genes.

Samples With Prior Pathogen Isolation

Data Description

To benchmark PathoGFair on additional data, 84 samples (Supplementary Table T2), sampled in Palestine were provided by the Swiss Tropical and Public Health Institute (Supplementary Figure S6) [66]. These samples were sampled either from chicken meat, chicken stool, or human stool, in 2021 or 2022. In these samples, *Salmonella Enterica* has been isolated in 19 samples, and *Campylobacter Jejuni* in 65 samples. The wet lab procedures performed to isolate and prepare these samples for sequencing adhered to standard microbiological techniques, including cultivation, enrichment, and isolation steps [66]. The generated sequencing data

were provided under BioProjects PRJNA942086 (S. enterica [67]) and PRJNA942088 (C. jejuni [68]).

Preprocessing
Negligible contamination or host sequences were found between 0% and 0.02% (Supplementary online Figure S7), as expected because of the prior isolation of the pathogen. The number of reads ranges between 3k and 217k reads per sample, after quality control.

Taxonomy Profiling
As presented in the interactive KRONA plot (Supplementary online Figure S7), the first 19 samples, *Salmonella Enterica* isolates, are assigned correctly to *Salmonella Enterica*, and the remaining 65 samples are assigned correctly to *Campylobacter Jejuni*. With the KRONA plot (Supplementary online Figure S7), the total number of reads for each sample can be seen along with detailed percentages on the assigned taxa at each taxonomic rank.

Gene-based Pathogen Identification
In this workflow, we identified VF and AMR genes for all samples, thanks to the higher number of reads retained after preprocessing and the prior isolation of the pathogens. Consequently, VF genes were detected in all samples, with more VF genes identified than AMR genes (Supplementary Figure S8), as in general, the number of VF genes in a bacterial genome is often higher than the number of AMR genes. Samples containing *Salmonella* exhibited more VF genes (172 to 207) compared to samples with *Campylobacter* (96 to 113). The opposite trend was observed for AMR genes, *Campylobacter* samples typically had 12 AMR genes detected, while *Salmonella* samples mostly had 6 AMR genes (Supplementary Figure S8)

The analysis revealed that samples with similarly isolated pathogens clustered together based on detected VF genes (4). For example, samples with *Salmonella* and *Campylobacter* formed distinct clusters. Moreover, correlations were observed among samples from different hosts, sampling years, and pathogenic species.

Specific VF genes were found in samples with similar isolated pathogens, indicating potential strain-specific differences. For instance, Cj1419c, a methyltransferase Capsule biosynthesis and transport gene product, was exclusively found in samples with *Campylobacter* sequenced in 2022, while flgB gene, encoding flagellar basal body rod protein, was only detected in *Campylobacter* sequenced in 2021. flaA (flagellin), a VF gene product identifying *Campylobacter* jejuni, was present in samples with *Salmonella* from human stool sampled in 2022 and all samples with *Campylobacter*, but not in samples with *Salmonella* from chicken meat, chicken stool, or human stool sampled in 2021.

Furthermore, certain VF genes such as spvC (type III secretion system effector SpvC phosphothreonine lyase) and pefB (plasmid-encoded fimbriae regulatory protein), associated with *Salmonella* subsp. enterica serovar Typhimurium str. LT2, were exclusively found in *Salmonella* from human stool sampled in 2022. Conversely, fyuA, a pesticin/yersiniabactin receptor protein that identifies *Yersiniabactin* Yersinia pestis, was detected in every *Salmonella* sample except those from human stool sampled in 2022. Finally, some VF genes, like flif, a flagellar M-ring protein known in *Yersinia* enterocolitica subsp. enterocolitica, were found in all samples, irrespective of the pathogen species.

Allele-based Pathogen Identification
The 19 *Salmonella Enterica* samples were mapped against the reference genome of the expected pathogen, *Salmonella enterica subspecies enterica serovar typhimurium* (NC_003197.2 [65]), and the 65 *Campylobacter Jejuni* samples were mapped against *Campylobacter Jejuni* (NC_002163.1).

The 19 *Salmonella Enterica* samples have an average mapping coverage of 94.6% and an average mean mapping depth of 31 per base. The average total number of variants found per *Salmonella Enterica* sample is 43,420. For the 65 *Campylobacter Jejuni* samples

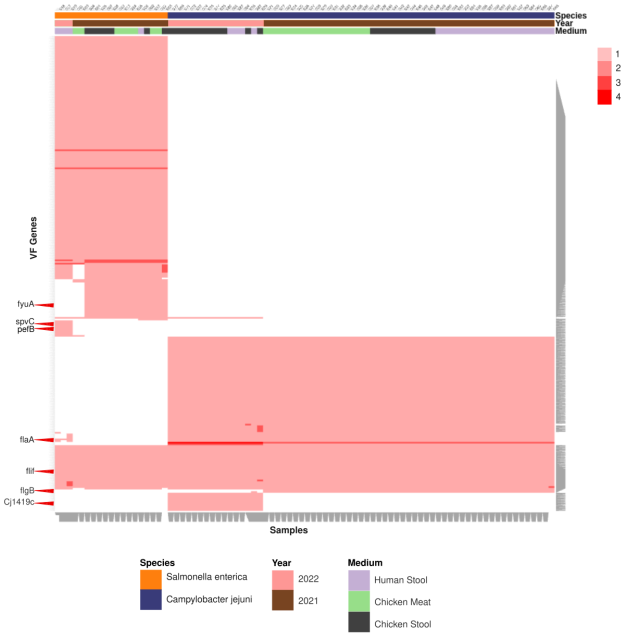

Figure 4. Cluster-map showing the identified VF genes on the y-axis for tested samples presented on the x-axis, clustered based on sample information such as sampling year, isolated pathogen species, and the original host of the sample. Clustering was performed using hierarchical clustering implemented in the Clustergrammer Python package

the average mapping coverage is 93.7%, the average mean mapping depth is 42 per base and the average total number of variants found per sample is 26,654. These high values for the average total number of variants identified for samples are expected since the used subspecies for the mapping are different than the subspecies of the samples.

PathoGFAIR Samples Aggregation and Visualisation
The isolated samples exhibited a higher count of identified AMR genes compared to the metagenomic samples without prior isolation, enabling the incorporation of additional genes into concatenated gene consensus sequences. The resulting phylogenetic tree, constructed based on the AMR genes (Supplementary Figure S9), distinctly delineated different *Salmonella* strains. Similarly, this differentiation was evident in the phylogenetic tree based on the VF genes.

Conclusion

In conclusion, we present PathoGFAIR, a collection of Galaxy FAIR adaptable workflows, designed for pathogens detection and tracking. These five workflows span the entire analysis pipeline, ranging from preprocessing reads to advanced analyses including taxonomy profiling, virulence and antimicrobial resistance gene identification, SNP detection, and evolutionary history comparisons. The workflows generate diverse visualisations for a comprehensive understanding of the results, accompanied by interactive reports detailing all relevant inputs and outputs.

Our workflows have successfully identified pathogens down to genus, species, or subspecies taxonomic ranks across diverse samples, surpassing limitations observed in comparable pipelines. Our workflows facilitate comprehensive sample comparisons across diverse types, conditions, and sequencing techniques by offering interpretative and publication-ready visualisations. The open-access and user-friendly design of PathoGFAIR mitigates accessibility challenges and reduces reliance on local computational resources by leveraging Galaxy's infrastructure for computational tasks, a fea-

ture that sets it apart from similar pipelines. This scalable workflow is a versatile solution for processing (meta)genomic samples, extending its utility beyond detecting foodborne pathogens.

In our findings, optimising sampling, preparation, and sequencing conditions, such as a 24-hour sample incubation, significantly enhances the identification of virulence and antimicrobial resistance genes. Indeed, the workflows' performance correlates with sample characteristics, with higher CFU/mL values and read counts, and lower Ct values yielding more comprehensive results, which can be used to establish sampling guidelines. Moreover, as the Pre-processing workflow effectively removes host sequences, adaptive sampling during sequencing to exclude host DNA is not necessary. The workflows are still able to detect pathogens at least at genus taxonomic rank for samples without prior pathogen isolation.

We further supported the scientific community by introducing new 46 benchmark samples, making them publicly available. This demonstrates our significant investment of time and resources, providing valuable assets for future research.

In addition to the allele-based pathogen identification method, our workflow can be further enhanced by incorporating MLST. MLST, or Multi-Locus Sequence Typing, offers an alternative approach by characterizing isolates through the sequences of housekeeping genes [45]. This method provides valuable information about the genetic diversity and evolutionary relationships among isolates, allowing for more precise identification and tracking of pathogens. By integrating MLST using MLST (v 2.22.0) tool [69]into our workflow, users can benefit from a comprehensive analysis that combines both alleles and variants identification methods, providing a more robust and accurate pathogen detection and tracking solution.

In the future, integrating PathoGFAIR with Galaxy's automated bot system holds the promise of ongoing updates and analyses requiring minimal human involvement. By establishing a dedicated bot for PathoGFAIR, continuous results will be effortlessly refreshed whenever new datasets are uploaded, similar to the Galaxy bot created for SARS-CoV-2 [70]. The Galaxy bot for SARS-CoV-2 automatically updates and reanalyses data with each new upload, maintaining up-to-date results and reducing the need for manual intervention. This automation ensures real-time, efficient data processing and analysis, enhancing the workflow's accuracy and timeliness. Leveraging the user-friendly interface of the Galaxy platform ensures accessibility for users of all computational skill levels, streamlining the entire process from sample upload to result interpretation with ease. This study not only presents a robust computational solution but also lays the groundwork for semi-automated, efficient, and user-friendly pathogen detection and tracking workflows.

Additional Files

Supplementary Figure S1. Violin plot with the percentage of quality-controlled host reads detected and removed in samples with respect to adaptive sampling during sequencing (Host excluded or not) - Samples Without Prior Pathogen Isolation
Supplementary Figure S2. Cluster-map for the taxonomy profiling at the subspecies taxonomic rank, created using hierarchical clustering to group similar taxa based on their abundance profiles - Samples Without Prior Pathogen Isolation
Supplementary online Figure S3. Krone Pie Chart for the taxonomy profiling - Samples Without Prior Pathogen Isolation
Supplementary Figure S4. Bar chart for the total number of VF genes (orange) and AMR genes (blue) found in samples with respect to incubation duration before DNA isolation - Samples Without Prior Pathogen Isolation
Supplementary Figure S5. Phylogenetic tree, using the nucleotide evolution model; General Time Reversible (GTR) model with a CAT approximation for rate heterogeneity across sites [50], for the identified AMR genes - Samples Without Prior Pathogen Isolation
Supplementary Figure S6. Upset plot illustrating the intersections of different metadata categories, including sampling year, pathogen species, and the original host of the samples, highlighting common and unique attributes among the datasets - Samples With Prior Pathogen Isolation
Supplementary online Figure S7. Krona Pie Chart for the taxonomy profiling - Samples With Prior Pathogen Isolation
Supplementary Figure S8. Violin plot for the total number of VFs and AMR genes - Samples With Prior Pathogen Isolation
Supplementary Figure S9. Phylogenetic tree, using the nucleotide evolution model; General Time Reversible (GTR) model with a CAT approximation for rate heterogeneity across sites [50], for the identified AMR genes - Samples With Prior Pathogen Isolation
Supplementary Table T1. Metadata for Samples Without Prior Pathogen Isolation
Supplementary Table T2. Metadata for Samples With Prior Pathogen Isolation

Availability of Source Code and Requirements

Lists the following:

- Project name: PathoGFAIR https://usegalaxy-eu.github.io/PathoGFAIR/
- Workflows on public Galaxy servers: https://training.galaxyproject.org/training-material/workflows/embed.html?query=pathogfair
- Workflows (v 0.1) on WorkflowHub: https://workflowhub.eu/search?utf8=%E2%9C%93&q=pathogfair
- Workflows (v 0.1) on Dockstore: https://dockstore.org/search?organization=iwc-workflows&entryType=workflows&search=engy
- Tutorial: https://training.galaxyproject.org/training-material/topics/microbiome/tutorials/pathogen-detection-from-nanopore-foodborne-data/tutorial.html
- Data analysis home page: https://github.com/usegalaxy-eu/PathoGFAIR
- Operating system(s): Platform independent
- Other requirements: Account on a Galaxy server
- License: MIT license

Data Availability

The raw sequence reads of the 46 samples without prior isolation are available on Sequence Read Archive (SRA) under BioProjects [71]. The protocol for the preparation of these samples is available on Protocol.io [62]. The workflows presented in the Methods section are available on Intergalactic Workflow Commission (IWC) and two workflow registries (Dockstore and WorkflowHub). The training material to understand, learn, and try the workflows is available on the Galaxy Training Network (GTN) [59]. The Jupyter notebook for additional visualisations and generating the figures of this paper is available in a GitHub repository [72].

Declarations

List of Abbreviations

AMR: Antimicrobial resistance; API: Application Programming Interface; CFU: Colony-forming unit; Ct: Cycle Threshold; EFSA: European Food Safety Authority; EU: European Union; FAIR: Findable Accessible Interoperable Resulable; GTN: Galaxy Training Network; IWC: Intergalactic Workflow Commission; MLST: Multilocus se-

quence typing; NGS: Next-Generation Sequencing; QC: Quality Control; RKI: Robert Koch Institute; SNP: Single-nucleotide polymorphism; SRA: Sequence Read Archive; VF: Virulence Factor; VFDB: Virulence Factor database; WHO: World Health Organization; WGS: Whole Genome Sequencing.

Funding

This research was supported by the Digital Life Science Call for Academia-Industry Collaborations under EOSC-Life funding [73]. Additionally, financial support was provided by the German Federal Ministry of Education and Research BMBF grant 031 A538A de.NBI-RBC and the Ministry of Science, Research and the Arts Baden-Württemberg (MWK) within the framework of LIBIS/de.NBI Freiburg. This work was supported by the Programme d'Investissements d'Avenir (PIA), grant Agence Nationale de la Recherche, number ANR-11-INBS-0013.

Author's Contributions

Engy Nasr (E.N.) led the formal analysis, investigation, software development, validation, visualisation, and writing of both the original draft and the review and editing phases. E.N. also designed the workflows, tested them, created training materials, documented and published datasets, workflows, codes, protocols, and wrote the manuscript. Anna Henger (A.H.) supported conceptualisation, formal analysis, funding acquisition, investigation, project administration, validation, visualisation, and writing. A.H. also prepared and sequenced all project datasets, ensuring alignment of analysis results with lab preparation conditions. Björn Grüning (B.G.) supported conceptualisation, formal analysis, funding acquisition, investigation, project administration, and software development. B.G. integrated new databases into analytical tools and maintained Galaxy tools used in PathoGFAIR. Paul Zierep (P.Z.) contributed to formal analysis, investigation, software development, supervision, validation, visualisation, and writing. P.Z. supervised the project and edited parts of the workflows and training materials. Bérénice Batut (B.B.) led the conceptualisation, funding acquisition, methodology, project administration, software development, and supervision. B.B. applied for the EOSC Life Industry Call Grant, designed the project's main goals and guidelines, and managed and edited the full project. All authors read and approved the final manuscript.

Acknowledgements

This research was made possible by the invaluable support of the entire Freiburg Galaxy team, Bioinformatics, University of Freiburg. The authors extend their special thanks to Wolfgang Maier and Mina Ansari for their technical expertise and academic guidance. We also appreciate the contributions of all the researchers who provided input to the project, with particular gratitude to Tobias Schindler for his exceptional assistance and to Peter van Heusden for his insightful contributions.

References

1. Elbehiry A, Abalkhail A, Marzouk E, Elmanssury AE, Almuzaini AM, Alfheeaid H, et al. An Overview of the Public Health Challenges in Diagnosing and Controlling Human Foodborne Pathogens. Vaccines 2023 Mar;11(4):725. https://www.mdpi.com/2076-393X/11/4/725.
2. Wei X, Zhao X. Advances in typing and identification of foodborne pathogens. Current Opinion in Food Science 2021 Feb;37:52–57. https://linkinghub.elsevier.com/retrieve/pii/S2214799320300692, doi:10.1016/j.cofs.2020.09.002.
3. Organization WH. WHO global strategy for food safety 2022–2030: towards stronger food safety systems and global cooperation: executive summary. World Health Organization; 2022. https://www.who.int/publications/i/item/9789240057685, accessed 24 June 2024.
4. Priyanka B, Patil RK, Dwarakanath S. A review on detection methods used for foodborne pathogens. Indian Journal of Medical Research 2016;144(3):327. http://www.ijmr.org.in/text.asp?2016/144/3/327/198677.
5. Yang S, Johnson MA, Hansen MA, Bush E, Li S, Vinatzer BA. Metagenomic sequencing for detection and identification of the boxwood blight pathogen Calonectria pseudonaviculata. Scientific Reports 2022 Jan;12(1):1399. https://www.nature.com/articles/s41598-022-05381-x.
6. Thomas T, Gilbert J, Meyer F. Metagenomics - a guide from sampling to data analysis. Microbial Informatics and Experimentation 2012 Dec;2(1):3. https://microbialinformaticsj.biomedcentral.com/articles/10.1186/2042-5783-2-3.
7. Bogaerts B, Van den Bossche A, Verhaegen B, Delbrassinne L, Mattheus W, Nouws S, et al. Closing the gap: Oxford Nanopore Technologies R10 sequencing allows comparable results to Illumina sequencing for SNP-based outbreak investigation of bacterial pathogens. Journal of Clinical Microbiology 2024 Mar;0(0):e01576–23. https://journals.asm.org/doi/10.1128/jcm.01576-23, doi:10.1128/jcm.01576-23.
8. Joensen KG, Scheutz F, Lund O, Hasman H, Kaas RS, Nielsen EM, et al. Real-Time Whole-Genome Sequencing for Routine Typing, Surveillance, and Outbreak Detection of Verotoxigenic Escherichia coli. Journal of Clinical Microbiology 2014 May;52(5):1501–1510. https://journals.asm.org/doi/10.1128/JCM.03617-13, doi:10.1128/JCM.03617-13.
9. Allard MW, Bell R, Ferreira CM, Gonzalez-Escalona N, Hoffmann M, Muruvanda T, et al. Genomics of foodborne pathogens for microbial food safety. Current Opinion in Biotechnology 2018 Feb;49:224–229. https://linkinghub.elsevier.com/retrieve/pii/S0958166917301398, doi:10.1016/j.copbio.2017.11.002.
10. Naccache SN, Federman S, Veeraraghavan N, Zaharia M, Lee D, Samayoa E, et al. A cloud-compatible bioinformatics pipeline for ultrarapid pathogen identification from next-generation sequencing of clinical samples. Genome Research 2014 Jul;24(7):1180–1192. http://genome.cshlp.org/lookup/doi/10.1101/gr.171934.113, doi:10.1101/gr.171934.113.
11. One Codex | A fast, easy-to-use platform for microbiome sequencing and analysis;, accessed 24 June 2024.
12. Clarke EL, Taylor LJ, Zhao C, Connell A, Lee JJ, Fett B, et al. Sunbeam: an extensible pipeline for analyzing metagenomic sequencing experiments. Microbiome 2019 Dec;7(1):46. https://microbiomejournal.biomedcentral.com/articles/10.1186/s40168-019-0658-x, doi:10.1186/s40168-019-0658-x.
13. Kalantar KL, Carvalho T, de Bourcy CFA, Dimitrov B, Dingle G, Egger R, et al. IDseq—An open source cloud-based pipeline and analysis service for metagenomic pathogen detection and monitoring. GigaScience 2020 Oct;9(10):giaa111. https://academic.oup.com/gigascience/article/doi/10.1093/gigascience/giaa111/5918865, doi:10.1093/gigascience/giaa111.
14. Chan Zuckerberg ID - Detect & Track Infectious Diseases;, accessed 24 June 2024.
15. The Galaxy Community, Afgan E, Nekrutenko A, Grüning BA, Blankenberg D, Goecks J, et al. The Galaxy platform for accessible, reproducible and collaborative biomedical analyses: 2022 update. Nucleic Acids Research 2022 Jul;50(W1):W345–W351. https://academic.oup.com/nar/article/50/W1/W345/6572001, doi:10.1093/nar/gkac247.

16. Yuen D, Cabansay L, Duncan A, Luu G, Hogue G, Overbeck C, et al. The Dockstore: enhancing a community platform for sharing reproducible and accessible computational protocols. Nucleic Acids Research 2021 Jul;49(W1):W624–W632. https://doi.org/10.1093/nar/gkab346.
17. Implementing FAIR Digital Objects in the EOSC-Life Workflow Collaboratory;, https://zenodo.org/records/4605654, doi:10.5281/zenodo.4605654. Accessed 25 June 2024.
18. Hiltemann S, Rasche H, Gladman S, Hotz HR, Larivière D, Blankenberg D, et al. Galaxy Training: A powerful framework for teaching! PLOS Computational Biology 2023 Jan;19(1):e1010752. https://dx.plos.org/10.1371/journal.pcbi.1010752, doi:10.1371/journal.pcbi.1010752.
19. Llarena A, Ribeiro-Gonçalves BF, Nuno Silva D, Halkilahti J, Machado MP, Da Silva MS, et al. INNUENDO: A cross-sectoral platform for the integration of genomics in the surveillance of food-borne pathogens. EFSA Supporting Publications 2018 Nov;15(11). https://data.europa.eu/doi/10.2903/sp.efsa.2018.EN-1498, doi:10.2903/sp.efsa.2018.EN-1498.
20. Andrusch A, Dabrowski PW, Klenner J, Tausch SH, Kohl C, Osman AA, et al. PAIPline: pathogen identification in metagenomic and clinical next generation sequencing samples. Bioinformatics 2018 Sep;34(17):i715–i721. https://academic.oup.com/bioinformatics/article/34/17/i715/5093217, doi:10.1093/bioinformatics/bty595.
21. Sayers S, Li L, Ong E, Deng S, Fu G, Lin Y, et al. Victors: a web-based knowledge base of virulence factors in human and animal pathogens. Nucleic Acids Research 2019 Jan;47(D1):D693–D700. https://dx.doi.org/10.1093/nar/gky999, doi:10.1093/nar/gky999.
22. Chen S, Zhou Y, Chen Y, Gu J. fastp: an ultra-fast all-in-one FASTQ preprocessor. Bioinformatics 2018 Sep;34(17):i884–i890. https://academic.oup.com/bioinformatics/article/34/17/i884/5093234, doi:10.1093/bioinformatics/bty560.
23. Porechop — de.NBI Nanopore Training Course latest documentation;, https://denbi-nanopore-training-course.readthedocs.io/en/latest/read_qc/Porechop_1.html, accessed 24 June 2024.
24. Li H. Minimap2: pairwise alignment for nucleotide sequences. Bioinformatics 2018 Sep;34(18):3094–3100. https://academic.oup.com/bioinformatics/article/34/18/3094/4994778, doi:10.1093/bioinformatics/bty191.
25. Chaisson MJ, Tesler G. Mapping single molecule sequencing reads using basic local alignment with successive refinement (BLASR): application and theory. BMC Bioinformatics 2012 Dec;13(1):238. https://bmcbioinformatics.biomedcentral.com/articles/10.1186/1471-2105-13-238, doi:10.1186/1471-2105-13-238.
26. Li H, Durbin R. Fast and accurate short read alignment with Burrows–Wheeler transform. Bioinformatics 2009 Jul;25(14):1754–1760. https://academic.oup.com/bioinformatics/article/25/14/1754/225615, doi:10.1093/bioinformatics/btp324.
27. Sedlazeck FJ, Rescheneder P, Smolka M, Fang H, Nattestad M, Von Haeseler A, et al. Accurate detection of complex structural variations using single-molecule sequencing. Nature Methods 2018 Jun;15(6):461–468. https://www.nature.com/articles/s41592-018-0001-7, doi:10.1038/s41592-018-0001-7.
28. Wu TD, Watanabe CK. GMAP: a genomic mapping and alignment program for mRNA and EST sequences. Bioinformatics 2005 May;21(9):1859–1875. https://academic.oup.com/bioinformatics/article-lookup/doi/10.1093/bioinformatics/bti310, doi:10.1093/bioinformatics/bti310.
29. Langmead B, Salzberg SL. Fast gapped-read alignment with Bowtie 2. Nature Methods 2012 Apr;9(4):357–359. https://www.nature.com/articles/nmeth.1923, doi:10.1038/nmeth.1923.
30. Lu J, Rincon N, Wood DE, Breitwieser FP, Pockrandt C, Langmead B, et al. Metagenome analysis using the Kraken software suite. Nature Protocols 2022 Dec;17(12):2815–2839. https://www.nature.com/articles/s41596-022-00738-y, doi:10.1038/s41596-022-00738-y.
31. Katz LS, Griswold T, Lindsey R, Lauer A, Im MS, Williams G, et al. Kraken with Kalamari: Contamination Detection; 2021, accessed 24 June 2024.
32. Ewels P, Magnusson M, Lundin S, Käller M. MultiQC: summarize analysis results for multiple tools and samples in a single report. Bioinformatics 2016 Oct;32(19):3047–3048. https://academic.oup.com/bioinformatics/article/32/19/3047/2196507, doi:10.1093/bioinformatics/btw354.
33. Nanoplot — RCAC Biocontainers v1.0 documentation;, https://biocontainer-doc.readthedocs.io/en/latest/source/nanoplot/nanoplot.html, accessed 24 June 2024.
34. Portik DM, Brown CT, Pierce-Ward NT. Evaluation of taxonomic classification and profiling methods for long-read shotgun metagenomic sequencing datasets. Bioinformatics; 2022.
35. Leidenfrost RM, Pöther DC, Jäckel U, Wünschiers R. Benchmarking the MinION: Evaluating long reads for microbial profiling. Scientific Reports 2020;10(1):5125. https://www.nature.com/articles/s41598-020-61989-x, doi:10.1038/s41598-020-61989-x.
36. Govender KN, Eyre DW. Benchmarking taxonomic classifiers with Illumina and Nanopore sequence data for clinical metagenomic diagnostic applications. Microbial Genomics 2022 Oct;8(10). https://www.microbiologyresearch.org/content/journal/mgen/10.1099/mgen.0.000886, doi:10.1099/mgen.0.000886.
37. Ondov BD, Bergman NH, Phillippy AM. Interactive metagenomic visualization in a Web browser. BMC Bioinformatics 2011 Dec;12(1):385. https://bmcbioinformatics.biomedcentral.com/articles/10.1186/1471-2105-12-385, doi:10.1186/1471-2105-12-385.
38. Bik HM, Pitch Interactive. Phinch: An interactive, exploratory data visualization framework for –Omic datasets. Genomics; 2014.
39. Breitwieser FP, Salzberg SL. Pavian: Interactive analysis of metagenomics data for microbiomics and pathogen identification; 2016, https://www.biorxiv.org/content/10.1101/084715v1, doi:10.1101/084715.
40. Lin Y, Yuan J, Kolmogorov M, Shen MW, Chaisson M, Pevzner PA. Assembly of long error-prone reads using de Bruijn graphs. Proceedings of the National Academy of Sciences 2016 Dec;113(52). https://pnas.org/doi/full/10.1073/pnas.1604560113, doi:10.1073/pnas.1604560113.
41. Oxford Nanopore Technologies. Medaka;, https://github.com/nanoporetech/medaka, accessed 24 June 2024.
42. Seemann T. ABRicate;, https://github.com/tseemann/abricate, accessed 24 June 2024. Version: 2014-07-17.
43. Chen L, Zheng D, Liu B, Yang J, Jin Q. VFDB 2016: hierarchical and refined dataset for big data analysis—10 years on. Nucleic Acids Research 2016 Jan;44(D1):D694–D697. https://academic.oup.com/nar/article-lookup/doi/10.1093/nar/gkv1239, doi:10.1093/nar/gkv1239.
44. Feldgarden M, Brover V, Haft DH, Prasad AB, Slotta DJ, Tolstoy I, et al. Validating the AMRFinder Tool and Resistance Gene Database by Using Antimicrobial Resistance Genotype-Phenotype Correlations in a Collection of Isolates. Antimicrobial Agents and Chemotherapy 2019 Nov;63(11):e00483–19. https://journals.asm.org/doi/10.1128/AAC.00483-19, doi:10.1128/AAC.00483-19.
45. Pearce ME, Alikhan NF, Dallman TJ, Zhou Z, Grant K, Maiden MCJ. Comparative analysis of core genome MLST and SNP typing within a European Salmonella serovar Enteritidis outbreak. International Journal of Food Microbiology 2018;274:1–11. https://linkinghub.elsevier.com/retrieve/pii/S0168160518300746, doi:10.1016/j.ijfoodmicro.2018.02.023.
46. Hong Kong University - Biomedical Algorithms Lab (BAL).

Clair3 - Symphonizing pileup and full-alignment for high-performance long-read variant calling;, original Release Date: 2021-03-30. Accessed 25 June 2024.
47. Danecek P, Bonfield JK, Liddle J, Marshall J, Ohan V, Pollard MO, et al. Twelve years of SAMtools and BCFtools. GigaScience 2021 Jan;10(2):giab008. https://academic.oup.com/gigascience/article/doi/10.1093/gigascience/giab008/6137722.
48. Cingolani P, Platts A, Wang LL, Coon M, Nguyen T, Wang L, et al. A program for annotating and predicting the effects of single nucleotide polymorphisms, SnpEff: SNPs in the genome of Drosophila melanogaster strain w ^{1118} ; iso-2; iso-3. Fly 2012 Apr;6(2):80–92. http://www.tandfonline.com/doi/abs/10.4161/fly.19695.
49. Danecek P, McCarthy SA. BCFtools/csq: Haplotype-aware variant consequences;, https://www.biorxiv.org/content/10.1101/090811v2, accessed 24 June 2024.
50. Price MN, Dehal PS, Arkin AP. FastTree 2 – Approximately Maximum-Likelihood Trees for Large Alignments. PLoS ONE 2010 Mar;5(3):e9490. https://dx.plos.org/10.1371/journal.pone.0009490, doi:10.1371/journal.pone.0009490.
51. jupyter/jupyter: Jupyter metapackage for installation, docs and chat;, https://github.com/jupyter/jupyter/tree/master, accessed 24 June 2024.
52. Hafeez A, Sial A. Comparative Analysis of Data Visualization Libraries Matplotlib and Seaborn in Python [HEC Y Cat]. International Journal of Advanced Trends in Computer Science and Engineering 2021 Feb;10:2770–281. Doi:10.30534/ijatcse/2021/391012021.
53. Martin M. Cutadapt removes adapter sequences from high-throughput sequencing reads. EMBnetjournal 2011 May;17(1):10. http://journal.embnet.org/index.php/embnetjournal/article/view/200, doi:10.14806/ej.17.1.200.
54. Bray S, Chilton J, Bernt M, Soranzo N, van den Beek M, Batut B, et al. The Planemo toolkit for developing, deploying, and executing scientific data analyses in Galaxy and beyond. Genome Research 2023;33(2):261–268. https://genome.cshlp.org/content/33/2/261, doi:10.1101/gr.276963.122.
55. Wilkinson MD, Dumontier M, Aalbersberg IJ, Appleton G, Axton M, Baak A, et al. The FAIR Guiding Principles for scientific data management and stewardship. Scientific Data 2016 Mar;3(1):160018. https://www.nature.com/articles/sdata201618.
56. Chue Hong NP, Katz DS, Barker M, Lamprecht AL, Martinez C, Psomopoulos FE, et al. FAIR Principles for Research Software (FAIR4RS Principles); 2021, https://rd-alliance.org/group/fair-research-software-fair4rs-wg/outcomes/fair-principles-research-software-fair4rs, doi:10.15497/RDA00065. Accessed 24 June 2024.
57. Visser Cd, Johansson LF, Kulkarni P, Mei H, Neerincx P, Velde KJvd, et al. Ten quick tips for building FAIR workflows. PLOS Computational Biology 2023 Sep;19(9):e1011369. https://journals.plos.org/ploscompbiol/article?id=10.1371/journal.pcbi.1011369.
58. Project G. Galaxy Workflows maintained by the Intergalactic Workflow Commission; 2024, accessed 24 June 2024. Original date: June 30, 2018.
59. Pathogen detection from (direct Nanopore) sequencing data using Galaxy - Foodborne Edition;, https://training.galaxyproject.org/training-material/topics/metagenomics/tutorials/pathogen-detection-from-nanopore-foodborne-data/tutorial.html, accessed 24 June 2024.
60. Soiland-Reyes S, Sefton P, Crosas M, Castro LJ, Coppens F, Fernández JM, et al. Packaging research artefacts with RO-Crate. Data Science 2022 Jan;5(2):97–138. https://content.iospress.com/articles/data-science/ds210053.
61. Sefton P, Ó Carragáin E, Soiland-Reyes S, Corcho O, Garijo D, Palma R, et al. RO-Crate Metadata Specification 1.1.3; 2023, https://zenodo.org/records/7867028, doi:10.5281/zenodo.7867028. Accessed 25 June 2024.
62. Nasr E, Henger A, Grüning B, Zierep P, Batut B. Samples Preparation for Foodborne Pathogen Detection and Tracking project; 2023, https://www.protocols.io/view/samples-preparation-for-foodborne-pathogen-detecti-cwhdxb26, doi:dx.doi.org/10.17504/protocols.io.8epv5x1jdg1b/v1. Accessed 01 July 2024.
63. CFU Full Form;, https://unacademy.com/content/neet-ug/full-forms/cfu/, accessed 24 June 2024.
64. Bioscientia. What do the terms dual target PCR and Ct value mean? | Laboratory Diagnostics; 2020, https://www.bioscientia.de/en/home/our-news/2020/07/what-do-the-terms-dual-target-pcr-and-ct-value-mean/, accessed 24 June 2024.
65. McClelland M, Sanderson KE, Spieth J, Clifton SW, Latreille P, Courtney L, et al. Complete genome sequence of Salmonella enterica serovar Typhimurium LT2. Nature 2001;413(6858):852–856. https://www.nature.com/articles/35101614, doi:10.1038/35101614.
66. Abukhattab S, Hosch S, Abu-Rmeileh NME, Hasan S, Vonaesch P, Crump L, et al. Whole-genome sequencing for One Health surveillance of antimicrobial resistance in conflict zones: a case study of Salmonella spp. and Campylobacter spp. in the West Bank, Palestine. Applied and Environmental Microbiology 2023 Sep;89(9):e00658–23. https://journals.asm.org/doi/10.1128/aem.00658-23, doi:10.1128/aem.00658-23.
67. Salmonella enterica subsp. enterica (ID 942086) - BioProject - NCBI;, https://www.ncbi.nlm.nih.gov/bioproject/942086, accessed 24 June 2024.
68. Campylobacter jejuni subsp. jejuni (ID 942088) - BioProject - NCBI;, https://www.ncbi.nlm.nih.gov/bioproject/942088, accessed 24 June 2024.
69. Seemann T. mlst;, original Release Date: 2014-05-03. Accessed 24 June 2024.
70. Maier W, Bray S, van den Beek M, Bouvier D, Coraor N, Miladi M, et al. Ready-to-use public infrastructure for global SARS-CoV-2 monitoring. Nature Biotechnology 2021 Oct;39(10):1178–1179. https://www.nature.com/articles/s41587-021-01069-1, doi:10.1038/s41587-021-01069-1.
71. PRJNA982679 - SRA - NCBI;, https://www.ncbi.nlm.nih.gov/sra/PRJNA982679, accessed 24 June 2024.
72. usegalaxy-eu/PathoGFAIR: PathoGFAIR: Galaxy FAIR Workflows for Pathogen Detection and Samples Comparison;, https://github.com/usegalaxy-eu/PathoGFAIR, accessed 25 June 2024.
73. Digital Life Sciences Internal Call for Academia-Industry Collaborations;, https://www.eosc-life.eu/industrycall/, accessed 24 June 2024.

Figure 1

Click here to access/download;Figure;Figure_1.pdf

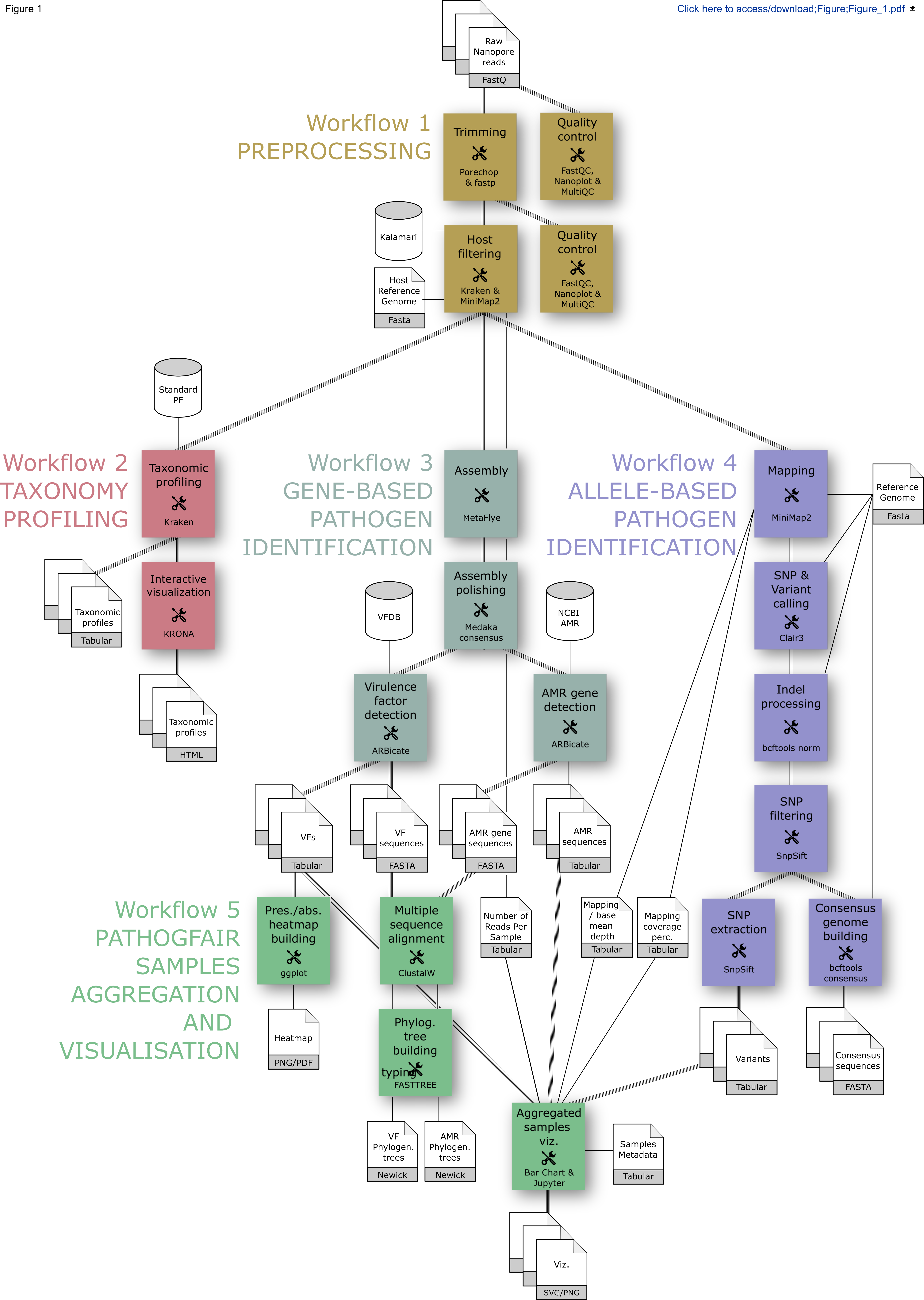

Figure 2

Click here to access/download;Figure;Figure_2.pdf

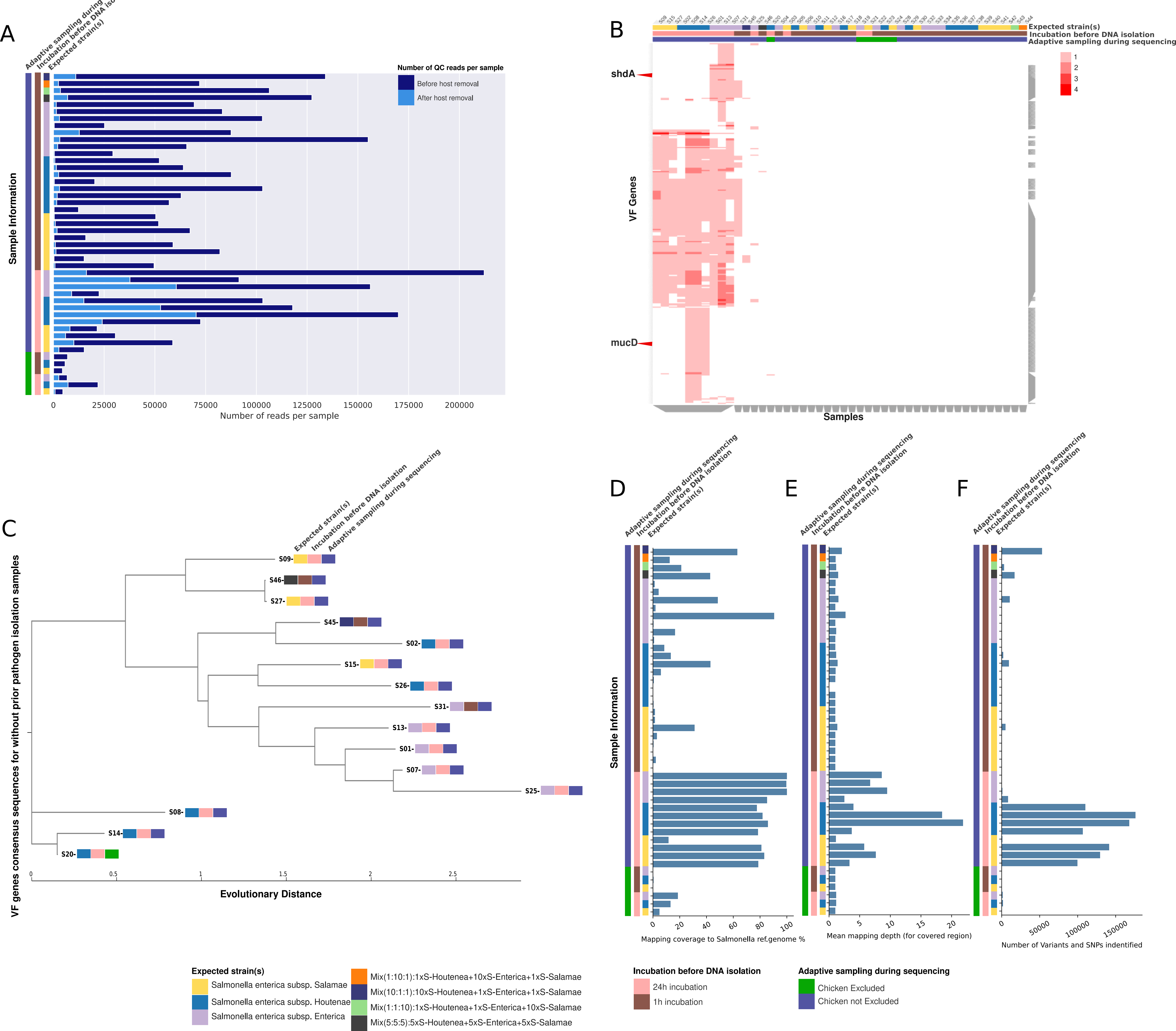

Click here to access/download;Figure;Figure_3.pdf

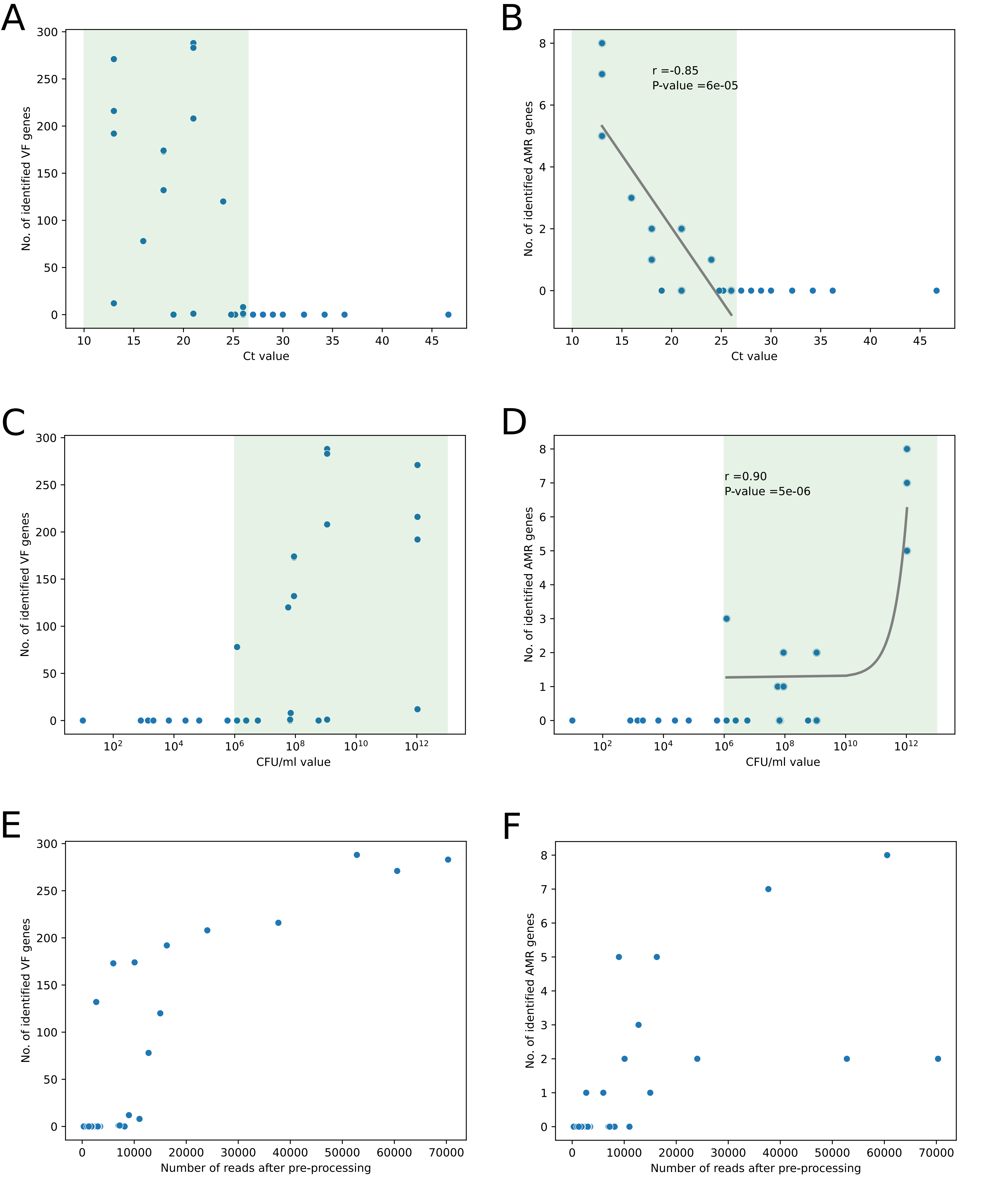

Figure 4

Click here to access/download;Figure;Figure_4.pdf

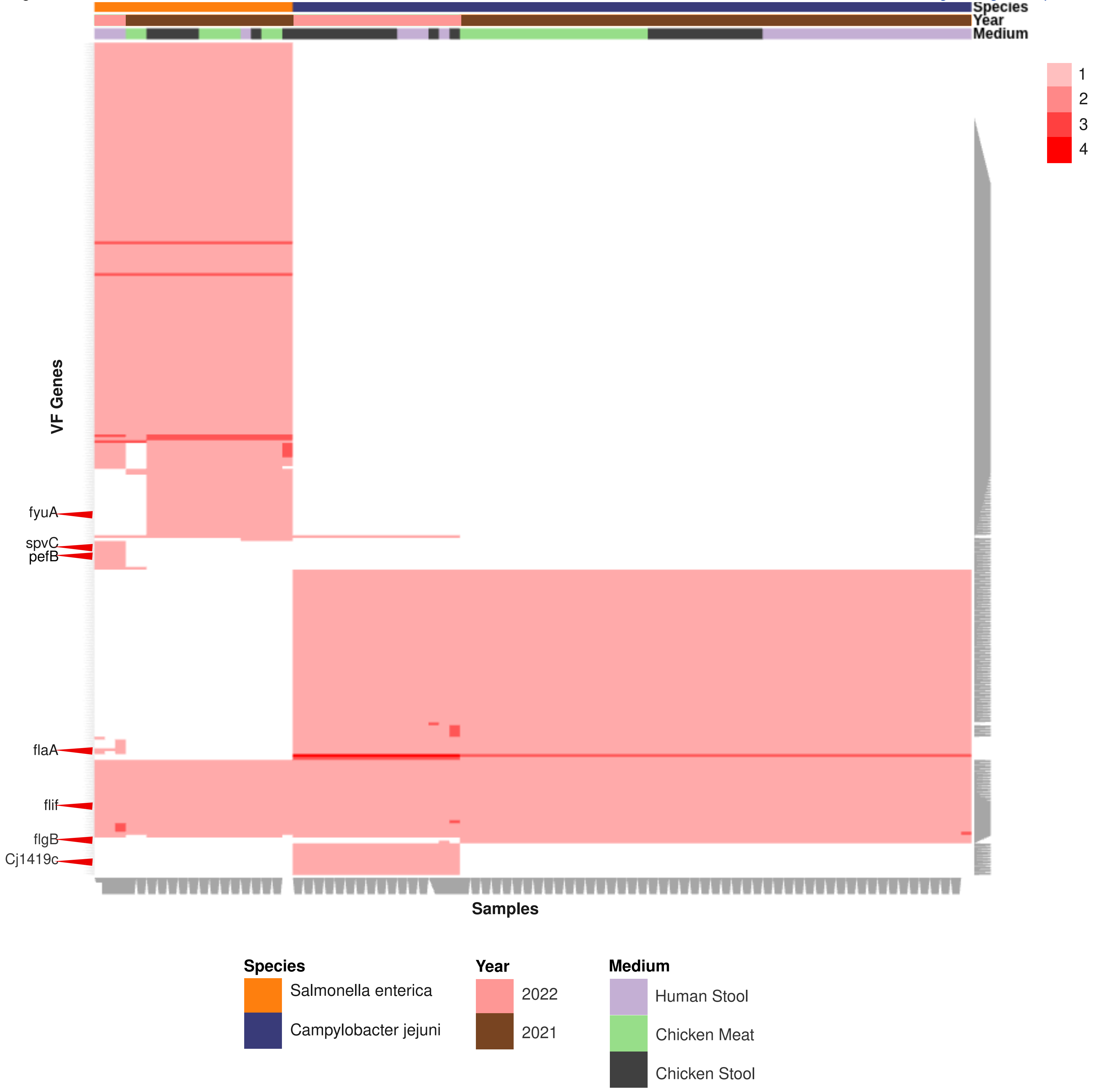

Supplementary Figure S1

Click here to access/download
Supplementary Material
Supplementary_Figure_S1.png

Supplementary Figure S2

Click here to access/download
Supplementary Material
Supplementary_Figure_S2.png

Supplementary Figure S4

Click here to access/download
Supplementary Material
Supplementary_Figure_S4.png

Supplementary Table S5

Click here to access/download
Supplementary Material
Supplementary_Figure_S5.pdf

Supplementary Figure S6

Click here to access/download
Supplementary Material
Supplementary_Figure_S6.png

Supplementary Table S8

Click here to access/download
Supplementary Material
Supplementary_Figure_S8.pdf

Supplementary Table S9

Click here to access/download
Supplementary Material
Supplementary_Figure_S9.pdf

Supplementary Table T1

Click here to access/download
Supplementary Material
Supplementary_Table_T1.tsv

Supplementary Table T2

Click here to access/download
Supplementary Material
Supplementary_Table_T2.tsv

Personal Cover Click here to access/download;Personal Cover;Cover_letter.pdf

Dear GigaScience Editorial Board,

We are pleased to submit our manuscript entitled "PathoGFAIR: A Series of FAIR and Adaptable (Meta)Genomics Workflows for (Foodborne) Pathogens Detection and Tracking", authored by Engy Nasr, Anna Henger, Björn Grüning, Paul Zierep, and myself, for consideration as a technical note.

Foodborne pathogen contamination represents a significant global health threat, affecting approximately 600 million people annually. Microbiological analysis is critical during foodborne outbreak investigations to detect and identify responsible pathogens and trace contamination sources. Metagenomic approaches provide a comprehensive overview of the genomic composition of microbial communities, enabling the detection of potential pathogens in samples. Coupled with user-friendly sequencing techniques like Oxford Nanopore sequencing, these approaches become faster and easier to implement. However, a major limitation is the lack of accessible, user-friendly, and openly available pipelines for pathogen identification and tracking from metagenomic data.

In this manuscript, we introduce PathoGFAIR, a suite of Galaxy-based FAIR workflows that utilise advanced tools for the detection and tracking of pathogens from metagenomic Nanopore sequencing data. Although primarily designed for the identification of foodborne pathogens, these workflows are versatile and applicable to any metagenomic Nanopore dataset. PathoGFAIR features several visualisations and comprehensive reports to ensure thorough analysis. We validated PathoGFAIR using 130 samples containing diverse pathogens from multiple hosts under various experimental conditions. Our workflows successfully identified and tracked expected pathogens at the species level in both isolated and non-isolated samples, given sufficient Colony-Forming Unit (CFU) and Cycle Threshold (Ct) values.

PathoGFAIR's integration into the Galaxy and WorkflowHub ecosystems ensures reproducibility and accessibility. Additionally, we have developed dedicated Galaxy training materials to support users in the interpretation of the workflows and provide ongoing assistance from the Galaxy and microbial communities.

We believe our integration efforts and the accompanying training materials meet the editorial guidelines on reproducible research, making this manuscript a suitable technical note for GigaScience. Given the increasing publication of pathogen protocols, we are confident that our manuscript will be valuable to a broad spectrum of GigaScience readers.

We confirm that this manuscript is original, has not been published previously, and is not under consideration by any other journal. The authors declare no conflicts of interest.

Thank you for your consideration.

Sincerely,
Dr. Bérénice Batut

Graphical Abstract
Click here to access/download;Graphical Abstract;graphical_abstract.pdf

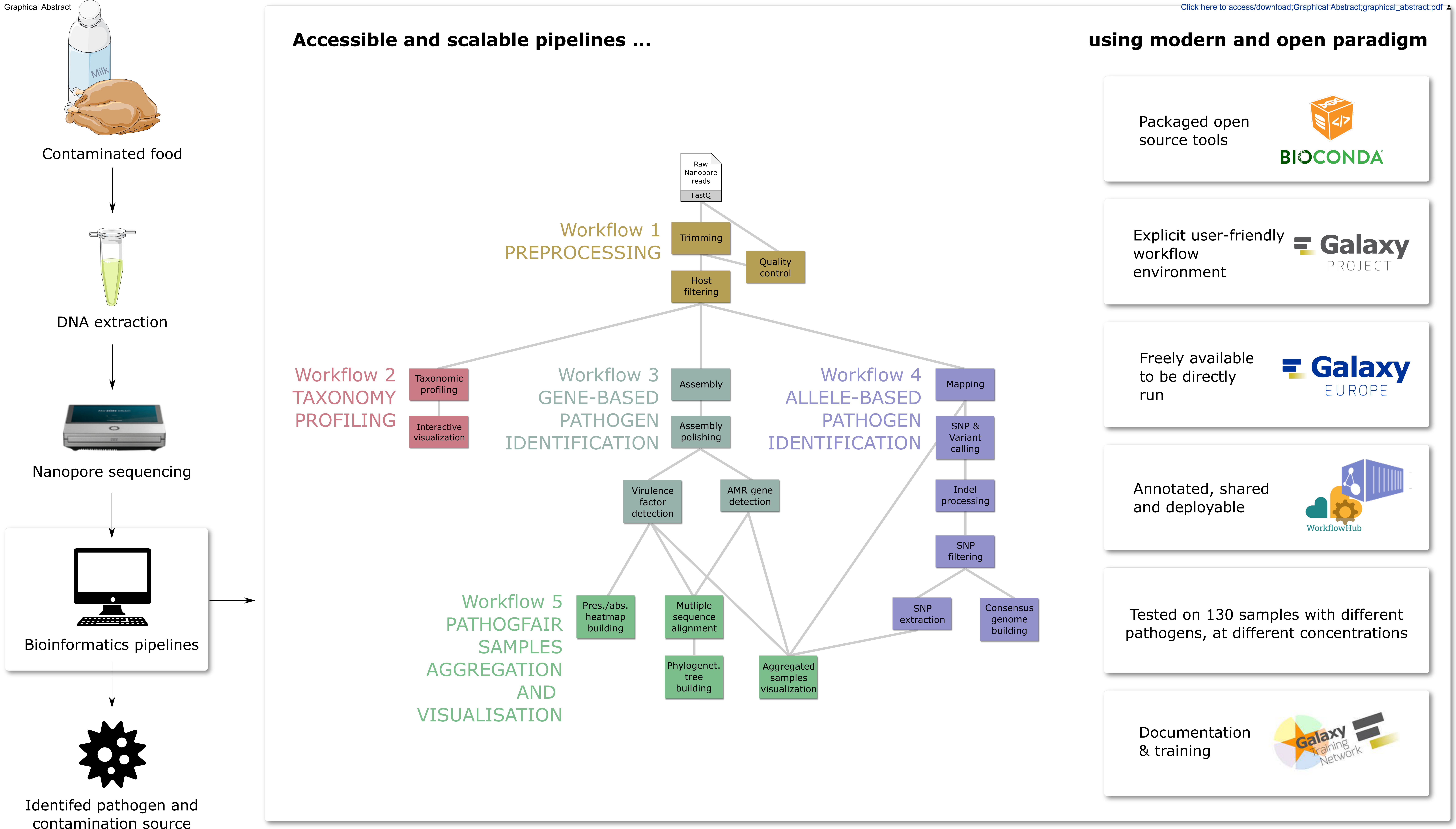

Supplement: giaf017_GIGA-D-24-00270_Original_Submission [file giaf017_giga-d-24-00270_original_submission.pdf]
