# Supplementary material for: PathoGFAIR: a collection of FAIR and adaptable (meta)genomics workflows for (foodborne) pathogens detection and tracking
Source: Gigascience. 2025 Sep 26;14:giaf017. doi: 10.1093/gigascience/giaf017 (PMC12466118; doi:10.1093/gigascience/giaf017)
Supplement: giaf017_GIGA-D-24-00270_Revision_1 [file giaf017_giga-d-24-00270_revision_1.pdf]

# GigaScience

## PathoGFAIR: a collection of FAIR and adaptable (meta)genomics workflows for (foodborne) pathogens detection and tracking

--Manuscript Draft--

| | |
|---|---|
| **Manuscript Number:** | GIGA-D-24-00270R1 |
| **Full Title:** | PathoGFAIR: a collection of FAIR and adaptable (meta)genomics workflows for (foodborne) pathogens detection and tracking |
| **Article Type:** | Technical Note |
| **Funding Information:** | EOSC-Life — Dr Bérénice Batut<br>Bundesministerium für Bildung, Wissenschaft und Forschung (031 A538A de.NBI-RBC) — Dr Björn Grüning<br>Ministerium für Wissenschaft, Forschung und Kunst Baden-Württemberg — Dr Björn Grüning<br>Agence Nationale de la Recherche (ANR-11-INBS-0013) — Not applicable |
| **Abstract:** | Background: Food contamination by pathogens poses a global health threat, affecting an estimated 600 million people annually. During a foodborne outbreak investigation, microbiological analysis of food vehicles detects responsible pathogens and traces contamination sources. Metagenomic approaches offer a comprehensive view of the genomic composition of microbial communities, facilitating the detection of potential pathogens in samples. Combined with sequencing techniques like Oxford Nanopore sequencing, such metagenomic approaches become faster and easier to apply. A key limitation of these approaches is the lack of accessible, easy-to-use, and openly available pipelines for pathogen identification and tracking from (meta)genomic data.<br>Findings: PathoGFAIR is a collection of Galaxy-based FAIR workflows employing state-of-the-art tools to detect and track pathogens from metagenomic Nanopore sequencing. Although initially developed to detect pathogens in food datasets, the workflows can be applied to other metagenomic Nanopore pathogenic data. PathoGFAIR incorporates visualisations and reports for comprehensive results. We tested PathoGFAIR on 130 samples containing different pathogens from multiple hosts under various experimental conditions. For all but one sample, workflows have successfully detected expected pathogens at least at the species rank. Further taxonomic ranks are detected for samples with sufficiently high Colony-forming unit (CFU) and low Cycle Threshold (Ct) values.<br>Conclusions: PathoGFAIR detects the pathogens at species and subspecies taxonomic ranks in all but one tested sample, regardless of whether the pathogen is isolated or the sample is incubated before sequencing. Importantly, PathoGFAIR is easy to use and can be straightforwardly adapted and extended for other types of analysis and sequencing techniques, making it usable in various pathogen detection scenarios. |
| **Corresponding Author:** | Bérénice Batut, Ph.D.<br>CNRS UAR 3601: Institut Français de Bioinformatique<br>Evry, FRANCE |
| **Corresponding Author Secondary Information:** | |
| **Corresponding Author's Institution:** | CNRS UAR 3601: Institut Français de Bioinformatique |
| **Corresponding Author's Secondary Institution:** | |
| **First Author:** | Engy Nasr |
| **First Author Secondary Information:** | |
| **Order of Authors:** | Engy Nasr<br>Anna Henger, Ph.D. |

Powered by Editorial Manager® and ProduXion Manager® from Aries Systems Corporation

| | |
|---|---|
| | Björn Grüning, Ph.D. |
| | Paul Zierep, Ph.D. |
| | Bérénice Batut, Ph.D. |
| **Order of Authors Secondary Information:** | |
| **Response to Reviewers:** | > We sincerely thank the editor and reviewers for their valuable suggestions and constructive feedback. Suggested changes to the manuscript have been implemented and highlighted in red for ease of tracking.<br><br>Reviewer #1<br><br>The authors present PathoGFAIR, a set of Galaxy workflows for the metagenomic analysis of shotgun Nanopore sequencing from isolated and non-isolated pathogens in contaminated food samples. They complement their work by analysing and releasing two datasets, one from isolated and the other from non-isolated samples, with the primary objective of illustrating the potentiality of the workflows. These datasets could also be used as benchmarks for future works.<br><br>The manuscript is generally well-written, and the authors highlight the advantages of the proposed workflows in Table 1 by comparing them to similar solutions. The workflows are well integrated into the Galaxy network, are available on the three main usegalaxy instances, and provide a thorough tutorial through the Galaxy training platform. A notable advantage of PathoGFAIR over similar workflows is that, thanks to Galaxy, the final user can easily tailor them by replacing any tool in the workflow with others available in the Galaxy ecosystem. This also allows easy updates for the tools in the workflows.<br><br>> We appreciate your insightful feedback and suggestions for improving our manuscript. Your comments are invaluable in helping us enhance the clarity and robustness of our work.<br><br>A few minor points that, if addressed, in my opinion, could further strengthen the manuscript:<br><br>1 - The rationale behind the tool selection in each of the four workflows is not always clear. While insights are present for workflows 1 and 4, this is not true for workflows 2 and 3. The reader would benefit from understanding why one tool has been preferred over another for the same task, even more so, given the possibility to modify the workflows easily, when this preference could be the other way around in particular use cases or conditions.<br><br>> We agree that the rationale behind tool selection should be clearer, particularly for Workflows 2 and 3. For Workflow 2 (Taxonomy Profiling), Kraken2 was selected due to its speed, high sensitivity, and ability to handle large reference databases, which is essential for the detection of diverse pathogens in metagenomic datasets. For Workflow 3 (Gene-based Pathogen Identification), ABRicate was chosen for its comprehensive virulence factor and antimicrobial resistance gene databases and ease of use in conjunction with the Galaxy platform.<br>> We have then revised the "Implementation" section of the manuscript to clearly explain the rationale for selecting Kraken2 for taxonomy profiling and ABRicate for gene-based pathogen identification in Workflows 2 and 3. The description now emphasises the tool's advantages for specific tasks and highlights when other tools could be appropriate depending on user needs or specific experimental conditions.<br><br>2—One of the main factors for a successful metagenomic analysis is the correctness, completeness, and up-to-dateness of the reference data. The authors should briefly describe how PathoGFAIR addresses this in Galaxy.<br><br>> Thank you for highlighting this critical factor. Ensuring the accuracy and currency of reference data is indeed fundamental for robust metagenomic analysis. PathoGFAIR leverages Galaxy’s integrated Data Managers, which enables Galaxy admins to provide up-to-date reference data. These Data Managers automate the download, installation, and regular update of essential reference databases, ensuring that |

PathoGFAIR users work with complete, accurate, and up-to-date reference information.
> PathoGFAIR workflows are configured to use well-maintained and reputable sources, such as NCBI and other public pathogen reference repositories e.g. StandardPlusPF, Kalamari, and VFDB, which further support accuracy and comprehensiveness. Additionally, Galaxy's user-friendly interface enables users to select preferred references or request the inclusion of specific databases via Galaxy administrators, adding to the workflow's adaptability for diverse use cases.
> We have added details in the "Implementation" section on PathoGFAIR's use of Galaxy's Data Managers, explaining how these tools support reliable reference data management. Furthermore, to illustrate the robustness of PathoGFAIR, we have benchmarked it against comparable systems and calculated sensitivity and precision metrics of PathoGFAIR with tested samples, now included in the manuscript to demonstrate PathoGFAIR's accuracy in pathogen identification, in an added "Benchmarking PathoGFAIR" section.

3—While this workflow is clearly stated to be tailored for shotgun metagenomic sequencing, the authors contrast this approach only with targeted sequencing. Instead, they should also discuss the 16s rRNA metagenomic approach, for which Nanopore kits are available, and why PathoGFAIR has been limited to the analysis of shotgun data.

> While Nanopore 16S rRNA sequencing kits are indeed available, PathoGFAIR was intentionally developed for shotgun metagenomic sequencing due to its broader scope in pathogen identification. Unlike 16S rRNA sequencing, which is limited to bacterial taxa, shotgun metagenomics enables the comprehensive detection of a broader range of pathogens, including bacteria targeted in targeted in our workflows, as well as viruses, and fungi, which could be easily incorporated in the future without requiring predefined amplicon targets.
> Moreover, shotgun metagenomics allows access to complete genomes rather than specific gene regions, facilitating taxa-agnostic identification of antimicrobial resistance (AMR) and virulence genes. This capability is central to PathoGFAIR's functionality, as it enables detailed characterisation and tracking of pathogens beyond taxonomic profiling.
> We have updated the introduction to include a comparison between shotgun metagenomic and 16S rRNA sequencing, clarifying why shotgun metagenomics offers the broader capabilities required by PathoGFAIR.

Reviewer #2

Nasr and colleagues present an, at times, well-written manuscript with an interesting and robust pipeline that includes well-known softwares (you must make sure to cite the authors of these).

> We sincerely thank the reviewer for their insightful feedback and constructive comments.
> We have ensured that all software tools mentioned in the manuscript are properly cited, with appropriate references to their original authors.

However, the manuscript is, quote "...a collection of Galaxy-based FAIR workflows employing state-of-the-art tools to detect and track pathogens from metagenomic Nanopore sequencing". Its repeated how well it works, they even compare it to other software in table 1 (without proper benchmark). These initial statements are however not supported by the findings. The Salmonella from the spiked samples are, as expected from food matrix present in low quantity), difficult to do more than state that the genus is present, and only a fraction of the samples can actually "complete" the entire pipeline. Also, the benchmarking is not really benchmarking (compare and measure this software against other competing software). No such comparison is done, and even though the intention of PathoGFAIR as stated throughout the paper, is detection and analysis of metagenomic samples, the benchmarking is done on isolate based wgs.

> Table 1 is a feature comparison rather than a performance benchmark. Its purpose is to highlight key aspects of PathoGFAIR, such as modularity, reproducibility, ease of

use, and adherence to FAIR principles (Findability, Accessibility, Interoperability, and Reusability). These features are crucial for making PathoGFAIR accessible and adaptable for pathogen detection in diverse metagenomic contexts. Unlike a performance-focused benchmark, Table 1 emphasises design and usability differences between PathoGFAIR and other pipelines.
> The manuscript introduces PathoGFAIR as a collection of FAIR workflows and presents its core capabilities through two distinct use cases. It is intended as a technical note, focusing on system design, workflows, and practical application, rather than a direct performance comparison with competing pipelines. We appreciate your suggestion and have expanded our efforts to include a performance benchmark using the spiked non-isolated samples from the first use case. This benchmark compares PathoGFAIR with the pipelines in Table 1 and evaluates their performance. Results are presented as a heatmap (Supplementary Figure S9) in the newly added “Benchmarking PathoGFAIR” section, accompanied by detailed explanatory paragraphs.
> Additionally, we have included a replication guide in the PathoGFAIR GitHub repository and a protocol on protocols.io detailing the benchmarking steps. These additions ensure transparency and reproducibility of the benchmarking process.
> We have substantiated all statements about PathoGFAIR’s performance with findings from the use cases and the new benchmarking sections. For instance, in the first use case, pathogens were detected with a success rate of 97.8% at the species rank, 60.9% at the subspecies rank, and 13% at the strain rank. PathoGFAIR successfully identified pathogens in all tested samples but one across both use cases and the benchmark. It is important to note that samples do not need to complete the entire pipeline for pathogen identification. Pathogen detection occurs as early as the second workflow (Taxonomy profiling), while subsequent workflows provide additional analyses, such as pathogenicity characteristics and visualisation. This modular design allows workflows to function independently or in parallel, ensuring flexibility and efficiency for various metagenomic analyses.
> Finally, we agree that further benchmarking with additional metagenomics datasets could provide deeper insights into PathoGFAIR’s performance and will explore this in future work.

It is also evident that the authors are not microbiologists as the manuscript is riddled with taxonomical misunderstandings about the vast genus Salmonella and when to use capital letters and italics.

> Thank you for highlighting the taxonomic inconsistencies. We have carefully reviewed and revised the manuscript to ensure that all taxonomic names, including Salmonella and other genera, as well as their subsequent taxonomic ranks, are correctly formatted according to scientific conventions (e.g., italicization and capitalization). These have been applied consistently throughout the text and figures, following the guidelines outlined in this reference (https://www.ncbi.nlm.nih.gov/pmc/articles/PMC86943/). We appreciate your attention to this detail.

I am also lacking a proper discussion here on the results found in the spiking experiment in light of current EU legislation on Salmonella. Can this pipeline help in this regard?

> Thank you for raising this important point. The primary aim of our study was not to propose an alternative to EN/ISO 6579, as such a method would require a comprehensive validation process, including sensitivity analyses, to meet regulatory standards. Instead, our focus was on the development and optimisation of a bioinformatic workflow for the detailed characterisation of microbial species, including subspecies rank resolution, within a food matrix using whole genome sequencing (WGS).
> EN/ISO 6579 specifies an enrichment step followed by classical microbiology or real-time PCR for confirmation of Salmonella presence. In contrast, our workflows aim to analyse the enrichment broth directly, leveraging WGS to characterise all Salmonella present in the sample without requiring single-strain isolation. This approach offers the added benefit of insights into subspecies diversity and potential links to previous outbreak strains
> The spiking experiment presented in our study served as a proof of concept,

Powered by Editorial Manager® and ProduXion Manager® from Aries Systems Corporation

demonstrating the feasibility of detecting and characterising Salmonella in complex food matrices using WGS. This lays the foundation for future applications where detailed subspecies-rank information is critical.
> We have added a paragraph in the conclusion section to further discuss the potential applications of this approach in light of current EU legislation on Salmonella and its possible role in supporting outbreak investigations and traceability efforts.

Sensitivity and specificity metrics are also lacking.

> We have now included sensitivity matric for PathoGFAIR at the end of both use case sections in the manuscript. As we explain in the text, specificity matric can not be applied because both use cases do not include true negative samples, making specificity calculations inapplicable in this context. These additions provide a clearer evaluation of PathoGFAIR's performance and ensure that the results are appropriately framed given the experimental design.

Abstract

"foodborne pathogen data" / "metagenomic Nanopore pathogenic data" - suggest to rewrite, as what I think you are trying to say is " initially developed to detect foodborne pathogens from metagenomic nanopore data, the workflow can be used to detect any pathogen."

> Thank you for your suggestion. We have updated the abstract to provide clearer phrasing, incorporating your feedback. The revised text now accurately conveys that while the workflow was initially developed to detect foodborne pathogens from metagenomic Nanopore data, it is versatile and can be used to detect a wide range of pathogens.

"Colony-forming unit and Cycle Threshold values." rewrite sentence, I do not completely understand what you are trying to say. what is "sufficient colony forming units?" It will vary as well between pathogens (infection dose varies). You could rather state your sensitivity of the pipeline here - even though i think that sampling prep, library prep and seq influences that more than the bioinformatics.

> We agree that Ct and CFU values are closely tied to sample preparation and library preparation, which significantly impact the performance of any bioinformatics workflow. In the first use case (samples without prior isolation), where these values were provided as part of the metadata, we examined their influence on our workflows performance. Specifically, we observed that higher CFU/ml values and lower Ct values improved the comprehensiveness of pathogen detection, particularly for challenging samples like those in Use Case 1 (spiked samples)
> Rather than defining an absolute threshold for sensitivity, we focused on how these parameters influenced the bioinformatics outcomes and we provided practical recommendations for users. These recommendations highlight the conditions under which optimal sensitivity and accuracy can be achieved when using PathoGFAIR, emphasizing the importance of proper sample preparation and sequencing.
> To address your suggestion, the sentence in the abstract has been revised to better reflect the role of Ct and CFU values in influencing workflow outcomes. Additional clarification has been added to the use cases and conclusion sections, detailing the sensitivity and best practices for pathogen detection.

"In any sample": did you test all matrixes?

> We meant to say "in all tested samples within the two use cases". The sentence has been rewritten for clarity to avoid any misinterpretation.

"sample is isolated or incubated before seq" you cannot isolate a sample, but you isolate a bacteria from a sample. unprecise language.

> Thank you for pointing out the imprecise language. We have revised the sentence to accurately describe the isolation of bacteria from a sample.

Introduction

Powered by Editorial Manager® and ProduXion Manager® from Aries Systems Corporation

In general, its well written, but a bit unprecise here and there. The authors also rely a lot on the following words: "rapid" "accurate".

> Thank you for your feedback. We have carefully revised the introduction to enhance clarity and precision. Redundant terms such as “rapid” and “accurate” have been removed and more specific descriptions have been provided where necessary to ensure greater precision in the language.

"outbreaks and epidemics" - rewrite, these are the same.
"efforts to mitigate their spread and ensure food safety" again, complementary terms - rewrite.
"global public health authorities" we do have everything from local to global food safety and public health authorities, I think one should highlight this. There is a difference between for instance EFSA and ECDC.
"The utilisation of Nanopore sequencing data, as exemplified in studies like [7]," citing practices like this is not really reader friendly. Suggest to write what they actually did in seven (as for instance the detection of blah in blah as shown in 7).
"Once (meta)genomics data has been generated, bioinformatics approaches enable the rapid and accurate detection"; repetition of chapter above. You write in the former chapter that "the utilisation of nanopore data" which also includes bioinformatics of course.

> Thank you for your suggestions. We have revised the manuscript to address your points.
> The terms "outbreaks and epidemics" have been clarified to avoid redundancy.
> The phrase "efforts to mitigate their spread and ensure food safety" has been reworded to provide more distinct and complementary descriptions.
> We have highlighted the range of food safety and public health authorities, specifying the differences between local, national, and global organizations, such as EFSA and ECDC.
> The citation of study [7] has been updated to more clearly describe the findings, such as the utility in closing genomic gaps with real-time, long-read sequencing data for outbreak investigation as shown in the referenced study.
> The repetition in the chapter regarding bioinformatics and Nanopore sequencing has been removed, ensuring the flow of the manuscript is smoother and avoids redundancy.

"isolation can be complex"? do you mean complicated or work intensive?

> “Work-intensive” is indeed a more accurate description of what we meant. We intended to convey that the isolation process is laborious and time-consuming, often involving multiple steps and advanced techniques. We have revised the sentence to make this clearer.

SURPI and Sunbeam is freely available?
https://microbiomejournal.biomedcentral.com/articles/10.1186/s40168-019-0658-x
https://chiulab.ucsf.edu/surpi/

> Thank you for your comment. As noted in Table 1, SURPI and Sunbeam are freely available. We have clarified the text to ensure this is clearly stated.

"PathoGFAIR: pathogen identification and tracking from metagenomics". Im not convinced that it can perform tracing in an outbreak where only a few SNPs are allowed.

> Thank you for your feedback. By tracking, we are referring to monitoring the presence of the pathogens across samples using sample metadata such as location and time. PathoGFAIR enables users to identify when and where a pathogen appears in the sample based on the metadata.
> We do not mean ‘tracing’ in the context of an outbreak where only a few SNPs are involved. While PathoGFAIR primarily focuses on pathogen identification, we recognise that additional tools such as MLST and SNP-based tracing would significantly enhance its ability to trace pathogens at finer taxonomic ranks, including subspecies and strain-

rank differences. We have acknowledged this potential extension in the conclusion section of the manuscript.

PathoGFAIR does not really speed up the process of sampling, does it. Actually, it takes more time to extract crude dna from a sample than to place it in a enrichment broth or do a dilution series, so the presteps are not really a part of this.

> You are correct that PathoGFAIR does not affect the actual sampling or DNA extraction time, as it focuses on the bioinformatics analysis. The primary aim of the samples in this study was to demonstrate the functionality of PathoGFAIR and to provide recommendations for best practices in downstream analysis. The pre-steps, such as DNA extraction or enrichment broth preparation, are outside the scope of PathoGFAIR's capabilities.

"Tracking pathogens" - again, if species level is the lowest rank it can go to, its not enough to perform tracking.

> We have clarified the explanation in the manuscript to distinguish between "tracking" and "tracing".
> Tracking refers to monitoring the presence of a pathogen across all given samples, focusing on when and where it appears, using sample metadata such as time and location. Tracing refers to more detailed methods, such as SNP or MLST analysis, which can identify finer taxonomic differences, including strain-level or subspecies-level distinctions.
> This distinction should help avoid any confusion between the two terms in the context of our study.

Implementation

Overview

"input data is seq data generated w nanopore" basecalling is not included in the workflow?
How is this performed? It affects the quality of the reads, so its nice to know what you did.

> Thank you for your comment. As outlined in our published protocol for sample preparation (Step 8: https://www.protocols.io/view/samples-preparation-for-foodborne-pathogen-detecti-8epv5x1jdg1b/v1?step=8), as well as in the manuscript's first use case (Samples without prior isolation), real-time basecalling was performed using the MinKNOW software with the MinION Mk1C device. We recognize that basecalling is a crucial step, as it affects the quality of the reads, and we have now added a brief explanation in the overview section to clarify how this is handled in our workflow.

The chapter is very wordy, and contains a lot of fill-words with salespitches almost. I would recommend rewriting it, for instance:
Chapter that starts with subsequently and describes the different workflows and how they work together can be compressed.
And the last three sections are salespitching.

> To address the concern regarding the wordiness, we have condensed the section describing how the workflows interconnect. We have focused on providing a more concise explanation while retaining the essential technical details. Additionally, we have removed any language that could be perceived as overly promotional. However, we believe it is important to maintain the discussion of how workflows work together, as this highlights the flexibility and modularity of PathoGFAIR.

WF1: Preprocessing

How stringent filtering and quality control are implemented in the workflow? How good quality do you need for the wp2-4 to work sufficiently well? Did you test?

> Thank you for your question. Quality control in PathoGFAIR is performed using the Fastp tool, which removes low-quality reads and adapters. For downstream analyses,

we set a quality score of Q20 or higher, which is generally sufficient for Nanopore data (https://journals.asm.org/doi/10.1128/jcm.01631-22). However, we have not tested alternative quality filters with our use cases.
> We visualise quality control results, including read length distribution and base quality scores, using MultiQC, which allows users to assess the quality before proceeding to the subsequent workflows. In Use Case 1 (spiked samples), we found that samples with fewer than 2,700 reads after preprocessing were excluded from further analyses in Workflows 3 and 4, as they failed to generate the necessary contigs. This cutoff can be adjusted based on the user's datasets. While we have not tested higher quality score thresholds, users can modify the parameters to suit their data's characteristics.

Food vehicle animal? What is that - do you mean that if you extract dna from bovine meat, you map to bovine genome?

> Thank you for pointing out the unclear terminology. The term "food vehicle animal" refers to the animal from which the food product originated. For example, in the case of chicken meat, the food vehicle animal is the chicken. To make this clear, we have updated the terminology to "host" or "food source".
> Regarding mapping to the host genome, when DNA is extracted from a food product like bovine meat, we map the reads to the bovine genome to remove host-derived sequences. This ensures that only non-host (potentially pathogen-related) sequences are retained for downstream analysis. The user can select the appropriate host genome from a drop-down list before running the workflow.
> We have updated the terminology and provided clearer examples of host genome removal for various food types in the manuscript.

"a tool ten times faster etc etc." is discussion and should be removed from what I think is materials and methods even though the title of the section is workflow 1.

> We respectfully disagree with the suggestion to remove the performance-related details about the tool being "ten times faster."  This information is essential for providing context about the tool selection in Workflow 1. Performance metrics, such as speed and computational efficiency, are integral critical considerations in the design of the workflow and help justify why specific tools were chosen over others. Therefore, we believe it is important to include this information in the description of Workflow 1 to give a complete understanding of its implementation.

What is a food host?

> The term "food host" was used to refer to the food product that may be contaminated by a pathogen (e.g. chicken, cow, milk, etc.). To improve clarity, we have updated the terminology to simply "host" and provided clearer examples of how host genome fragments are removed for various food types during analysis.

Kalamari database includes many foodborne pathogens, such as Shigella, E. coli, Campylobacter etc etc. how can you just remove all reads that match to this database?

> Thank you for your comment. In Workflow 1, we use Kraken2 along with the Kalamari database to filter out host-associated sequences (e.g., cow, chicken, etc.) rather than pathogen sequences. Specifically, we select taxIDs related to probable food sources (like cow, and chicken) to remove host genomic DNA, while ensuring that pathogen-associated sequences are retained. The Kalamari database includes foodborne pathogens such as Shigella, E. coli, and Campylobacter, but these sequences are not removed in this step.
> We have clarified this distinction in the manuscript, highlighting that the primary aim of using the Kalamari database is to eliminate host-related contamination while preserving pathogen-related sequences for further analysis.

Table 1:  Innuendo is based on isolate WGS, and not intended for WGS. Also, it has its own built in wgMLST schema employed using chewbbacca, so it definitely has allele-abased pathogen identification. Its intended for illumina data. Victors are strictly a platform to analyse virulence factors and not intended even for taxonomic profiling, and its webinterface doesn't work. IDseq has step-by-step guides available on their

Powered by Editorial Manager® and ProduXion Manager® from Aries Systems Corporation

webpage, so I think that qualifies as a tutorial. You can also contact them (user support). I guess the same is true for OneCodex, as you actually pay for that one. So the table is unprecise at best and should be corrected (I didn't go through Submeam, SURPI or PAIPline specs to try to check if you got it correctly).

> Thank you for your detailed feedback on Table 1. We have carefully reviewed the specificity of each of the pipelines, and we appreciate your insights, particularly regarding Innuendo, Victors, IDseq, and OneCodex. Below are the changes we have made based on your feedback
> Innuendo: We have corrected the table to clarify that Innuendo is specifically designed for isolate WGS data and not metagenomic samples. We have also updated the entry to indicate that Innuendo utilises allele-based pathogen identification. Additionally, it is intended for Illumina data, not Nanopore sequencing.
> Victors: As you mentioned, Victors is strictly focused on analysing virulence factors and is not intended for taxonomy profiling. We have revised the table to reflect that it is only suitable for gene-based pathogen identification. The * next to the web interface entry already indicated that it malfunctioned when tested, which remains unchanged.
> IDseq: While IDseq does provide step-by-step guides on its webpage, which could qualify as a tutorial, we have updated the table to clarify that these guides are considered documentation rather than a full tutorial, as we define tutorials (e.g providing test datasets and teaching material such as those available in Galaxy Training Network (GTN). We have also acknowledged that IDseq offers user support
> OneCodex: As you correctly point out, OneCodex offers user support as it is a paid service. We have updated the table to reflect this and have noted that it also provides user documentation.
> In addition to these specific updates, we also took this opportunity to re-verify all information in the table by consulting documentation, websites, and published papers for all listed tools, including SURPI, Sunbeam, and PAIPline. This thorough review ensures the accuracy of the table entries, and we have applied these corrections across all pipelines and systems.

Rewrite this. Further, I think you should only include systems / pipelines that are intended for metagenomics.

> Thank you for your feedback. Our intention was to provide a comprehensive comparison of systems and pipelines used for pathogen identification, which includes not only those focused specifically on metagenomics but also those applicable to isolate-based sequencing. However, we understand your concern about focusing solely on metagenomic pipelines.
Additionally, we have updated the comparison to include (BugSeq [https://bugseq.com/analysis#pathogen-identification]) which performs similar analyses to our workflows, albeit it is not free or openly available. This highlights the accessibility advantage of PathoGFAIR.

You have a footnote * that I cannot see in the table as well.

> The footnote (*) refers to the Victors pipeline and is located in the third row of the table. We have made sure to clarify this in the revised version of the manuscript.

WF2 taxonomy profiling

The first sentence needs rewriting.

> We appreciate your suggestion. We have revised the first sentence to improve clarity and ensure it better conveys the intended message.

Two sentences from "Although Kraken2 is a tool design…….." belongs in discussion.

> Thank you for your comment. As we do not have a separate discussion section, we believe it is important to explain the rationale behind our tool choices within the implementation section. This allows us to highlight the specific features of each tool, such as Kraken2, that make them suitable for our workflows, without delving into the results.

WF3

Medaka consensus pipeline : "This task is performed using neural networks applied from a pileup of individual sequencing reads against a draft assembly. " what draft assembly did you use here to create a consensus sequence? Actually, its not polishing contigs, its assemblying them?

> To clarify, Medaka is used to polish the consensus sequences generated from contigs, rather than performing the assembly itself. The contigs are first assembled using a different tool, and Medaka is then applied to refine and improve the accuracy of the consensus sequence based on the individual reads.

Again, there is some descriptions of the software which belongs in the discussion, say the perks one gets from using this tool over the other.

> Thank you for your feedback. As previously mentioned, the implementation section is designed not only to explain the workflows but also to justify the choices made in selecting the tools used. This includes highlighting the advantages of specific tools within the context of our workflows, as these decisions are integral to understanding the functionality and efficiency of the system.

I do not however get how screening for virulence genes = pathogen identification. The thing is that in a complex food matrix or faecal samples from animals, things like stx phages will also be present. These are not stec pathogens unless the phage is inside an e.coli. How do you make sure of the host for such mobile genetic elements as these virulence and amr genes often are located on? Seeing as this is the basis of your pathogen detection?

> Thank you for this insightful comment. We fully recognize the complexity of detecting virulence and AMR genes, especially when considering mobile genetic elements (MGEs) like phages and plasmids, which can carry these genes independently of their host.
> While it is true that VF and AMR genes can be located on MGEs, these genes are still highly informative markers for identifying potential pathogens. In foodborne outbreak contexts, the presence of these genes often correlates strongly with pathogenic organisms (https://pmc.ncbi.nlm.nih.gov/articles/PMC6653787/).
> Our workflows do not directly cross-verify associations for MGEs, but they generate comprehensive outputs that allow users to conduct such analyses. For example, Workflow 2 (Taxonomy Profiling) generates taxonomic abundance data, including species and subspecies identification using Kraken2, which can be used to identify the host organism. Workflow 3 (Gene-based Pathogen Identification) identifies virulence and AMR genes within the sample. By comparing the taxonomic profiling results from Workflow 2 with the VF and AMR gene data from Workflow 3, users can validate the relevance of detected genes and minimise the risk of misidentifying genes located on MGEs.
> In our use cases, we had prior knowledge of the expected pathogens (e.g., Salmonella and Campylobacter), and the species identified in the samples matched the expected results. For example, in Workflow 4 for the second use case, high mapping coverage (>90%) for both Salmonella and Campylobacter confirmed that the VF and AMR genes detected were linked to correct bacterial hosts, as identified by Workflow 1.
> We acknowledge that additional measures could enhance the accuracy of this process. In future versions of PathoGFAIR, we plan to introduce a validation step to cross-reference VF and AMR gene detection with broader taxonomic markers (e.g., 16S rRNA), ensuring that genes detected from MGEs are correctly attributed to the pathogenic organism. We mentioned these potential improvements in the conclusion section of the manuscript.

WF4

A bit again on choosing software over the other that is discussion food.

> As we mentioned previously, the implementation section is designed to provide not

only a description of our workflows but also the rationale behind our tool selection. We believe it is important to explain why specific tools were chosen based on their features, performance, and suitability for the workflows, as this contextual information supports the reader's understanding of our approach.

Wf4/wf5

I am worried about the reliance on snp based technics for nanopore reads. Is the quality good enough to achieve sufficiently robust results?

> Thank you for raising this important point. We acknowledge that SNP-based techniques for Nanopore reads can be challenging due to higher error rates compared to short-read sequencing.
> To address this, we enforce stringent quality control measures and apply a minimum depth of covering threshold of 10x to ensure reliable SNP calls. This minimises the inclusion of false-positive variants caused by sequencing errors.
> Additionally, we use Clair3, a state-of-the-art variant caller specifically optimised for Nanopore long-read sequencing. Clair3 leverages deep learning to enhance the accuracy of SNP and small indel detection, making it a robust choice for long-read data.
In our workflows, SNP-based analyses have been successfully applied in use cases with known pathogens, where results matched expected outcomes, providing additional confidence in the reliability of this approach.
> We have updated the manuscript to include these details, explicitly addressing the robustness of SNP-based techniques in the context of Nanopore reads and the steps taken to ensure accurate results.

Easily adaptable workflows

Last section is repetition (about each wf operating independently)

> Thank you for your observation. While the final section does revisit points mentioned earlier, it serves a distinct purpose: to summarise and emphasise how the workflows can operate independently or in combination, offering flexibility to the users. This summary is intended to provide a clear take-home message for readers about the modular design of PathoGFAIR and its practical applications. We believe this is an important inclusion to ensure users understand how to best utilise the workflows for their specific needs.

Use cases

Samples Without Prior Pathogen Isolation

Data generation

Please revise how to write Salmonella names correctly. They should be in italics for genus, species and subspecies names, while the serovar/serotype is non italic and capital letter. So the correct term would be:
* Salmonella enterica subsp. enterica serovar Houtenae, or in short; Salmonella Houtenae.
* The strain DSM554 is of serovar Typhimurium, and this should referenced like this: Salmonella enterica subsp. enterica serovar Typhimurium strain DSM 554

> Thank you for highlighting this important correction. We have reviewed the manuscript thoroughly to ensure that all mentions of Salmonella species, subspecies, and serovars are formatted according to the correct nomenclature standards. Additionally, any occurrences in figures and figure captions have been updated accordingly. We appreciate your attention to detail, and the manuscript now adheres to the proper scientific conventions for nomenclature.

First two sentences are contradictory to eachother?

> Thank you for pointing this out. We understand how the original phrasing could appear contradictory. Upon review, we have clarified the text as follows:

> "In this study, all pathogens in the samples were known beforehand. For non-isolated samples, the pathogens were deliberately spiked to simulate real scenarios, while for isolated samples, prior identification ensured the pathogen's identity."
> This revised wording eliminates ambiguity and ensures consistency in describing the experimental setup. We appreciate your feedback in helping us improve the clarity of our manuscript.

Sentence starting "15 samples were incubated"; don't start sentence with number, it looks like 33.15

> Thank you for pointing this out. The sentence has been revised to avoid starting with a number. It now reads:
> "A total of 15 samples were incubated..."
> We appreciate your feedback in improving the readability of the manuscript.

How much meat did you use? What CFU/g does these ct values translate too? Its important to know the sensitivity relative to legislation. The limit is zero in 100 grams, but I don't assume you tested 100g?

> Thank you for your insightful comments. We have updated the text to clarify the experimental details and address your questions directly.
> This study primarily focused on developing and validating a bioinformatics workflow for characterising microbial species down to the subspecies rank within a food matrix using whole genome sequencing. The main objective was to enhance traceability for outbreak investigations rather than establish specific detection limits.
> To address your questions:
> - How much meat was used? Chicken samples of 25g each were used for the analysis, in line with the EN/ISO 6579 method for Salmonella detection.
> - What CFU/g do these Ct values translate to? This study did not directly calculate CFU/g from Ct values, as the primary goal was proof-of-concept testing of bioinformatics workflows. Instead, we spiked samples with known concentrations of Salmonella enterica (representing three different subspecies) to test the pipeline.
> The workflow was tested on both enriched and unenriched samples spiked with 2 different Salmonella concentrations of 3 different Salmonella enterica subspecies:
> - Enriched samples were incubated for 24 hours following the EN/ISO 6579 protocol to exponentially increase the Salmonella concentration.
> - Unenriched samples (marked as "1h" in the manuscript) were processed immediately after preparation without an enrichment period. While we did not specifically define the processing time for unenriched samples, it included handling and immediate testing steps.
> We hope these clarifications sufficiently address your concerns. If further detail is needed, we are happy to expand the discussion.

What does adaptive sampling mean? To exclude chicken DNA?

> Thank you for pointing this out. We acknowledge that the term "adaptive sampling" should be clarified when it is first introduced in the manuscript. We have now revised the text to explicitly explain adaptive sampling when it is first mentioned. Specifically, we state that adaptive sampling is a feature of Nanopore sequencing that enables selective sequencing by enriching specific DNA regions of interest (e.g., microbial DNA) while depleting unwanted sequences (e.g., host DNA, such as chicken DNA). This clarification ensures that readers unfamiliar with the term understand its relevance and application.

The point v sentence under description of supplementary table t1 is a bit weird punctuation

> We have revised the sentence to make it clearer.

Gene-based pathogen identification

Working with meat to detect low abundance pathogenic bacteria is challenging without enrichment of the expected pathogen with selective methods. Just incubating it a x temperature might work for some bacteria, but others need special atmosphere

(campylobacter, clostridia) and nutrients. How do you accommodate this?

> Thank you for pointing that out. We have updated the text to clarify that our workflows do not take experimental design into account. While we provide recommendations for interpretation, we do not factor in specific experimental conditions, such as temperature or atmosphere, in the analysis.

Figure 2 B:
The grey bares samples ? why are they collapsed in the left corner?

> Thank you for the questions. We have revised the figure legend and description to better explain the Clustergram visualisation, emphasising the role of the similarity slider in the clustering process and how the grey bars represent samples clustered based on metadata categories. Additionally, we have included a note about the interactive version available on the PathoGFAIR GitHub repository, where users can adjust the clustering granularity and explore the data further. This should help clarify the appearance of the collapsed samples and provide context for how users can engage with the figure.

And shy are sdhA and mucD highlighted?

> We highlighted sdhA and mucD because they were used as key examples in the main text to illustrate our findings and provide context for interpreting the results. Highlighting these genes allows readers to easily connect the visual representation to the specific examples discussed in the manuscript.

Also, please put genes in italics.

> Thank you for pointing that out. We have now italicised the gene names in both the text and the figures as requested.

the grey bars on the right (y-axis) are not annotated?

> The grey bars on the y-axis represent clusters of Virulence Factor (VF) genes, and due to the large number of genes (over 2,100), annotating each one within the figure would be too cluttered. However, we have made sure to annotate and highlight the VF genes discussed as examples in the use case sections to provide clarity and context for the results.

To which reference genome are the barplot in d referring to? I can see for instance in f
trs of genes hard to annotate them all, however, we annotated the ones we mention as examples in the use cases sections, explaining the results.
hat there is a number of snps or variants for the Houtenae and Typhimurium, but not Salamae, was the latter used as reference?

> Thank you for your comment. We used Salmonella enterica subsp. enterica serovar Typhimurium (NC_003197.2) as the reference genome for all analysis results of Workflow 4 on the first use case. The bar plots in panel D and the SNPs/variants in panel F were derived based on this reference genome. The observed differences in SNP numbers, particularly between Houtenae and Typhimurium, are largely influenced by the enrichment process (incubation period before sequencing).
> We chose Typhimurium as the reference genome due to its well-annotated and widely used genome in genomic studies, which allows for consistency across samples. However, we recognise that using a reference genome more closely related to the specific subspecies in the samples (e.g., Salmonella salamae) could potentially provide more accurate and comprehensive SNP detection results.
> In Workflow 4, selecting a reference genome is an input choice that users make before running the analysis. The Galaxy interface allows users to select any reference genome from a drop-down menu or upload their own reference genome if it is not available in the list. This flexibility ensures that the reference genome can be customized according to the user's specific use case and dataset requirements.

"an AIDA autotransporter-like protein, only found in Enterica strain samples but not in samples spiked with Houtenae or Salamae strains." All these strains are of the

subspecies enterica

> Thank you for your comment. We have revised the sentence to clarify that all the mentioned strains belong to Salmonella enterica subsp. enterica, and the distinction was meant to highlight the presence of the AIDA autotransporter-like protein specifically in strains from Enterica (such as Typhimurium), but not in those spiked with Houtenae or Salamae strains. This revision has been applied throughout the manuscript for consistency and clarity.

Figure 3: punctuations a bit off here and there.

> The punctuation in the Figure 3 caption has been reviewed and corrected for clarity and consistency.

Why do you operate with cfu/ml? You added it to meat? It should be cfu/g?

> Meat was used as a classical background matrix, aligning with typical sample types analysed under ISO 6579 standards (products intended for human consumption). This method adheres to various European regulations, such as Regulation (EC) No 2160/2003 of the European Parliament and of the Council of 17 November 2003 on the control of Salmonella and other specified foodborne zoonotic agents, as well as Commission Regulation (EC) No 1688/2005 of 14 October 2005. The latter implements Regulation (EC) No 853/2004, providing special guarantees concerning Salmonella for consignments of certain meat and eggs to Finland and Sweden (Text with EEA relevance). The Regulation(EC) No 2160/2003 of the European Parliament e.g. says clearly: As regards poultry meat, the aim is to place on the market poultry meat with reasonable assurance that it is free from relevant salmonella. Similar statements can also be found in other European regulations.
> The concentration of Salmonella was determined in cfu/ml, as this refers to the resuspension of the centrifuged pellet in the enrichment broth. We understand the importance of reporting pathogen concentrations in cfu/g when working directly with meat, but in this case, the results were based on enrichment broth after resuspension."

It would be nice with a presentation of the resistance panel of the three spiked strains before presenting the amr genes.

> Thank you for your suggestion. The resistance profiles associated with the identified AMR genes are generated by our workflow and are available as part of the analysis outputs. These results, presented as a comprehensive table, are accessible via the output Galaxy history, which is publicly available on the PathoGFAIR GitHub repository (entry number 15291, https://usegalaxy.eu/api/datasets/4838ba20a6d86765e25ff74fab63aeed/display?to_ext=tabular).

"Similar but inverse relations are observed for CFU/mL value (Figure 3 C & D), with a threshold for VF and AMR gene detection at 106 ." cfu/ml of what? The rinse? Added ml? I don't even know how much meat were included in the dna extractions.

> The colony counts refer to the concentration of Salmonella per milliliter (cfu/ml) in the rinse solution after the enrichment process, which involves resuspending the centrifuged pellet from the enrichment broth. These values represent the microbial concentration in the liquid phase after enrichment, not the original concentration in the meat matrix. Regarding the amount of meat used in DNA extractions, approximately 25 mg of meat was included per aliquot for extraction.
> We have clarified this in the manuscript, specifying that the CFU/mL values refer to the bacterial concentration in the rinse solution and the amount of meat used for the DNA extraction process.

"The further the samples are from these thresholds, the higher the number of VF genes and AMR genes identified. Indeed, the three top scattered dots with identified VF genes between 250 and 300 (Figure 3 A, C, E) are the samples with the highest number of reads, higher CFU/mL value, and a relatively lower Ct value compared to other samples." The tendency is ok, but not all. For instance, you have several exceptions here for both amr genes and vf genes. Maybe mark the dots after say

| | |
|---|---|
| | spiked strain/enrichment or not?<br><br>> We acknowledge that while the overall tendency shows a correlation between higher CFU/mL and lower Ct values with the number of identified VF and AMR genes, there are except... |
| **Additional Information:** | |
| **Question** | **Response** |
| Are you submitting this manuscript to a special series or article collection? | No |
| **Experimental design and statistics**<br><br>Full details of the experimental design and statistical methods used should be given in the Methods section, as detailed in our Minimum Standards Reporting Checklist. Information essential to interpreting the data presented should be made available in the figure legends.<br><br>Have you included all the information requested in your manuscript? | Yes |
| **Resources**<br><br>A description of all resources used, including antibodies, cell lines, animals and software tools, with enough information to allow them to be uniquely identified, should be included in the Methods section. Authors are strongly encouraged to cite Research Resource Identifiers (RRIDs) for antibodies, model organisms and tools, where possible.<br><br>Have you included the information requested as detailed in our Minimum Standards Reporting Checklist? | Yes |
| **Availability of data and materials**<br><br>All datasets and code on which the conclusions of the paper rely must be either included in your submission or deposited in publicly available repositories (where available and ethically appropriate), referencing such data using | Yes |

| | |
|---|---|
| a unique identifier in the references and in the “Availability of Data and Materials” section of your manuscript.<br><br>Have you have met the above requirement as detailed in our Minimum Standards Reporting Checklist? | |

Powered by Editorial Manager® and ProduXion Manager® from Aries Systems Corporation

LaTeX - Main Document (TeX file) Click here to access/download;LaTeX - Main Document (TeX file);main.tex

Placeholder for OUP logo oup.pdf

*GigaScience*, 2023, 1–15

doi: xx.xxxx/xxxx
Manuscript in Preparation
Technical Note

TECHNICAL NOTE

# PathoGFAIR: a collection of FAIR and adaptable (meta)genomics workflows for (foodborne) pathogens detection and tracking

Engy Nasr[1], Anna Henger[2], Björn Grüning[1], Paul Zierep[1] and Bérénice Batut[3,4,*]

[1]Bioinformatics Group, Department of Computer Science, University of Freiburg, Georges-Koehler-Allee 106, 79110 Freiburg im Breisgau, Germany and [2]Biolytix AG, 4243 Dittingen, Switzerland and [3]CNRS, Institut Français de Bioinformatique, IFB-Core, UAR 3601, 91000 Evry, France and [4]Plateforme AuBi, Mésocentre Clermont-Auvergne, Université Clermont Auvergne, 63170 Aubière, France

[*]berenice.batut@gmail.com

## Abstract

**Background:** Food contamination by pathogens poses a global health threat, affecting an estimated 600 million people annually. During a foodborne outbreak investigation, microbiological analysis of food vehicles detects responsible pathogens and traces contamination sources. Metagenomic approaches offer a comprehensive view of the genomic composition of microbial communities, facilitating the detection of potential pathogens in samples. Combined with sequencing techniques like Oxford Nanopore sequencing, such metagenomic approaches become faster and easier to apply. A key limitation of these approaches is the lack of accessible, easy-to-use, and openly available pipelines for pathogen identification and tracking from (meta)genomic data. **Findings:** PathoGFAIR is a collection of Galaxy-based FAIR workflows employing state-of-the-art tools to detect and track pathogens from metagenomic Nanopore sequencing. Although initially developed to detect pathogens in food datasets, the workflows can be applied to other metagenomic Nanopore pathogenic data. PathoGFAIR incorporates visualisations and reports for comprehensive results. We tested PathoGFAIR on 130 samples containing different pathogens from multiple hosts under various experimental conditions. For all but one sample, workflows have successfully detected expected pathogens at least at the species rank. Further taxonomic ranks are detected for samples with sufficiently high Colony-forming unit (CFU) and low Cycle Threshold (Ct) values. **Conclusions:** PathoGFAIR detects the pathogens at species and subspecies taxonomic ranks in all but one tested sample, regardless of whether the pathogen is isolated or the sample is incubated before sequencing. Importantly, PathoGFAIR is easy to use and can be straightforwardly adapted and extended for other types of analysis and sequencing techniques, making it usable in various pathogen detection scenarios.

**Key words**: Galaxy; Public health; Nanopore; Pipeline; Open source; Benchmark samples; Visualisation

## Introduction

Foodborne pathogens pose a significant threat to public health worldwide, causing millions of cases of illness and even death every year [1, 2]. These diverse microorganisms, spanning bacteria, viruses, parasites, and fungi, can contaminate a variety of foods, leading to both localised outbreaks and widespread epidemics. Ensuring food safety and controlling foodborne pathogens are key priorities for public health authorities at local, regional, and global levels, including agencies such as the European Food Safety Authority (EFSA), European Centre for Disease Prevention and Control (ECDC), and the World Health Organisation (WHO) [3].

Traditional methods for identifying the source of food contamination require isolation of the target pathogen. This process is not only time-consuming but can be labor-intensive, often requiring multiple steps and sophisticated techniques, and lacks a guaranteed

**Compiled on:** December 5, 2024.
Draft manuscript prepared by the author.

success rate [4]. In contrast, shotgun metagenomic approaches provide a solution to these challenges, as they give an overview of the genomic composition in the sample, including the food source itself, the microbial community, and any possible pathogens and their complete genetic information [5]. Importantly, shotgun metagenomic approaches eliminate the need for prior isolation of the targeted pathogen, as required by Whole Genome Sequencing (WGS) methods, and they are not limited to specific genes as opposed to real-time PCR approaches [6] or 16S rRNA sequencing . While 16S rRNA sequencing is widely used for bacterial taxonomic profiling, it is limited in scope compared to shotgun metagenomic sequencing. The latter allows for the detection of a wide range of pathogens, including bacteria, viruses, and fungi, and gives access to the full genomes enabling the taxa-agnostic identification of antimicrobial resistance (AMR) and virulence genes. This broader scope makes shotgun sequencing more suitable for comprehensive pathogen detection, especially in complex foodborne outbreak investigations [7].

Nanopore sequencing provides long-read data that can capture comprehensive genetic information. Its utilisation, as exemplified by studies like [8], demonstrates its utility in closing genomic gaps, delivering real-time sequencing data, and enhancing the capabilities of metagenomic approaches for outbreak investigations. This technology enables more accurate and rapid pathogen detection, a critical advancement in scenarios where timely responses are essential for effective outbreak management.

Once sequencing data is generated, it must be processed using bioinformatics tools to identify pathogens, their genetic variations, and Virulence Factor (VF) genes, thereby facilitating timely and accurate detection [9, 10]. However, available tools and workflows require bioinformatic and computational knowledge and expertise. For example, tool parameters need to be adapted to the specific use case. End-to-end platforms (Table 1) that allow users to analyse their samples are either restricted with only a limited free trial (e.g. BugSeq [11]) or paid subscription (e.g. OneCodex [12]), or require high computational resources (e.g. SURPI [13] and Sunbeam [14]). For certain free resources, the underlying workflow is not available and adaptable for the user. For example, IDseq [15] (also known as CZID [16]), a free cloud-based service for pathogen detection can only be externally accessed through the dedicated online user interface. Furthermore, some of these workflows are specific to a certain host, pathogen, or sequencing technique, lacking the flexibility for customisation.

Galaxy [17] is an open-source platform for FAIR data analysis. It enables users to apply a comprehensive suite of bioinformatics tools (that can be combined into workflows) through either its user-friendly web interface or its automatable Application Programming Interface (API) for integrating and customising workflows, enhancing user flexibility. It ensures reproducibility by capturing the necessary information to repeat and understand data analyses. Galaxy offers a collection of high-quality pre-built workflows that can be either used directly or are easily adapted to the user's needs via the Galaxy workflow editor. Galaxy workflows can be executed on any Galaxy server, even on the private Galaxy server, making it suitable also for data where privacy concerns are important. Furthermore, Galaxy via the major public servers [17] freely provides a large computing infrastructure allowing for the execution of computationally challenging workflows, which is often the case for metagenomic analysis.

Here, we present PathoGFAIR, a collection of Galaxy-based workflows for pathogen identification and tracking its presence among (meta)genomics Oxford Nanopore sequencing data. The workflows are openly available on two workflow registries (Dockstore [18] and WorkflowHub [19]). They can be used directly on three major Galaxy servers (usegalaxy.org, usegalaxy.eu, usegalaxy.org.au) or installed in any other Galaxy server. The workflows are created to work agnostically, detecting all pathogens present in the samples without prior knowledge of the target pathogen. As the workflows are created in Galaxy, they can be adapted, e.g. for other sequencing techniques or with various downstream analyses, such as differential expression analysis, or further statistics and visualisations [17]. Workflows are documented and supported by an extensive tutorial freely available via the Galaxy Training Network (GTN) [20]. Overall, PathoGFAIR offers an easy-to-use computational solution that speeds up the process of sampling, detecting, and tracking pathogens. Links to workflows and tutorials can be found on PathoGFAIR homepage: https://usegalaxy-eu.github.io/PathoGFAIR/

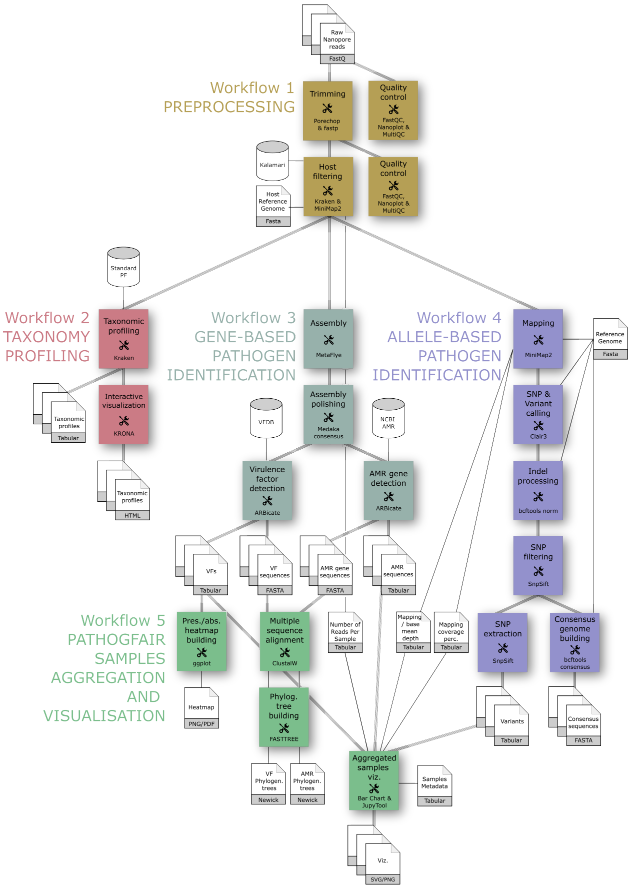

**Figure 1.** Flowchart of the PathoGFAIR workflows. Workflow 1 (olive green) takes as input sequencing data generated by Oxford Nanopore technologies and performs quality control and host filtering. Then three parallel workflows are executed on the output of Workflow 1: Workflow 2 (red) for taxonomy profiling, Workflow 3 (dark cyan) for gene-based pathogen identification, and Workflow 4 (purple) for SNP-based pathogen identification. These four workflows can run individually and in parallel. Finally, all outputs for the different provided datasets are aggregated in Workflow 5 (green) for PathoGFAIR Samples Aggregation and Visualisation.

## Implementation

### Overview

PathoGFAIR comprises a collection of 5 workflows, implemented in Galaxy (Figure 1). Each workflow serves a specific function and can be executed independently, enabling users to tailor their analysis according to their requirements.

The input data for PathoGFAIR comprises sequencing data generated using Oxford Nanopore technologies, along with an optional metadata table describing the datasets. Basecalling for converting raw signal data from the Nanopore sequencer into nucleotide sequences is not included within the PathoGFAIR workflows. In the use cases presented later in the manuscript, real-time basecalling is performed using the MinKNOW software (Oxford Nanopore Tech-

nologies) before the reads are used in the workflows. Basecalling is a crucial step, as it affects the quality of the reads. Users are encouraged to ensure that high-quality basecalling is performed before starting the analysis with PathoGFAIR.

The datasets are preprocessed in Workflow 1, which encompasses quality control and host removal procedures. Subsequently, the preprocessed data is directed to three parallel workflows: taxonomy profiling (Workflow 2), gene-based pathogen identification (Workflow 3), and allele-based pathogen identification (Workflow 4). This parallel execution allows for efficient analysis and flexibility in workflow selection. Notably, Workflow 4 can optionally synchronise with Workflow 2 or Workflow 3 to leverage prior taxonomic analysis or gene-based pathogen identification results, providing users with flexibility based on specific use cases. By using detailed taxonomic identification from Workflow 2 or gene-based pathogen identification from Workflow 3, Workflow 4 enhances mapping and SNP detection accuracy. This process involves selecting the correct reference genome of the pathogen for mapping, informed by results from Workflow 2, Workflow 3, or even Workflow 1, which performs initial taxonomy assignment during the host filtering step.

Since each workflow can be executed independently, users can focus on specific aspects of pathogen detection or analysis. This modular approach empowers users to utilise the full range of functions offered by each workflow individually or to combine them as needed for comprehensive pathogen detection.

Finally, in Workflow 5, outputs from the previous workflows and the metadata of the dataset are aggregated and visualised for comprehensive pathogen tracking across samples. This aggregation step ensures a holistic view of pathogen presence and distribution, facilitating further insights and analysis.

Overall, the independent nature of PathoGFAIR's workflows provides users with a user-friendly and customisable approach to pathogen detection, allowing for both comprehensive analyses and targeted investigations based on specific research needs or objectives.

Ensuring the accuracy and currency of reference data is indeed fundamental for robust metagenomic analysis. PathoGFAIR leverages Galaxy's integrated Data Managers, which enables Galaxy admins to provide up-to-date reference data. These Data Managers automate the download, installation, and regular update of essential reference databases, ensuring that PathoGFAIR users work with complete, accurate, and up-to-date reference information. PathoGFAIR workflows are configured to use well-maintained and reputable sources, such as NCBI and other public pathogen reference repositories, which further support accuracy and comprehensiveness. Additionally, Galaxy's user-friendly interface enables users to select preferred references or request the inclusion of specific databases via Galaxy administrators, adding to the workflow's adaptability for diverse use cases.

PathoGFAIR offers a competitive, and accessible solution (Table 1) to detect and track pathogens in metagenomic Nanopore data through its five Galaxy-based FAIR and customisable workflows.

## Workflow 1: Preprocessing

Workflow 1 encompasses essential preprocessing steps to ensure the quality and integrity of sequencing data.

Quality control and sequence filtering, based on quality, length, or low complexity, are performed using Fastp (v 0.23.2) [24]. Porechop (v 0.2.4) [25] trims low-quality base pairs and removes duplicates and adapters. Quality thresholds are set to ensure that reads have an average quality score of Q20, aligning with accepted standards for Nanopore sequencing, where Q20 or higher quality is typically sufficient for reliable results [26].

Quality-controlled (QC) reads are cleaned of sequences from the host or food source (e.g. bovine in case of bovine meat) by mapping to their reference genome using Minimap2 (v 2.26) (RRID:SCR_018550) [27], a tool tens of times faster than mainstream long-read mappers such as BLASR [28], BWA-MEM [29], NGMLR [30] and GMAP [31] and three times as fast as Bowtie2 [32] designed for Illumina short reads [27]. A variety of reference genomes (e.g. Human, Chicken, or Cow) can be installed on Galaxy servers to work with Minimap2. A wide variety of reference genomes are integrated into Minimap2 on Galaxy, providing users with a convenient selection to choose from before executing the workflow. Kraken2 (v 1.2) [33] is applied for further contamination detection e.g. human sequences using the Kalamari database. The Kalamari database includes mitochondrial sequences of various known hosts [34]. Host/food source reads matched to the Kalamari database are assessed and removed using Krakentools (v 1.2) [33].

The workflow returns QC reads without contamination or host sequences as well as interactive reports, produced by FastQC (v 0.12.1) (RRID:SCR_014583), fastp and MultiQC (v 1.11) (RRID:SCR_014982) [35]. Furthermore, Nanoplot (v 1.39.0) [36] is employed to provide detailed quality metrics specifically tailored to the preprocessing step, enriching the suite of analytical insights and facilitating robust data evaluation.

## Workflow 2: Taxonomy Profiling

Workflow 2 performs taxonomic profiling of the microbial community to identify pathogens and other microorganisms for the QC reads from Workflow 1, using Kraken2 (v 1.2) [33] and the PlusPF (archaea, bacteria, viral, plasmid, human, UniVec_Core, protozoa, fungi, and plant) Refseq database (June 7, 2022). Although Kraken2 is a tool designed for short-read sequencing and is known for its false positive taxonomy assignments, particularly at lower microbial abundances [37], its application to long-reads can still yield a substantial overview of the microbial community. This is particularly true for discerning bacteria that could potentially be pathogenic at genus and species taxonomic ranks [38, 39]. Kraken2 allows for the rapid assignment of taxonomy at multiple ranks, from kingdom to species, using an efficient exact k-mer matching algorithm. Other tools such as Centrifuge (RRID:SCR_016665) or MetaPhlAn (RRID:SCR_004915) are viable alternatives, also available on Galaxy. Kraken2 is selected for its speed, sensitivity, and ability to work with large reference databases, a critical factor when analysing complex metagenomic samples [33, 40]. The produced community profile is visualised using Krona (RRID:SCR_012785) [41] and observed interactively for different taxonomic ranks using Phinch [42] or Pavian [43].

## Workflow 3: Gene-based Pathogen Identification

In this workflow, the pathogens are identified by the presence of genes associated with pathogenicity. QC reads from Workflow 1 are assembled into contigs using Metaflye (v 2.9.1) (RRID:SCR_017016) [44]. The contigs are then polished using the Medaka Consensus Pipeline (v 1.7.2) [45], which generates consensus sequences using neural networks and shows improved accuracy over graph-based approaches for Oxford Nanopore reads. The polished contigs are afterwards screened using ABRicate (v 1.0.1) [46] for virulence factors (VF) with the Virulence Factor DataBase (VFDB) [47] and for antimicrobial resistance (AMR) genes with AMRFinderPlus [48] database. ABRicate is chosen for its versatility, as it supports multiple databases, including those for antimicrobial resistance genes and the Virulence Factor Database (VFDB). This makes it a comprehensive tool for gene-based pathogen detection, capable of identifying a wide range of relevant genetic markers [46].

**Table 1.** Comparison of features between PathoGFAIR and other similar pipelines or systems. This comparison sheds light on various features and characteristics, such as accessibility, technical specifications, and the scope of analyses offered by each system. It serves as a reference to evaluate the suitability of PathoGFAIR and other similar pipelines or systems for specific needs and requirements

| **Features** | **PathoGFAIR** | **IDseq** | **BugSeq** | **SURPI** | **OneCodex** | **Sunbeam** | **Innuendo** [21] | **PAIPline** [22] | **Victors** [23] |
|---|---|---|---|---|---|---|---|---|---|
| **General Characteristics** | | | | | | | | | |
| Free of Charge | ✓ | ✓ | ✗* | ✓ | ✗ | ✓ | ✓ | ✓ | ✓ |
| Open Source Code | ✓ | ✓ | ✗ | ✓ | ✗ | ✓ | ✓ | ✓ | ✗ |
| Web Interface | ✓ | ✓ | ✓ | ✗ | ✓ | ✗ | ✓ | ✗ | ✓** |
| Automatable API | ✓ | ✗ | ✗ | ✗ | ✗ | ✓ | ✓ | ✗ | ✗ |
| **Accessibility and Availability** | | | | | | | | | |
| Simple end-user Modification | ✓ | ✗ | ✗ | ✗ | ✗ | ✓ | ✗ | ✓ | ✗ |
| Publicly Available Web-server | ✓ | ✓ | ✓ | ✗ | ✓ | ✗ | ✗ | ✗ | ✓ |
| Last Updated | 2024 | 2024 | 2024 | 2014 | 2023 | 2024 | 2018 | 2018 | 2019 |
| **User Support and Documentation** | | | | | | | | | |
| Tutorial | ✓ | ✗ | ✗ | ✗ | ✗ | ✗ | ✗ | ✗ | ✗ |
| Documentation | ✓ | ✓ | ✓ | ✓ | ✓ | ✓ | ✓ | ✓ | ✓ |
| User support | ✓ | ✓ | ✓ | ✗ | ✓ | ✗ | ✗ | ✗ | ✗ |
| **Technical Specifications** | | | | | | | | | |
| Workflow Manager | Galaxy | - | - | - | - | Snakemake | Nextflow | - | - |
| Sequencing Technique | Nanopore*** | Illumina & Nanopore | Illumina & Nanopore | Illumina | - | Illumina | Illumina | Illumina | - |
| **Analyses** | | | | | | | | | |
| Preprocessing | ✓ | ✓ | ✓ | ✓ | ✓ | ✓ | ✗ | ✓ | ✗ |
| Taxonomy Profiling | ✓ | ✓ | ✓ | ✓ | ✓ | ✓ | ✗ | ✓ | ✗ |
| Gene-based Pathogen Identification | ✓ | ✓ | ✓ | ✓ | ✓ | ✓ | ✓ | ✓ | ✓ |
| Allele-based Pathogen Identification | ✓ | ✗ | ✓ | ✗ | ✗ | ✗ | ✓ | ✗ | ✓ |
| Samples aggregation and Visualisations | ✓ | ✓ | ✗ | ✗ | ✓ | ✓ | ✗ | ✗ | ✗ |

* Free trial of 10 samples is available.
** Malfunctioned when tested.
*** Can be easily adapted to any other types of sequencing techniques via Galaxy, a customisable and automatable API.

## Workflow 4: Allele-based Pathogen Identification

Another approach to identifying pathogens is to use an allelic approach by detecting SNPs, i.e. markers showing evolutionary histories of homogeneous strains [49]. This process includes SNP calling, aimed at identifying novel pathogen strains and elucidating discrepancies compared to reference sequences, thereby facilitating the tracing of emerging variants. Within Workflow 4, both complex variants and SNPs are discerned, serving as crucial elements for subsequent pathogen identification and variant tracing purposes.

QC reads from Workflow 1 are mapped using Minimap2 (v 2.26) to a selected reference genome of a suspected pathogen. Users can choose the reference genome based on their prior knowledge of the target pathogen, the taxonomic analysis in Workflow 2, or the detected pathogenic genes in Workflow 3. Variant calling for mapped reads is performed using Clair3 (v 0.1.12) [50]. Clair3, a tool developed for long reads, has been chosen because it is demonstrated to be faster and more accurate than the Medaka variant pipeline, which its developer has declared deprecated in favour of Clair3 [45]. After that, all complex variants and their information, such as type, genomics position, and quality score, are normalised using bcftools norm (v 1.9) [51]. The normalised reads are filtered using SnpSift filter (v 4.3) (RRID:SCR_015624) [52] based on the SNP quality computed in the SNPs identification step with Clair3. Filtered variants fields required for further analyses are extracted using SnpSift extract fields (v 4.3) (RRID:SCR_015624) [52]. Finally, a consensus sequence for each sample is built using bcftools consensus (v 1.9) (RRID:SCR_005227) [53]. In addition to the variants, this workflow outputs tables including summary metrics like the mapping coverage (breadth of coverage) percentages for every sample, per base covering mean depth (depth of covering), and quality filtered complex variants and SNPs numbers. For more accurate results, users should consider only SNPs with a minimum depth of covering of 10x to ensure reliable calls, as demonstrated in the analyses of the following Use Cases section. This threshold effectively minimises the inclusion of false-positive variants, a challenge often encountered with Nanopore sequencing data due to its inherent error rates.

## Workflow 5: PathoGFAIR Samples Aggregation and Visualisation

In all previously described workflows, individual samples are analysed separately. Workflow 5 consolidates the outputs from Workflows 1, 2, 3, and 4 along with sample metadata to generate various visualisations and reports. These reports illustrate the detected pathogens and facilitate the visualisation and tracking of their presence across all samples.

Virulence Factor (VF) tables from Workflow 3 are used to generate clustered heatmaps showing the VF genes using ggplot2 Heatmap (v 3.4.0) (RRID:SCR_014601). VF sequences are concatenated per sample, generating a consensus sequence of identified VF genes per sample, and aligned over all samples using ClustalW (v 2.1)(RRID:SCR_017277). A phylogenetic tree of the virulence gene sequences is then generated from the multiple sequence alignment using FASTTREE (v 2.1.10) (RRID:SCR_015501) [54] and visualised using Newick Display (v 1.6). The same is performed on the antimicrobial resistance (AMR) tables from Workflow 3. From Workflows 1 and 4 output tables, bar charts are generated.

Other outputs are aggregated and processed within a Jupyter Notebook [55], interactively launched in Galaxy using JupyTool (v 1.0.0). This Notebook showcases the integration of sample metadata to generate analysis-specific plots, leveraging Python (v 3.10.12) [56] libraries such as Pandas (v 1.5.3) [57, 58], Matplotlib (v 3.7.1) [59], Seaborn (v 0.12.2) [60], and Numpy (v 1.24.3) [61]. Examples of these plots include bar plots illustrating the number of reads before and after quality control for all samples, scatter plots visualising relationships between different variables such as pathogen count and sample characteristics, and interactive cluster maps displaying the clustering patterns of samples based on pathogen composition. These visualisation techniques are further elucidated and exemplified in the Use Cases section of this study, where the output tables from the workflows are aggregated with the corresponding sample metadata and visualised to facilitate comprehensive visual analysis.

Virulence factors (VF) and antimicrobial resistance (AMR) genes are often found on mobile genetic elements (MGEs) such as plasmids or phages, meaning they can sometimes appear independently of their bacterial hosts. To address this challenge, PathoGFAIR integrates taxonomic profiling from Workflow 2 with gene detection results from Workflow 3. This cross-referencing ensures accurate attribution of VF and AMR genes to their respective host organisms. For further validation, Workflow 4 enables users to map consensus genomes, generated in Workflow 5 from detected VF genes, against any reference genomes. This process confirms whether these VF or AMR genes, detected in Workflow 3, are genuinely linked to the bacterial genome or merely associated with MGEs, with additional coverage metrics helping to ensure accurate mapping. Future updates to PathoGFAIR will include expanded methodologies to validate gene-host associations using broader taxonomic markers, further refining the precision of pathogen characterisation..

## Workflow Reports

As all PathoGFAIR workflows are designed to run seamlessly on the Galaxy platform, an interactive report is automatically generated upon completion of each workflow. These reports provide a comprehensive overview of the respective workflow's inputs and outputs. In PathoGFAIR, special attention has been given to refining these reports for enhanced user experience. The reports are carefully curated to automatically showcase and emphasise only the most informative, easily interpretable, and accessible outputs for each workflow. This ensures that users can efficiently extract key insights from the results, facilitating a streamlined and user-friendly analysis experience.

## Easily Adaptable Workflows

The workflows can process raw shotgun (meta)genomics sequencing data from any sample, not only food.

PathoGFAIR has been initially developed to take Oxford Nanopore data as inputs. However, PathoGFAIR can work with Illumina data or other types of sequencing technique data. To adapt to Illumina sequencing only one tool needs to be changed in Workflow 1: Porechop [25] with Cutadapt (RRID:SCR_011841) [62]. Workflows 2, 3, 4, and 5 can be used directly with Illumina datasets without any adaptation. Some tools can be changed based on the tool's known performance towards short and long reads, such as Clair3 (v 0.1.12) [50] and Metaflye (v 2.9.1) [44]. All the mentioned tools are accessible within Galaxy, allowing for seamless interchangeability.

The workflows can also be adapted to process paired-end reads, by adjusting the tools' parameters to take paired-end read samples instead of single-end reads. These changes can be applied with little effort by using the user-friendly workflow editor in Galaxy.

Users can seamlessly switch between different host reference genomes and Kraken2 databases, as PathoGFAIR supports various pre-installed databases on the Galaxy servers. This feature enhances user convenience and efficiently explores different configurations to suit specific analysis requirements.

Similarly, tool versions and parameters can be adapted, e.g. to compare results with legacy versions of the workflows. New tool versions are automatically installed on public Galaxy servers using a sophisticated update infrastructure, ensuring a straightforward mechanism to keep the infrastructure up-to-date [63]. Every time

a tool is updated, an update of the workflows is suggested, tested with functional tests, and released on the workflow registries once accepted.

Each of the five PathoGFAIR workflows is designed for a distinct type of analysis. Workflows 2, 3, and 4 operate independently, offering the flexibility to run them concurrently or skip them as per user requirements. This modular structure allows users to tailor the analysis to their specific needs, activating only the functionalities necessary for the desired workflow outcome.

## FAIR Workflows

The FAIR principles [64], which emphasise the importance of making research objects Findable, Accessible, Interoperable, and Reusable, offer valuable guidance for optimising the utility and promoting the reproducibility and reusability of any research object (data, software [65], or workflows).

PathoGFAIR has been developed with the FAIR principle in mind and follows the ten tips for building FAIR workflows, as suggested by de Visser *et al.* [66]. First, by using Galaxy as a workflow manager, the workflows are portable (Tip 6) and come with a reproducible computational environment (Tip 7). The tools integrated into the workflows use file format standards such as FASTA and FASTQ for sequence data, SAM and BAM from the Samtools project for alignment data, VCF for genetic variations, GenBank and GFF3 for genomic annotations, and PDB for structural data (Tip 5) [64]. As explained in the previous section, the workflows are provided with default values (Tip 8) and are modular (Tip 9).

The workflows are available on the GitHub repository of IWC, the Intergalactic Workflow Commission of the Galaxy community (Tip 3) [67]. Workflows in this repository are reviewed and tested using test data before publication and with every new Galaxy release. The IWC automatically updates the workflows whenever a new version of any tool used in these workflows is released. Deposited workflows follow best practices, are versioned using GitHub releases, and contain important metadata (e.g. License, Author, Institutes) (Tip 2). The workflows are automatically added to two workflow repositories (Dockstore [18] and WorkflowHub [19]) to facilitate the discovery and re-use of workflows in an accessible and interoperable way (Tip 1). Via Dockstore or WorkflowHub, the PathoGFAIR workflows can be installed on any up-to-date Galaxy server. They are already publicly available on three main Galaxy servers (usegalaxy.org, usegalaxy.eu, usegalaxy.org.au), which any user can use and modify without restriction.

A thorough explanation of how to use the workflows in PathoGFAIR including a more global description of pathogen identification from Oxford Nanopore data can be found in a dedicated extensive tutorial [68] together with example input data and results (Tips 4 and 10), freely available and hosted via the Galaxy Training Network (GTN) [20] infrastructure.

Finally, for every invocation of the workflows, a Research Object Crate (RO-Crate [69, 70]) can be created to store the data products of the different steps, along with the run-associated metadata (including parameters, tool, and workflow version).

# Use Cases

To showcase PathoGFAIR and its capabilities, 130 samples from two distinct studies—one involving samples with prior pathogen isolation and the other without— were analysed. In the case of non-isolated samples, pathogens were deliberately spiked to mimic real-world scenarios. For isolated samples, prior identification ensured the pathogens' identities were known. All samples underwent sequencing using Oxford Nanopore technology, highlighting the workflow's adaptability across diverse sample preparation methods.All workflows of PathoGFAIR were evaluated for their main intended tasks, e.g., the preprocessing workflow for its reads quality retaining and hosts sequences removal performance, but also for their ability to identify the correct pathogen, and how well the accuracy with respect to different sampling conditions is.

## Samples Without Prior Pathogen Isolation

### *Data Generation*

In this study, 46 samples had been prepared given the following protocol [71]. Chicken meat was spiked with either one of three *Salmonella enterica* subspecies (*S. enterica* subsp. *houtenae* DSM 9221, *S. enterica* subsp. *enterica* DSM 554, or *S. enterica* subsp. *salamae* DSM 9220) or a mix of them, with concentrations that give Cycle Threshold (Ct) values between 25 and 33. A total of 15 samples were incubated at 37°C for 24 hours before DNA isolation to facilitate bacterial growth. All samples were after incubated at 56°C for 1 hour with lysis buffer and 20 $ng/\mu l$ Proteinase K, followed by DNA extraction according to the STAR BEADS Pathogen DNA/RNA Extraction kit (CYANAGEN SRL, Bologna, Italy) instructions. In this study, approximately 25 mg of meat were used per aliquot for DNA extraction. DNA concentrations were measured with the Qubit® 4.0 Fluorometer (Thermo Fisher Scientific) using the double-stranded DNA (dsDNA) High-Sensitivity (HS) assay kit (Thermo Fisher Scientific), following the manufacturer's protocol. The quality was evaluated with a Nanodrop® 1000 (Thermo Fisher Scientific), assessing the 260/280 nm and 260/230 nm ratios. 260/280 and 260/230 ratios were close to the expected ranges 1.8–2.0 and 2.0–2.2, respectively. Extracted DNA was barcoded before sequencing using the Native barcoding genomic DNA (with EXP-NBD104, EXP-NBD114, and SQK-LSK109) protocol (Oxford Nanopore). DNA was then loaded on an R9.4.1 MinION Mk flow cell (Oxford Nanopore). SpotON sample port cover and priming port were closed and sequencing was started. The sequencing device control, data acquisition, and real-time basecalling were carried out by the MinKNOW software of the MinION Mk1C device. For 6 samples, adaptive sampling, a technique used in Nanopore sequencing to selectively sequence microbial DNA while excluding unwanted host DNA (here chicken DNA), was used. Generated sequencing data is available via BioProject PRJNA982679. Metadata for the 46 samples is summarised in Supplementary Table T1 into five pieces of information: (i) expected subspecie(s), (ii) incubation before DNA isolation, (iii) adaptive sampling during sequencing, (iv) Colony-forming unit (CFU)/mL [72], a measure providing a quantitative assessment of viable microbial entities within a given sample and measured using standard microbiological techniques such as serial dilution and plating on agar medium, provides, (v) Cycle Threshold (Ct) values [73], values inversely proportional to the amount of nucleic acid present in the samples.

### *Preprocessing*

The number of reads after quality control varies significantly between samples (Figure 2 A), which impacts downstream analyses.

For host detection using Minimap2 (v 2.26), the option *PacBio/Oxford Nanopore read to reference mapping* was set here. As expected from the samples sequencing protocol (chicken samples and not isolated pathogen), most sequences were assigned to chicken (Gallus gallus galGal6): above 90% in 31 samples and between 55% to 85% for the remaining 15 samples (Supplementary Figure S1). However, the percentage of identified host DNA (between 60% and 98%) was not as low as expected for the 6 samples that had undergone adaptive sampling to exclude chicken DNA during sequencing. This shows that the adaptive sampling to exclude chicken in some samples during sequencing may not had removed all the chicken sequences. All sequences identified as chicken were removed (Figure 2 A). After QC and host removal, 19 samples had less than 1,000 reads. These samples could only be analysed using the taxonomy profiling as highlighted in the next sections.

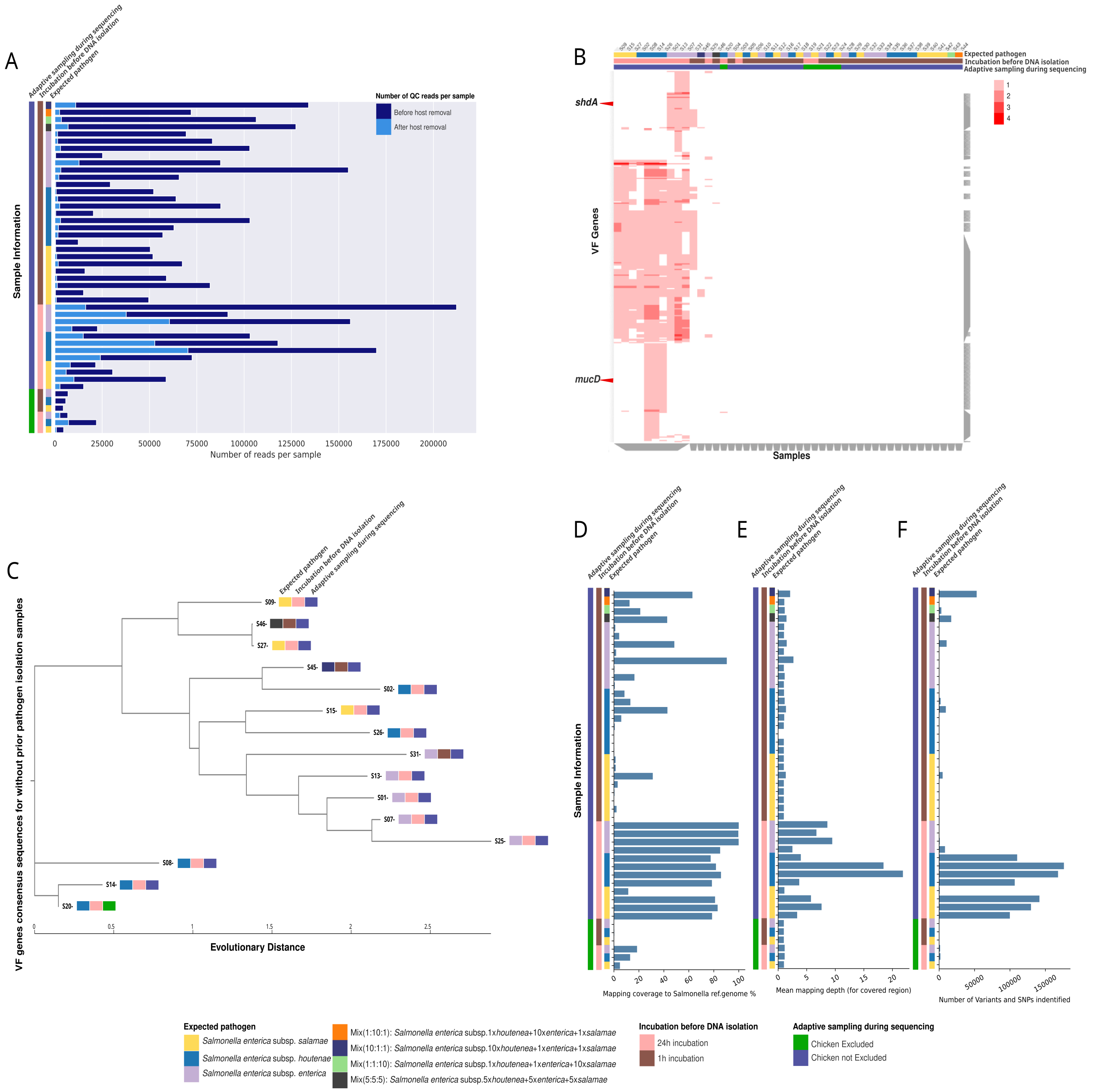


**Figure 2.** (**A**) Bar plot showing the total number of quality-controlled reads per sample before (dark blue) and after (light blue) host sequences removal. On the left, the metadata of the samples are displayed: (i) the expected *S. enterica* subsp. *salamae* in yellow, *S. enterica* subsp. *houtenae* in blue, and *S. enterica* subsp. *enterica* in light purple), (ii) incubation before DNA isolation (incubated for 24h in pink and incubated for 1h in brown), and (iii) adaptive sampling during sequencing (chicken excluded in green and chicken not excluded in purple)(**B**) Clustergram displaying the identified VF genes' abundances per sample. The VF genes are presented on the y-axis and all 46 non-isolated samples are on the x-axis along with their sample information. On the top are the metadata of the samples with the same color code as in **A** The grey bars on the bottom and on the right represent dendrogram VF gene (right) and samples' metadata (bottom) clusters found with hierarchical clustering with a clustering granularity of 0.5. (**C**) Phylogenetic tree, using the nucleotide evolution model; General Time Reversible (GTR) model with a CAT approximation for rate heterogeneity across sites [54]. The Phylogenetic tree was built on the VF genes consensus sequences concatenated per sample and aligned for all samples. (**D**) Bar plot with the mapping coverage (breadth of coverage), i.e. the percentage of covered bases of each sample to the reference genome, measured by calculating the percentage of positions within each bin with at least one base aligned against it. (**E**) Bar plot with the mean of the mapping depth (depth of coverage) of bases mapped to corresponding bases in the reference genome for every sample. (**F**) Bar plot with the number of variants and SNPs found per sample. Mapping coverage percentage and the depth mean indicate whether to trust the variants and SNPs found by the workflow or not, the higher the coverage percentage and the depth mean, the more trusted the SNPs results for the sample.

### Taxonomy Profiling

S. enterica was detected in Workflow 2 for all samples except one, at its species and different subspeciestaxonomic ranks (Interactive KRONA plot in Supplementary online Figure S2 and Supplementary Table T3).

### Gene-based Pathogen Identification

In Workflow 3, Metaflye (v 2.9.1) tool mode's option was chosen to be *Nanopore-HQ*, users can expand the workflow and change this option according to their datasets sequencing technique.

No contig was built for 10 of the 27 samples with less than 2,700 reads. The identification of Virulence Factors (VF) or AntiMicrobial Resistance (AMR) genes was then made impossible. For the other 17 samples, only 1 or 2 contigs were created, not enough for identifying VF and AMR genes.

For the remaining 19 samples with created contigs (from 3 to 157) and number of reads higher than 2,700, VF genes were identified in 15 samples (Figure 2 B), 12 of which were incubated before DNA isolation for 24 hours. 3 of the 15 samples were incubated for only 1 hour before DNA isolation, resulting in a few VF genes (Figure 2 B) identified, compared to the other 12 samples, mostly because of the low number of reads (Figure 3 E) from almost the absence of incubation (Figure 2 A). It was, for example, the case for the mixed samples, i.e. samples spiked with all 3 *S. enterica* subspecies, or samples spiked only with *S. enterica* subsp. *houtenae* and adaptively sampled during sequencing.

Some identified VF genes were found more than once in the same sample, with a maximum of 4 times. Common VF genes were identified for samples expecting identical *S. enterica* subspecies (Figure 2 B), such as the *mucD* gene, a serine protease *mucD* precursor, which was only found in *S. enterica* subsp. *houtenae* spiked samples, or *shdA*, an AIDA autotransporter-like protein, only found in *S. enterica* subsp. *enterica* spiked samples, but not in samples spiked with only *S. enterica* subsp. *houtenae* or *S. enterica* subsp. *salamae*.

Similar results were found for AMR genes (Supplementary Figure S3, 3 F). The sampling conditions affected the number of identified VF and AMR genes as shown by the relationships between the Ct value, CFU/mL value, or the number of remaining reads after preprocessing (Figure 3). The lower the Ct value, the higher the number of VF genes and AMR genes identified (Figure 3 A & B). No VF or AMR genes were detected for samples with Ct values above 26. For Ct values below 26, there was a negative correlation (Pearson $R = -0.85$, $p$-value $= 6 \times 10^{-05}$) between the Ct value and the number of identified AMR genes. Similar but inverse relations were observed for CFU/mL value (Figure 3 C & D), with a threshold for VF and AMR gene detection at $10^6$. VF and AMR genes were then detected if several conditions were fulfilled: a Ct value below 26, CFU/mL value above $10^6$, and at least 5,000 reads after preprocessing. The further the samples were from these thresholds, the higher the number of VF genes and AMR genes identified. Indeed, the three top scattered dots (in red - Figure 3 A, C & E), with identified VF genes between 250 and 300 were the samples with the highest number of reads, higher CFU/mL value, and a relatively lower Ct value compared to other samples. Generally, allowing samples to incubate for a short period before sequencing enhances microbial growth, resulting in higher CFU/mL values and lower Ct values. This increase in microbial concentration improves the efficiency of direct sequencing by providing more genetic material for analysis, facilitating faster and more accurate pathogen detection.

### Allele-based Pathogen Identification

In Workflow 4, samples were mapped against a reference genome of an expected pathogen chosen by the user. *S. enterica subsp. enterica* ser. Typhimurium (NC_003197.2) was chosen for this data, as it is widely recognised and extensively used in genomic studies due to its complete and well-annotated genome sequence [74]. However, given the diversity among the serovariants of *S. enterica* subsp. *enterica*, a high number of complex variants and SNPs are anticipated.

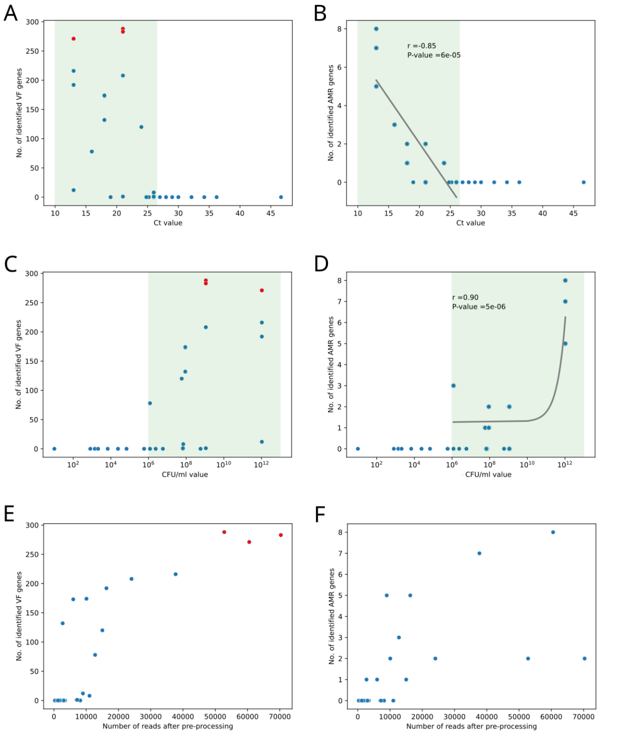


**Figure 3.** Scatterplots showing the number of identified VF genes (**A, C, E**) and AMR genes (**B, D, F**) in relationship to the Ct value (**A, B**), CFU/mL value (**C, D**), and the number of reads after preprocessing (**E, F**). The green area (**A, B, C, D**) highlights Ct values or CFU/mL values for which genes had been detected. Pearson correlation for values in the green area: (**A**) $R = -0.33$ and $p$-value $= 0.23$, (**B**) $R = -0.85$ and $p$-value $= 5.83e^{-05}$, (**C**) $R = 0.17$ and $p$-value $= 0.53$, (**D**) $R = 0.90$ and $p$-value $= 4.68e^{-06}$

The provided mapping statistics (mapping coverage (breadth of coverage) and mapping depth (depth of coverage) in Figure 2 D, E) serve as proxies for assessing the number and quality of identified SNPs (Figure 2 F). SNPs with low mapping depth are less reliable than those with higher depth. Reliable SNP calling typically requires a depth of at least 10, achieved in 2 samples. Samples with the highest mean mapping depth corresponded to samples with the highest number of reads after preprocessing (Figure 2 A). The higher the coverage and the mean mapping depth, the more quality SNPs were identified (Figure 2 D-F). Some of the samples spiked with *S. enterica* subsp. *enterica* had a high breadth of coverage but a low mean depth of coverage depth, as a result, the number of their quality filtered identified SNPs was low.

### PathoGFAIR Samples Aggregation and Visualisation

For the samples for which VF or AMR genes had been identified, phylogenetic trees were built on the concatenated genes consensus sequences (Figure 2 C for VF genes, Supplementary Figure S4 for AMR genes). These trees help track divergence between samples and could then highlight the contamination point or an evolution of the subspecies because of mutations. Indeed, samples spiked with *S. enterica* subsp. *enterica* were found together in the VFs-based tree (Figure 2 C), so the identified VF genes were unique to these samples and could clearly separate the samples from samples with other *S. enterica* subspecies. The samples spiked with *S. enterica* subsp. *houtenae* were mostly clustered together, except for 2 samples because of extra identified VF genes common with samples spiked with *S. enterica* subsp. *enterica* and/or *S. enterica* subsp. *salamae* . The 2 samples spiked with a mix of the 3 subspecies were found in the middle of the tree (Figure 2 C), showing that a mix

of VF genes related to the different subspecies was identified. The mixed sample, S45, spiked with a higher concentration of *S. enterica* subsp. *houtenae* than the other subspecies, was close to the sample, S02, spiked with *S. enterica* subsp. *houtenae* only. For AMR genes phylogenetic tree (Supplementary Figure S4), samples were not as clearly separated as the tree for VF genes, mostly because the number of identified AMR genes was relatively low compared to the number of identified VF genes.

#### *Sensitivity*

The performance of the workflows was evaluated based on their ability to identify the expected *S. enterica* pathogen, as well as *S. enterica* subspecies and strain taxonomic ranks for the tested samples (Supplementary Table T3). In a metagenomic setting, other detected species cannot be regarded as false positives, as they may naturally be present in the sample. Therefore, only sensitivity was reported.

For the taxonomy profiling (Workflow 2), the expected pathogen was detected at its species taxonomic rank in all but one sample, resulting in a sensitivity of 97.8%. At the subspecies taxonomic rank, the expected subspecies was detected in 28 out of 46 samples, yielding a sensitivity of 64.0%. To further evaluate subspecies classification performance, the sample-wise sensitivity (the percentage of correctly identified *S. enterica* subspecies out of all detected *S. enterica* subspecies) was calculated. Averaged across all samples, the sample-wise sensitivity was 47.3%. In the gene-based pathogen identification (Workflow 3), at least one virulence factor (VF) gene of the expected pathogen, at strain taxonomic rank, was detected in 13 out of 46 samples, corresponding to a sensitivity of 28.2%. For the samples in which no VF gene was detected, no contigs could be generated, preventing gene calling.

Changing the workflow's default settings, such as using different reference databases for preprocessing in Workflow 1, taxonomy profiling in Workflow 2, or gene-based pathogen identification in Workflow 3, would likely impact these metrics. Different reference databases could influence the accuracy and sensitivity of taxonomic classification and pathogen identification, as they may contain varying levels of strain-specific data. Adjusting parameters like threshold values, filtering criteria, or the inclusion of additional databases could also affect the detection sensitivity and overall performance, potentially improving or reducing the workflow's ability to accurately identify pathogens and associated genes in the given samples.

### Samples With Prior Pathogen Isolation

#### *Data Description*

To further test PathoGFair, 84 public datasets were used [75]. These samples were sampled in Palestine by the Swiss Tropical and Public Health Institute either from chicken meat, chicken stool, or human stool, in 2021 or 2022 (Supplementary Figure S5). In these samples, *S. enterica* had been isolated in 19 samples, and *Campylobacter jejuni* in 65 samples. The generated sequencing data are provided under BioProjects PRJNA942086 (S. enterica [76]) and PRJNA942088 (C. jejuni [77]).

#### *Preprocessing*

Negligible contamination or host sequences were found between 0% and 0.02% (Supplementary online Figure S6), as expected because of the prior isolation of the pathogen. The number of reads ranges between 3k and 217k reads per sample, after quality control.

#### *Taxonomy Profiling*

As presented in the interactive KRONA plot (Supplementary online Figure S6), the first 19 samples, *S. enterica* isolates, were assigned correctly to *S. enterica*, and the remaining 65 samples were assigned correctly to *C. jejuni*. With the KRONA plot (Supplementary online Figure S6), the total number of reads for each sample can be seen along with detailed percentages on the assigned taxa at each taxonomic rank.

#### *Gene-based Pathogen Identification*

In this workflow, we identified VF and AMR genes for all samples, thanks to the higher number of reads retained after preprocessing and the prior isolation of the pathogens. Consequently, VF genes were detected in all samples, with more VF genes identified than AMR genes (Supplementary Figure S7), as in general, the number of VF genes in a bacterial genome is often higher than the number of AMR genes. Samples containing *S. enterica* exhibited more VF genes (172 to 207) compared to samples with *C. jejuni* (96 to 113). The opposite trend was observed for AMR genes, *C. jejuni* samples typically had 12 AMR genes detected, while *S. enterica* samples mostly had 6 AMR genes (Supplementary Figure S7)

The analysis revealed that samples with similarly isolated pathogens clustered together based on detected VF genes (4). For example, samples with *S. enterica* and *C. jejuni* formed distinct clusters. Moreover, correlations were observed among samples from different hosts, sampling years, and pathogenic species.

Specific VF genes were found in samples with similar isolated pathogens, indicating potential subspecies-specific differences. For instance, Cj1419c, a methyltransferase Capsule biosynthesis and transport gene product, was exclusively found in *C. jejuni* isolates samples sequenced in 2022, while flgB gene, encoding flagellar basal body rod protein, was only detected in *C. jejuni* isolates samples sequenced in 2021. *flaA* (flagellin), a VF gene product identifying *C. jejuni*, was present in *S. enterica* isolates samples from human stool sampled in 2022 and all *C. jejuni* isolates samples, but not in *S. enterica* isolates samples from chicken meat, chicken stool, or human stool sampled in 2021.

Furthermore, certain VF genes such as *spvC* (type III secretion system effector *SpvC* phosphothreonine lyase) and pefB (plasmid-encoded fimbriae regulatory protein), associated with *S. enterica* subsp. *enterica* ser. Typhimurium str. LT2, were exclusively found in *S. enterica* isolates samples from human stool sampled in 2022. Conversely, *fyuA*, a pesticin/yersiniabactin receptor protein that identifies *Yersinia pestis*, was detected in every *S. enterica* isolates sample except those from human stool sampled in 2022. Finally, some VF genes, like *flif*, a flagellar M-ring protein known in *Yersinia enterocolitica* subsp. *enterocolitica*, were found in all samples, irrespective of the pathogen species.

#### *Allele-based Pathogen Identification*

The 19 *S. enterica* isolates samples were mapped against the reference genome of the expected pathogen, *S. enterica subsp. enterica* ser. Typhimurium (NC_003197.2 [74]), and the 65 *C. jejuni* isolates samples were mapped against *C. jejuni* (NC_002163.1).

The 19 *S. enterica* isolates samples have an average mapping coverage of 94.6% and an average mean mapping depth of 31 per base. The average total number of variants found per *S. enterica* isolates sample was 43,420. For the 65 *C. jejuni* isolates samples, the average mapping coverage was 93.7%, the average mean mapping depth was 42 per base and the average total number of variants found per sample was 26,654. These high values for the average total number of variants identified for samples were expected since the used subspecies for the mapping are different than the subspecies of the samples.

#### *PathoGFAIR Samples Aggregation and Visualisation*

The isolated samples exhibited a higher count of identified AMR genes compared to the metagenomic samples without prior isolation, enabling the incorporation of additional genes into concatenated gene consensus sequences. The resulting phylogenetic tree, constructed based on the AMR genes (Supplementary Figure S8), distinctly delineated different *S. enterica* subspecies . Similarly, this differentiation is evident in the phylogenetic tree based on the VF

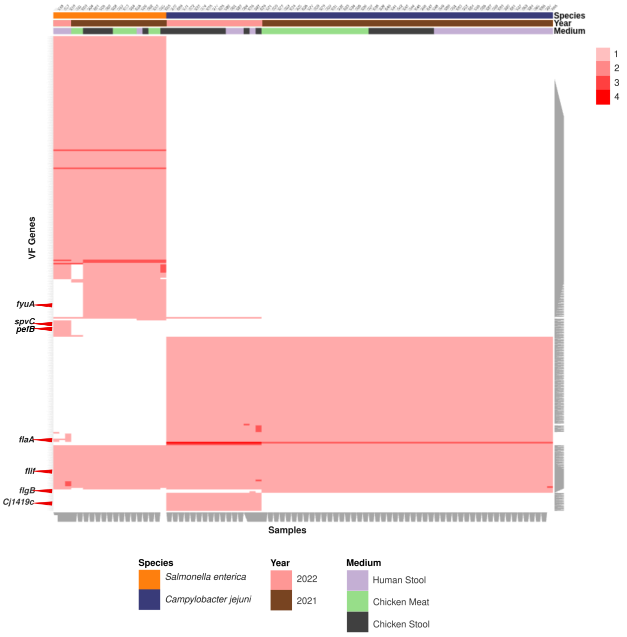


**Figure 4.** Cluster-map showing the identified VF genes on the y-axis for tested samples presented on the x-axis, clustered based on sample information such as sampling year, isolated pathogen species, and the original host of the sample. Clustering was performed using hierarchical clustering implemented in the Clustergrammer Python package

genes.

***Sensitivity***

Known species were successfully identified and confirmed across all workflows, including taxonomy profiling and VF genes identification (Supplementary Online Figure S6), achieving 100% expected pathogen detection at the expected taxonomic rank for all 84 tested samples resulting in a sensitivity of 1. Since there were no true negatives (TN) or conditions without pathogens in the dataset, specificity could not be calculated.

## Benchmarking PathoGFAIR

To evaluate the effectiveness of PathoGFAIR workflows, a benchmarking analysis was performed comparing PathoGFAIR's pathogen detection capabilities with the systems and pipelines listed in Table 1. The primary goal was to assess each pipeline's ability to accurately detect and identify pathogens from shotgun Nanopore metagenomic data using the samples without prior pathogen isolation. The detailed procedures for the selection and benchmarking process can be found on Protocols.io [78]. Replication instructions, including all benchmark systems, sample metadata, notebooks, and results, are publicly available through the PathoGFAIR GitHub repository.

### Selection of the pipelines for the benchmarking

To identify suitable systems and pipelines for the benchmark, each pipeline in Table 1 was evaluated based on availability, accepted input sequencing technique compatibility, and pathogen identification capability (Supplementary Table T4). Pipelines were classified as "Free Access" if available at no cost, "Free Trial" if partially or temporarily accessible, "Paid Access Only" if requiring payment, or "Non-functional" if outdated or inoperative. Compatibility with single-end Nanopore metagenomic sequencing data was verified to ensure each pipeline's applicability for pathogen-focused workflows. Each pipeline's pathogen identification capabilities were also assessed, examining relevant algorithms and tools to determine their accuracy and sensitivity within metagenomic datasets.

Following these criteria, pipelines requiring paid access (e.g. OneCodex) or being non-functional, as well as those incompatible with Nanopore data (e.g., SURPI, Sunbeam, Innuendo, and PAIPline) or lacking robust pathogen identification features, were excluded from further analysis.

Each selected pipeline was subsequently set up and tested to confirm ease of use and reproducibility. During testing, Victors was found to be non-functional, and SURPI presented difficulties, requiring local downloads of large host and pathogen reference databases. SURPI's fragmented documentation contained outdated and contradictory requirements, with no updates since 2014. Innuendo, though documented, also presented usability challenges with incomplete setup instructions. BugSeq, while user-friendly and accessible through its web interface, had an average processing time of four hours from sample upload to results delivery. However, its free trial is limited to just 10 samples, and it lacked transparency regarding the tools, workflows, and databases used for analysis, as it is neither open-source nor adaptable. Ultimately, only IDseq (CZID) and our workflows, PathoGFAIR, met the selection criteria and were included for benchmarking (Supplementary Table T4).

IDseq offers a highly user-friendly interface, similar to PathoGFAIR on the Galaxy platform. However, unlike PathoGFAIR, IDseq's pipeline is limited in terms of adaptability to its workflows, particularly with respect to the tools and parameters used. This restriction can hinder customisation and flexibility for users with specific analytical needs, reducing the pipeline's versatility compared to PathoGFAIR. The IDseq pipeline took one hour for sample uploads and an additional hour and a half to complete the analysis across all samples.

### Benchmarking on samples without prior pathogen isolation

The benchmark was conducted using the 46 samples without prior pathogen isolation, from the first use case. The samples are chicken meat spiked with pathogens and sequenced using Nanopore as explained in [71]. Metadata for the 46 samples is summarised in Supplementary Table T1, including the expected *S. enterica* subspecies (*S. enterica* subsp. *houtenae* DSM 9221, *S. enterica* subsp. *enterica* DSM 554, and/or *S. enterica* subsp. *salamae* DSM 9220). For PathoGFAIR, taxonomy profiling from Workflow 2 and VF gene identification from Workflow 3 were evaluated.

In both PathoGFAIR and IDseq (Supplementary Table T3), *Salmonella enterica* was detected in all samples except one, resulting in a 97.8% detection rate. In the exceptional sample, *Salmonella* was not detected by either system. PathoGFAIR provided additional resolution, identifying the subspecies taxonomic rank in 60.9% of samples and the strain taxonomic rank in 13% of samples. In contrast, IDseq's output report did not provide pathogen information beyond the species rank (Supplementary Figure S9).

Further benchmarking with diverse public metagenomic datasets could offer more comprehensive insights into the performance of PathoGFAIR across different experimental conditions and sample types. Such analyses would help assess the workflow's robustness and adaptability to a wider range of use cases, contributing to its ongoing validation and refinement.

## Conclusion

In conclusion, we present PathoGFAIR, a collection of Galaxy FAIR adaptable workflows, designed for pathogen detection and tracking among samples. These five workflows span the entire analysis pipeline, ranging from preprocessing reads to advanced analyses including taxonomy profiling, virulence and antimicrobial resis-

tance gene identification, SNP detection, and evolutionary history comparisons. The workflows generate diverse visualisations for a comprehensive understanding of the results, accompanied by interactive reports detailing all relevant inputs and outputs.

Our workflows have successfully identified pathogens down to genus, species, or subspecies taxonomic ranks across diverse samples, surpassing limitations observed in comparable pipelines. Our workflows facilitate comprehensive sample comparisons across diverse types, conditions, and sequencing techniques by offering interpretative and publication-ready visualisations. The open-access and user-friendly design of PathoGFAIR mitigates accessibility challenges and reduces reliance on local computational resources by leveraging Galaxy's infrastructure for computational tasks, a feature that sets it apart from similar pipelines. This scalable workflow is a versatile solution for processing (meta)genomic samples, extending its utility beyond detecting foodborne pathogens.

In our findings, optimising sampling, preparation, and sequencing conditions, such as a 24-hour sample incubation, significantly enhances the identification of virulence and antimicrobial resistance genes. Indeed, the workflows' performance correlates with sample characteristics, with higher CFU/mL values and read counts, and lower Ct values yielding more comprehensive results, which can be used to establish sampling guidelines. Moreover, as the Preprocessing workflow effectively removes host sequences, adaptive sampling during sequencing to exclude host DNA is not necessary. The workflows were still able to detect pathogens at least at species taxonomic rank for samples without prior pathogen isolation.

The experimental setup for this study serves as a proof of concept, demonstrating the feasibility of using WGS for the detection and characterisation of *S. enterica* in spiked food matrices. By detecting pathogens across varying CFU/mL levels, as shown in the spiking experiments, the workflows showcase their sensitivity and practical applicability in adhering to stringent regulatory standards. These results establish a solid foundation for applying PathoGFAIR in food safety laboratories and outbreak investigations, where detailed subspecies-rank information is critical for monitoring and traceability.

PathoGFAIR's utility extends to enrichment-based analyses of foodborne pathogens, aligning with EU food safety standards such as EN/ISO 6579. Traditionally, these standards require enrichment followed by classical microbiological methods or PCR confirmation. PathoGFAIR complements these approaches by enabling direct analysis of enrichment broths through WGS, facilitating efficient detection and subspecies characterisation of *S. enterica*. This capability enhances food safety monitoring and outbreak response by differentiating *S. enterica* subspecies from previous outbreak strains, thereby advancing traceability and improving public health interventions.

We further supported the scientific community by introducing new 46 benchmark samples, making them publicly available. This demonstrates our significant investment of time and resources, providing valuable assets for future research.

In addition to the allele-based pathogen identification method, our workflow can be further enhanced by incorporating MLST. MLST, or Multi-Locus Sequence Typing, offers an alternative approach by characterising isolates through the sequences of housekeeping genes [49]. This method provides valuable information about the genetic diversity and evolutionary relationships among isolates, allowing for more precise identification and tracing of pathogens. By integrating MLST using MLST (v 2.22.0) tool [79]into our workflow, users can benefit from a comprehensive analysis that combines both alleles and variants identification methods, providing a more robust and accurate pathogen detection and tracing solution.

To address the complexity of detecting virulence and AMR genes located on mobile genetic elements (MGEs), future versions of PathoGFAIR can incorporate additional validation steps. Specifically, virulence and AMR gene detection could be cross-referenced with broader taxonomic markers (e.g., 16S rRNA) to ensure genes detected from MGEs are correctly attributed to their respective pathogenic hosts. These enhancements aim to further improve the accuracy of pathogen detection by more reliably linking virulence and AMR genes to their bacterial hosts, thereby refining the overall precision of the workflows.

In the future, integrating PathoGFAIR with Galaxy's automated bot system holds the promise of ongoing updates and analyses requiring minimal human involvement. By establishing a dedicated bot for PathoGFAIR, continuous results will be effortlessly refreshed whenever new datasets are uploaded, similar to the Galaxy bot created for SARS-CoV-2 [80]. The Galaxy bot for SARS-CoV-2 automatically updates and reanalyses data with each new upload, maintaining up-to-date results and reducing the need for manual intervention. This automation ensures real-time, efficient data processing and analysis, enhancing the workflow's accuracy and timeliness. Leveraging the user-friendly interface of the Galaxy platform ensures accessibility for users of all computational skill levels, streamlining the entire process from sample upload to result interpretation with ease. This study not only presents a robust computational solution but also lays the groundwork for semi-automated, efficient, and user-friendly pathogen detection and tracking workflows.

## Additional Files

Supplementary Figure S1. Violin plot with the percentage of quality-controlled host reads detected and removed in samples with respect to adaptive sampling during sequencing (Host excluded or not) - Samples Without Prior Pathogen Isolation

Supplementary online Figure S2. Krone Pie Chart for the taxonomy profiling - Samples Without Prior Pathogen Isolation

Supplementary Figure S3. Bar chart for the total number of VF genes (orange) and AMR genes (blue) found in samples with respect to incubation duration before DNA isolation - Samples Without Prior Pathogen Isolation

Supplementary Figure S4. Phylogenetic tree, using the nucleotide evolution model; General Time Reversible (GTR) model with a CAT approximation for rate heterogeneity across sites [54], for the identified AMR genes - Samples Without Prior Pathogen Isolation

Supplementary Figure S5. Upset plot illustrating the intersections of different metadata categories, including sampling year, pathogen species, and the original host of the samples, highlighting common and unique attributes among the datasets - Samples With Prior Pathogen Isolation

Supplementary online Figure S6. Krona Pie Chart for the taxonomy profiling - Samples With Prior Pathogen Isolation

Supplementary Figure S7. Violin plot for the total number of VFs and AMR genes - Samples With Prior Pathogen Isolation

Supplementary Figure S8. Phylogenetic tree, using the nucleotide evolution model; General Time Reversible (GTR) model with a CAT approximation for rate heterogeneity across sites [54], for the identified AMR genes - Samples With Prior Pathogen Isolation

Supplementary Figure S9. Heatmap with PathoGFAIR benchmark using the first Use Case datasets and compare it with similar analysis systems presented in Table 1

Supplementary Table T1. Metadata for Samples Without Prior Pathogen Isolation

Supplementary Table T2. Metadata for Samples With Prior Pathogen Isolation

Supplementary Table T3. PathoGFAIR and IDSeq pathogen detection results for Samples Without Prior Isolation at various taxonomic ranks

Supplementary Table T4. Benchmarking PathoGFAIR systems and pipelines evaluation

## Availability of Source Code and Requirements

Lists the following:

- Project name: PathoGFAIR https://usegalaxy-eu.github.io/PathoGFAIR/
- Workflows on public Galaxy servers: https://training.galaxyproject.org/training-material/workflows/embed.html?query=pathogfair
- Workflows (v 0.1) on WorkflowHub: https://workflowhub.eu/search?utf8=%E2%9C%93&q=pathogfair
- Workflows (v 0.1) on Dockstore: https://dockstore.org/search?organization=iwc-workflows&entryType=workflows&search=engy
- Tutorial: https://training.galaxyproject.org/training-material/topics/microbiome/tutorials/pathogen-detection-from-nanopore-foodborne-data/tutorial.html
- Data analysis home page: https://github.com/usegalaxy-eu/PathoGFAIR
- Operating system(s): Platform independent
- Other requirements: Account on a Galaxy server
- License: MIT license

## Data Availability

The raw sequence reads of the 46 samples without prior isolation are available on Sequence Read Archive (SRA) under BioProjects [81]. The protocol for the preparation of these samples is available on Protocols.io [71]. The workflows presented in the Methods section are available on Intergalactic Workflow Commission (IWC) and two workflow registries (Dockstore and WorkflowHub). The training material to understand, learn, and try the workflows is available on the Galaxy Training Network (GTN) [68]. The Jupyter notebook for additional visualisations and generating the figures of this paper is available in a GitHub repository [82]. Benchmarking PathoGFAIR protocol is available on protocols.io [78].

## Declarations

### List of Abbreviations

AMR: Antimicrobial resistance; API: Application Programming Interface; CFU: Colony-forming unit; Ct: Cycle Threshold; EFSA: European Food Safety Authority; EU: European Union; FAIR: Findable Accessible Interoperable Resulable; GTN: Galaxy Training Network; IWC: Intergalactic Workflow Commission; MLST: Multilocus sequence typing; NGS: Next-Generation Sequencing; QC: Quality Control; RKI: Robert Koch Institute; SNP: Single-nucleotide polymorphism; SRA: Sequence Read Archive; VF: Virulence Factor; VFDB: Virulence Factor database; WHO: World Health Organization; WGS: Whole Genome Sequencing.

### Funding

This research was supported by the Digital Life Science Call for Academia-Industry Collaborations under EOSC-Life funding [83]. Additionally, financial support was provided by the German Federal Ministry of Education and Research BMBF grant 031 A538A de.NBI-RBC and the Ministry of Science, Research and the Arts Baden-Württemberg (MWK) within the framework of LIBIS/de.NBI Freiburg. This work was supported by the Programme d'Investissements d'Avenir (PIA), grant Agence Nationale de la Recherche, number ANR-11-INBS-0013.

### Author's Contributions

Engy Nasr (E.N.) led the formal analysis, investigation, software development, validation, visualisation, and writing of both the original draft and the review and editing phases. E.N. also designed the workflows, tested them, created training materials, documented and published datasets, workflows, codes, protocols, and wrote the manuscript. Anna Henger (A.H.) supported conceptualisation, formal analysis, funding acquisition, investigation, project administration, validation, visualisation, and writing. A.H. also prepared and sequenced all project datasets, ensuring alignment of analysis results with lab preparation conditions. Björn Grüning (B.G.) supported conceptualisation, formal analysis, funding acquisition, investigation, project administration, and software development. B.G. integrated new databases into analytical tools and maintained Galaxy tools used in PathoGFAIR. Paul Zierep (P.Z.) contributed to formal analysis, investigation, software development, supervision, validation, visualisation, and writing. P.Z. supervised the project and edited parts of the workflows and training materials. Bérénice Batut (B.B.) led the conceptualisation, funding acquisition, methodology, project administration, software development, and supervision. B.B. applied for the EOSC Life Industry Call Grant, designed the project's main goals and guidelines, and managed and edited the full project. All authors read and approved the final manuscript.

## Acknowledgements

This research is made possible by the invaluable support of the entire Freiburg Galaxy team, Bioinformatics, University of Freiburg. The authors extend their special thanks to Wolfgang Maier and Mina Ansari for their technical expertise and academic guidance. We also appreciate the contributions of all the researchers who provided input to the project, with particular gratitude to Tobias Schindler for his exceptional assistance and to Peter van Heusden for his insightful contributions.

## References

1. Elbehiry A, Abalkhail A, Marzouk E, Elmanssury AE, Almuzaini AM, Alfheeaid H, et al. An Overview of the Public Health Challenges in Diagnosing and Controlling Human Foodborne Pathogens. Vaccines 2023 Mar;11(4):725. https://www.mdpi.com/2076-393X/11/4/725.
2. Wei X, Zhao X. Advances in typing and identification of foodborne pathogens. Current Opinion in Food Science 2021 Feb;37:52–57. https://linkinghub.elsevier.com/retrieve/pii/S2214799320300692, doi:10.1016/j.cofs.2020.09.002.
3. Organization WH. WHO global strategy for food safety 2022–2030: towards stronger food safety systems and global cooperation: executive summary. World Health Organization; 2022. https://www.who.int/publications/i/item/9789240057685, accessed 24 June 2024.
4. Priyanka B, Patil RK, Dwarakanath S. A review on detection methods used for foodborne pathogens. Indian Journal of Medical Research 2016;144(3):327. http://www.ijmr.org.in/text.asp?2016/144/3/327/198677.
5. Yang S, Johnson MA, Hansen MA, Bush E, Li S, Vinatzer BA. Metagenomic sequencing for detection and identification of the boxwood blight pathogen Calonectria pseudonaviculata. Scientific Reports 2022 Jan;12(1):1399. https://www.nature.com/articles/s41598-022-05381-x.
6. Thomas T, Gilbert J, Meyer F. Metagenomics - a guide from sampling to data analysis. Microbial Informatics and Experimentation 2012 Dec;2(1):3. https://microbialinformaticsj.biomedcentral.com/articles/10.1186/2042-5783-2-3.

7. Thompson LR, Sanders JG, McDonald D, Amir A, Ladau J, Locey KJ, et al. A communal catalogue reveals Earth's multiscale microbial diversity. Nature 2017 Nov;551(7681):457–463. https://www.nature.com/articles/nature24621.
8. Bogaerts B, Van den Bossche A, Verhaegen B, Delbrassinne L, Mattheus W, Nouws S, et al. Closing the gap: Oxford Nanopore Technologies R10 sequencing allows comparable results to Illumina sequencing for SNP-based outbreak investigation of bacterial pathogens. Journal of Clinical Microbiology 2024 Mar;0(0):e01576–23. https://journals.asm.org/doi/10.1128/jcm.01576-23, doi:10.1128/jcm.01576-23.
9. Joensen KG, Scheutz F, Lund O, Hasman H, Kaas RS, Nielsen EM, et al. Real-Time Whole-Genome Sequencing for Routine Typing, Surveillance, and Outbreak Detection of Verotoxigenic Escherichia coli. Journal of Clinical Microbiology 2014 May;52(5):1501–1510. https://journals.asm.org/doi/10.1128/JCM.03617-13, doi:10.1128/JCM.03617-13.
10. Allard MW, Bell R, Ferreira CM, Gonzalez-Escalona N, Hoffmann M, Muruvanda T, et al. Genomics of foodborne pathogens for microbial food safety. Current Opinion in Biotechnology 2018 Feb;49:224–229. https://linkinghub.elsevier.com/retrieve/pii/S0958166917301398, doi:10.1016/j.copbio.2017.11.002.
11. Fan J, Huang S, Chorlton SD. BugSeq: a highly accurate cloud platform for long-read metagenomic analyses. BMC Bioinformatics 2021 Dec;22(1):160. https://bmcbioinformatics.biomedcentral.com/articles/10.1186/s12859-021-04089-5.
12. One Codex | A fast, easy-to-use platform for microbiome sequencing and analysis;, accessed 24 June 2024.
13. Naccache SN, Federman S, Veeraraghavan N, Zaharia M, Lee D, Samayoa E, et al. A cloud-compatible bioinformatics pipeline for ultrarapid pathogen identification from next-generation sequencing of clinical samples. Genome Research 2014 Jul;24(7):1180–1192. http://genome.cshlp.org/lookup/doi/10.1101/gr.171934.113, doi:10.1101/gr.171934.113.
14. Clarke EL, Taylor LJ, Zhao C, Connell A, Lee JJ, Fett B, et al. Sunbeam: an extensible pipeline for analyzing metagenomic sequencing experiments. Microbiome 2019 Dec;7(1):46. https://microbiomejournal.biomedcentral.com/articles/10.1186/s40168-019-0658-x, doi:10.1186/s40168-019-0658-x.
15. Kalantar KL, Carvalho T, de Bourcy CFA, Dimitrov B, Dingle G, Egger R, et al. IDseq—An open source cloud-based pipeline and analysis service for metagenomic pathogen detection and monitoring. GigaScience 2020 Oct;9(10):giaa111. https://academic.oup.com/gigascience/article/doi/10.1093/gigascience/giaa111/5918865, doi:10.1093/gigascience/giaa111.
16. Chan Zuckerberg ID - Detect & Track Infectious Diseases;, accessed 24 June 2024.
17. The Galaxy Community, Afgan E, Nekrutenko A, Grüning BA, Blankenberg D, Goecks J, et al. The Galaxy platform for accessible, reproducible and collaborative biomedical analyses: 2022 update. Nucleic Acids Research 2022 Jul;50(W1):W345–W351. https://academic.oup.com/nar/article/50/W1/W345/6572001, doi:10.1093/nar/gkac247.
18. Yuen D, Cabansay L, Duncan A, Luu G, Hogue G, Overbeck C, et al. The Dockstore: enhancing a community platform for sharing reproducible and accessible computational protocols. Nucleic Acids Research 2021 Jul;49(W1):W624–W632. https://doi.org/10.1093/nar/gkab346.
19. Implementing FAIR Digital Objects in the EOSC-Life Workflow Collaboratory;, https://zenodo.org/records/4605654, doi:10.5281/zenodo.4605654. Accessed 25 June 2024.
20. Hiltemann S, Rasche H, Gladman S, Hotz HR, Larivière D, Blankenberg D, et al. Galaxy Training: A powerful framework for teaching! PLOS Computational Biology 2023 Jan;19(1):e1010752. https://dx.plos.org/10.1371/journal.pcbi.1010752, doi:10.1371/journal.pcbi.1010752.
21. Llarena A, Ribeiro-Gonçalves BF, Nuno Silva D, Halkilahti J, Machado MP, Da Silva MS, et al. INNUENDO: A cross-sectoral platform for the integration of genomics in the surveillance of food-borne pathogens. EFSA Supporting Publications 2018 Nov;15(11). https://data.europa.eu/doi/10.2903/sp.efsa.2018.EN-1498, doi:10.2903/sp.efsa.2018.EN-1498.
22. Andrusch A, Dabrowski PW, Klenner J, Tausch SH, Kohl C, Osman AA, et al. PAIPline: pathogen identification in metagenomic and clinical next generation sequencing samples. Bioinformatics 2018 Sep;34(17):i715–i721. https://academic.oup.com/bioinformatics/article/34/17/i715/5093217, doi:10.1093/bioinformatics/bty595.
23. Sayers S, Li L, Ong E, Deng S, Fu G, Lin Y, et al. Victors: a web-based knowledge base of virulence factors in human and animal pathogens. Nucleic Acids Research 2019 Jan;47(D1):D693–D700. https://dx.doi.org/10.1093/nar/gky999, doi:10.1093/nar/gky999.
24. Chen S, Zhou Y, Chen Y, Gu J. fastp: an ultra-fast all-in-one FASTQ preprocessor. Bioinformatics 2018 Sep;34(17):i884–i890. https://academic.oup.com/bioinformatics/article/34/17/i884/5093234, doi:10.1093/bioinformatics/bty560.
25. Porechop — de.NBI Nanopore Training Course latest documentation;, https://denbi-nanopore-training-course.readthedocs.io/en/latest/read_qc/Porechop_1.html, accessed 24 June 2024.
26. Wagner GE, Dabernig-Heinz J, Lipp M, Cabal A, Simantzik J, Kohl M, et al. Real-Time Nanopore Q20+ Sequencing Enables Extremely Fast and Accurate Core Genome MLST Typing and Democratizes Access to High-Resolution Bacterial Pathogen Surveillance. Journal of Clinical Microbiology 2023 Apr;61(4):e01631–22. https://journals.asm.org/doi/10.1128/jcm.01631-22.
27. Li H. Minimap2: pairwise alignment for nucleotide sequences. Bioinformatics 2018 Sep;34(18):3094–3100. https://academic.oup.com/bioinformatics/article/34/18/3094/4994778, doi:10.1093/bioinformatics/bty191.
28. Chaisson MJ, Tesler G. Mapping single molecule sequencing reads using basic local alignment with successive refinement (BLASR): application and theory. BMC Bioinformatics 2012 Dec;13(1):238. https://bmcbioinformatics.biomedcentral.com/articles/10.1186/1471-2105-13-238, doi:10.1186/1471-2105-13-238.
29. Li H, Durbin R. Fast and accurate short read alignment with Burrows–Wheeler transform. Bioinformatics 2009 Jul;25(14):1754–1760. https://academic.oup.com/bioinformatics/article/25/14/1754/225615, doi:10.1093/bioinformatics/btp324.
30. Sedlazeck FJ, Rescheneder P, Smolka M, Fang H, Nattestad M, Von Haeseler A, et al. Accurate detection of complex structural variations using single-molecule sequencing. Nature Methods 2018 Jun;15(6):461–468. https://www.nature.com/articles/s41592-018-0001-7, doi:10.1038/s41592-018-0001-7.
31. Wu TD, Watanabe CK. GMAP: a genomic mapping and alignment program for mRNA and EST sequences. Bioinformatics 2005 May;21(9):1859–1875. https://academic.oup.com/bioinformatics/article-lookup/doi/10.1093/bioinformatics/bti310, doi:10.1093/bioinformatics/bti310.
32. Langmead B, Salzberg SL. Fast gapped-read alignment with Bowtie 2. Nature Methods 2012 Apr;9(4):357–359. https://www.nature.com/articles/nmeth.1923, doi:10.1038/nmeth.1923.
33. Lu J, Rincon N, Wood DE, Breitwieser FP, Pockrandt C, Langmead B, et al. Metagenome analysis using the Kraken software suite. Nature Protocols 2022 Dec;17(12):2815–2839. https://www.nature.com/articles/s41596-022-00738-y, doi:10.1038/s41596-022-00738-y.
34. Katz LS, Griswold T, Lindsey R, Lauer A, Im MS, Williams G, et al. Kraken with Kalamari: Contamination Detection; 2021,

accessed 24 June 2024.
35. Ewels P, Magnusson M, Lundin S, Käller M. MultiQC: summarize analysis results for multiple tools and samples in a single report. Bioinformatics 2016 Oct;32(19):3047–3048. https://academic.oup.com/bioinformatics/article/32/19/3047/2196507, doi:10.1093/bioinformatics/btw354.
36. Nanoplot — RCAC Biocontainers v1.0 documentation;, https://biocontainer-doc.readthedocs.io/en/latest/source/nanoplot/nanoplot.html, accessed 24 June 2024.
37. Portik DM, Brown CT, Pierce-Ward NT. Evaluation of taxonomic classification and profiling methods for long-read shotgun metagenomic sequencing datasets. Bioinformatics; 2022.
38. Leidenfrost RM, Pöther DC, Jäckel U, Wünschiers R. Benchmarking the MinION: Evaluating long reads for microbial profiling. Scientific Reports 2020;10(1):5125. https://www.nature.com/articles/s41598-020-61989-x, doi:10.1038/s41598-020-61989-x.
39. Govender KN, Eyre DW. Benchmarking taxonomic classifiers with Illumina and Nanopore sequence data for clinical metagenomic diagnostic applications. Microbial Genomics 2022 Oct;8(10). https://www.microbiologyresearch.org/content/journal/mgen/10.1099/mgen.0.000886, doi:10.1099/mgen.0.000886.
40. Meyer F, Fritz A, Deng ZL, Koslicki D, Lesker TR, Gurevich A, et al. Critical Assessment of Metagenome Interpretation: the second round of challenges. Nature Methods 2022 Apr;19(4):429–440. https://www.nature.com/articles/s41592-022-01431-4.
41. Ondov BD, Bergman NH, Phillippy AM. Interactive metagenomic visualization in a Web browser. BMC Bioinformatics 2011 Dec;12(1):385. https://bmcbioinformatics.biomedcentral.com/articles/10.1186/1471-2105-12-385, doi:10.1186/1471-2105-12-385.
42. Bik HM, Pitch Interactive. Phinch: An interactive, exploratory data visualization framework for –Omic datasets. Genomics; 2014.
43. Breitwieser FP, Salzberg SL. Pavian: Interactive analysis of metagenomics data for microbiomics and pathogen identification; 2016, https://www.biorxiv.org/content/10.1101/084715v1, doi:10.1101/084715.
44. Lin Y, Yuan J, Kolmogorov M, Shen MW, Chaisson M, Pevzner PA. Assembly of long error-prone reads using de Bruijn graphs. Proceedings of the National Academy of Sciences 2016 Dec;113(52). https://pnas.org/doi/full/10.1073/pnas.1604560113, doi:10.1073/pnas.1604560113.
45. Oxford Nanopore Technologies. Medaka;, https://github.com/nanoporetech/medaka, accessed 24 June 2024.
46. Seemann T. ABRicate;, https://github.com/tseemann/abricate, accessed 24 June 2024. Version: 2014-07-17.
47. Chen L, Zheng D, Liu B, Yang J, Jin Q. VFDB 2016: hierarchical and refined dataset for big data analysis—10 years on. Nucleic Acids Research 2016 Jan;44(D1):D694–D697. https://academic.oup.com/nar/article-lookup/doi/10.1093/nar/gkv1239, doi:10.1093/nar/gkv1239.
48. Feldgarden M, Brover V, Haft DH, Prasad AB, Slotta DJ, Tolstoy I, et al. Validating the AMRFinder Tool and Resistance Gene Database by Using Antimicrobial Resistance Genotype-Phenotype Correlations in a Collection of Isolates. Antimicrobial Agents and Chemotherapy 2019 Nov;63(11):e00483–19. https://journals.asm.org/doi/10.1128/AAC.00483-19, doi:10.1128/AAC.00483-19.
49. Pearce ME, Alikhan NF, Dallman TJ, Zhou Z, Grant K, Maiden MCJ. Comparative analysis of core genome MLST and SNP typing within a European Salmonella serovar Enteritidis outbreak. International Journal of Food Microbiology 2018;274:1–11. https://linkinghub.elsevier.com/retrieve/pii/S0168160518300746, doi:10.1016/j.ijfoodmicro.2018.02.023.
50. Hong Kong University - Biomedical Algorithms Lab (BAL). Clair3 - Symphonizing pileup and full-alignment for high-performance long-read variant calling;, original Release Date: 2021-03-30. Accessed 25 June 2024.
51. Danecek P, Bonfield JK, Liddle J, Marshall J, Ohan V, Pollard MO, et al. Twelve years of SAMtools and BCFtools. GigaScience 2021 Jan;10(2):giab008. https://academic.oup.com/gigascience/article/doi/10.1093/gigascience/giab008/6137722.
52. Cingolani P, Platts A, Wang LL, Coon M, Nguyen T, Wang L, et al. A program for annotating and predicting the effects of single nucleotide polymorphisms, SnpEff: SNPs in the genome of Drosophila melanogaster strain w $^{1118}$ ; iso-2; iso-3. Fly 2012 Apr;6(2):80–92. http://www.tandfonline.com/doi/abs/10.4161/fly.19695.
53. Danecek P, McCarthy SA. BCFtools/csq: Haplotype-aware variant consequences;, https://www.biorxiv.org/content/10.1101/090811v2, accessed 24 June 2024.
54. Price MN, Dehal PS, Arkin AP. FastTree 2 – Approximately Maximum-Likelihood Trees for Large Alignments. PLoS ONE 2010 Mar;5(3):e9490. https://dx.plos.org/10.1371/journal.pone.0009490, doi:10.1371/journal.pone.0009490.
55. jupyter/jupyter: Jupyter metapackage for installation, docs and chat;, https://github.com/jupyter/jupyter/tree/master, accessed 24 June 2024.
56. Van Rossum G, Drake FL. Python 3 Reference Manual. Scotts Valley, CA: CreateSpace; 2009.
57. Wes McKinney. Data Structures for Statistical Computing in Python. In: Stéfan van der Walt, Jarrod Millman, editors. Proceedings of the 9th Python in Science Conference; 2010. p. 56 – 61.
58. pandas development team T, pandas-dev/pandas: Pandas. Zenodo; 2020. https://doi.org/10.5281/zenodo.3509134.
59. Hunter JD. Matplotlib: A 2D graphics environment. Computing in Science & Engineering 2007;9(3):90–95.
60. Waskom M. seaborn: statistical data visualization. Journal of Open Source Software 2021 Apr;6(60):3021. https://joss.theoj.org/papers/10.21105/joss.03021.
61. Harris CR, Millman KJ, van der Walt SJ, Gommers R, Virtanen P, Cournapeau D, et al. Array programming with NumPy. Nature 2020 Sep;585(7825):357–362. https://doi.org/10.1038/s41586-020-2649-2.
62. Martin M. Cutadapt removes adapter sequences from high-throughput sequencing reads. EMBnetjournal 2011 May;17(1):10. http://journal.embnet.org/index.php/embnetjournal/article/view/200, doi:10.14806/ej.17.1.200.
63. Bray S, Chilton J, Bernt M, Soranzo N, van den Beek M, Batut B, et al. The Planemo toolkit for developing, deploying, and executing scientific data analyses in Galaxy and beyond. Genome Research 2023;33(2):261–268. https://genome.cshlp.org/content/33/2/261, doi:10.1101/gr.276963.122.
64. Wilkinson MD, Dumontier M, Aalbersberg IJ, Appleton G, Axton M, Baak A, et al. The FAIR Guiding Principles for scientific data management and stewardship. Scientific Data 2016 Mar;3(1):160018. https://www.nature.com/articles/sdata201618.
65. Chue Hong NP, Katz DS, Barker M, Lamprecht AL, Martinez C, Psomopoulos FE, et al. FAIR Principles for Research Software (FAIR4RS Principles); 2021, https://rd-alliance.org/group/fair-research-software-fair4rs-wg/outcomes/fair-principles-research-software-fair4rs, doi:10.15497/RDA00065. Accessed 24 June 2024.
66. Visser Cd, Johansson LF, Kulkarni P, Mei H, Neerincx P, Velde KJvd, et al. Ten quick tips for building FAIR workflows. PLOS Computational Biology 2023 Sep;19(9):e1011369. https://journals.plos.org/ploscompbiol/article?id=10.1371/journal.pcbi.1011369.
67. Project G. Galaxy Workflows maintained by the Intergalactic Workflow Commission; 2024, accessed 24 June 2024. Original date: June 30, 2018.

68. Pathogen detection from (direct Nanopore) sequencing data using Galaxy - Foodborne Edition;, https://training.galaxyproject.org/training-material/topics/metagenomics/tutorials/pathogen-detection-from-nanopore-foodborne-data/tutorial.html, accessed 24 June 2024.
69. Soiland-Reyes S, Sefton P, Crosas M, Castro LJ, Coppens F, Fernández JM, et al. Packaging research artefacts with RO-Crate. Data Science 2022 Jan;5(2):97–138. https://content.iospress.com/articles/data-science/ds210053.
70. Sefton P, Ó Carragáin E, Soiland-Reyes S, Corcho O, Garijo D, Palma R, et al. RO-Crate Metadata Specification 1.1.3; 2023, https://zenodo.org/records/7867028, doi:10.5281/zenodo.7867028. Accessed 25 June 2024.
71. Nasr E, Henger A, Grüning B, Zierep P, Batut B. Samples Preparation for Foodborne Pathogen Detection and Tracking project; 2023, https://www.protocols.io/view/samples-preparation-for-foodborne-pathogen-detecti-cwhdxb26, doi:dx.doi.org/10.17504/protocols.io.8epv5x1jdg1b/v1. Accessed 01 July 2024.
72. CFU Full Form;, https://unacademy.com/content/neet-ug/full-forms/cfu/, accessed 24 June 2024.
73. Bioscientia. What do the terms dual target PCR and Ct value mean? | Laboratory Diagnostics; 2020, https://www.bioscientia.de/en/home/our-news/2020/07/what-do-the-terms-dual-target-pcr-and-ct-value-mean/, accessed 24 June 2024.
74. McClelland M, Sanderson KE, Spieth J, Clifton SW, Latreille P, Courtney L, et al. Complete genome sequence of Salmonella enterica serovar Typhimurium LT2. Nature 2001;413(6858):852–856. https://www.nature.com/articles/35101614, doi:10.1038/35101614.
75. Abukhattab S, Hosch S, Abu-Rmeileh NME, Hasan S, Vonaesch P, Crump L, et al. Whole-genome sequencing for One Health surveillance of antimicrobial resistance in conflict zones: a case study of Salmonella spp. and Campylobacter spp. in the West Bank, Palestine. Applied and Environmental Microbiology 2023 Sep;89(9):e00658–23. https://journals.asm.org/doi/10.1128/aem.00658-23, doi:10.1128/aem.00658-23.
76. Salmonella enterica subsp. enterica (ID 942086) - BioProject - NCBI;, https://www.ncbi.nlm.nih.gov/bioproject/942086, accessed 24 June 2024.
77. Campylobacter jejuni subsp. jejuni (ID 942088) - BioProject - NCBI;, https://www.ncbi.nlm.nih.gov/bioproject/942088, accessed 24 June 2024.
78. Nasr E, Henger A, Grüning B, Zierep P, Batut B. Benchmarking PathoGFAIR; 2024, https://www.protocols.io/view/benchmarking-pathogfair-e6nvwbp4zvmk/v1, doi:dx.doi.org/10.17504/protocols.io.e6nvwbp4zvmk/v1. Accessed 14 November 2024.
79. Seemann T. mlst;, original Release Date: 2014-05-03. Accessed 24 June 2024.
80. Maier W, Bray S, van den Beek M, Bouvier D, Coraor N, Miladi M, et al. Ready-to-use public infrastructure for global SARS-CoV-2 monitoring. Nature Biotechnology 2021 Oct;39(10):1178–1179. https://www.nature.com/articles/s41587-021-01069-1, doi:10.1038/s41587-021-01069-1.
81. PRJNA982679 - SRA - NCBI;, https://www.ncbi.nlm.nih.gov/sra/PRJNA982679, accessed 24 June 2024.
82. usegalaxy-eu/PathoGFAIR: PathoGFAIR: Galaxy FAIR Workflows for Pathogen Detection and Samples Comparison;, https://github.com/usegalaxy-eu/PathoGFAIR, accessed 25 June 2024.
83. Digital Life Sciences Internal Call for Academia-Industry Collaborations;, https://www.eosc-life.eu/industrycall/, accessed 24 June 2024.

Manuscript
Click here to access/download;Manuscript;mansucript.pdf

*GigaScience*, 2023, 1–15

doi: xx.xxxx/xxxx
Manuscript in Preparation
Technical Note

TECHNICAL NOTE

# PathoGFAIR: a collection of FAIR and adaptable (meta)genomics workflows for (foodborne) pathogens detection and tracking

Engy Nasr[1], Anna Henger[2], Björn Grüning[1], Paul Zierep[1] and Bérénice Batut[3,4,*]

[1]Bioinformatics Group, Department of Computer Science, University of Freiburg, Georges-Koehler-Allee 106, 79110 Freiburg im Breisgau, Germany and [2]Biolytix AG, 4243 Dittingen, Switzerland and [3]CNRS, Institut Français de Bioinformatique, IFB-Core, UAR 3601, 91000 Evry, France and [4]Plateforme AuBi, Mésocentre Clermont-Auvergne, Université Clermont Auvergne, 63170 Aubière, France

[*]berenice.batut@gmail.com

## Abstract

**Background:** Food contamination by pathogens poses a global health threat, affecting an estimated 600 million people annually. During a foodborne outbreak investigation, microbiological analysis of food vehicles detects responsible pathogens and traces contamination sources. Metagenomic approaches offer a comprehensive view of the genomic composition of microbial communities, facilitating the detection of potential pathogens in samples. Combined with sequencing techniques like Oxford Nanopore sequencing, such metagenomic approaches become faster and easier to apply. A key limitation of these approaches is the lack of accessible, easy-to-use, and openly available pipelines for pathogen identification and tracking from (meta)genomic data. **Findings:** PathoGFAIR is a collection of Galaxy-based FAIR workflows employing state-of-the-art tools to detect and track pathogens from metagenomic Nanopore sequencing. Although initially developed to detect pathogens in food datasets, the workflows can be applied to other metagenomic Nanopore pathogenic data. PathoGFAIR incorporates visualisations and reports for comprehensive results. We tested PathoGFAIR on 130 samples containing different pathogens from multiple hosts under various experimental conditions. For all but one sample, workflows have successfully detected expected pathogens at least at the species rank. Further taxonomic ranks are detected for samples with sufficiently high Colony-forming unit (CFU) and low Cycle Threshold (Ct) values. **Conclusions:** PathoGFAIR detects the pathogens at species and subspecies taxonomic ranks in all but one tested sample, regardless of whether the pathogen is isolated or the sample is incubated before sequencing. Importantly, PathoGFAIR is easy to use and can be straightforwardly adapted and extended for other types of analysis and sequencing techniques, making it usable in various pathogen detection scenarios.

**Key words**: Galaxy; Public health; Nanopore; Pipeline; Open source; Benchmark samples; Visualisation

## Introduction

Foodborne pathogens pose a significant threat to public health worldwide, causing millions of cases of illness and even death every year [1, 2]. These diverse microorganisms, spanning bacteria, viruses, parasites, and fungi, can contaminate a variety of foods, leading to both localised outbreaks and widespread epidemics. Ensuring food safety and controlling foodborne pathogens are key priorities for public health authorities at local, regional, and global levels, including agencies such as the European Food Safety Authority (EFSA), European Centre for Disease Prevention and Control (ECDC), and the World Health Organisation (WHO) [3].

Traditional methods for identifying the source of food contamination require isolation of the target pathogen. This process is not only time-consuming but can be labor-intensive, often requiring multiple steps and sophisticated techniques, and lacks a guaranteed

**Compiled on:** December 5, 2024.
Draft manuscript prepared by the author.

success rate [4]. In contrast, shotgun metagenomic approaches provide a solution to these challenges, as they give an overview of the genomic composition in the sample, including the food source itself, the microbial community, and any possible pathogens and their complete genetic information [5]. Importantly, shotgun metagenomic approaches eliminate the need for prior isolation of the targeted pathogen, as required by Whole Genome Sequencing (WGS) methods, and they are not limited to specific genes as opposed to real-time PCR approaches [6] or 16S rRNA sequencing . While 16S rRNA sequencing is widely used for bacterial taxonomic profiling, it is limited in scope compared to shotgun metagenomic sequencing. The latter allows for the detection of a wide range of pathogens, including bacteria, viruses, and fungi, and gives access to the full genomes enabling the taxa-agnostic identification of antimicrobial resistance (AMR) and virulence genes. This broader scope makes shotgun sequencing more suitable for comprehensive pathogen detection, especially in complex foodborne outbreak investigations [7].

Nanopore sequencing provides long-read data that can capture comprehensive genetic information. Its utilisation, as exemplified by studies like [8], demonstrates its utility in closing genomic gaps, delivering real-time sequencing data, and enhancing the capabilities of metagenomic approaches for outbreak investigations. This technology enables more accurate and rapid pathogen detection, a critical advancement in scenarios where timely responses are essential for effective outbreak management.

Once sequencing data is generated, it must be processed using bioinformatics tools to identify pathogens, their genetic variations, and Virulence Factor (VF) genes, thereby facilitating timely and accurate detection [9, 10]. However, available tools and workflows require bioinformatic and computational knowledge and expertise. For example, tool parameters need to be adapted to the specific use case. End-to-end platforms (Table 1) that allow users to analyse their samples are either restricted with only a limited free trial (e.g. BugSeq [11]) or paid subscription (e.g. OneCodex [12]), or require high computational resources (e.g. SURPI [13] and Sunbeam [14]). For certain free resources, the underlying workflow is not available and adaptable for the user. For example, IDseq [15] (also known as CZID [16]), a free cloud-based service for pathogen detection can only be externally accessed through the dedicated online user interface. Furthermore, some of these workflows are specific to a certain host, pathogen, or sequencing technique, lacking the flexibility for customisation.

Galaxy [17] is an open-source platform for FAIR data analysis. It enables users to apply a comprehensive suite of bioinformatics tools (that can be combined into workflows) through either its user-friendly web interface or its automatable Application Programming Interface (API) for integrating and customising workflows, enhancing user flexibility. It ensures reproducibility by capturing the necessary information to repeat and understand data analyses. Galaxy offers a collection of high-quality pre-built workflows that can be either used directly or are easily adapted to the user's needs via the Galaxy workflow editor. Galaxy workflows can be executed on any Galaxy server, even on the private Galaxy server, making it suitable also for data where privacy concerns are important. Furthermore, Galaxy via the major public servers [17] freely provides a large computing infrastructure allowing for the execution of computationally challenging workflows, which is often the case for metagenomic analysis.

Here, we present PathoGFAIR, a collection of Galaxy-based workflows for pathogen identification and tracking its presence among (meta)genomics Oxford Nanopore sequencing data. The workflows are openly available on two workflow registries (Dockstore [18] and WorkflowHub [19]). They can be used directly on three major Galaxy servers (usegalaxy.org, usegalaxy.eu, usegalaxy.org.au) or installed in any other Galaxy server. The workflows are created to work agnostically, detecting all pathogens present in the samples without prior knowledge of the target

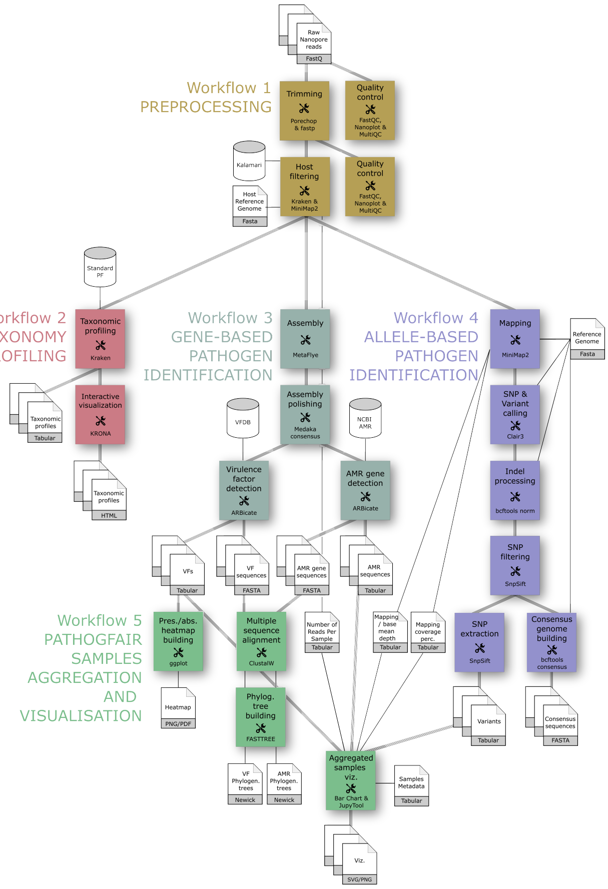


**Figure 1.** Flowchart of the PathoGFAIR workflows. Workflow 1 (olive green) takes as input sequencing data generated by Oxford Nanopore technologies and performs quality control and host filtering. Then three parallel workflows are executed on the output of Workflow 1: Workflow 2 (red) for taxonomy profiling, Workflow 3 (dark cyan) for gene-based pathogen identification, and Workflow 4 (purple) for SNP-based pathogen identification. These four workflows can run individually and in parallel. Finally, all outputs for the different provided datasets are aggregated in Workflow 5 (green) for PathoGFAIR Samples Aggregation and Visualisation.

pathogen. As the workflows are created in Galaxy, they can be adapted, e.g. for other sequencing techniques or with various downstream analyses, such as differential expression analysis, or further statistics and visualisations [17]. Workflows are documented and supported by an extensive tutorial freely available via the Galaxy Training Network (GTN) [20]. Overall, PathoGFAIR offers an easy-to-use computational solution that speeds up the process of sampling, detecting, and tracking pathogens. Links to workflows and tutorials can be found on PathoGFAIR homepage: https://usegalaxy-eu.github.io/PathoGFAIR/

## Implementation

### Overview

PathoGFAIR comprises a collection of 5 workflows, implemented in Galaxy (Figure 1). Each workflow serves a specific function and can be executed independently, enabling users to tailor their analysis according to their requirements.

The input data for PathoGFAIR comprises sequencing data generated using Oxford Nanopore technologies, along with an optional metadata table describing the datasets. Basecalling for converting raw signal data from the Nanopore sequencer into nucleotide sequences is not included within the PathoGFAIR workflows. In the use cases presented later in the manuscript, real-time basecalling is performed using the MinKNOW software (Oxford Nanopore Tech-

nologies) before the reads are used in the workflows. Basecalling is a crucial step, as it affects the quality of the reads. Users are encouraged to ensure that high-quality basecalling is performed before starting the analysis with PathoGFAIR.

The datasets are preprocessed in Workflow 1, which encompasses quality control and host removal procedures. Subsequently, the preprocessed data is directed to three parallel workflows: taxonomy profiling (Workflow 2), gene-based pathogen identification (Workflow 3), and allele-based pathogen identification (Workflow 4). This parallel execution allows for efficient analysis and flexibility in workflow selection. Notably, Workflow 4 can optionally synchronise with Workflow 2 or Workflow 3 to leverage prior taxonomic analysis or gene-based pathogen identification results, providing users with flexibility based on specific use cases. By using detailed taxonomic identification from Workflow 2 or gene-based pathogen identification from Workflow 3, Workflow 4 enhances mapping and SNP detection accuracy. This process involves selecting the correct reference genome of the pathogen for mapping, informed by results from Workflow 2, Workflow 3, or even Workflow 1, which performs initial taxonomy assignment during the host filtering step.

Since each workflow can be executed independently, users can focus on specific aspects of pathogen detection or analysis. This modular approach empowers users to utilise the full range of functions offered by each workflow individually or to combine them as needed for comprehensive pathogen detection.

Finally, in Workflow 5, outputs from the previous workflows and the metadata of the dataset are aggregated and visualised for comprehensive pathogen tracking across samples. This aggregation step ensures a holistic view of pathogen presence and distribution, facilitating further insights and analysis.

Overall, the independent nature of PathoGFAIR's workflows provides users with a user-friendly and customisable approach to pathogen detection, allowing for both comprehensive analyses and targeted investigations based on specific research needs or objectives.

Ensuring the accuracy and currency of reference data is indeed fundamental for robust metagenomic analysis. PathoGFAIR leverages Galaxy's integrated Data Managers, which enables Galaxy admins to provide up-to-date reference data. These Data Managers automate the download, installation, and regular update of essential reference databases, ensuring that PathoGFAIR users work with complete, accurate, and up-to-date reference information. PathoGFAIR workflows are configured to use well-maintained and reputable sources, such as NCBI and other public pathogen reference repositories, which further support accuracy and comprehensiveness. Additionally, Galaxy's user-friendly interface enables users to select preferred references or request the inclusion of specific databases via Galaxy administrators, adding to the workflow's adaptability for diverse use cases.

PathoGFAIR offers a competitive, and accessible solution (Table 1) to detect and track pathogens in metagenomic Nanopore data through its five Galaxy-based FAIR and customisable workflows.

## Workflow 1: Preprocessing

Workflow 1 encompasses essential preprocessing steps to ensure the quality and integrity of sequencing data.

Quality control and sequence filtering, based on quality, length, or low complexity, are performed using Fastp (v 0.23.2) [24]. Porechop (v 0.2.4) [25] trims low-quality base pairs and removes duplicates and adapters. Quality thresholds are set to ensure that reads have an average quality score of Q20, aligning with accepted standards for Nanopore sequencing, where Q20 or higher quality is typically sufficient for reliable results [26].

Quality-controlled (QC) reads are cleaned of sequences from the host or food source (e.g. bovine in case of bovine meat) by mapping to their reference genome using Minimap2 (v 2.26) (RRID:SCR_018550) [27], a tool tens of times faster than mainstream long-read mappers such as BLASR [28], BWA-MEM [29], NGMLR [30] and GMAP [31] and three times as fast as Bowtie2 [32] designed for Illumina short reads [27]. A variety of reference genomes (e.g. Human, Chicken, or Cow) can be installed on Galaxy servers to work with Minimap2. A wide variety of reference genomes are integrated into Minimap2 on Galaxy, providing users with a convenient selection to choose from before executing the workflow. Kraken2 (v 1.2) [33] is applied for further contamination detection e.g. human sequences using the Kalamari database. The Kalamari database includes mitochondrial sequences of various known hosts [34]. Host/food source reads matched to the Kalamari database are assessed and removed using Krakentools (v 1.2) [33].

The workflow returns QC reads without contamination or host sequences as well as interactive reports, produced by FastQC (v 0.12.1) (RRID:SCR_014583), fastp and MultiQC (v 1.11) (RRID:SCR_014982) [35]. Furthermore, Nanoplot (v 1.39.0) [36] is employed to provide detailed quality metrics specifically tailored to the preprocessing step, enriching the suite of analytical insights and facilitating robust data evaluation.

## Workflow 2: Taxonomy Profiling

Workflow 2 performs taxonomic profiling of the microbial community to identify pathogens and other microorganisms for the QC reads from Workflow 1, using Kraken2 (v 1.2) [33] and the PlusPF (archaea, bacteria, viral, plasmid, human, UniVec_Core, protozoa, fungi, and plant) Refseq database (June 7, 2022). Although Kraken2 is a tool designed for short-read sequencing and is known for its false positive taxonomy assignments, particularly at lower microbial abundances [37], its application to long-reads can still yield a substantial overview of the microbial community. This is particularly true for discerning bacteria that could potentially be pathogenic at genus and species taxonomic ranks [38, 39]. Kraken2 allows for the rapid assignment of taxonomy at multiple ranks, from kingdom to species, using an efficient exact k-mer matching algorithm. Other tools such as Centrifuge (RRID:SCR_016665) or MetaPhlAn (RRID:SCR_004915) are viable alternatives, also available on Galaxy. Kraken2 is selected for its speed, sensitivity, and ability to work with large reference databases, a critical factor when analysing complex metagenomic samples [33, 40]. The produced community profile is visualised using Krona (RRID:SCR_012785) [41] and observed interactively for different taxonomic ranks using Phinch [42] or Pavian [43].

## Workflow 3: Gene-based Pathogen Identification

In this workflow, the pathogens are identified by the presence of genes associated with pathogenicity. QC reads from Workflow 1 are assembled into contigs using Metaflye (v 2.9.1) (RRID:SCR_017016) [44]. The contigs are then polished using the Medaka Consensus Pipeline (v 1.7.2) [45], which generates consensus sequences using neural networks and shows improved accuracy over graph-based approaches for Oxford Nanopore reads. The polished contigs are afterwards screened using ABRicate (v 1.0.1) [46] for virulence factors (VF) with the Virulence Factor DataBase (VFDB) [47] and for antimicrobial resistance (AMR) genes with AMRFinderPlus [48] database. ABRicate is chosen for its versatility, as it supports multiple databases, including those for antimicrobial resistance genes and the Virulence Factor Database (VFDB). This makes it a comprehensive tool for gene-based pathogen detection, capable of identifying a wide range of relevant genetic markers [46].

**Table 1.** Comparison of features between PathoGFAIR and other similar pipelines or systems. This comparison sheds light on various features and characteristics, such as accessibility, technical specifications, and the scope of analyses offered by each system. It serves as a reference to evaluate the suitability of PathoGFAIR and other similar pipelines or systems for specific needs and requirements

| Features | PathoGFAIR | IDseq | BugSeq | SURPI | OneCodex | Sunbeam | Innuendo [21] | PAIPline [22] | Victors [23] |
|---|---|---|---|---|---|---|---|---|---|
| **General Characteristics** | | | | | | | | | |
| Free of Charge | ✓ | ✓ | ✗* | ✓ | ✗ | ✓ | ✓ | ✓ | ✓ |
| Open Source Code | ✓ | ✓ | ✗ | ✓ | ✗ | ✓ | ✓ | ✓ | ✗ |
| Web Interface | ✓ | ✓ | ✓ | ✗ | ✓ | ✗ | ✓ | ✗ | ✓** |
| Automatable API | ✓ | ✗ | ✗ | ✗ | ✗ | ✓ | ✓ | ✗ | ✗ |
| **Accessibility and Availability** | | | | | | | | | |
| Simple end-user Modification | ✓ | ✗ | ✗ | ✗ | ✗ | ✓ | ✗ | ✓ | ✗ |
| Publicly Available Web-server | ✓ | ✓ | ✓ | ✗ | ✓ | ✗ | ✗ | ✗ | ✓ |
| Last Updated | 2024 | 2024 | 2024 | 2014 | 2023 | 2024 | 2018 | 2018 | 2019 |
| **User Support and Documentation** | | | | | | | | | |
| Tutorial | ✓ | ✗ | ✗ | ✗ | ✗ | ✗ | ✗ | ✗ | ✗ |
| Documentation | ✓ | ✓ | ✓ | ✓ | ✓ | ✓ | ✓ | ✓ | ✓ |
| User support | ✓ | ✓ | ✓ | ✗ | ✓ | ✗ | ✗ | ✗ | ✗ |
| **Technical Specifications** | | | | | | | | | |
| Workflow Manager | Galaxy | - | - | - | - | Snakemake | Nextflow | - | - |
| Sequencing Technique | Nanopore*** | Illumina & Nanopore | Illumina & Nanopore | Illumina | - | Illumina | Illumina | Illumina | - |
| **Analyses** | | | | | | | | | |
| Preprocessing | ✓ | ✓ | ✓ | ✓ | ✓ | ✓ | ✗ | ✓ | ✗ |
| Taxonomy Profiling | ✓ | ✓ | ✓ | ✓ | ✓ | ✓ | ✗ | ✓ | ✗ |
| Gene-based Pathogen Identification | ✓ | ✓ | ✓ | ✓ | ✓ | ✓ | ✓ | ✓ | ✓ |
| Allele-based Pathogen Identification | ✓ | ✗ | ✓ | ✗ | ✗ | ✗ | ✓ | ✗ | ✓ |
| Samples aggregation and Visualisations | ✓ | ✓ | ✗ | ✗ | ✓ | ✓ | ✗ | ✗ | ✗ |

* Free trial of 10 samples is available.
** Malfunctioned when tested.
*** Can be easily adapted to any other types of sequencing techniques via Galaxy, a customisable and automatable API.

## Workflow 4: Allele-based Pathogen Identification

Another approach to identifying pathogens is to use an allelic approach by detecting SNPs, i.e. markers showing evolutionary histories of homogeneous strains [49]. This process includes SNP calling, aimed at identifying novel pathogen strains and elucidating discrepancies compared to reference sequences, thereby facilitating the tracing of emerging variants. Within Workflow 4, both complex variants and SNPs are discerned, serving as crucial elements for subsequent pathogen identification and variant tracing purposes.

QC reads from Workflow 1 are mapped using Minimap2 (v 2.26) to a selected reference genome of a suspected pathogen. Users can choose the reference genome based on their prior knowledge of the target pathogen, the taxonomic analysis in Workflow 2, or the detected pathogenic genes in Workflow 3. Variant calling for mapped reads is performed using Clair3 (v 0.1.12) [50]. Clair3, a tool developed for long reads, has been chosen because it is demonstrated to be faster and more accurate than the Medaka variant pipeline, which its developer has declared deprecated in favour of Clair3 [45]. After that, all complex variants and their information, such as type, genomics position, and quality score, are normalised using bcftools norm (v 1.9) [51]. The normalised reads are filtered using SnpSift filter (v 4.3) (RRID:SCR_015624) [52] based on the SNP quality computed in the SNPs identification step with Clair3. Filtered variants fields required for further analyses are extracted using SnpSift extract fields (v 4.3) (RRID:SCR_015624) [52]. Finally, a consensus sequence for each sample is built using bcftools consensus (v 1.9) (RRID:SCR_005227) [53]. In addition to the variants, this workflow outputs tables including summary metrics like the mapping coverage (breadth of coverage) percentages for every sample, per base covering mean depth (depth of covering), and quality filtered complex variants and SNPs numbers. For more accurate results, users should consider only SNPs with a minimum depth of covering of 10x to ensure reliable calls, as demonstrated in the analyses of the following Use Cases section. This threshold effectively minimises the inclusion of false-positive variants, a challenge often encountered with Nanopore sequencing data due to its inherent error rates.

## Workflow 5: PathoGFAIR Samples Aggregation and Visualisation

In all previously described workflows, individual samples are analysed separately. Workflow 5 consolidates the outputs from Workflows 1, 2, 3, and 4 along with sample metadata to generate various visualisations and reports. These reports illustrate the detected pathogens and facilitate the visualisation and tracking of their presence across all samples.

Virulence Factor (VF) tables from Workflow 3 are used to generate clustered heatmaps showing the VF genes using ggplot2 Heatmap (v 3.4.0) (RRID:SCR_014601). VF sequences are concatenated per sample, generating a consensus sequence of identified VF genes per sample, and aligned over all samples using ClustalW (v 2.1)(RRID:SCR_017277). A phylogenetic tree of the virulence gene sequences is then generated from the multiple sequence alignment using FASTTREE (v 2.1.10) (RRID:SCR_015501) [54] and visualised using Newick Display (v 1.6). The same is performed on the antimicrobial resistance (AMR) tables from Workflow 3. From Workflows 1 and 4 output tables, bar charts are generated.

Other outputs are aggregated and processed within a Jupyter Notebook [55], interactively launched in Galaxy using JupyTool (v 1.0.0). This Notebook showcases the integration of sample metadata to generate analysis-specific plots, leveraging Python (v 3.10.12) [56] libraries such as Pandas (v 1.5.3) [57, 58], Matplotlib (v 3.7.1) [59], Seaborn (v 0.12.2) [60], and Numpy (v 1.24.3) [61]. Examples of these plots include bar plots illustrating the number of reads before and after quality control for all samples, scatter plots visualising relationships between different variables such as pathogen count and sample characteristics, and interactive cluster maps displaying the clustering patterns of samples based on pathogen composition. These visualisation techniques are further elucidated and exemplified in the Use Cases section of this study, where the output tables from the workflows are aggregated with the corresponding sample metadata and visualised to facilitate comprehensive visual analysis.

Virulence factors (VF) and antimicrobial resistance (AMR) genes are often found on mobile genetic elements (MGEs) such as plasmids or phages, meaning they can sometimes appear independently of their bacterial hosts. To address this challenge, PathoGFAIR integrates taxonomic profiling from Workflow 2 with gene detection results from Workflow 3. This cross-referencing ensures accurate attribution of VF and AMR genes to their respective host organisms. For further validation, Workflow 4 enables users to map consensus genomes, generated in Workflow 5 from detected VF genes, against any reference genomes. This process confirms whether these VF or AMR genes, detected in Workflow 3, are genuinely linked to the bacterial genome or merely associated with MGEs, with additional coverage metrics helping to ensure accurate mapping. Future updates to PathoGFAIR will include expanded methodologies to validate gene-host associations using broader taxonomic markers, further refining the precision of pathogen characterisation..

## Workflow Reports

As all PathoGFAIR workflows are designed to run seamlessly on the Galaxy platform, an interactive report is automatically generated upon completion of each workflow. These reports provide a comprehensive overview of the respective workflow's inputs and outputs. In PathoGFAIR, special attention has been given to refining these reports for enhanced user experience. The reports are carefully curated to automatically showcase and emphasise only the most informative, easily interpretable, and accessible outputs for each workflow. This ensures that users can efficiently extract key insights from the results, facilitating a streamlined and user-friendly analysis experience.

## Easily Adaptable Workflows

The workflows can process raw shotgun (meta)genomics sequencing data from any sample, not only food.

PathoGFAIR has been initially developed to take Oxford Nanopore data as inputs. However, PathoGFAIR can work with Illumina data or other types of sequencing technique data. To adapt to Illumina sequencing only one tool needs to be changed in Workflow 1: Porechop [25] with Cutadapt (RRID:SCR_011841) [62]. Workflows 2, 3, 4, and 5 can be used directly with Illumina datasets without any adaptation. Some tools can be changed based on the tool's known performance towards short and long reads, such as Clair3 (v 0.1.12) [50] and Metaflye (v 2.9.1) [44]. All the mentioned tools are accessible within Galaxy, allowing for seamless interchangeability.

The workflows can also be adapted to process paired-end reads, by adjusting the tools' parameters to take paired-end read samples instead of single-end reads. These changes can be applied with little effort by using the user-friendly workflow editor in Galaxy.

Users can seamlessly switch between different host reference genomes and Kraken2 databases, as PathoGFAIR supports various pre-installed databases on the Galaxy servers. This feature enhances user convenience and efficiently explores different configurations to suit specific analysis requirements.

Similarly, tool versions and parameters can be adapted, e.g. to compare results with legacy versions of the workflows. New tool versions are automatically installed on public Galaxy servers using a sophisticated update infrastructure, ensuring a straightforward mechanism to keep the infrastructure up-to-date [63]. Every time

a tool is updated, an update of the workflows is suggested, tested with functional tests, and released on the workflow registries once accepted.

Each of the five PathoGFAIR workflows is designed for a distinct type of analysis. Workflows 2, 3, and 4 operate independently, offering the flexibility to run them concurrently or skip them as per user requirements. This modular structure allows users to tailor the analysis to their specific needs, activating only the functionalities necessary for the desired workflow outcome.

## FAIR Workflows

The FAIR principles [64], which emphasise the importance of making research objects Findable, Accessible, Interoperable, and Reusable, offer valuable guidance for optimising the utility and promoting the reproducibility and reusability of any research object (data, software [65], or workflows).

PathoGFAIR has been developed with the FAIR principle in mind and follows the ten tips for building FAIR workflows, as suggested by de Visser *et al.* [66]. First, by using Galaxy as a workflow manager, the workflows are portable (Tip 6) and come with a reproducible computational environment (Tip 7). The tools integrated into the workflows use file format standards such as FASTA and FASTQ for sequence data, SAM and BAM from the Samtools project for alignment data, VCF for genetic variations, GenBank and GFF3 for genomic annotations, and PDB for structural data (Tip 5) [64]. As explained in the previous section, the workflows are provided with default values (Tip 8) and are modular (Tip 9).

The workflows are available on the GitHub repository of IWC, the Intergalactic Workflow Commission of the Galaxy community (Tip 3) [67]. Workflows in this repository are reviewed and tested using test data before publication and with every new Galaxy release. The IWC automatically updates the workflows whenever a new version of any tool used in these workflows is released. Deposited workflows follow best practices, are versioned using GitHub releases, and contain important metadata (e.g. License, Author, Institutes) (Tip 2). The workflows are automatically added to two workflow repositories (Dockstore [18] and WorkflowHub [19]) to facilitate the discovery and re-use of workflows in an accessible and interoperable way (Tip 1). Via Dockstore or WorkflowHub, the PathoGFAIR workflows can be installed on any up-to-date Galaxy server. They are already publicly available on three main Galaxy servers (usegalaxy.org, usegalaxy.eu, usegalaxy.org.au), which any user can use and modify without restriction.

A thorough explanation of how to use the workflows in PathoGFAIR including a more global description of pathogen identification from Oxford Nanopore data can be found in a dedicated extensive tutorial [68] together with example input data and results (Tips 4 and 10), freely available and hosted via the Galaxy Training Network (GTN) [20] infrastructure.

Finally, for every invocation of the workflows, a Research Object Crate (RO-Crate [69, 70]) can be created to store the data products of the different steps, along with the run-associated metadata (including parameters, tool, and workflow version).

# Use Cases

To showcase PathoGFAIR and its capabilities, 130 samples from two distinct studies—one involving samples with prior pathogen isolation and the other without— were analysed. In the case of non-isolated samples, pathogens were deliberately spiked to mimic real-world scenarios. For isolated samples, prior identification ensured the pathogens' identities were known. All samples underwent sequencing using Oxford Nanopore technology, highlighting the workflow's adaptability across diverse sample preparation methods.All workflows of PathoGFAIR were evaluated for their main intended tasks, e.g., the preprocessing workflow for its reads quality retaining and hosts sequences removal performance, but also for their ability to identify the correct pathogen, and how well the accuracy with respect to different sampling conditions is.

## Samples Without Prior Pathogen Isolation

### *Data Generation*

In this study, 46 samples had been prepared given the following protocol [71]. Chicken meat was spiked with either one of three *Salmonella enterica* subspecies (*S. enterica* subsp. *houtenae* DSM 9221, *S. enterica* subsp. *enterica* DSM 554, or *S. enterica* subsp. *salamae* DSM 9220) or a mix of them, with concentrations that give Cycle Threshold (Ct) values between 25 and 33. A total of 15 samples were incubated at 37°C for 24 hours before DNA isolation to facilitate bacterial growth. All samples were after incubated at 56°C for 1 hour with lysis buffer and 20 $ng/\mu l$ Proteinase K, followed by DNA extraction according to the STAR BEADS Pathogen DNA/RNA Extraction kit (CYANAGEN SRL, Bologna, Italy) instructions. In this study, approximately 25 mg of meat were used per aliquot for DNA extraction. DNA concentrations were measured with the Qubit® 4.0 Fluorometer (Thermo Fisher Scientific) using the double-stranded DNA (dsDNA) High-Sensitivity (HS) assay kit (Thermo Fisher Scientific), following the manufacturer's protocol. The quality was evaluated with a Nanodrop® 1000 (Thermo Fisher Scientific), assessing the 260/280 nm and 260/230 nm ratios. 260/280 and 260/230 ratios were close to the expected ranges 1.8–2.0 and 2.0–2.2, respectively. Extracted DNA was barcoded before sequencing using the Native barcoding genomic DNA (with EXP-NBD104, EXP-NBD114, and SQK-LSK109) protocol (Oxford Nanopore). DNA was then loaded on an R9.4.1 MinION Mk flow cell (Oxford Nanopore). SpotON sample port cover and priming port were closed and sequencing was started. The sequencing device control, data acquisition, and real-time basecalling were carried out by the MinKNOW software of the MinION Mk1C device. For 6 samples, adaptive sampling, a technique used in Nanopore sequencing to selectively sequence microbial DNA while excluding unwanted host DNA (here chicken DNA), was used. Generated sequencing data is available via BioProject PRJNA982679. Metadata for the 46 samples is summarised in Supplementary Table T1 into five pieces of information: (i) expected subspecie(s), (ii) incubation before DNA isolation, (iii) adaptive sampling during sequencing, (iv) Colony-forming unit (CFU)/mL [72], a measure providing a quantitative assessment of viable microbial entities within a given sample and measured using standard microbiological techniques such as serial dilution and plating on agar medium, provides, (v) Cycle Threshold (Ct) values [73], values inversely proportional to the amount of nucleic acid present in the samples.

### *Preprocessing*

The number of reads after quality control varies significantly between samples (Figure 2 A), which impacts downstream analyses.

For host detection using Minimap2 (v 2.26), the option *PacBio/Oxford Nanopore read to reference mapping* was set here. As expected from the samples sequencing protocol (chicken samples and not isolated pathogen), most sequences were assigned to chicken (Gallus gallus galGal6): above 90% in 31 samples and between 55% to 85% for the remaining 15 samples (Supplementary Figure S1). However, the percentage of identified host DNA (between 60% and 98%) was not as low as expected for the 6 samples that had undergone adaptive sampling to exclude chicken DNA during sequencing. This shows that the adaptive sampling to exclude chicken in some samples during sequencing may not had removed all the chicken sequences. All sequences identified as chicken were removed (Figure 2 A). After QC and host removal, 19 samples had less than 1,000 reads. These samples could only be analysed using the taxonomy profiling as highlighted in the next sections.

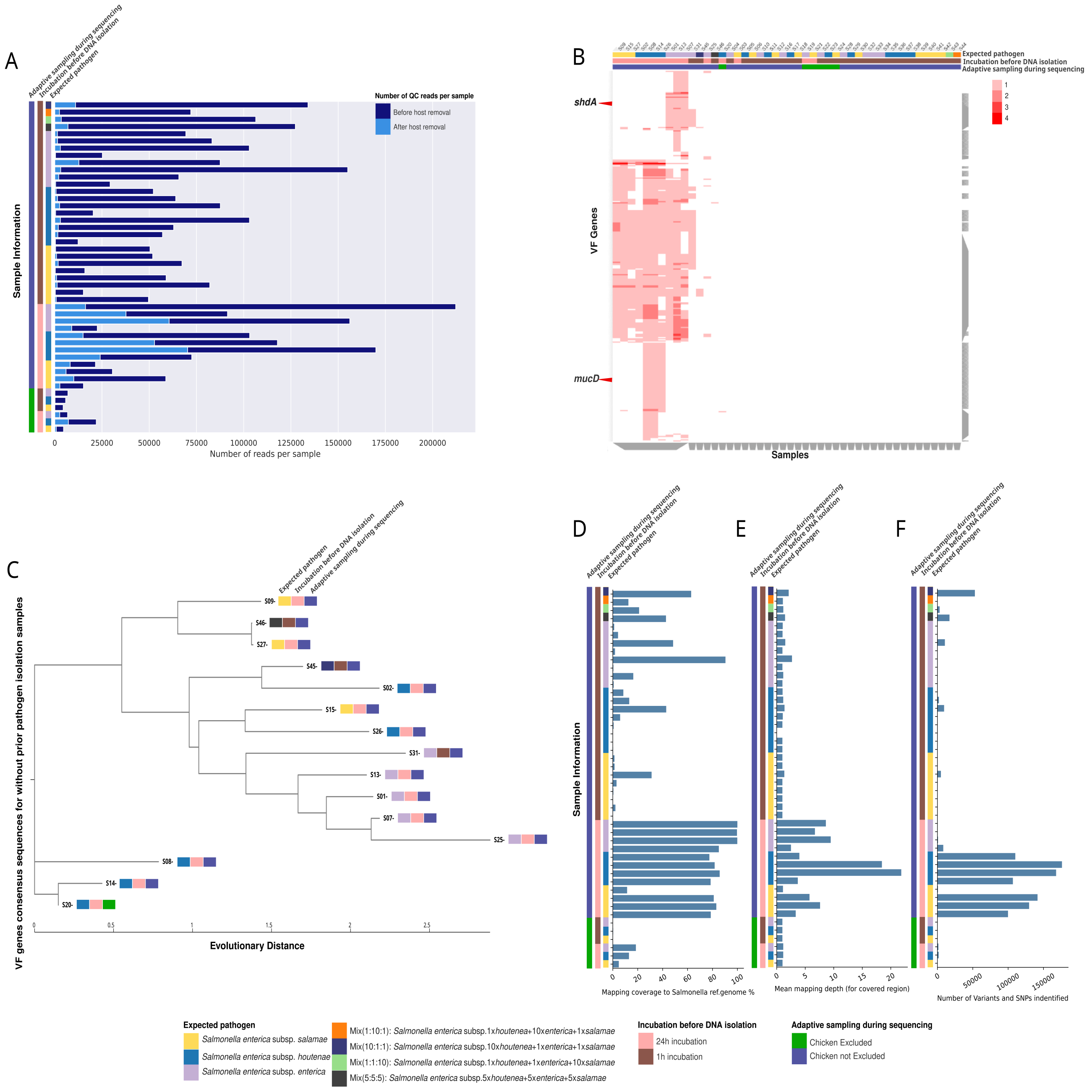


**Figure 2.** (**A**) Bar plot showing the total number of quality-controlled reads per sample before (dark blue) and after (light blue) host sequences removal. On the left, the metadata of the samples are displayed: (i) the expected *S. enterica* subsp. *salamae* in yellow, *S. enterica* subsp. *houtenae* in blue, and *S. enterica* subsp. *enterica* in light purple), (ii) incubation before DNA isolation (incubated for 24h in pink and incubated for 1h in brown), and (iii) adaptive sampling during sequencing (chicken excluded in green and chicken not excluded in purple)(**B**) Clustergram displaying the identified VF genes' abundances per sample. The VF genes are presented on the y-axis and all 46 non-isolated samples are on the x-axis along with their sample information. On the top are the metadata of the samples with the same color code as in **A** The grey bars on the bottom and on the right represent dendrogram VF gene (right) and samples' metadata (bottom) clusters found with hierarchical clustering with a clustering granularity of 0.5. (**C**) Phylogenetic tree, using the nucleotide evolution model; General Time Reversible (GTR) model with a CAT approximation for rate heterogeneity across sites [54]. The Phylogenetic tree was built on the VF genes consensus sequences concatenated per sample and aligned for all samples. (**D**) Bar plot with the mapping coverage (breadth of coverage), i.e. the percentage of covered bases of each sample to the reference genome, measured by calculating the percentage of positions within each bin with at least one base aligned against it. (**E**) Bar plot with the mean of the mapping depth (depth of coverage) of bases mapped to corresponding bases in the reference genome for every sample. (**F**) Bar plot with the number of variants and SNPs found per sample. Mapping coverage percentage and the depth mean indicate whether to trust the variants and SNPs found by the workflow or not, the higher the coverage percentage and the depth mean, the more trusted the SNPs results for the sample.

### *Taxonomy Profiling*

*S. enterica* was detected in Workflow 2 for all samples except one, at its species and different subspeciestaxonomic ranks (Interactive KRONA plot in Supplementary online Figure S2 and Supplementary Table T3).

### *Gene-based Pathogen Identification*

In Workflow 3, Metaflye (v 2.9.1) tool mode's option was chosen to be *Nanopore-HQ*, users can expand the workflow and change this option according to their datasets sequencing technique.

No contig was built for 10 of the 27 samples with less than 2,700 reads. The identification of Virulence Factors (VF) or AntiMicrobial Resistance (AMR) genes was then made impossible. For the other 17 samples, only 1 or 2 contigs were created, not enough for identifying VF and AMR genes.

For the remaining 19 samples with created contigs (from 3 to 157) and number of reads higher than 2,700, VF genes were identified in 15 samples (Figure 2 B), 12 of which were incubated before DNA isolation for 24 hours. 3 of the 15 samples were incubated for only 1 hour before DNA isolation, resulting in a few VF genes (Figure 2 B) identified, compared to the other 12 samples, mostly because of the low number of reads (Figure 3 E) from almost the absence of incubation (Figure 2 A). It was, for example, the case for the mixed samples, i.e. samples spiked with all 3 *S. enterica* subspecies, or samples spiked only with *S. enterica* subsp. *houtenae* and adaptively sampled during sequencing.

Some identified VF genes were found more than once in the same sample, with a maximum of 4 times. Common VF genes were identified for samples expecting identical *S. enterica* subspecies (Figure 2 B), such as the *mucD* gene, a serine protease *mucD* precursor, which was only found in *S. enterica* subsp. *houtenae* spiked samples, or *shdA*, an AIDA autotransporter-like protein, only found in *S. enterica* subsp. *enterica* spiked samples, but not in samples spiked with only *S. enterica* subsp. *houtenae* or *S. enterica* subsp. *salamae*.

Similar results were found for AMR genes (Supplementary Figure S3, 3 F). The sampling conditions affected the number of identified VF and AMR genes as shown by the relationships between the Ct value, CFU/mL value, or the number of remaining reads after preprocessing (Figure 3). The lower the Ct value, the higher the number of VF genes and AMR genes identified (Figure 3 A & B). No VF or AMR genes were detected for samples with Ct values above 26. For Ct values below 26, there was a negative correlation (Pearson $R = -0.85$, $p$-value $= 6 \times 10^{-05}$) between the Ct value and the number of identified AMR genes. Similar but inverse relations were observed for CFU/mL value (Figure 3 C & D), with a threshold for VF and AMR gene detection at $10^{6}$. VF and AMR genes were then detected if several conditions were fulfilled: a Ct value below 26, CFU/mL value above $10^{6}$, and at least 5,000 reads after preprocessing. The further the samples were from these thresholds, the higher the number of VF genes and AMR genes identified. Indeed, the three top scattered dots (in red - Figure 3 A, C & E), with identified VF genes between 250 and 300 were the samples with the highest number of reads, higher CFU/mL value, and a relatively lower Ct value compared to other samples. Generally, allowing samples to incubate for a short period before sequencing enhances microbial growth, resulting in higher CFU/mL values and lower Ct values. This increase in microbial concentration improves the efficiency of direct sequencing by providing more genetic material for analysis, facilitating faster and more accurate pathogen detection.

### *Allele-based Pathogen Identification*

In Workflow 4, samples were mapped against a reference genome of an expected pathogen chosen by the user. *S. enterica subsp. enterica* ser. Typhimurium (NC_003197.2) was chosen for this data, as it is widely recognised and extensively used in genomic studies due to its complete and well-annotated genome sequence [74]. However, given the diversity among the serovariants of *S. enterica* subsp. *enterica*, a high number of complex variants and SNPs are anticipated.

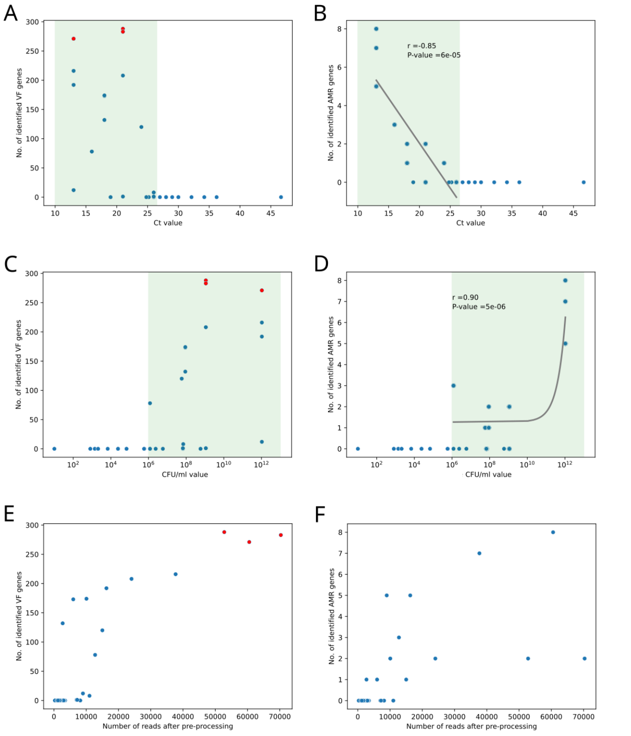


**Figure 3.** Scatterplots showing the number of identified VF genes (**A, C, E**) and AMR genes (**B, D, F**) in relationship to the Ct value (**A, B**), CFU/mL value (**C, D**), and the number of reads after preprocessing (**E, F**). The green area (**A, B, C, D**) highlights Ct values or CFU/mL values for which genes had been detected. Pearson correlation for values in the green area: (**A**) $R = -0.33$ and $p$-value $= 0.23$, (**B**) $R = -0.85$ and $p$-value $= 5.83e^{-05}$, (**C**) $R = 0.17$ and $p$-value $= 0.53$, (**D**) $R = 0.90$ and $p$-value $= 4.68e^{-06}$

The provided mapping statistics (mapping coverage (breadth of coverage) and mapping depth (depth of coverage) in Figure 2 D, E) serve as proxies for assessing the number and quality of identified SNPs (Figure 2 F). SNPs with low mapping depth are less reliable than those with higher depth. Reliable SNP calling typically requires a depth of at least 10, achieved in 2 samples. Samples with the highest mean mapping depth corresponded to samples with the highest number of reads after preprocessing (Figure 2 A). The higher the coverage and the mean mapping depth, the more quality SNPs were identified (Figure 2 D-F). Some of the samples spiked with *S. enterica* subsp. *enterica* had a high breadth of coverage but a low mean depth of coverage depth, as a result, the number of their quality filtered identified SNPs was low.

### *PathoGFAIR Samples Aggregation and Visualisation*

For the samples for which VF or AMR genes had been identified, phylogenetic trees were built on the concatenated genes consensus sequences (Figure 2 C for VF genes, Supplementary Figure S4 for AMR genes). These trees help track divergence between samples and could then highlight the contamination point or an evolution of the subspecies because of mutations. Indeed, samples spiked with *S. enterica* subsp. *enterica* were found together in the VFs-based tree (Figure 2 C), so the identified VF genes were unique to these samples and could clearly separate the samples from samples with other *S. enterica* subspecies. The samples spiked with *S. enterica* subsp. *houtenae* were mostly clustered together, except for 2 samples because of extra identified VF genes common with samples spiked with *S. enterica* subsp. *enterica* and/or *S. enterica* subsp. *salamae* . The 2 samples spiked with a mix of the 3 subspecies were found in the middle of the tree (Figure 2 C), showing that a mix

of VF genes related to the different subspecies was identified. The mixed sample, S45, spiked with a higher concentration of *S. enterica* subsp. *houtenae* than the other subspecies, was close to the sample, S02, spiked with *S. enterica* subsp. *houtenae* only. For AMR genes phylogenetic tree (Supplementary Figure S4), samples were not as clearly separated as the tree for VF genes, mostly because the number of identified AMR genes was relatively low compared to the number of identified VF genes.

#### *Sensitivity*

The performance of the workflows was evaluated based on their ability to identify the expected *S. enterica* pathogen, as well as *S. enterica* subspecies and strain taxonomic ranks for the tested samples (Supplementary Table T3). In a metagenomic setting, other detected species cannot be regarded as false positives, as they may naturally be present in the sample. Therefore, only sensitivity was reported.

For the taxonomy profiling (Workflow 2), the expected pathogen was detected at its species taxonomic rank in all but one sample, resulting in a sensitivity of 97.8%. At the subspecies taxonomic rank, the expected subspecies was detected in 28 out of 46 samples, yielding a sensitivity of 64.0%. To further evaluate subspecies classification performance, the sample-wise sensitivity (the percentage of correctly identified *S. enterica* subspecies out of all detected *S. enterica* subspecies) was calculated. Averaged across all samples, the sample-wise sensitivity was 47.3%. In the gene-based pathogen identification (Workflow 3), at least one virulence factor (VF) gene of the expected pathogen, at strain taxonomic rank, was detected in 13 out of 46 samples, corresponding to a sensitivity of 28.2%. For the samples in which no VF gene was detected, no contigs could be generated, preventing gene calling.

Changing the workflow's default settings, such as using different reference databases for preprocessing in Workflow 1, taxonomy profiling in Workflow 2, or gene-based pathogen identification in Workflow 3, would likely impact these metrics. Different reference databases could influence the accuracy and sensitivity of taxonomic classification and pathogen identification, as they may contain varying levels of strain-specific data. Adjusting parameters like threshold values, filtering criteria, or the inclusion of additional databases could also affect the detection sensitivity and overall performance, potentially improving or reducing the workflow's ability to accurately identify pathogens and associated genes in the given samples.

## Samples With Prior Pathogen Isolation

#### *Data Description*

To further test PathoGFair, 84 public datasets were used [75]. These samples were sampled in Palestine by the Swiss Tropical and Public Health Institute either from chicken meat, chicken stool, or human stool, in 2021 or 2022 (Supplementary Figure S5). In these samples, *S. enterica* had been isolated in 19 samples, and *Campylobacter jejuni* in 65 samples. The generated sequencing data are provided under BioProjects PRJNA942086 (S. enterica [76]) and PRJNA942088 (C. jejuni [77]).

#### *Preprocessing*

Negligible contamination or host sequences were found between 0% and 0.02% (Supplementary online Figure S6), as expected because of the prior isolation of the pathogen. The number of reads ranges between 3k and 217k reads per sample, after quality control.

#### *Taxonomy Profiling*

As presented in the interactive KRONA plot (Supplementary online Figure S6), the first 19 samples, *S. enterica* isolates, were assigned correctly to *S. enterica*, and the remaining 65 samples were assigned correctly to *C. jejuni*. With the KRONA plot (Supplementary online Figure S6), the total number of reads for each sample can be seen along with detailed percentages on the assigned taxa at each taxonomic rank.

#### *Gene-based Pathogen Identification*

In this workflow, we identified VF and AMR genes for all samples, thanks to the higher number of reads retained after preprocessing and the prior isolation of the pathogens. Consequently, VF genes were detected in all samples, with more VF genes identified than AMR genes (Supplementary Figure S7), as in general, the number of VF genes in a bacterial genome is often higher than the number of AMR genes. Samples containing *S. enterica* exhibited more VF genes (172 to 207) compared to samples with *C. jejuni* (96 to 113). The opposite trend was observed for AMR genes, *C. jejuni* samples typically had 12 AMR genes detected, while *S. enterica* samples mostly had 6 AMR genes (Supplementary Figure S7)

The analysis revealed that samples with similarly isolated pathogens clustered together based on detected VF genes (4). For example, samples with *S. enterica* and *C. jejuni* formed distinct clusters. Moreover, correlations were observed among samples from different hosts, sampling years, and pathogenic species.

Specific VF genes were found in samples with similar isolated pathogens, indicating potential subspecies-specific differences. For instance, Cj1419c, a methyltransferase Capsule biosynthesis and transport gene product, was exclusively found in *C. jejuni* isolates samples sequenced in 2022, while flgB gene, encoding flagellar basal body rod protein, was only detected in *C. jejuni* isolates samples sequenced in 2021. *flaA* (flagellin), a VF gene product identifying *C. jejuni*, was present in *S. enterica* isolates samples from human stool sampled in 2022 and all *C. jejuni* isolates samples, but not in *S. enterica* isolates samples from chicken meat, chicken stool, or human stool sampled in 2021.

Furthermore, certain VF genes such as *spvC* (type III secretion system effector *SpvC* phosphothreonine lyase) and pefB (plasmid-encoded fimbriae regulatory protein), associated with *S. enterica subsp. enterica* ser. Typhimurium str. LT2, were exclusively found in *S. enterica* isolates samples from human stool sampled in 2022. Conversely, *fyuA*, a pesticin/yersiniabactin receptor protein that identifies *Yersinia pestis*, was detected in every *S. enterica* isolates sample except those from human stool sampled in 2022. Finally, some VF genes, like *flif*, a flagellar M-ring protein known in *Yersinia enterocolitica* subsp. *enterocolitica*, were found in all samples, irrespective of the pathogen species.

#### *Allele-based Pathogen Identification*

The 19 *S. enterica* isolates samples were mapped against the reference genome of the expected pathogen, *S. enterica subsp. enterica* ser. Typhimurium (NC_003197.2 [74]), and the 65 *C. jejuni* isolates samples were mapped against *C. jejuni* (NC_002163.1).

The 19 *S. enterica* isolates samples have an average mapping coverage of 94.6% and an average mean mapping depth of 31 per base. The average total number of variants found per *S. enterica* isolates sample was 43,420. For the 65 *C. jejuni* isolates samples, the average mapping coverage was 93.7%, the average mean mapping depth was 42 per base and the average total number of variants found per sample was 26,654. These high values for the average total number of variants identified for samples were expected since the used subspecies for the mapping are different than the subspecies of the samples.

#### *PathoGFAIR Samples Aggregation and Visualisation*

The isolated samples exhibited a higher count of identified AMR genes compared to the metagenomic samples without prior isolation, enabling the incorporation of additional genes into concatenated gene consensus sequences. The resulting phylogenetic tree, constructed based on the AMR genes (Supplementary Figure S8), distinctly delineated different *S. enterica* subspecies . Similarly, this differentiation is evident in the phylogenetic tree based on the VF

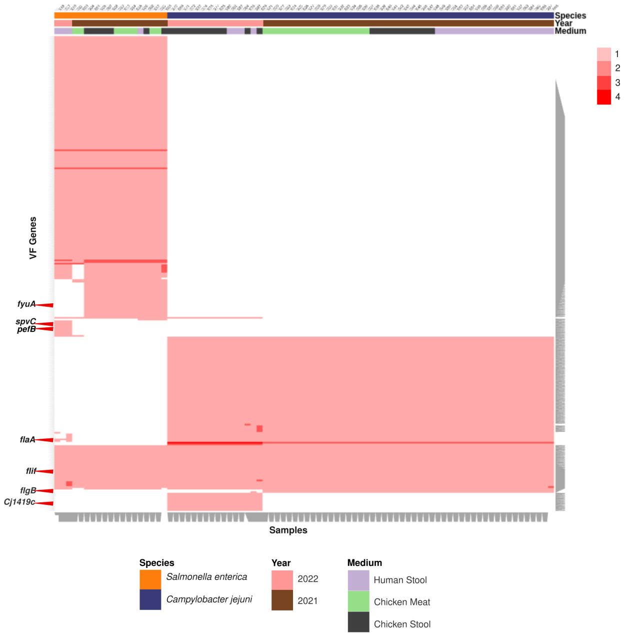


**Figure 4.** Cluster-map showing the identified VF genes on the y-axis for tested samples presented on the x-axis, clustered based on sample information such as sampling year, isolated pathogen species, and the original host of the sample. Clustering was performed using hierarchical clustering implemented in the Clustergrammer Python package

genes.

***Sensitivity***

Known species were successfully identified and confirmed across all workflows, including taxonomy profiling and VF genes identification (Supplementary Online Figure S6), achieving 100% expected pathogen detection at the expected taxonomic rank for all 84 tested samples resulting in a sensitivity of 1. Since there were no true negatives (TN) or conditions without pathogens in the dataset, specificity could not be calculated.

## Benchmarking PathoGFAIR

To evaluate the effectiveness of PathoGFAIR workflows, a benchmarking analysis was performed comparing PathoGFAIR's pathogen detection capabilities with the systems and pipelines listed in Table 1. The primary goal was to assess each pipeline's ability to accurately detect and identify pathogens from shotgun Nanopore metagenomic data using the samples without prior pathogen isolation. The detailed procedures for the selection and benchmarking process can be found on Protocols.io [78]. Replication instructions, including all benchmark systems, sample metadata, notebooks, and results, are publicly available through the PathoGFAIR GitHub repository.

### Selection of the pipelines for the benchmarking

To identify suitable systems and pipelines for the benchmark, each pipeline in Table 1 was evaluated based on availability, accepted input sequencing technique compatibility, and pathogen identification capability (Supplementary Table T4). Pipelines were classified as "Free Access" if available at no cost, "Free Trial" if partially or temporarily accessible, "Paid Access Only" if requiring payment, or "Non-functional" if outdated or inoperative. Compatibility with single-end Nanopore metagenomic sequencing data was verified to ensure each pipeline's applicability for pathogen-focused workflows. Each pipeline's pathogen identification capabilities were also assessed, examining relevant algorithms and tools to determine their accuracy and sensitivity within metagenomic datasets.

Following these criteria, pipelines requiring paid access (e.g. OneCodex) or being non-functional, as well as those incompatible with Nanopore data (e.g., SURPI, Sunbeam, Innuendo, and PAIPline) or lacking robust pathogen identification features, were excluded from further analysis.

Each selected pipeline was subsequently set up and tested to confirm ease of use and reproducibility. During testing, Victors was found to be non-functional, and SURPI presented difficulties, requiring local downloads of large host and pathogen reference databases. SURPI's fragmented documentation contained outdated and contradictory requirements, with no updates since 2014. Innuendo, though documented, also presented usability challenges with incomplete setup instructions. BugSeq, while user-friendly and accessible through its web interface, had an average processing time of four hours from sample upload to results delivery. However, its free trial is limited to just 10 samples, and it lacked transparency regarding the tools, workflows, and databases used for analysis, as it is neither open-source nor adaptable. Ultimately, only IDseq (CZID) and our workflows, PathoGFAIR, met the selection criteria and were included for benchmarking (Supplementary Table T4).

IDseq offers a highly user-friendly interface, similar to PathoGFAIR on the Galaxy platform. However, unlike PathoGFAIR, IDseq's pipeline is limited in terms of adaptability to its workflows, particularly with respect to the tools and parameters used. This restriction can hinder customisation and flexibility for users with specific analytical needs, reducing the pipeline's versatility compared to PathoGFAIR. The IDseq pipeline took one hour for sample uploads and an additional hour and a half to complete the analysis across all samples.

### Benchmarking on samples without prior pathogen isolation

The benchmark was conducted using the 46 samples without prior pathogen isolation, from the first use case. The samples are chicken meat spiked with pathogens and sequenced using Nanopore as explained in [71]. Metadata for the 46 samples is summarised in Supplementary Table T1, including the expected *S. enterica* subspecies (*S. enterica* subsp. *houtenae* DSM 9221, *S. enterica* subsp. *enterica* DSM 554, and/or *S. enterica* subsp. *salamae* DSM 9220). For PathoGFAIR, taxonomy profiling from Workflow 2 and VF gene identification from Workflow 3 were evaluated.

In both PathoGFAIR and IDseq (Supplementary Table T3), *Salmonella enterica* was detected in all samples except one, resulting in a 97.8% detection rate. In the exceptional sample, *Salmonella* was not detected by either system. PathoGFAIR provided additional resolution, identifying the subspecies taxonomic rank in 60.9% of samples and the strain taxonomic rank in 13% of samples. In contrast, IDseq's output report did not provide pathogen information beyond the species rank (Supplementary Figure S9).

Further benchmarking with diverse public metagenomic datasets could offer more comprehensive insights into the performance of PathoGFAIR across different experimental conditions and sample types. Such analyses would help assess the workflow's robustness and adaptability to a wider range of use cases, contributing to its ongoing validation and refinement.

## Conclusion

In conclusion, we present PathoGFAIR, a collection of Galaxy FAIR adaptable workflows, designed for pathogen detection and tracking among samples. These five workflows span the entire analysis pipeline, ranging from preprocessing reads to advanced analyses including taxonomy profiling, virulence and antimicrobial resis-

tance gene identification, SNP detection, and evolutionary history comparisons. The workflows generate diverse visualisations for a comprehensive understanding of the results, accompanied by interactive reports detailing all relevant inputs and outputs.

Our workflows have successfully identified pathogens down to genus, species, or subspecies taxonomic ranks across diverse samples, surpassing limitations observed in comparable pipelines. Our workflows facilitate comprehensive sample comparisons across diverse types, conditions, and sequencing techniques by offering interpretative and publication-ready visualisations. The open-access and user-friendly design of PathoGFAIR mitigates accessibility challenges and reduces reliance on local computational resources by leveraging Galaxy's infrastructure for computational tasks, a feature that sets it apart from similar pipelines. This scalable workflow is a versatile solution for processing (meta)genomic samples, extending its utility beyond detecting foodborne pathogens.

In our findings, optimising sampling, preparation, and sequencing conditions, such as a 24-hour sample incubation, significantly enhances the identification of virulence and antimicrobial resistance genes. Indeed, the workflows' performance correlates with sample characteristics, with higher CFU/mL values and read counts, and lower Ct values yielding more comprehensive results, which can be used to establish sampling guidelines. Moreover, as the Pre-processing workflow effectively removes host sequences, adaptive sampling during sequencing to exclude host DNA is not necessary. The workflows were still able to detect pathogens at least at species taxonomic rank for samples without prior pathogen isolation.

The experimental setup for this study serves as a proof of concept, demonstrating the feasibility of using WGS for the detection and characterisation of *S. enterica* in spiked food matrices. By detecting pathogens across varying CFU/mL levels, as shown in the spiking experiments, the workflows showcase their sensitivity and practical applicability in adhering to stringent regulatory standards. These results establish a solid foundation for applying PathoGFAIR in food safety laboratories and outbreak investigations, where detailed subspecies-rank information is critical for monitoring and traceability.

PathoGFAIR's utility extends to enrichment-based analyses of foodborne pathogens, aligning with EU food safety standards such as EN/ISO 6579. Traditionally, these standards require enrichment followed by classical microbiological methods or PCR confirmation. PathoGFAIR complements these approaches by enabling direct analysis of enrichment broths through WGS, facilitating efficient detection and subspecies characterisation of *S. enterica*. This capability enhances food safety monitoring and outbreak response by differentiating *S. enterica* subspecies from previous outbreak strains, thereby advancing traceability and improving public health interventions.

We further supported the scientific community by introducing new 46 benchmark samples, making them publicly available. This demonstrates our significant investment of time and resources, providing valuable assets for future research.

In addition to the allele-based pathogen identification method, our workflow can be further enhanced by incorporating MLST. MLST, or Multi-Locus Sequence Typing, offers an alternative approach by characterising isolates through the sequences of housekeeping genes [49]. This method provides valuable information about the genetic diversity and evolutionary relationships among isolates, allowing for more precise identification and tracing of pathogens. By integrating MLST using MLST (v 2.22.0) tool [79]into our workflow, users can benefit from a comprehensive analysis that combines both alleles and variants identification methods, providing a more robust and accurate pathogen detection and tracing solution.

To address the complexity of detecting virulence and AMR genes located on mobile genetic elements (MGEs), future versions of PathoGFAIR can incorporate additional validation steps. Specifically, virulence and AMR gene detection could be cross-referenced with broader taxonomic markers (e.g., 16S rRNA) to ensure genes detected from MGEs are correctly attributed to their respective pathogenic hosts. These enhancements aim to further improve the accuracy of pathogen detection by more reliably linking virulence and AMR genes to their bacterial hosts, thereby refining the overall precision of the workflows.

In the future, integrating PathoGFAIR with Galaxy's automated bot system holds the promise of ongoing updates and analyses requiring minimal human involvement. By establishing a dedicated bot for PathoGFAIR, continuous results will be effortlessly refreshed whenever new datasets are uploaded, similar to the Galaxy bot created for SARS-CoV-2 [80]. The Galaxy bot for SARS-CoV-2 automatically updates and reanalyses data with each new upload, maintaining up-to-date results and reducing the need for manual intervention. This automation ensures real-time, efficient data processing and analysis, enhancing the workflow's accuracy and timeliness. Leveraging the user-friendly interface of the Galaxy platform ensures accessibility for users of all computational skill levels, streamlining the entire process from sample upload to result interpretation with ease. This study not only presents a robust computational solution but also lays the groundwork for semi-automated, efficient, and user-friendly pathogen detection and tracking workflows.

## Additional Files

Supplementary Figure S1. Violin plot with the percentage of quality-controlled host reads detected and removed in samples with respect to adaptive sampling during sequencing (Host excluded or not) - Samples Without Prior Pathogen Isolation

Supplementary online Figure S2. Krone Pie Chart for the taxonomy profiling - Samples Without Prior Pathogen Isolation

Supplementary Figure S3. Bar chart for the total number of VF genes (orange) and AMR genes (blue) found in samples with respect to incubation duration before DNA isolation - Samples Without Prior Pathogen Isolation

Supplementary Figure S4. Phylogenetic tree, using the nucleotide evolution model; General Time Reversible (GTR) model with a CAT approximation for rate heterogeneity across sites [54], for the identified AMR genes - Samples Without Prior Pathogen Isolation

Supplementary Figure S5. Upset plot illustrating the intersections of different metadata categories, including sampling year, pathogen species, and the original host of the samples, highlighting common and unique attributes among the datasets - Samples With Prior Pathogen Isolation

Supplementary online Figure S6. Krona Pie Chart for the taxonomy profiling - Samples With Prior Pathogen Isolation

Supplementary Figure S7. Violin plot for the total number of VFs and AMR genes - Samples With Prior Pathogen Isolation

Supplementary Figure S8. Phylogenetic tree, using the nucleotide evolution model; General Time Reversible (GTR) model with a CAT approximation for rate heterogeneity across sites [54], for the identified AMR genes - Samples With Prior Pathogen Isolation

Supplementary Figure S9. Heatmap with PathoGFAIR benchmark using the first Use Case datasets and compare it with similar analysis systems presented in Table 1

Supplementary Table T1. Metadata for Samples Without Prior Pathogen Isolation

Supplementary Table T2. Metadata for Samples With Prior Pathogen Isolation

Supplementary Table T3. PathoGFAIR and IDSeq pathogen detection results for Samples Without Prior Isolation at various taxonomic ranks

Supplementary Table T4. Benchmarking PathoGFAIR systems and pipelines evaluation

## Availability of Source Code and Requirements

Lists the following:

- Project name: PathoGFAIR https://usegalaxy-eu.github.io/PathoGFAIR/
- Workflows on public Galaxy servers: https://training.galaxyproject.org/training-material/workflows/embed.html?query=pathogfair
- Workflows (v 0.1) on WorkflowHub: https://workflowhub.eu/search?utf8=%E2%9C%93&q=pathogfair
- Workflows (v 0.1) on Dockstore: https://dockstore.org/search?organization=iwc-workflows&entryType=workflows&search=engy
- Tutorial: https://training.galaxyproject.org/training-material/topics/microbiome/tutorials/pathogen-detection-from-nanopore-foodborne-data/tutorial.html
- Data analysis home page: https://github.com/usegalaxy-eu/PathoGFAIR
- Operating system(s): Platform independent
- Other requirements: Account on a Galaxy server
- License: MIT license

## Data Availability

The raw sequence reads of the 46 samples without prior isolation are available on Sequence Read Archive (SRA) under BioProjects [81]. The protocol for the preparation of these samples is available on Protocols.io [71]. The workflows presented in the Methods section are available on Intergalactic Workflow Commission (IWC) and two workflow registries (Dockstore and WorkflowHub). The training material to understand, learn, and try the workflows is available on the Galaxy Training Network (GTN) [68]. The Jupyter notebook for additional visualisations and generating the figures of this paper is available in a GitHub repository [82]. Benchmarking PathoGFAIR protocol is available on protocols.io [78].

## Declarations

### List of Abbreviations

AMR: Antimicrobial resistance; API: Application Programming Interface; CFU: Colony-forming unit; Ct: Cycle Threshold; EFSA: European Food Safety Authority; EU: European Union; FAIR: Findable Accessible Interoperable Resulable; GTN: Galaxy Training Network; IWC: Intergalactic Workflow Commission; MLST: Multilocus sequence typing; NGS: Next-Generation Sequencing; QC: Quality Control; RKI: Robert Koch Institute; SNP: Single-nucleotide polymorphism; SRA: Sequence Read Archive; VF: Virulence Factor; VFDB: Virulence Factor database; WHO: World Health Organization; WGS: Whole Genome Sequencing.

### Funding

This research was supported by the Digital Life Science Call for Academia-Industry Collaborations under EOSC-Life funding [83]. Additionally, financial support was provided by the German Federal Ministry of Education and Research BMBF grant 031 A538A de.NBI-RBC and the Ministry of Science, Research and the Arts Baden-Württemberg (MWK) within the framework of LIBIS/de.NBI Freiburg. This work was supported by the Programme d'Investissements d'Avenir (PIA), grant Agence Nationale de la Recherche, number ANR-11-INBS-0013.

### Author's Contributions

Engy Nasr (E.N.) led the formal analysis, investigation, software development, validation, visualisation, and writing of both the original draft and the review and editing phases. E.N. also designed the workflows, tested them, created training materials, documented and published datasets, workflows, codes, protocols, and wrote the manuscript. Anna Henger (A.H.) supported conceptualisation, formal analysis, funding acquisition, investigation, project administration, validation, visualisation, and writing. A.H. also prepared and sequenced all project datasets, ensuring alignment of analysis results with lab preparation conditions. Björn Grüning (B.G.) supported conceptualisation, formal analysis, funding acquisition, investigation, project administration, and software development. B.G. integrated new databases into analytical tools and maintained Galaxy tools used in PathoGFAIR. Paul Zierep (P.Z.) contributed to formal analysis, investigation, software development, supervision, validation, visualisation, and writing. P.Z. supervised the project and edited parts of the workflows and training materials. Bérénice Batut (B.B.) led the conceptualisation, funding acquisition, methodology, project administration, software development, and supervision. B.B. applied for the EOSC Life Industry Call Grant, designed the project's main goals and guidelines, and managed and edited the full project. All authors read and approved the final manuscript.

## Acknowledgements

This research is made possible by the invaluable support of the entire Freiburg Galaxy team, Bioinformatics, University of Freiburg. The authors extend their special thanks to Wolfgang Maier and Mina Ansari for their technical expertise and academic guidance. We also appreciate the contributions of all the researchers who provided input to the project, with particular gratitude to Tobias Schindler for his exceptional assistance and to Peter van Heusden for his insightful contributions.

## References

1. Elbehiry A, Abalkhail A, Marzouk E, Elmanssury AE, Almuzaini AM, Alfheeaid H, et al. An Overview of the Public Health Challenges in Diagnosing and Controlling Human Foodborne Pathogens. Vaccines 2023 Mar;11(4):725. https://www.mdpi.com/2076-393X/11/4/725.
2. Wei X, Zhao X. Advances in typing and identification of foodborne pathogens. Current Opinion in Food Science 2021 Feb;37:52–57. https://linkinghub.elsevier.com/retrieve/pii/S2214799320300692, doi:10.1016/j.cofs.2020.09.002.
3. Organization WH. WHO global strategy for food safety 2022–2030: towards stronger food safety systems and global cooperation: executive summary. World Health Organization; 2022. https://www.who.int/publications/i/item/9789240057685, accessed 24 June 2024.
4. Priyanka B, Patil RK, Dwarakanath S. A review on detection methods used for foodborne pathogens. Indian Journal of Medical Research 2016;144(3):327. http://www.ijmr.org.in/text.asp?2016/144/3/327/198677.
5. Yang S, Johnson MA, Hansen MA, Bush E, Li S, Vinatzer BA. Metagenomic sequencing for detection and identification of the boxwood blight pathogen Calonectria pseudonaviculata. Scientific Reports 2022 Jan;12(1):1399. https://www.nature.com/articles/s41598-022-05381-x.
6. Thomas T, Gilbert J, Meyer F. Metagenomics - a guide from sampling to data analysis. Microbial Informatics and Experimentation 2012 Dec;2(1):3. https://microbialinformaticsj.biomedcentral.com/articles/10.1186/2042-5783-2-3.

7. Thompson LR, Sanders JG, McDonald D, Amir A, Ladau J, Locey KJ, et al. A communal catalogue reveals Earth's multiscale microbial diversity. Nature 2017 Nov;551(7681):457–463. https://www.nature.com/articles/nature24621.
8. Bogaerts B, Van den Bossche A, Verhaegen B, Delbrassinne L, Mattheus W, Nouws S, et al. Closing the gap: Oxford Nanopore Technologies R10 sequencing allows comparable results to Illumina sequencing for SNP-based outbreak investigation of bacterial pathogens. Journal of Clinical Microbiology 2024 Mar;0(0):e01576–23. https://journals.asm.org/doi/10.1128/jcm.01576-23, doi:10.1128/jcm.01576-23.
9. Joensen KG, Scheutz F, Lund O, Hasman H, Kaas RS, Nielsen EM, et al. Real-Time Whole-Genome Sequencing for Routine Typing, Surveillance, and Outbreak Detection of Verotoxigenic Escherichia coli. Journal of Clinical Microbiology 2014 May;52(5):1501–1510. https://journals.asm.org/doi/10.1128/JCM.03617-13, doi:10.1128/JCM.03617-13.
10. Allard MW, Bell R, Ferreira CM, Gonzalez-Escalona N, Hoffmann M, Muruvanda T, et al. Genomics of foodborne pathogens for microbial food safety. Current Opinion in Biotechnology 2018 Feb;49:224–229. https://linkinghub.elsevier.com/retrieve/pii/S0958166917301398, doi:10.1016/j.copbio.2017.11.002.
11. Fan J, Huang S, Chorlton SD. BugSeq: a highly accurate cloud platform for long-read metagenomic analyses. BMC Bioinformatics 2021 Dec;22(1):160. https://bmcbioinformatics.biomedcentral.com/articles/10.1186/s12859-021-04089-5.
12. One Codex | A fast, easy-to-use platform for microbiome sequencing and analysis;, accessed 24 June 2024.
13. Naccache SN, Federman S, Veeraraghavan N, Zaharia M, Lee D, Samayoa E, et al. A cloud-compatible bioinformatics pipeline for ultrarapid pathogen identification from next-generation sequencing of clinical samples. Genome Research 2014 Jul;24(7):1180–1192. http://genome.cshlp.org/lookup/doi/10.1101/gr.171934.113, doi:10.1101/gr.171934.113.
14. Clarke EL, Taylor LJ, Zhao C, Connell A, Lee JJ, Fett B, et al. Sunbeam: an extensible pipeline for analyzing metagenomic sequencing experiments. Microbiome 2019 Dec;7(1):46. https://microbiomejournal.biomedcentral.com/articles/10.1186/s40168-019-0658-x, doi:10.1186/s40168-019-0658-x.
15. Kalantar KL, Carvalho T, de Bourcy CFA, Dimitrov B, Dingle G, Egger R, et al. IDseq—An open source cloud-based pipeline and analysis service for metagenomic pathogen detection and monitoring. GigaScience 2020 Oct;9(10):giaa111. https://academic.oup.com/gigascience/article/doi/10.1093/gigascience/giaa111/5918865, doi:10.1093/gigascience/giaa111.
16. Chan Zuckerberg ID - Detect & Track Infectious Diseases;, accessed 24 June 2024.
17. The Galaxy Community, Afgan E, Nekrutenko A, Grüning BA, Blankenberg D, Goecks J, et al. The Galaxy platform for accessible, reproducible and collaborative biomedical analyses: 2022 update. Nucleic Acids Research 2022 Jul;50(W1):W345–W351. https://academic.oup.com/nar/article/50/W1/W345/6572001, doi:10.1093/nar/gkac247.
18. Yuen D, Cabansay L, Duncan A, Luu G, Hogue G, Overbeck C, et al. The Dockstore: enhancing a community platform for sharing reproducible and accessible computational protocols. Nucleic Acids Research 2021 Jul;49(W1):W624–W632. https://doi.org/10.1093/nar/gkab346.
19. Implementing FAIR Digital Objects in the EOSC-Life Workflow Collaboratory;, https://zenodo.org/records/4605654, doi:10.5281/zenodo.4605654. Accessed 25 June 2024.
20. Hiltemann S, Rasche H, Gladman S, Hotz HR, Larivière D, Blankenberg D, et al. Galaxy Training: A powerful framework for teaching! PLOS Computational Biology 2023 Jan;19(1):e1010752. https://dx.plos.org/10.1371/journal.pcbi.1010752, doi:10.1371/journal.pcbi.1010752.
21. Llarena A, Ribeiro-Gonçalves BF, Nuno Silva D, Halkilahti J, Machado MP, Da Silva MS, et al. INNUENDO: A cross-sectoral platform for the integration of genomics in the surveillance of food-borne pathogens. EFSA Supporting Publications 2018 Nov;15(11). https://data.europa.eu/doi/10.2903/sp.efsa.2018.EN-1498, doi:10.2903/sp.efsa.2018.EN-1498.
22. Andrusch A, Dabrowski PW, Klenner J, Tausch SH, Kohl C, Osman AA, et al. PAIPline: pathogen identification in metagenomic and clinical next generation sequencing samples. Bioinformatics 2018 Sep;34(17):i715–i721. https://academic.oup.com/bioinformatics/article/34/17/i715/5093217, doi:10.1093/bioinformatics/bty595.
23. Sayers S, Li L, Ong E, Deng S, Fu G, Lin Y, et al. Victors: a web-based knowledge base of virulence factors in human and animal pathogens. Nucleic Acids Research 2019 Jan;47(D1):D693–D700. https://dx.doi.org/10.1093/nar/gky999, doi:10.1093/nar/gky999.
24. Chen S, Zhou Y, Chen Y, Gu J. fastp: an ultra-fast all-in-one FASTQ preprocessor. Bioinformatics 2018 Sep;34(17):i884–i890. https://academic.oup.com/bioinformatics/article/34/17/i884/5093234, doi:10.1093/bioinformatics/bty560.
25. Porechop — de.NBI Nanopore Training Course latest documentation;, https://denbi-nanopore-training-course.readthedocs.io/en/latest/read_qc/Porechop_1.html, accessed 24 June 2024.
26. Wagner GE, Dabernig-Heinz J, Lipp M, Cabal A, Simantzik J, Kohl M, et al. Real-Time Nanopore Q20+ Sequencing Enables Extremely Fast and Accurate Core Genome MLST Typing and Democratizes Access to High-Resolution Bacterial Pathogen Surveillance. Journal of Clinical Microbiology 2023 Apr;61(4):e01631–22. https://journals.asm.org/doi/10.1128/jcm.01631-22.
27. Li H. Minimap2: pairwise alignment for nucleotide sequences. Bioinformatics 2018 Sep;34(18):3094–3100. https://academic.oup.com/bioinformatics/article/34/18/3094/4994778, doi:10.1093/bioinformatics/bty191.
28. Chaisson MJ, Tesler G. Mapping single molecule sequencing reads using basic local alignment with successive refinement (BLASR): application and theory. BMC Bioinformatics 2012 Dec;13(1):238. https://bmcbioinformatics.biomedcentral.com/articles/10.1186/1471-2105-13-238, doi:10.1186/1471-2105-13-238.
29. Li H, Durbin R. Fast and accurate short read alignment with Burrows–Wheeler transform. Bioinformatics 2009 Jul;25(14):1754–1760. https://academic.oup.com/bioinformatics/article/25/14/1754/225615, doi:10.1093/bioinformatics/btp324.
30. Sedlazeck FJ, Rescheneder P, Smolka M, Fang H, Nattestad M, Von Haeseler A, et al. Accurate detection of complex structural variations using single-molecule sequencing. Nature Methods 2018 Jun;15(6):461–468. https://www.nature.com/articles/s41592-018-0001-7, doi:10.1038/s41592-018-0001-7.
31. Wu TD, Watanabe CK. GMAP: a genomic mapping and alignment program for mRNA and EST sequences. Bioinformatics 2005 May;21(9):1859–1875. https://academic.oup.com/bioinformatics/article-lookup/doi/10.1093/bioinformatics/bti310, doi:10.1093/bioinformatics/bti310.
32. Langmead B, Salzberg SL. Fast gapped-read alignment with Bowtie 2. Nature Methods 2012 Apr;9(4):357–359. https://www.nature.com/articles/nmeth.1923, doi:10.1038/nmeth.1923.
33. Lu J, Rincon N, Wood DE, Breitwieser FP, Pockrandt C, Langmead B, et al. Metagenome analysis using the Kraken software suite. Nature Protocols 2022 Dec;17(12):2815–2839. https://www.nature.com/articles/s41596-022-00738-y, doi:10.1038/s41596-022-00738-y.
34. Katz LS, Griswold T, Lindsey R, Lauer A, Im MS, Williams G, et al. Kraken with Kalamari: Contamination Detection; 2021,

accessed 24 June 2024.
35. Ewels P, Magnusson M, Lundin S, Käller M. MultiQC: summarize analysis results for multiple tools and samples in a single report. Bioinformatics 2016 Oct;32(19):3047–3048. https://academic.oup.com/bioinformatics/article/32/19/3047/2196507, doi:10.1093/bioinformatics/btw354.
36. Nanoplot — RCAC Biocontainers v1.0 documentation;, https://biocontainer-doc.readthedocs.io/en/latest/source/nanoplot/nanoplot.html, accessed 24 June 2024.
37. Portik DM, Brown CT, Pierce-Ward NT. Evaluation of taxonomic classification and profiling methods for long-read shotgun metagenomic sequencing datasets. Bioinformatics; 2022.
38. Leidenfrost RM, Pöther DC, Jäckel U, Wünschiers R. Benchmarking the MinION: Evaluating long reads for microbial profiling. Scientific Reports 2020;10(1):5125. https://www.nature.com/articles/s41598-020-61989-x, doi:10.1038/s41598-020-61989-x.
39. Govender KN, Eyre DW. Benchmarking taxonomic classifiers with Illumina and Nanopore sequence data for clinical metagenomic diagnostic applications. Microbial Genomics 2022 Oct;8(10). https://www.microbiologyresearch.org/content/journal/mgen/10.1099/mgen.0.000886, doi:10.1099/mgen.0.000886.
40. Meyer F, Fritz A, Deng ZL, Koslicki D, Lesker TR, Gurevich A, et al. Critical Assessment of Metagenome Interpretation: the second round of challenges. Nature Methods 2022 Apr;19(4):429–440. https://www.nature.com/articles/s41592-022-01431-4.
41. Ondov BD, Bergman NH, Phillippy AM. Interactive metagenomic visualization in a Web browser. BMC Bioinformatics 2011 Dec;12(1):385. https://bmcbioinformatics.biomedcentral.com/articles/10.1186/1471-2105-12-385, doi:10.1186/1471-2105-12-385.
42. Bik HM, Pitch Interactive. Phinch: An interactive, exploratory data visualization framework for –Omic datasets. Genomics; 2014.
43. Breitwieser FP, Salzberg SL. Pavian: Interactive analysis of metagenomics data for microbiomics and pathogen identification; 2016, https://www.biorxiv.org/content/10.1101/084715v1, doi:10.1101/084715.
44. Lin Y, Yuan J, Kolmogorov M, Shen MW, Chaisson M, Pevzner PA. Assembly of long error-prone reads using de Bruijn graphs. Proceedings of the National Academy of Sciences 2016 Dec;113(52). https://pnas.org/doi/full/10.1073/pnas.1604560113, doi:10.1073/pnas.1604560113.
45. Oxford Nanopore Technologies. Medaka;, https://github.com/nanoporetech/medaka, accessed 24 June 2024.
46. Seemann T. ABRicate;, https://github.com/tseemann/abricate, accessed 24 June 2024. Version: 2014-07-17.
47. Chen L, Zheng D, Liu B, Yang J, Jin Q. VFDB 2016: hierarchical and refined dataset for big data analysis—10 years on. Nucleic Acids Research 2016 Jan;44(D1):D694–D697. https://academic.oup.com/nar/article-lookup/doi/10.1093/nar/gkv1239, doi:10.1093/nar/gkv1239.
48. Feldgarden M, Brover V, Haft DH, Prasad AB, Slotta DJ, Tolstoy I, et al. Validating the AMRFinder Tool and Resistance Gene Database by Using Antimicrobial Resistance Genotype-Phenotype Correlations in a Collection of Isolates. Antimicrobial Agents and Chemotherapy 2019 Nov;63(11):e00483–19. https://journals.asm.org/doi/10.1128/AAC.00483-19, doi:10.1128/AAC.00483-19.
49. Pearce ME, Alikhan NF, Dallman TJ, Zhou Z, Grant K, Maiden MCJ. Comparative analysis of core genome MLST and SNP typing within a European Salmonella serovar Enteritidis outbreak. International Journal of Food Microbiology 2018;274:1–11. https://linkinghub.elsevier.com/retrieve/pii/S0168160518300746, doi:10.1016/j.ijfoodmicro.2018.02.023.
50. Hong Kong University - Biomedical Algorithms Lab (BAL). Clair3 - Symphonizing pileup and full-alignment for high-performance long-read variant calling;, original Release Date: 2021-03-30. Accessed 25 June 2024.
51. Danecek P, Bonfield JK, Liddle J, Marshall J, Ohan V, Pollard MO, et al. Twelve years of SAMtools and BCFtools. GigaScience 2021 Jan;10(2):giab008. https://academic.oup.com/gigascience/article/doi/10.1093/gigascience/giab008/6137722.
52. Cingolani P, Platts A, Wang LL, Coon M, Nguyen T, Wang L, et al. A program for annotating and predicting the effects of single nucleotide polymorphisms, SnpEff: SNPs in the genome of Drosophila melanogaster strain w $^{1118}$ ; iso-2; iso-3. Fly 2012 Apr;6(2):80–92. http://www.tandfonline.com/doi/abs/10.4161/fly.19695.
53. Danecek P, McCarthy SA. BCFtools/csq: Haplotype-aware variant consequences;, https://www.biorxiv.org/content/10.1101/090811v2, accessed 24 June 2024.
54. Price MN, Dehal PS, Arkin AP. FastTree 2 – Approximately Maximum-Likelihood Trees for Large Alignments. PLoS ONE 2010 Mar;5(3):e9490. https://dx.plos.org/10.1371/journal.pone.0009490, doi:10.1371/journal.pone.0009490.
55. jupyter/jupyter: Jupyter metapackage for installation, docs and chat;, https://github.com/jupyter/jupyter/tree/master, accessed 24 June 2024.
56. Van Rossum G, Drake FL. Python 3 Reference Manual. Scotts Valley, CA: CreateSpace; 2009.
57. Wes McKinney. Data Structures for Statistical Computing in Python. In: Stéfan van der Walt, Jarrod Millman, editors. Proceedings of the 9th Python in Science Conference; 2010. p. 56 – 61.
58. pandas development team T, pandas-dev/pandas: Pandas. Zenodo; 2020. https://doi.org/10.5281/zenodo.3509134.
59. Hunter JD. Matplotlib: A 2D graphics environment. Computing in Science & Engineering 2007;9(3):90–95.
60. Waskom M. seaborn: statistical data visualization. Journal of Open Source Software 2021 Apr;6(60):3021. https://joss.theoj.org/papers/10.21105/joss.03021.
61. Harris CR, Millman KJ, van der Walt SJ, Gommers R, Virtanen P, Cournapeau D, et al. Array programming with NumPy. Nature 2020 Sep;585(7825):357–362. https://doi.org/10.1038/s41586-020-2649-2.
62. Martin M. Cutadapt removes adapter sequences from high-throughput sequencing reads. EMBnetjournal 2011 May;17(1):10. http://journal.embnet.org/index.php/embnetjournal/article/view/200, doi:10.14806/ej.17.1.200.
63. Bray S, Chilton J, Bernt M, Soranzo N, van den Beek M, Batut B, et al. The Planemo toolkit for developing, deploying, and executing scientific data analyses in Galaxy and beyond. Genome Research 2023;33(2):261–268. https://genome.cshlp.org/content/33/2/261, doi:10.1101/gr.276963.122.
64. Wilkinson MD, Dumontier M, Aalbersberg IJ, Appleton G, Axton M, Baak A, et al. The FAIR Guiding Principles for scientific data management and stewardship. Scientific Data 2016 Mar;3(1):160018. https://www.nature.com/articles/sdata201618.
65. Chue Hong NP, Katz DS, Barker M, Lamprecht AL, Martinez C, Psomopoulos FE, et al. FAIR Principles for Research Software (FAIR4RS Principles); 2021, https://rd-alliance.org/group/fair-research-software-fair4rs-wg/outcomes/fair-principles-research-software-fair4rs, doi:10.15497/RDA00065. Accessed 24 June 2024.
66. Visser Cd, Johansson LF, Kulkarni P, Mei H, Neerincx P, Velde KJvd, et al. Ten quick tips for building FAIR workflows. PLOS Computational Biology 2023 Sep;19(9):e1011369. https://journals.plos.org/ploscompbiol/article?id=10.1371/journal.pcbi.1011369.
67. Project G. Galaxy Workflows maintained by the Intergalactic Workflow Commission; 2024, accessed 24 June 2024. Original date: June 30, 2018.

68. Pathogen detection from (direct Nanopore) sequencing data using Galaxy - Foodborne Edition;, https://training.galaxyproject.org/training-material/topics/metagenomics/tutorials/pathogen-detection-from-nanopore-foodborne-data/tutorial.html, accessed 24 June 2024.
69. Soiland-Reyes S, Sefton P, Crosas M, Castro LJ, Coppens F, Fernández JM, et al. Packaging research artefacts with RO-Crate. Data Science 2022 Jan;5(2):97–138. https://content.iospress.com/articles/data-science/ds210053.
70. Sefton P, Ó Carragáin E, Soiland-Reyes S, Corcho O, Garijo D, Palma R, et al. RO-Crate Metadata Specification 1.1.3; 2023, https://zenodo.org/records/7867028, doi:10.5281/zenodo.7867028. Accessed 25 June 2024.
71. Nasr E, Henger A, Grüning B, Zierep P, Batut B. Samples Preparation for Foodborne Pathogen Detection and Tracking project; 2023, https://www.protocols.io/view/samples-preparation-for-foodborne-pathogen-detecti-cwhdxb26, doi:dx.doi.org/10.17504/protocols.io.8epv5x1jdg1b/v1. Accessed 01 July 2024.
72. CFU Full Form;, https://unacademy.com/content/neet-ug/full-forms/cfu/, accessed 24 June 2024.
73. Bioscientia. What do the terms dual target PCR and Ct value mean? | Laboratory Diagnostics; 2020, https://www.bioscientia.de/en/home/our-news/2020/07/what-do-the-terms-dual-target-pcr-and-ct-value-mean/, accessed 24 June 2024.
74. McClelland M, Sanderson KE, Spieth J, Clifton SW, Latreille P, Courtney L, et al. Complete genome sequence of Salmonella enterica serovar Typhimurium LT2. Nature 2001;413(6858):852–856. https://www.nature.com/articles/35101614, doi:10.1038/35101614.
75. Abukhattab S, Hosch S, Abu-Rmeileh NME, Hasan S, Vonaesch P, Crump L, et al. Whole-genome sequencing for One Health surveillance of antimicrobial resistance in conflict zones: a case study of Salmonella spp. and Campylobacter spp. in the West Bank, Palestine. Applied and Environmental Microbiology 2023 Sep;89(9):e00658–23. https://journals.asm.org/doi/10.1128/aem.00658-23, doi:10.1128/aem.00658-23.
76. Salmonella enterica subsp. enterica (ID 942086) - BioProject - NCBI;, https://www.ncbi.nlm.nih.gov/bioproject/942086, accessed 24 June 2024.
77. Campylobacter jejuni subsp. jejuni (ID 942088) - BioProject - NCBI;, https://www.ncbi.nlm.nih.gov/bioproject/942088, accessed 24 June 2024.
78. Nasr E, Henger A, Grüning B, Zierep P, Batut B. Benchmarking PathoGFAIR; 2024, https://www.protocols.io/view/benchmarking-pathogfair-e6nvwbp4zvmk/v1, doi:dx.doi.org/10.17504/protocols.io.e6nvwbp4zvmk/v1. Accessed 14 November 2024.
79. Seemann T. mlst;, original Release Date: 2014-05-03. Accessed 24 June 2024.
80. Maier W, Bray S, van den Beek M, Bouvier D, Coraor N, Miladi M, et al. Ready-to-use public infrastructure for global SARS-CoV-2 monitoring. Nature Biotechnology 2021 Oct;39(10):1178–1179. https://www.nature.com/articles/s41587-021-01069-1, doi:10.1038/s41587-021-01069-1.
81. PRJNA982679 - SRA - NCBI;, https://www.ncbi.nlm.nih.gov/sra/PRJNA982679, accessed 24 June 2024.
82. usegalaxy-eu/PathoGFAIR: PathoGFAIR: Galaxy FAIR Workflows for Pathogen Detection and Samples Comparison;, https://github.com/usegalaxy-eu/PathoGFAIR, accessed 25 June 2024.
83. Digital Life Sciences Internal Call for Academia-Industry Collaborations;, https://www.eosc-life.eu/industrycall/, accessed 24 June 2024.

Figure 1

Click here to access/download;Figure;Figure_1.pdf

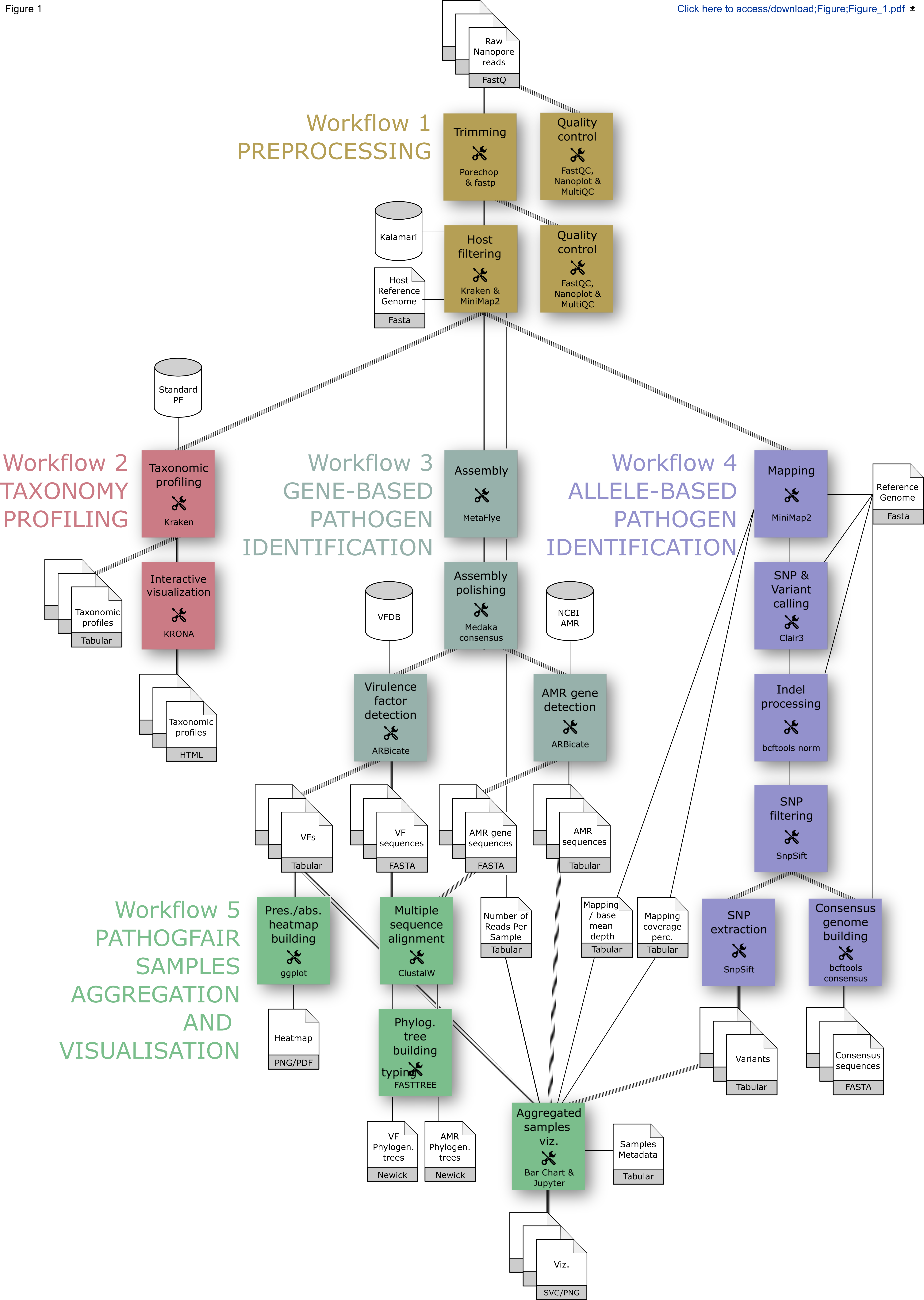

Supplementary Figure S1

Click here to access/download
**Supplementary Material**
Supplementary_Figure_S1.png

Supplementary Table T1

Click here to access/download
**Supplementary Material**
Supplementary_Table_T1.tsv

Supplementary Table T2

Click here to access/download
**Supplementary Material**
Supplementary_Table_T2.tsv

Supplementary Figure S3

Click here to access/download
**Supplementary Material**
Supplementary_Figure_S3.png

Supplementary Figure S4

Click here to access/download
**Supplementary Material**
Supplementary_Figure_S4.png

Supplementary Figure S5

Click here to access/download
**Supplementary Material**
Supplementary_Figure_S5.png

Supplementary Figure S7

Click here to access/download
**Supplementary Material**
Supplementary_Figure_S7.png

Supplementary Figure S8

Click here to access/download
**Supplementary Material**
Supplementary_Figure_S8.png

Supplementary Figure S9

Click here to access/download
**Supplementary Material**
Supplementary_Figure_S9.png

Supplementary Table T3

Click here to access/download
**Supplementary Material**
Supplementary_Table_T3.tsv

Supplementary Table T4

Click here to access/download
**Supplementary Material**
Supplementary_Table_T4.tsv

Personal Cover Click here to access/download;Personal Cover;Cover_letter.pdf

Dear GigaScience Editorial Board,

We are pleased to submit our manuscript entitled "PathoGFAIR: A Series of FAIR and Adaptable (Meta)Genomics Workflows for (Foodborne) Pathogens Detection and Tracking", authored by Engy Nasr, Anna Henger, Björn Grüning, Paul Zierep, and myself, for consideration as a technical note.

Foodborne pathogen contamination represents a significant global health threat, affecting approximately 600 million people annually. Microbiological analysis is critical during foodborne outbreak investigations to detect and identify responsible pathogens and trace contamination sources. Metagenomic approaches provide a comprehensive overview of the genomic composition of microbial communities, enabling the detection of potential pathogens in samples. Coupled with user-friendly sequencing techniques like Oxford Nanopore sequencing, these approaches become faster and easier to implement. However, a major limitation is the lack of accessible, user-friendly, and openly available pipelines for pathogen identification and tracking from metagenomic data.

In this manuscript, we introduce PathoGFAIR, a suite of Galaxy-based FAIR workflows that utilise advanced tools for the detection and tracking of pathogens from metagenomic Nanopore sequencing data. Although primarily designed for the identification of foodborne pathogens, these workflows are versatile and applicable to any metagenomic Nanopore dataset. PathoGFAIR features several visualisations and comprehensive reports to ensure thorough analysis. We validated PathoGFAIR using 130 samples containing diverse pathogens from multiple hosts under various experimental conditions. Our workflows successfully identified and tracked expected pathogens at the species level in both isolated and non-isolated samples, given sufficient Colony-Forming Unit (CFU) and Cycle Threshold (Ct) values.

PathoGFAIR's integration into the Galaxy and WorkflowHub ecosystems ensures reproducibility and accessibility. Additionally, we have developed dedicated Galaxy training materials to support users in the interpretation of the workflows and provide ongoing assistance from the Galaxy and microbial communities.

We believe our integration efforts and the accompanying training materials meet the editorial guidelines on reproducible research, making this manuscript a suitable technical note for GigaScience. Given the increasing publication of pathogen protocols, we are confident that our manuscript will be valuable to a broad spectrum of GigaScience readers.

We confirm that this manuscript is original, has not been published previously, and is not under consideration by any other journal. The authors declare no conflicts of interest.

Thank you for your consideration.

Sincerely,
Dr. Bérénice Batut
