# Supplementary material for: PathoGFAIR: a collection of FAIR and adaptable (meta)genomics workflows for (foodborne) pathogens detection and tracking
Source: Gigascience. 2025 Sep 26;14:giaf017. doi: 10.1093/gigascience/giaf017 (PMC12466118; doi:10.1093/gigascience/giaf017)
Supplement: giaf017_Authors_Response_To_Reviewer_Comments_Original_Submission [file giaf017_authors_response_to_reviewer_comments_original_submission.pdf]

Author's Response To Reviewer Comments

> We sincerely thank the editor and reviewers for their valuable suggestions and constructive feedback. Suggested changes to the manuscript have been implemented and highlighted in red for ease of tracking.

Reviewer #1

The authors present PathoGFAIR, a set of Galaxy workflows for the metagenomic analysis of shotgun Nanopore sequencing from isolated and non-isolated pathogens in contaminated food samples. They complement their work by analysing and releasing two datasets, one from isolated and the other from non-isolated samples, with the primary objective of illustrating the potentiality of the workflows. These datasets could also be used as benchmarks for future works.

The manuscript is generally well-written, and the authors highlight the advantages of the proposed workflows in Table 1 by comparing them to similar solutions. The workflows are well integrated into the Galaxy network, are available on the three main usegalaxy instances, and provide a thorough tutorial through the Galaxy training platform. A notable advantage of PathoGFAIR over similar workflows is that, thanks to Galaxy, the final user can easily tailor them by replacing any tool in the workflow with others available in the Galaxy ecosystem. This also allows easy updates for the tools in the workflows.

> We appreciate your insightful feedback and suggestions for improving our manuscript. Your comments are invaluable in helping us enhance the clarity and robustness of our work.

A few minor points that, if addressed, in my opinion, could further strengthen the manuscript:

1 - The rationale behind the tool selection in each of the four workflows is not always clear. While insights are present for workflows 1 and 4, this is not true for workflows 2 and 3. The reader would benefit from understanding why one tool has been preferred over another for the same task, even more so, given the possibility to modify the workflows easily, when this preference could be the other way around in particular use cases or conditions.

> We agree that the rationale behind tool selection should be clearer, particularly for Workflows 2 and 3. For Workflow 2 (Taxonomy Profiling), Kraken2 was selected due to its speed, high sensitivity, and ability to handle large reference databases, which is essential for the detection of diverse pathogens in metagenomic datasets. For Workflow 3 (Gene-based Pathogen Identification), ABRicate was chosen for its comprehensive virulence factor and antimicrobial resistance gene databases and ease of use in conjunction with the Galaxy platform.
> We have then revised the "Implementation" section of the manuscript to clearly explain the rationale for selecting Kraken2 for taxonomy profiling and ABRicate for gene-based pathogen identification in Workflows 2 and 3. The description now emphasises the tool's advantages for specific tasks and highlights when other tools could be appropriate depending on user needs or specific experimental conditions.

2—One of the main factors for a successful metagenomic analysis is the correctness, completeness, and up-to-dateness of the reference data. The authors should briefly describe how PathoGFAIR addresses this in Galaxy.

> Thank you for highlighting this critical factor. Ensuring the accuracy and currency of reference data is indeed fundamental for robust metagenomic analysis. PathoGFAIR leverages Galaxy's integrated Data Managers, which enables Galaxy admins to provide up-to-date reference data. These Data Managers automate the download, installation, and regular update of essential reference databases, ensuring that PathoGFAIR users work with complete, accurate, and up-to-date reference information.
> PathoGFAIR workflows are configured to use well-maintained and reputable sources, such as NCBI and other public pathogen reference repositories e.g. StandardPlusPF, Kalamari, and VFDB, which further support accuracy and comprehensiveness. Additionally, Galaxy's user-friendly interface enables users to select preferred references or request the inclusion of specific databases via Galaxy administrators, adding

to the workflow’s adaptability for diverse use cases.
> We have added details in the “Implementation” section on PathoGFAIR’s use of Galaxy’s Data Managers, explaining how these tools support reliable reference data management. Furthermore, to illustrate the robustness of PathoGFAIR, we have benchmarked it against comparable systems and calculated sensitivity and precision metrics of PathoGFAIR with tested samples, now included in the manuscript to demonstrate PathoGFAIR’s accuracy in pathogen identification, in an added “Benchmarking PathoGFAIR” section.

3—While this workflow is clearly stated to be tailored for shotgun metagenomic sequencing, the authors contrast this approach only with targeted sequencing. Instead, they should also discuss the 16s rRNA metagenomic approach, for which Nanopore kits are available, and why PathoGFAIR has been limited to the analysis of shotgun data.

> While Nanopore 16S rRNA sequencing kits are indeed available, PathoGFAIR was intentionally developed for shotgun metagenomic sequencing due to its broader scope in pathogen identification. Unlike 16S rRNA sequencing, which is limited to bacterial taxa, shotgun metagenomics enables the comprehensive detection of a broader range of pathogens, including bacteria targeted in targeted in our workflows, as well as viruses, and fungi, which could be easily incorporated in the future without requiring predefined amplicon targets.
> Moreover, shotgun metagenomics allows access to complete genomes rather than specific gene regions, facilitating taxa-agnostic identification of antimicrobial resistance (AMR) and virulence genes. This capability is central to PathoGFAIR’s functionality, as it enables detailed characterisation and tracking of pathogens beyond taxonomic profiling.
> We have updated the introduction to include a comparison between shotgun metagenomic and 16S rRNA sequencing, clarifying why shotgun metagenomics offers the broader capabilities required by PathoGFAIR.

Reviewer #2

Nasr and colleagues present an, at times, well-written manuscript with an interesting and robust pipeline that includes well-known softwares (you must make sure to cite the authors of these).

> We sincerely thank the reviewer for their insightful feedback and constructive comments.
> We have ensured that all software tools mentioned in the manuscript are properly cited, with appropriate references to their original authors.

However, the manuscript is, quote "...a collection of Galaxy-based FAIR workflows employing state-of-the-art tools to detect and track pathogens from metagenomic Nanopore sequencing". Its repeated how well it works, they even compare it to other software in table 1 (without proper benchmark). These initial statements are however not supported by the findings. The Salmonella from the spiked samples are, as expected from food matrix present in low quantity), difficult to do more than state that the genus is present, and only a fraction of the samples can actually "complete" the entire pipeline. Also, the benchmarking is not really benchmarking (compare and measure this software against other competing software). No such comparison is done, and even though the intention of PathoGFAIR as stated throughout the paper, is detection and analysis of metagenomic samples, the benchmarking is done on isolate based wgs.

> Table 1 is a feature comparison rather than a performance benchmark. Its purpose is to highlight key aspects of PathoGFAIR, such as modularity, reproducibility, ease of use, and adherence to FAIR principles (Findability, Accessibility, Interoperability, and Reusability). These features are crucial for making PathoGFAIR accessible and adaptable for pathogen detection in diverse metagenomic contexts. Unlike a performance-focused benchmark, Table 1 emphasises design and usability differences between PathoGFAIR and other pipelines.
> The manuscript introduces PathoGFAIR as a collection of FAIR workflows and presents its core capabilities through two distinct use cases. It is intended as a technical note, focusing on system design, workflows, and practical application, rather than a direct performance comparison with competing pipelines. We appreciate your suggestion and have expanded our efforts to include a performance benchmark using the spiked non-isolated samples from the first use case. This benchmark compares PathoGFAIR with the pipelines in Table 1 and evaluates their performance. Results are presented as a

heatmap (Supplementary Figure S9) in the newly added “Benchmarking PathoGFAIR” section, accompanied by detailed explanatory paragraphs.
> Additionally, we have included a replication guide in the PathoGFAIR GitHub repository and a protocol on protocols.io detailing the benchmarking steps. These additions ensure transparency and reproducibility of the benchmarking process.
> We have substantiated all statements about PathoGFAIR’s performance with findings from the use cases and the new benchmarking sections. For instance, in the first use case, pathogens were detected with a success rate of 97.8% at the species rank, 60.9% at the subspecies rank, and 13% at the strain rank. PathoGFAIR successfully identified pathogens in all tested samples but one across both use cases and the benchmark. It is important to note that samples do not need to complete the entire pipeline for pathogen identification. Pathogen detection occurs as early as the second workflow (Taxonomy profiling), while subsequent workflows provide additional analyses, such as pathogenicity characteristics and visualisation. This modular design allows workflows to function independently or in parallel, ensuring flexibility and efficiency for various metagenomic analyses.
> Finally, we agree that further benchmarking with additional metagenomics datasets could provide deeper insights into PathoGFAIR’s performance and will explore this in future work.

It is also evident that the authors are not microbiologists as the manuscript is riddled with taxonomical misunderstandings about the vast genus Salmonella and when to use capital letters and italics.

> Thank you for highlighting the taxonomic inconsistencies. We have carefully reviewed and revised the manuscript to ensure that all taxonomic names, including Salmonella and other genera, as well as their subsequent taxonomic ranks, are correctly formatted according to scientific conventions (e.g., italicization and capitalization). These have been applied consistently throughout the text and figures, following the guidelines outlined in this reference (https://www.ncbi.nlm.nih.gov/pmc/articles/PMC86943/). We appreciate your attention to this detail.

I am also lacking a proper discussion here on the results found in the spiking experiment in light of current EU legislation on Salmonella. Can this pipeline help in this regard?

> Thank you for raising this important point. The primary aim of our study was not to propose an alternative to EN/ISO 6579, as such a method would require a comprehensive validation process, including sensitivity analyses, to meet regulatory standards. Instead, our focus was on the development and optimisation of a bioinformatic workflow for the detailed characterisation of microbial species, including subspecies rank resolution, within a food matrix using whole genome sequencing (WGS).
> EN/ISO 6579 specifies an enrichment step followed by classical microbiology or real-time PCR for confirmation of Salmonella presence. In contrast, our workflows aim to analyse the enrichment broth directly, leveraging WGS to characterise all Salmonella present in the sample without requiring single-strain isolation. This approach offers the added benefit of insights into subspecies diversity and potential links to previous outbreak strains
> The spiking experiment presented in our study served as a proof of concept, demonstrating the feasibility of detecting and characterising Salmonella in complex food matrices using WGS. This lays the foundation for future applications where detailed subspecies-rank information is critical.
> We have added a paragraph in the conclusion section to further discuss the potential applications of this approach in light of current EU legislation on Salmonella and its possible role in supporting outbreak investigations and traceability efforts.

Sensitivity and specificity metrics are also lacking.

> We have now included sensitivity matric for PathoGFAIR at the end of both use case sections in the manuscript. As we explain in the text, specificity matric can not be applied because both use cases do not include true negative samples, making specificity calculations inapplicable in this context. These additions provide a clearer evaluation of PathoGFAIR’s performance and ensure that the results are appropriately framed given the experimental design.

Abstract

"foodborne pathogen data" / "metagenomic Nanopore pathogenic data" - suggest to rewrite, as what I think you are trying to say is " initially developed to detect foodborne pathogens from metagenomic nanopore data, the workflow can be used to detect any pathogen."

> Thank you for your suggestion. We have updated the abstract to provide clearer phrasing, incorporating your feedback. The revised text now accurately conveys that while the workflow was initially developed to detect foodborne pathogens from metagenomic Nanopore data, it is versatile and can be used to detect a wide range of pathogens.

"Colony-forming unit and Cycle Threshold values." rewrite sentence, I do not completely understand what you are trying to say. what is "sufficient colony forming units?" It will vary as well between pathogens (infection dose varies). You could rather state your sensitivity of the pipeline here - even though i think that sampling prep, library prep and seq influences that more than the bioinformatics.

> We agree that Ct and CFU values are closely tied to sample preparation and library preparation, which significantly impact the performance of any bioinformatics workflow. In the first use case (samples without prior isolation), where these values were provided as part of the metadata, we examined their influence on our workflows performance. Specifically, we observed that higher CFU/ml values and lower Ct values improved the comprehensiveness of pathogen detection, particularly for challenging samples like those in Use Case 1 (spiked samples)
> Rather than defining an absolute threshold for sensitivity, we focused on how these parameters influenced the bioinformatics outcomes and we provided practical recommendations for users. These recommendations highlight the conditions under which optimal sensitivity and accuracy can be achieved when using PathoGFAIR, emphasizing the importance of proper sample preparation and sequencing.
> To address your suggestion, the sentence in the abstract has been revised to better reflect the role of Ct and CFU values in influencing workflow outcomes. Additional clarification has been added to the use cases and conclusion sections, detailing the sensitivity and best practices for pathogen detection.

"In any sample": did you test all matrixes?

> We meant to say “in all tested samples within the two use cases”. The sentence has been rewritten for clarity to avoid any misinterpretation.

"sample is isolated or incubated before seq" you cannot isolate a sample, but you isolate a bacteria from a sample. unprecise language.

> Thank you for pointing out the imprecise language. We have revised the sentence to accurately describe the isolation of bacteria from a sample.

Introduction

In general, its well written, but a bit unprecise here and there. The authors also rely a lot on the following words: "rapid" "accurate".

> Thank you for your feedback. We have carefully revised the introduction to enhance clarity and precision. Redundant terms such as “rapid” and “accurate” have been removed and more specific descriptions have been provided where necessary to ensure greater precision in the language.

"outbreaks and epidemics" - rewrite, these are the same.
"efforts to mitigate their spread and ensure food safety" again, complementary terms - rewrite.
"global public health authorities" we do have everything from local to global food safety and public health authorities, I think one should highlight this. There is a difference between for instance EFSA and ECDC.
"The utilisation of Nanopore sequencing data, as exemplified in studies like [7]," citing practices like this is not really reader friendly. Suggest to write what they actually did in seven (as for instance the detection of blah in blah as shown in 7).
"Once (meta)genomics data has been generated, bioinformatics approaches enable the rapid and accurate detection"; repetition of chapter above. You write in the former chapter that "the utilisation of nanopore

data" which also includes bioinformatics of course.

> Thank you for your suggestions. We have revised the manuscript to address your points.
> The terms "outbreaks and epidemics" have been clarified to avoid redundancy.
> The phrase "efforts to mitigate their spread and ensure food safety" has been reworded to provide more distinct and complementary descriptions.
> We have highlighted the range of food safety and public health authorities, specifying the differences between local, national, and global organizations, such as EFSA and ECDC.
> The citation of study [7] has been updated to more clearly describe the findings, such as the utility in closing genomic gaps with real-time, long-read sequencing data for outbreak investigation as shown in the referenced study.
> The repetition in the chapter regarding bioinformatics and Nanopore sequencing has been removed, ensuring the flow of the manuscript is smoother and avoids redundancy.

"isolation can be complex"? do you mean complicated or work intensive?

> “Work-intensive” is indeed a more accurate description of what we meant. We intended to convey that the isolation process is laborious and time-consuming, often involving multiple steps and advanced techniques. We have revised the sentence to make this clearer.

SURPI and Sunbeam is freely available?
https://microbiomejournal.biomedcentral.com/articles/10.1186/s40168-019-0658-x
https://chiulab.ucsf.edu/surpi/

> Thank you for your comment. As noted in Table 1, SURPI and Sunbeam are freely available. We have clarified the text to ensure this is clearly stated.

"PathoGFAIR: pathogen identification and tracking from metagenomics". Im not convinced that it can perform tracing in an outbreak where only a few SNPs are allowed.

> Thank you for your feedback. By tracking, we are referring to monitoring the presence of the pathogens across samples using sample metadata such as location and time. PathoGFAIR enables users to identify when and where a pathogen appears in the sample based on the metadata.
> We do not mean ‘tracing’ in the context of an outbreak where only a few SNPs are involved. While PathoGFAIR primarily focuses on pathogen identification, we recognise that additional tools such as MLST and SNP-based tracing would significantly enhance its ability to trace pathogens at finer taxonomic ranks, including subspecies and strain-rank differences. We have acknowledged this potential extension in the conclusion section of the manuscript.

PathoGFAIR does not really speed up the process of sampling, does it. Actually, it takes more time to extract crude dna from a sample than to place it in a enrichment broth or do a dilution series, so the presteps are not really a part of this.

> You are correct that PathoGFAIR does not affect the actual sampling or DNA extraction time, as it focuses on the bioinformatics analysis. The primary aim of the samples in this study was to demonstrate the functionality of PathoGFAIR and to provide recommendations for best practices in downstream analysis. The pre-steps, such as DNA extraction or enrichment broth preparation, are outside the scope of PathoGFAIR’s capabilities.

"Tracking pathogens" - again, if species level is the lowest rank it can go to, its not enough to perform tracking.

> We have clarified the explanation in the manuscript to distinguish between “tracking” and “tracing”.
> Tracking refers to monitoring the presence of a pathogen across all given samples, focusing on when and where it appears, using sample metadata such as time and location. Tracing refers to more detailed methods, such as SNP or MLST analysis, which can identify finer taxonomic differences, including strain-level or subspecies-level distinctions.

> This distinction should help avoid any confusion between the two terms in the context of our study.

Implementation

Overview

"input data is seq data generated w nanopore" basecalling is not included in the workflow?
How is this performed? It affects the quality of the reads, so its nice to know what you did.

> Thank you for your comment. As outlined in our published protocol for sample preparation (Step 8: https://www.protocols.io/view/samples-preparation-for-foodborne-pathogen-detecti-8epv5x1jdg1b/v1?step=8), as well as in the manuscript's first use case (Samples without prior isolation), real-time basecalling was performed using the MinKNOW software with the MinION Mk1C device. We recognize that basecalling is a crucial step, as it affects the quality of the reads, and we have now added a brief explanation in the overview section to clarify how this is handled in our workflow.

The chapter is very wordy, and contains a lot of fill-words with salespitches almost. I would recommend rewriting it, for instance:
Chapter that starts with subsequently and describes the different workflows and how they work together can be compressed.
And the last three sections are salespitching.

> To address the concern regarding the wordiness, we have condensed the section describing how the workflows interconnect. We have focused on providing a more concise explanation while retaining the essential technical details. Additionally, we have removed any language that could be perceived as overly promotional. However, we believe it is important to maintain the discussion of how workflows work together, as this highlights the flexibility and modularity of PathoGFAIR.

WF1: Preprocessing

How stringent filtering and quality control are implemented in the workflow? How good quality do you need for the wp2-4 to work sufficiently well? Did you test?

> Thank you for your question. Quality control in PathoGFAIR is performed using the Fastp tool, which removes low-quality reads and adapters. For downstream analyses, we set a quality score of Q20 or higher, which is generally sufficient for Nanopore data (https://journals.asm.org/doi/10.1128/jcm.01631-22). However, we have not tested alternative quality filters with our use cases.
> We visualise quality control results, including read length distribution and base quality scores, using MultiQC, which allows users to assess the quality before proceeding to the subsequent workflows. In Use Case 1 (spiked samples), we found that samples with fewer than 2,700 reads after preprocessing were excluded from further analyses in Workflows 3 and 4, as they failed to generate the necessary contigs. This cutoff can be adjusted based on the user's datasets. While we have not tested higher quality score thresholds, users can modify the parameters to suit their data's characteristics.

Food vehicle animal? What is that - do you mean that if you extract dna from bovine meat, you map to bovine genome?

> Thank you for pointing out the unclear terminology. The term "food vehicle animal" refers to the animal from which the food product originated. For example, in the case of chicken meat, the food vehicle animal is the chicken. To make this clear, we have updated the terminology to "host" or "food source".
> Regarding mapping to the host genome, when DNA is extracted from a food product like bovine meat, we map the reads to the bovine genome to remove host-derived sequences. This ensures that only non-host (potentially pathogen-related) sequences are retained for downstream analysis. The user can select the appropriate host genome from a drop-down list before running the workflow.
> We have updated the terminology and provided clearer examples of host genome removal for various food types in the manuscript.

"a tool ten times faster etc etc." is discussion and should be removed from what I think is materials and methods even though the title of the section is workflow 1.

> We respectfully disagree with the suggestion to remove the performance-related details about the tool being "ten times faster." This information is essential for providing context about the tool selection in Workflow 1. Performance metrics, such as speed and computational efficiency, are integral critical considerations in the design of the workflow and help justify why specific tools were chosen over others. Therefore, we believe it is important to include this information in the description of Workflow 1 to give a complete understanding of its implementation.

What is a food host?

> The term "food host" was used to refer to the food product that may be contaminated by a pathogen (e.g. chicken, cow, milk, etc.). To improve clarity, we have updated the terminology to simply "host" and provided clearer examples of how host genome fragments are removed for various food types during analysis.

Kalamari database includes many foodborne pathogens, such as Shigella, E. coli, Campylobacter etc etc. how can you just remove all reads that match to this database?

> Thank you for your comment. In Workflow 1, we use Kraken2 along with the Kalamari database to filter out host-associated sequences (e.g., cow, chicken, etc.) rather than pathogen sequences. Specifically, we select taxIDs related to probable food sources (like cow, and chicken) to remove host genomic DNA, while ensuring that pathogen-associated sequences are retained. The Kalamari database includes foodborne pathogens such as Shigella, E. coli, and Campylobacter, but these sequences are not removed in this step.
> We have clarified this distinction in the manuscript, highlighting that the primary aim of using the Kalamari database is to eliminate host-related contamination while preserving pathogen-related sequences for further analysis.

Table 1: Innuendo is based on isolate WGS, and not intended for WGS. Also, it has its own built in wgMLST schema employed using chewbbacca, so it definitely has allele-abased pathogen identification. Its intended for illumina data. Victors are strictly a platform to analyse virulence factors and not intended even for taxonomic profiling, and its webinterface doesn't work. IDseq has step-by-step guides available on their webpage, so I think that qualifies as a tutorial. You can also contact them (user support). I guess the same is true for OneCodex, as you actually pay for that one. So the table is unprecise at best and should be corrected (I didn't go through Submeam, SURPI or PAIPline specs to try to check if you got it correctly).

> Thank you for your detailed feedback on Table 1. We have carefully reviewed the specificity of each of the pipelines, and we appreciate your insights, particularly regarding Innuendo, Victors, IDseq, and OneCodex. Below are the changes we have made based on your feedback
> Innuendo: We have corrected the table to clarify that Innuendo is specifically designed for isolate WGS data and not metagenomic samples. We have also updated the entry to indicate that Innuendo utilises allele-based pathogen identification. Additionally, it is intended for Illumina data, not Nanopore sequencing.
> Victors: As you mentioned, Victors is strictly focused on analysing virulence factors and is not intended for taxonomy profiling. We have revised the table to reflect that it is only suitable for gene-based pathogen identification. The * next to the web interface entry already indicated that it malfunctioned when tested, which remains unchanged.
> IDseq: While IDseq does provide step-by-step guides on its webpage, which could qualify as a tutorial, we have updated the table to clarify that these guides are considered documentation rather than a full tutorial, as we define tutorials (e.g providing test datasets and teaching material such as those available in Galaxy Training Network (GTN). We have also acknowledged that IDseq offers user support
> OneCodex: As you correctly point out, OneCodex offers user support as it is a paid service. We have updated the table to reflect this and have noted that it also provides user documentation.
> In addition to these specific updates, we also took this opportunity to re-verify all information in the table by consulting documentation, websites, and published papers for all listed tools, including SURPI, Sunbeam, and PAIPline. This thorough review ensures the accuracy of the table entries, and we have applied these corrections across all pipelines and systems.

Rewrite this. Further, I think you should only include systems / pipelines that are intended for metagenomics.

> Thank you for your feedback. Our intention was to provide a comprehensive comparison of systems and pipelines used for pathogen identification, which includes not only those focused specifically on metagenomics but also those applicable to isolate-based sequencing. However, we understand your concern about focusing solely on metagenomic pipelines.
Additionally, we have updated the comparison to include (BugSeq [https://bugseq.com/analysis#pathogen-identification]) which performs similar analyses to our workflows, albeit it is not free or openly available. This highlights the accessibility advantage of PathoGFAIR.

You have a footnote * that I cannot see in the table as well.

> The footnote (*) refers to the Victors pipeline and is located in the third row of the table. We have made sure to clarify this in the revised version of the manuscript.

WF2 taxonomy profiling

The first sentence needs rewriting.

> We appreciate your suggestion. We have revised the first sentence to improve clarity and ensure it better conveys the intended message.

Two sentences from "Although Kraken2 is a tool design…….." belongs in discussion.

> Thank you for your comment. As we do not have a separate discussion section, we believe it is important to explain the rationale behind our tool choices within the implementation section. This allows us to highlight the specific features of each tool, such as Kraken2, that make them suitable for our workflows, without delving into the results.

WF3

Medaka consensus pipeline : "This task is performed using neural networks applied from a pileup of individual sequencing reads against a draft assembly. " what draft assembly did you use here to create a consensus sequence? Actually, its not polishing contigs, its assemblying them?

> To clarify, Medaka is used to polish the consensus sequences generated from contigs, rather than performing the assembly itself. The contigs are first assembled using a different tool, and Medaka is then applied to refine and improve the accuracy of the consensus sequence based on the individual reads.

Again, there is some descriptions of the software which belongs in the discussion, say the perks one gets from using this tool over the other.

> Thank you for your feedback. As previously mentioned, the implementation section is designed not only to explain the workflows but also to justify the choices made in selecting the tools used. This includes highlighting the advantages of specific tools within the context of our workflows, as these decisions are integral to understanding the functionality and efficiency of the system.

I do not however get how screening for virulence genes = pathogen identification. The thing is that in a complex food matrix or faecal samples from animals, things like stx phages will also be present. These are not stec pathogens unless the phage is inside an e.coli. How do you make sure of the host for such mobile genetic elements as these virulence and amr genes often are located on? Seeing as this is the basis of your pathogen detection?

> Thank you for this insightful comment. We fully recognize the complexity of detecting virulence and AMR

genes, especially when considering mobile genetic elements (MGEs) like phages and plasmids, which can carry these genes independently of their host.
> While it is true that VF and AMR genes can be located on MGEs, these genes are still highly informative markers for identifying potential pathogens. In foodborne outbreak contexts, the presence of these genes often correlates strongly with pathogenic organisms (https://pmc.ncbi.nlm.nih.gov/articles/PMC6653787/).
> Our workflows do not directly cross-verify associations for MGEs, but they generate comprehensive outputs that allow users to conduct such analyses. For example, Workflow 2 (Taxonomy Profiling) generates taxonomic abundance data, including species and subspecies identification using Kraken2, which can be used to identify the host organism. Workflow 3 (Gene-based Pathogen Identification) identifies virulence and AMR genes within the sample. By comparing the taxonomic profiling results from Workflow 2 with the VF and AMR gene data from Workflow 3, users can validate the relevance of detected genes and minimise the risk of misidentifying genes located on MGEs.
> In our use cases, we had prior knowledge of the expected pathogens (e.g., Salmonella and Campylobacter), and the species identified in the samples matched the expected results. For example, in Workflow 4 for the second use case, high mapping coverage (>90%) for both Salmonella and Campylobacter confirmed that the VF and AMR genes detected were linked to correct bacterial hosts, as identified by Workflow 1.
> We acknowledge that additional measures could enhance the accuracy of this process. In future versions of PathoGFAIR, we plan to introduce a validation step to cross-reference VF and AMR gene detection with broader taxonomic markers (e.g., 16S rRNA), ensuring that genes detected from MGEs are correctly attributed to the pathogenic organism. We mentioned these potential improvements in the conclusion section of the manuscript.

WF4

A bit again on choosing software over the other that is discussion food.

> As we mentioned previously, the implementation section is designed to provide not only a description of our workflows but also the rationale behind our tool selection. We believe it is important to explain why specific tools were chosen based on their features, performance, and suitability for the workflows, as this contextual information supports the reader's understanding of our approach.

Wf4/wf5

I am worried about the reliance on snp based technics for nanopore reads. Is the quality good enough to achieve sufficiently robust results?

> Thank you for raising this important point. We acknowledge that SNP-based techniques for Nanopore reads can be challenging due to higher error rates compared to short-read sequencing.
> To address this, we enforce stringent quality control measures and apply a minimum depth of covering threshold of 10x to ensure reliable SNP calls. This minimises the inclusion of false-positive variants caused by sequencing errors.
> Additionally, we use Clair3, a state-of-the-art variant caller specifically optimised for Nanopore long-read sequencing. Clair3 leverages deep learning to enhance the accuracy of SNP and small indel detection, making it a robust choice for long-read data.
In our workflows, SNP-based analyses have been successfully applied in use cases with known pathogens, where results matched expected outcomes, providing additional confidence in the reliability of this approach.
> We have updated the manuscript to include these details, explicitly addressing the robustness of SNP-based techniques in the context of Nanopore reads and the steps taken to ensure accurate results.

Easily adaptable workflows

Last section is repetition (about each wf operating independently)

> Thank you for your observation. While the final section does revisit points mentioned earlier, it serves a distinct purpose: to summarise and emphasise how the workflows can operate independently or in

combination, offering flexibility to the users. This summary is intended to provide a clear take-home message for readers about the modular design of PathoGFAIR and its practical applications. We believe this is an important inclusion to ensure users understand how to best utilise the workflows for their specific needs.

Use cases

Samples Without Prior Pathogen Isolation

Data generation

Please revise how to write Salmonella names correctly. They should be in italics for genus, species and subspecies names, while the serovar/serotype is non italic and capital letter. So the correct term would be:
* Salmonella enterica subsp. enterica serovar Houtenae, or in short; Salmonella Houtenae.
* The strain DSM554 is of serovar Typhimurium, and this should referenced like this: Salmonella enterica subsp. enterica serovar Typhimurium strain DSM 554

> Thank you for highlighting this important correction. We have reviewed the manuscript thoroughly to ensure that all mentions of Salmonella species, subspecies, and serovars are formatted according to the correct nomenclature standards. Additionally, any occurrences in figures and figure captions have been updated accordingly. We appreciate your attention to detail, and the manuscript now adheres to the proper scientific conventions for nomenclature.

First two sentences are contradictory to eachother?

> Thank you for pointing this out. We understand how the original phrasing could appear contradictory. Upon review, we have clarified the text as follows:
> "In this study, all pathogens in the samples were known beforehand. For non-isolated samples, the pathogens were deliberately spiked to simulate real scenarios, while for isolated samples, prior identification ensured the pathogen's identity."
> This revised wording eliminates ambiguity and ensures consistency in describing the experimental setup. We appreciate your feedback in helping us improve the clarity of our manuscript.

Sentence starting "15 samples were incubated"; don't start sentence with number, it looks like 33.15

> Thank you for pointing this out. The sentence has been revised to avoid starting with a number. It now reads:
> "A total of 15 samples were incubated..."
> We appreciate your feedback in improving the readability of the manuscript.

How much meat did you use? What CFU/g does these ct values translate too? Its important to know the sensitivity relative to legislation. The limit is zero in 100 grams, but I don't assume you tested 100g?

> Thank you for your insightful comments. We have updated the text to clarify the experimental details and address your questions directly.
> This study primarily focused on developing and validating a bioinformatics workflow for characterising microbial species down to the subspecies rank within a food matrix using whole genome sequencing. The main objective was to enhance traceability for outbreak investigations rather than establish specific detection limits.
> To address your questions:
> - How much meat was used? Chicken samples of 25g each were used for the analysis, in line with the EN/ISO 6579 method for Salmonella detection.
> - What CFU/g do these Ct values translate to? This study did not directly calculate CFU/g from Ct values, as the primary goal was proof-of-concept testing of bioinformatics workflows. Instead, we spiked samples with known concentrations of Salmonella enterica (representing three different subspecies) to test the pipeline.
> The workflow was tested on both enriched and unenriched samples spiked with 2 different Salmonella

concentrations of 3 different Salmonella enterica subspecies:
> - Enriched samples were incubated for 24 hours following the EN/ISO 6579 protocol to exponentially increase the Salmonella concentration.
> - Unenriched samples (marked as "1h" in the manuscript) were processed immediately after preparation without an enrichment period. While we did not specifically define the processing time for unenriched samples, it included handling and immediate testing steps.
> We hope these clarifications sufficiently address your concerns. If further detail is needed, we are happy to expand the discussion.

What does adaptive sampling mean? To exclude chicken DNA?

> Thank you for pointing this out. We acknowledge that the term "adaptive sampling" should be clarified when it is first introduced in the manuscript. We have now revised the text to explicitly explain adaptive sampling when it is first mentioned. Specifically, we state that adaptive sampling is a feature of Nanopore sequencing that enables selective sequencing by enriching specific DNA regions of interest (e.g., microbial DNA) while depleting unwanted sequences (e.g., host DNA, such as chicken DNA). This clarification ensures that readers unfamiliar with the term understand its relevance and application.

The point v sentence under description of supplementary table t1 is a bit weird punctuation

> We have revised the sentence to make it clearer.

Gene-based pathogen identification

Working with meat to detect low abundance pathogenic bacteria is challenging without enrichment of the expected pathogen with selective methods. Just incubating it a x temperature might work for some bacteria, but others need special atmosphere (campylobacter, clostridia) and nutrients. How do you accommodate this?

> Thank you for pointing that out. We have updated the text to clarify that our workflows do not take experimental design into account. While we provide recommendations for interpretation, we do not factor in specific experimental conditions, such as temperature or atmosphere, in the analysis.

Figure 2 B:
The grey bares samples ? why are they collapsed in the left corner?

> Thank you for the questions. We have revised the figure legend and description to better explain the Clustergram visualisation, emphasising the role of the similarity slider in the clustering process and how the grey bars represent samples clustered based on metadata categories. Additionally, we have included a note about the interactive version available on the PathoGFAIR GitHub repository, where users can adjust the clustering granularity and explore the data further. This should help clarify the appearance of the collapsed samples and provide context for how users can engage with the figure.

And shy are sdhA and mucD highlighted?

> We highlighted sdhA and mucD because they were used as key examples in the main text to illustrate our findings and provide context for interpreting the results. Highlighting these genes allows readers to easily connect the visual representation to the specific examples discussed in the manuscript.

Also, please put genes in italics.

> Thank you for pointing that out. We have now italicised the gene names in both the text and the figures as requested.

the grey bars on the right (y-axis) are not annotated?

> The grey bars on the y-axis represent clusters of Virulence Factor (VF) genes, and due to the large

number of genes (over 2,100), annotating each one within the figure would be too cluttered. However, we have made sure to annotate and highlight the VF genes discussed as examples in the use case sections to provide clarity and context for the results.

To which reference genome are the barplot in d referring to? I can see for instance in f trs of genes hard to annotate them all, however, we annotated the ones we mention as examples in the use cases sections, explaining the results.
hat there is a number of snps or variants for the Houtenae and Typhimurium, but not Salamae, was the latter used as reference?

> Thank you for your comment. We used Salmonella enterica subsp. enterica serovar Typhimurium (NC_003197.2) as the reference genome for all analysis results of Workflow 4 on the first use case. The bar plots in panel D and the SNPs/variants in panel F were derived based on this reference genome. The observed differences in SNP numbers, particularly between Houtenae and Typhimurium, are largely influenced by the enrichment process (incubation period before sequencing).
> We chose Typhimurium as the reference genome due to its well-annotated and widely used genome in genomic studies, which allows for consistency across samples. However, we recognise that using a reference genome more closely related to the specific subspecies in the samples (e.g., Salmonella salamae) could potentially provide more accurate and comprehensive SNP detection results.
> In Workflow 4, selecting a reference genome is an input choice that users make before running the analysis. The Galaxy interface allows users to select any reference genome from a drop-down menu or upload their own reference genome if it is not available in the list. This flexibility ensures that the reference genome can be customized according to the user's specific use case and dataset requirements.

"an AIDA autotransporter-like protein, only found in Enterica strain samples but not in samples spiked with Houtenae or Salamae strains." All these strains are of the subspecies enterica

> Thank you for your comment. We have revised the sentence to clarify that all the mentioned strains belong to Salmonella enterica subsp. enterica, and the distinction was meant to highlight the presence of the AIDA autotransporter-like protein specifically in strains from Enterica (such as Typhimurium), but not in those spiked with Houtenae or Salamae strains. This revision has been applied throughout the manuscript for consistency and clarity.

Figure 3: punctuations a bit off here and there.

> The punctuation in the Figure 3 caption has been reviewed and corrected for clarity and consistency.

Why do you operate with cfu/ml? You added it to meat? It should be cfu/g?

> Meat was used as a classical background matrix, aligning with typical sample types analysed under ISO 6579 standards (products intended for human consumption). This method adheres to various European regulations, such as Regulation (EC) No 2160/2003 of the European Parliament and of the Council of 17 November 2003 on the control of Salmonella and other specified foodborne zoonotic agents, as well as Commission Regulation (EC) No 1688/2005 of 14 October 2005. The latter implements Regulation (EC) No 853/2004, providing special guarantees concerning Salmonella for consignments of certain meat and eggs to Finland and Sweden (Text with EEA relevance). The Regulation(EC) No 2160/2003 of the European Parliament e.g. says clearly: As regards poultry meat, the aim is to place on the market poultry meat with reasonable assurance that it is free from relevant salmonella. Similar statements can also be found in other European regulations.
> The concentration of Salmonella was determined in cfu/ml, as this refers to the resuspension of the centrifuged pellet in the enrichment broth. We understand the importance of reporting pathogen concentrations in cfu/g when working directly with meat, but in this case, the results were based on enrichment broth after resuspension."

It would be nice with a presentation of the resistance panel of the three spiked strains before presenting the amr genes.

> Thank you for your suggestion. The resistance profiles associated with the identified AMR genes are generated by our workflow and are available as part of the analysis outputs. These results, presented as a comprehensive table, are accessible via the output Galaxy history, which is publicly available on the PathoGFAIR GitHub repository (entry number 15291, https://usegalaxy.eu/api/datasets/4838ba20a6d86765e25ff74fab63aeed/display?to_ext=tabular).

"Similar but inverse relations are observed for CFU/mL value (Figure 3 C & D), with a threshold for VF and AMR gene detection at 106 ." cfu/ml of what? The rinse? Added ml? I don't even know how much meat were included in the dna extractions.

> The colony counts refer to the concentration of Salmonella per milliliter (cfu/ml) in the rinse solution after the enrichment process, which involves resuspending the centrifuged pellet from the enrichment broth. These values represent the microbial concentration in the liquid phase after enrichment, not the original concentration in the meat matrix. Regarding the amount of meat used in DNA extractions, approximately 25 mg of meat was included per aliquot for extraction.
> We have clarified this in the manuscript, specifying that the CFU/mL values refer to the bacterial concentration in the rinse solution and the amount of meat used for the DNA extraction process.

"The further the samples are from these thresholds, the higher the number of VF genes and AMR genes identified. Indeed, the three top scattered dots with identified VF genes between 250 and 300 (Figure 3 A, C, E) are the samples with the highest number of reads, higher CFU/mL value, and a relatively lower Ct value compared to other samples." The tendency is ok, but not all. For instance, you have several exceptions here for both amr genes and vf genes. Maybe mark the dots after say spiked strain/enrichment or not?

> We acknowledge that while the overall tendency shows a correlation between higher CFU/mL and lower Ct values with the number of identified VF and AMR genes, there are exceptions. To highlight this, we have marked the three top scattered dots in red in Figure 3, which correspond to the samples with the highest number of reads, higher CFU/mL values, and relatively lower Ct values. These dots represent specific cases mentioned in the manuscript for illustration.
> The variability in the data is due to several factors, such as sample type (spiked strain/enrichment) and other experimental conditions. We've clarified in the manuscript that although the general trend holds, exceptions do occur and that has been noted in the figure explanation.
> For consistency, the "Number of identified VF/AMR genes" is fixed on the y-axis across all sub-figures in Figure 3, so each data point stays in the same location vertically. The only variable changing across sub-figures is the x-axis, where we show either the ct or the CFU/ml value of each sample. Multiple dots are overlaid in the figure when there are similar numbers of identified genes, and the three red dots are used to illustrate the key points in our analysis.

Discussion bit here : "enerally, allowing samples to incubate for a short period before se quencing enhances microbial growth, resulting in higher CFU/mL values and lower Ct values. This increase in microbial concentra tion improves the efficiency of direct sequencing by providing more genetic material for analysis, facilitating faster and more accurate pathogen detection. "

> We appreciate your comment. Since we do not have a dedicated discussion section and are explaining the results within the use cases section, we believe this sentence is important for the observed trends in our data. The sentence reflects how incubation periods can influence microbial growth and sequencing efficiency, which is a key observation in our study.

Allele-based pathogen identification

"Salmonella enterica subspecies enterica serovar typhimarium (NC_003197.2)": see earlier comment on writing correct taxonomically for Salmonella.

> Thank you for pointing that out. The taxonomic naming has been corrected to reflect the proper classification for Salmonella enterica subsp. enterica serovar Typhimurium.

"However, given the diversity among Salmonella subspecies in the samples, a high number of complex variants and SNPs were anticipated. " You only operate with ONE subspecies of Salmonella - S. enterica subsp. enterica. That's the relevant subspecies, and contains over 2500 serovariants.

> You are correct that the term "subspecies" needs clarification. In our study, we focused on the diversity within serovariants of Salmonella enterica subsp. enterica. We have revised the sentence to reflect this and have ensured the consistent use of the correct throughout the manuscript.

I don't understand this process; in an outbreak setting you are dependent on tracing, i.e. showing that you isolates are clonal. Pathogfair relies on mapping to a reference genome, but that again relies on isolation of suspected isolate and building a high quality assembly for the allel-based pathogen identification to work. Its not enough to just show that you have that or that serotype, you will have to show that they are clonal (i.e. separated by a limited number of SNPs, say max 20 snps over the full length of the chromosome). This method cannot do this.

> We acknowledge that in an outbreak setting, establishing clonality is crucial for tracing the spread of pathogens, and this typically requires high-resolution techniques such as SNP-based phylogenetics. While our allele-based pathogen identification workflow maps to a reference genome and performs SNP calling, we understand that this alone may not be sufficient for detailed clonal tracking, where isolates are separated by only a few SNPs. As noted in previous responses and the conclusion section in the manuscript, our current approach does not focus on clonal tracing. To address this limitation, we plan to integrate Multi-Locus Sequence Typing (MLST) and expand SNP-based phylogenetics in a future iteration of the workflow, which will enable more robust clonal analyses.

Samples with prior pathogen isolation

Do understand you correctly that you now exstract dna from isolates? Not whole samples matrix? If so, how is this benchmarking a pipeline intended for metagenomics sequencing? If you were to extract dna from feces/ food and then use your pipeline, that would be benchmarking. However, this doesn't prove that your pipeline works as you intend it to/or claim that it does.

> Thank you for your question. We have now updated the manuscript to clarify the benchmarking process, which was conducted using the first use case (“samples without prior isolation”). The primary objective of having two use cases - one with isolates and the other with metagenomics samples - was to test PathoGFAIR’s capability to identify the expected pathogen from both types of input datasets, as long as they are initially nanopore sequenced.
> Previously, the term ‘Benchmark’ was incorrectly used. We have corrected this in the revised version and now only use the term “benchmarking” when comparing various pipelines using the samples without prior isolation datasets. In other instances, we refer to the analysis as ‘test analysis’. This distinction ensures that we are appropriately describing the purpose and context of each dataset

How were the samples prepared? If isolates, extraction method and sequencing techniques?

> Thank you for your question. As clarified in the manuscript, the 84 samples used in the second use case were provided by the Swiss Tropical and Public Health Institut in Switzerland. These samples were not prepared and sequenced as part of this project and publication. The DNA extraction and sequencing methods for these samples are described in the referenced publication, which is cited in the manuscript for further details. We have ensured that the data description is now more clearly outlined in the manuscript.

Species name is written non-capitalized first letter, so Campylobacter jejuni. All gene names should be italicized.

> Thank you for your feedback. The species names and gene names have been corrected to follow the proper formatting conventions: species names now have a capitalized first letter (e.g., Campylobacter jejuni), and all gene names are italicized throughout the manuscript.

Suggest rewriting sentence: The wet lab procedures performed to isolate and prepare these samples for

sequencing adhered to standard microbiological techniques, including cultivation, enrich ment, and isolation steps" to reflect actual sequel; enrichment, cultivation and isolation and verification."

> Thank you for your suggestion. We have removed the sentence as the details of the wet lab procedures, including enrichment, cultivation, isolation, and verification, are already described in the original paper.

Conclusion

If for use for solely isolates, I think assemblies are a better way to go than this pipeline; its more reliable for clonality analysis needed in outbreaks.

> We agree that high-quality assemblies are essential for clonality analysis, especially in outbreak situations. PathoGFAIR does include assembly generation in Workflow 3, where users can adjust tool parameters to optimise the assemblies according to their input datasets. This flexibility allows for more accurate clonal analysis, complementing the allele-based pathogen identification approach for cases where assembly-based methods are preferred.

"We further supported the scientific community by introducing new 46 benchmark samples, making them publicly available. This demonstrates our significant investment of time and resources, providing valuable assets for future research." There are now 82000 c. jejuni just on ncbi, of which 600 are complete. Salmonella genomes are clocking on 524500 assemblies on enterobase. The contribution of these strains are not because they are new samples, but because your isolates represent data from an underrepresented region of the world, namely Palestine.

> The 86 samples from Palestine, provided by the Swiss Tropical and Public Health Institute, are indeed valuable due to their representation of data from an underrepresented region of the world. These samples were not prepared specifically for our study, but have been referenced in the manuscript with appropriate citations.
> As for the 46 samples without prior isolation, spiked with 3 different subspecies of S. enterica, these were prepared by us to test the PathoGFAIR's workflows, and the generated data is publicly available on NCBI. Additionally, with the revised version of the manuscript, we have created a protocol (available on protocols.io) to benchmark PathoGFAIR alongside other similar pipelines using these 46 samples.

Supplmentary figure s4 is cropped so that x-line annotation is not visible.

> Thank you for pointing that out. We have reviewed and resubmitted Supplementary Figure S4, ensuring that all x-line annotations are visible.

SFigure 5 Midpoint root amr phylogenetic tree?

> Yes, the tree in Supplementary Figure 5 is a midpoint-rooted AMR phylogenetic tree.

Supplementary table 1: its unclear for me if you added this amount of bacteria or it was the result of after 1h or 24h enrichment. Also, I don't understand how much meat you used for the dna extraction. Same goes for ct values.

> The metadata for the 46 samples without prior isolations includes the bacterial concentration after enrichment, pelleting, and resuspension steps. The samples were spiked with one of three subspecies of S. enterica, and the incubation time is indicated in column T. For DNA extraction, 25g of spiked chicken meat was used per sample.

Cookie Preference Center

We use cookies which are necessary to make our site work. We may also use additional cookies to analyze, improve and personalize our content and your digital experience. For more information, see our Cookie Policy.

You may choose not to allow some types of cookies. However, blocking some types may impact your experience of our site and the services we are able to offer. See the different category headings below to find out more or change your settings.

Allow all

Manage Consent Preferences

Strictly Necessary Cookies

Always active

These cookies are necessary for the website to function and cannot be switched off in our systems. They are usually only set in response to actions made by you which amount to a request for services, such as setting your privacy preferences, logging in or filling in forms. You can set your browser to block or alert you about these cookies, but some parts of the site will not then work.

Functional Cookies

These cookies enable the website to provide enhanced functionality and personalisation. They may be set by us or by third party providers whose services we have added to our pages. If you do not allow these cookies then some or all of these services may not function properly.

Performance Cookies

These cookies allow us to count visits and traffic sources so we can measure and improve the performance of our site. They help us to know which pages are the most and least popular and see how visitors move around the site.

Targeting Cookies

These cookies may be set through our site by our advertising partners. They may be used by those companies to build a profile of your interests and show you relevant adverts on other sites. If you do not allow these cookies, you will experience less targeted advertising.

Cookie List

Clear

Apply Cancel
Consent Leg.Interest

Confirm my choices

Powered by onetrust
