# Supplementary material for: PathoGFAIR: a collection of FAIR and adaptable (meta)genomics workflows for (foodborne) pathogens detection and tracking
Source: Gigascience. 2025 Sep 26;14:giaf017. doi: 10.1093/gigascience/giaf017 (PMC12466118; doi:10.1093/gigascience/giaf017)
Supplement: giaf017_Reviewer_1_Report_Original_Submission — Federico Zambelli -- 7/31/2024 [file giaf017_reviewer_1_report_original_submission.pdf]

Reviewer Report

Title: PathoGFAIR: a collection of FAIR and adaptable (meta)genomics workflows for (foodborne) pathogens detection and tracking

Version: Original Submission **Date:** 7/31/2024

Reviewer name: Federico Zambelli

Reviewer Comments to Author:

The authors present PathoGFAIR, a set of Galaxy workflows for the metagenomic analysis of shotgun Nanopore sequencing from isolated and non-isolated pathogens in contaminated food samples. They complement their work by analysing and releasing two datasets, one from isolated and the other from non-isolated samples, with the primary objective of illustrating the potentiality of the workflows. These datasets could also be used as benchmarks for future works.The manuscript is generally well-written, and the authors highlight the advantages of the proposed workflows in Table 1 by comparing them to similar solutions. The workflows are well integrated into the Galaxy network, are available on the three main usegalaxy instances, and provide a thorough tutorial through the Galaxy training platform. A notable advantage of PathoGFAIR over similar workflows is that, thanks to Galaxy, the final user can easily tailor them by replacing any tool in the workflow with others available in the Galaxy ecosystem. This also allows easy updates for the tools in the workflows.A few minor points that, if addressed, in my opinion, could further strengthen the manuscript:1 - The rationale behind the tool selection in each of the four workflows is not always clear. While insights are present for workflows 1 and 4, this is not true for workflows 2 and 3. The reader would benefit from understanding why one tool has been preferred over another for the same task, even more so, given the possibility to modify the workflows easily, when this preference could be the other way around in particular use cases or conditions.2â€”One of the main factors for a successful metagenomic analysis is the correctness, completeness, and up-to-dateness of the reference data. The authors should briefly describe how PathoGFAIR addresses this in Galaxy.3â€”While this workflow is clearly stated to be tailored for shotgun metagenomic sequencing, the authors contrast this approach only with targeted sequencing. Instead, they should also discuss the 16s rRNA metagenomic approach, for which Nanopore kits are available, and why PathoGFAIR has been limited to the analysis of shotgun data.

Level of Interest

Please indicate how interesting you found the manuscript: Choose an item.

Quality of Written English

Please indicate the quality of language in the manuscript: Choose an item.

Declaration of Competing Interests

Please complete a declaration of competing interests, considering the following questions:

- Have you in the past five years received reimbursements, fees, funding, or salary from an organisation that may in any way gain or lose financially from the publication of this manuscript, either now or in the future?
- Do you hold any stocks or shares in an organisation that may in any way gain or lose financially from the publication of this manuscript, either now or in the future?
- Do you hold or are you currently applying for any patents relating to the content of the manuscript?
- Have you received reimbursements, fees, funding, or salary from an organization that holds or has applied for patents relating to the content of the manuscript?
- Do you have any other financial competing interests?
- Do you have any non-financial competing interests in relation to this paper?

If you can answer no to all of the above, write 'I declare that I have no competing interests' below. If your reply is yes to any, please give details below.

I declare that I have no competing interests

I agree to the open peer review policy of the journal. I understand that my name will be included on my report to the authors and, if the manuscript is accepted for publication, my named report including any attachments I upload will be posted on the website along with the authors' responses. I agree for my report to be made available under an Open Access Creative Commons CC-BY license (http://creativecommons.org/licenses/by/4.0/). I understand that any comments which I do not wish to be included in my named report can be included as confidential comments to the editors, which will not be published.

Choose an item.

To further support our reviewers, we have joined with Publons, where you can gain additional credit to further highlight your hard work (see: https://publons.com/journal/530/gigascience). On publication of this paper, your review will be automatically added to Publons, you can then choose whether or not to claim your Publons credit. I understand this statement.

Yes Choose an item.
