# Supplementary material for: PathoGFAIR: a collection of FAIR and adaptable (meta)genomics workflows for (foodborne) pathogens detection and tracking
Source: Gigascience. 2025 Sep 26;14:giaf017. doi: 10.1093/gigascience/giaf017 (PMC12466118; doi:10.1093/gigascience/giaf017)
Supplement: giaf017_Reviewer_2_Report_Original_Submission — Ann-Katrin Llarena -- 9/9/2024 [file giaf017_reviewer_2_report_original_submission.pdf]

Reviewer Report

Title: PathoGFAIR: a collection of FAIR and adaptable (meta)genomics workflows for (foodborne) pathogens detection and tracking

Version: Original Submission **Date:** 9/9/2024

Reviewer name: Ann-Katrin Llarena

Reviewer Comments to Author:

Nasr and colleagues present an, at times, well-written manuscript with an interesting and robust pipeline that includes well-known softwares (you must make sure to cite the authors of these). However, the manuscript is, quote "...a collection of Galaxy-based FAIR workflows employing state-of-the-art tools to detect and track pathogens from metagenomic Nanopore sequencing". Its repeated how well it works, they even compare it to other software in table 1 (without proper benchmark). These initial statements are however not supported by the findings. The Salmonelal from the spiked samples are, as expected from food matrix present in low quantity), difficult to do more than state that the genus is present, and only a fraction of the samples can actually "complete" the entire pipeline. Also, the benchmarking is not really benchmarking (compare and measure this software against other competing software). No such comparison is done, and even though the intention of PathoGFAIR as stated throughout the paper, is detection and analysis of metagenomic samples, the benchmarking is done on isolate based wgs.It is also evident that the authors are not microbiologists as the manuscript is riddled with taxonomical misunderstandings about the vast genus Salmonella and when to use capital letters and italics. I am also lacking a proper discussion here on the results found in the spiking experiment in light of current EU legislation on Salmonella. Can this pipeline help in this regard? Sensitivity and specificity metrics are also lacking.Abstract:"foodborne pathogen data" / "metagenomic Nanopore pathogenic data" - suggest to rewrite, as what I think you are trying to say is " initially developed to detect foodborne pathogens from metagenomic nanopore data, the workflow can be used to detect any pathogen.""Colony-forming unit and Cycle Threshold values." rewrite sentence, I do not completely understand what you are trying to say. what is "sufficient colony forming units?" It will vary as well between pathogens (infection dose varies). You could rather state your sensitivity of the pipeline here - even though i think that sampling prep, library prep and seq influences that more than the bioinformatics."In any sample": did you test all matrixes?"sample is isolated or incubated before seq" you cannot isolate a sample, but you isolate a bacteria from a sample. unprecise language.Introduction:In general, its well written, but a bit unprecise here and there. The authors also rely a lot on the following words: "rapid" "accurate"."outbreaks and epidemics" - rewrite, these are the same."efforts to mitigate their spread and ensure food safety" again, complementary terms - rewrite."global public health authorities" we do have everything from local to global food safety and public health authorities, I think one should highlight this. There is a difference between for instance EFSA and ECDC."isolation can be complex"? do you mean complicated or work intensive?"The utilisation of Nanopore sequencing data, as exemplified in studies like [7]," citing practices like this is not really reader friendly. Suggest to write what they actually did in seven (as for instance the detection of blah in

blah as shown in 7)."Once (meta)genomics data has been generated, bioinformatics approaches enable the rapid and accurate detection"; repetition of chapter above. You write in the former chapter that "the utilisation of nanopore data" which also includes bioinformatics of course.SURPI and Sunbeam is freely available? https://microbiomejournal.biomedcentral.com/articles/10.1186/s40168-019-0658-xhttps://chiulab.ucsf.edu/surpi/"PathoGFAIR: pathogen identification and tracking from metagenomics". Im not convinced that it can perform tracing in an outbreak where only a few SNPs are allowed.PathoGFAIR does not really speed up the process of sampling, does it. Actually, it takes more time to extract crude dna from a sample than to place it in a enrichment broth or do a dilution series, so the presteps are not really a part of this."Tracking pathogens" - again, if species level is the lowest rank it can go to, its not enough to perform tracking.Overview chapter"input data is seq data generated w nanopore" basecalling is not included in the workflow? How is this performed? It affects the quality of the reads, so its nice to know what you did.The chapter is very wordy, and contains a lot of fill-words with salespitches almost. I would recommend rewriting it, for instance:Chapter that starts with subsequently and describes the different workflows and how they work together can be compressed.And the last three sections are salespitching.WF1: Preprocessing:How stringent filtering and quality control are implemented in the workflow? How good quality do you need for the wp2-4 to work sufficiently well? Did you test?Food vehicle animal? What is that - do you mean that if you extract dna from bovine meat, you map to bovine genome?"a tool ten times faster etc etc." is discussion and should be removed from what I think is materials and methods even though the title of the section is workflow 1.What is a food host?Kalamari database includes many foodborne pathogens, such as Shigella, E. coli, Campylobacter etc etc. how can you just remove all reads that match to this database?Table 1: Innuendo is based on isolate WGS, and not intended for WGS. Also, it has its own built in wgMLST schema employed using chewbbacca, so it definitely has allele-abased pathogen identification. Its intended for illumina data. Victors are strictly a platform to analyse virulence factors and not intended even for taxonomic profiling, and its webinterface doesn't work. IDseq has step-by-step guides available on their webpage, so I think that qualifies as a tutorial. You can also contact them (user support). I guess the same is true for OneCodex, as you actually pay for that one. So the table is unprecise at best and should be corrected (I didn't go through Submeam, SURPI or PAIPline specs to try to check if you got it correctly). Rewrite this. Further, I think you should only include systems / pipelines that are intended for metagenomics. You have a footnote * that I cannot see in the table as well.WF2 taxonomy profiling:The first sentence needs rewriting.Two sentences from "Although Kraken2 is a tool design…….." belongs in discussion.WF3:Medaka consensus pipeline : "This task is performed using neural networks applied from a pileup of individual sequencing reads against a draft assembly. " what draft assembly did you use here to create a consensus sequence? Actually, its not polishing contigs, its assemblying them?Again, there is some descriptions of the software which belongs in the discussion, say the perks one gets from using this tool over the other.I do not however get how screening for virulence genes = pathogen identification. The thing is that in a complex food matrix or faecal samples from animals, things like stx phages will also be present. These are not stec pathogens unless the phage is inside an e.coli. How do you make sure of the host for such mobile genetic elements as these virulence and amr genes often are located on? Seeing as this is the basis of your pathogen detection?WF4:A bit again on choosing software over the other that is discussion food.Wf4/wf5:I am worried about the reliance on snp based technics for nanopore reads. Is the quality good enough to achieve sufficiently robust results?Easily adaptable

workflowsLast section is repetition (about each wf operating independently)Use cases:Data generation:Please revise how to write Salmonella names correctly. They should be in italics for genus, species and subspecies names, while the serovar/serotype is non italic and capital letter. So the correct term would be:* Salmonella enterica subsp. enterica serovar Houtenae, or in short; Salmonella Houtenae.* The strain DSM554 is of serovar Typhimurium, and this should referenced like this: Salmonella enterica subsp. enterica serovar Typhimurium strain DSM 554First two sentences are contradictory to eachother?Sentence starting "15 samples were incubated"; don't start sentence with number, it looks like 33.15How much meat did you use? What CFU/g does these ct values translate too? Its important to know the sensitivity relative to legislation. The limit is zero in 100grams, but I don't assume you tested 100g?What does adaptive sampling mean? To exclude chicken DNA?The point v sentence under description of supplementary table t1 is a bit weird punctuationGene-based pathogen identification:Working with meat to detect low abundance pathogenic bacteria is challenging without enrichment of the expected pathogen with selective methods. Just incubating it a x temperature might work for some bacteria, but others need special atmosphere (campylobacter, clostridia) and nutrients. How do you accommodate this?Figure 2 B:The grey bares samples ? why are they collapsed in the left corner? And shy are sdhA and mucD highlighted? Also, please put genes in italics.the grey bars on the right (y-axis) are not annotated?To which reference genome are the barplot in d referring to? I can see for instance in f that there is a number of snps or variants for the Houtenae and Typhimurium, but not Salamae, was the latter used as reference?"an AIDA autotransporter-like protein, only found in Enterica strain samples but not in samples spiked with Houtenae or Salamae strains." All these strains are of the subspecies entericaFigure 3: punctuations a bit off here and there.Why do you operate with cfu/ml? You added it to meat? It should be cfu/g?It would be nice with a presentation of the resistance panel of the three spiked strains before presenting the amr genes."Similar but inverse relations are observed for CFU/mL value (Figure 3 C & D), with a threshold for VF and AMR gene detection at 106 ." cfu/ml of what? The rinse? Added ml? I don't even know how much meat were included in the dna extractions."The further the samples are from these thresholds, the higher the number of VF genes and AMR genes identified. Indeed, the three top scattered dots with identified VF genes between 250 and 300 (Figure 3 A, C, E) are the samples with the highest number of reads, higher CFU/mL value, and a relatively lower Ct value compared to other samples." The tendency is ok, but not all. For instance, you have several exceptions here for both amr genes and vf genes. Maybe mark the dots after say spiked strain/enrichment or not?Discussion bit here : "enerally, allowing samples to incubate for a short period before se quencing enhances microbial growth, resulting in higher CFU/mL values and lower Ct values. This increase in microbial concentra tion improves the efficiency of direct sequencing by providing more genetic material for analysis, facilitating faster and more accurate pathogen detection. "Allele-based pathogen identification:"Salmonella enterica subspecies enterica serovar typhimarium (NC_003197.2)": see earlier comment on writing correct taxonomically for Salmonella."However, given the diversity among Salmonella subspecies in the samples, a high number of complex variants and SNPs were anticipated. " You only operate with ONE subspecies of Salmonella - S. enterica subsp. enterica. That's the relevant subspecies, and contains over 2500 serovariants.I don't understand this process; in an outbreak setting you are dependent on tracing, i.e. showing that you isolates are clonal. Pathogfair relies on mapping to a reference genome, but that again relies on isolation of suspected isolate and building a high quality assembly for the allel-based pathogen identification to work. Its not enough to just show

that you have that or that serotype, you will have to show that they are clonal (i.e. separated by a limited number of SNPs, say max 20 snps over the full length of the chromosome). This method cannot do this.Samples with prior pathogen isolation:Do understand you correctly that you now exstract dna from isolates? Not whole samples matrix? If so, how is this benchmarking a pipeline intended for metagenomics sequencing? If you were to extract dna from feces/ food and then use your pipeline, that would be benchmarking. However, this doesn't prove that your pipeline works as you intend it to/or claim that it does.How were the samples prepared? If isolates, extraction method and sequencing techniques?Species name is written non-capitalized first letter, so Campylobacter jejuni. All gene names should be italicized.Suggest rewriting sentence: The wet lab procedures performed to isolate and prepare these samples for sequencing adhered to standard microbiological techniques, including cultivation, enrich ment, and isolation steps" to reflect actual sequel; enrichment, cultivation and isolation and verification."Conclusion:If for use for solely isolates, I think assemblies are a better way to go than this pipeline; its more reliable for clonality analysis needed in outbreaks."We further supported the scientific community by introducing new 46 benchmark samples, making them publicly available. This demonstrates our significant investment of time and resources, providing valuable assets for future research." There are now 82000 c. jejuni just on ncbi, of which 600 are complete. Salmonella genomes are clocking on 524500 assemblies on enterobase. The contribution of these strains are not because they are new samples, but because your isolates represent data from an underrepresented region of the world, namely Palestine.Supplmentary figure s4 is cropped so that x-line annotation is not visible.SFigure 5 Midpoint root amr phylogenetic tree?Supplementary table 1: its unclear for me if you added this amount of bacteria or it was the result of after 1h or 24h enrichment. Also, I don't understand how much meat you used for the dna extraction. Same goes for ct values.

Level of Interest

Please indicate how interesting you found the manuscript: Choose an item.

Quality of Written English

Please indicate the quality of language in the manuscript: Choose an item.

Declaration of Competing Interests

Please complete a declaration of competing interests, considering the following questions:

- Have you in the past five years received reimbursements, fees, funding, or salary from an organisation that may in any way gain or lose financially from the publication of this manuscript, either now or in the future?
- Do you hold any stocks or shares in an organisation that may in any way gain or lose financially from the publication of this manuscript, either now or in the future?
- Do you hold or are you currently applying for any patents relating to the content of the manuscript?

- Have you received reimbursements, fees, funding, or salary from an organization that holds or has applied for patents relating to the content of the manuscript?
- Do you have any other financial competing interests?
- Do you have any non-financial competing interests in relation to this paper?

If you can answer no to all of the above, write 'I declare that I have no competing interests' below. If your reply is yes to any, please give details below.

I declare that I have no competing interests

I agree to the open peer review policy of the journal. I understand that my name will be included on my report to the authors and, if the manuscript is accepted for publication, my named report including any attachments I upload will be posted on the website along with the authors' responses. I agree for my report to be made available under an Open Access Creative Commons CC-BY license (http://creativecommons.org/licenses/by/4.0/). I understand that any comments which I do not wish to be included in my named report can be included as confidential comments to the editors, which will not be published.

Choose an item.

To further support our reviewers, we have joined with Publons, where you can gain additional credit to further highlight your hard work (see: https://publons.com/journal/530/gigascience). On publication of this paper, your review will be automatically added to Publons, you can then choose whether or not to claim your Publons credit. I understand this statement.

Yes Choose an item.
